# A robust high-performance H_2_S sensor enabling real-world wireless monitoring

**DOI:** 10.1038/s41467-026-76707-w

**Published:** 2026-08-19

**Authors:** Wei Wang, Handan Wang, Leif Riemenschneider, Chenchen Wang, Juntang Dong, Shivam Singh, Yana Vaynzof, Darius Pohl, Markus Löffler, Helin Sun, Lin He, Anping Dong, Luis Antonio Panes-Ruiz, Bernd Rellinghaus, Chuanhui Huang, Shirong Huang, Gianaurelio Cuniberti

**Affiliations:** 1 Institute for Materials Science and Max Bergmann Center for Biomaterials, TUD Dresden University of Technology, Dresden, Germany; 2 Cluster of Excellence CARE, TU Dresden and RWTH Aachen, Aachen, Germany; 3 School of Materials Science and Engineering, Shanghai Jiao Tong University, Shanghai, China; 4 School of Physics and Electronic Information, Yan’an University, Yan’an, China; 5 Leibniz Institute for Solid State and Materials Research (IFW), Dresden, Germany; 6 Chair for Emerging Electronic Technologies, TUD Dresden University of Technology, Dresden, Germany; 7 Dresden Center for Nanoanalysis (DCN), Center for Advancing Electronics Dresden (cfaed), TUD Dresden University of Technology, Dresden, Germany; 8 Hangzhou Institute of Medicine (HIM), Chinese Academy of Sciences, Hangzhou, China; 9 Dresden Center for Computational Materials Science (DCMS), TUD Dresden University of Technology, Dresden, Germany; 10 Cluster of Excellence CeTI, TU Dresden, Germany

**Keywords:** Nanoscale devices, Sensors and biosensors

## Abstract

Hydrogen sulfide (H_2_S) is a highly toxic and corrosive gas that requires continuous, real-time monitoring at ultra-low concentrations in complex environments. Here we report a wireless, ultra-low-power H_2_S sensing platform for long-term, on-site detection. The device achieves a response exceeding 10,000%, a calculated detection limit of 0.328 ppb, and a 7 s response at room temperature, while consuming less than 0.7 μW. This performance represents a 4–5-fold faster response and up to 10^5^-fold lower active sensing power than commercial H_2_S sensors. The Au-interfaced MOF-derived architecture synergistically modulates Schottky barriers and grain boundaries, producing a 0.4 eV Schottky barrier shift and increasing current density by more than 10^6^-fold. We further demonstrate practical monitoring of food spoilage, pipeline leakage, and H_2_S accumulation in confined industrial environments, highlighting a robust strategy for next-generation smart environmental monitoring.

## Abstract (web summary)

The authors report a wireless, ultra-low-power sensor that detects trace hydrogen sulfide rapidly at room temperature. The device also enables long-term monitoring of food spoilage, pipeline leaks, and industrial gas accumulation

## Introduction

Hydrogen sulfide (H_2_S) is a highly toxic^1,2^, corrosive^2^, and flammable^3^ gas that is widely produced in natural and anthropogenic processes^2,4^, including petroleum refining^5^, wastewater treatment^6,7^, mining^3,8^, paper pulping^9,10^, and food storage^11,12^. At concentrations as low as a few parts per million, H_2_S can cause severe irritation of the eyes and respiratory tract^13,14^, while acute exposure at higher concentrations can result in respiratory paralysis or death^14,15^. Its corrosive nature accelerates the degradation of metals and electronic components, posing additional risks to industrial infrastructure. Furthermore, the characteristic odor of H_2_S is an unreliable indicator at elevated concentrations due to olfactory fatigue, making sensory detection ineffective^2,4,16^. Consequently, accurate, sensitive, and real-time monitoring of H_2_S is essential for ensuring workplace safety, preventing environmental contamination, and maintaining the quality of perishable goods.

A range of sensing technologies—such as electrochemical cells, optical spectroscopies, and resistive devices—has been explored for H_2_S detection^17^. Resistive sensors are particularly attractive due to their low cost, scalable fabrication, and compatibility with integrated electronics^18,19^. Metal oxide semiconductors (MOS), such as ZnO^20,21^, SnO_2_^22^, Fe_2_O_3_^23^, CuO/Nb_2_O_5_^24^ and WO_3_^25^, have long served as effective resistive-type gas sensing materials due to their chemical stability, low cost, and tunable surface reactivity^26–30^. However, a major challenge for conventional metal oxide sensors is their limited sensitivity at room temperature. This limitation arises because their sensing mechanism typically relies on chemisorbed oxygen species interacting with target gases^31–34^, which requires elevated temperatures (≥200 °C) to activate surface reactions^35–37^. While such high-temperature operation is effective for enhancing response, it results in increased power consumption and restricts the sensor’s applicability in oxygen-deficient environments. Consequently, challenges remain in realizing ultra-low detection limits, stable long-term performance, and reliable operation under complex, real-world conditions.

In practical application scenarios, such as anaerobic food preservation systems and enclosed industrial environments (e.g., storage tanks, pipelines, and confined workspaces), the monitoring requirements for H_2_S are particularly stringent^38,39^. In anaerobic or low-oxygen food preservation conditions, trace-level H_2_S released during early-stage spoilage must be detected under high humidity and limited oxygen availability, where conventional metal-oxide sensors often exhibit sluggish response, poor reversibility, and elevated power consumption. In these settings, the elevated operating temperatures commonly required by traditional metal-oxide sensors not only increase power consumption but also pose safety risks and limit long-term, battery-powered deployment. Moreover, the reliance on oxygen-assisted surface reactions and unstable interfacial charge transport frequently leads to signal drift and reduced reliability under low-oxygen or confined conditions^40–42^. These practical constraints highlight the need for H_2_S sensors that meet stringent real-world monitoring requirements, including room-temperature operation, ultra-low power consumption, and stable performance under low-oxygen and confined conditions. Addressing these application-driven challenges requires a sensing mechanism that fundamentally decouples performance from thermal activation and instead relies on robust interfacial electronic modulation.

Metal-organic framework (MOF)-derived metal oxides have emerged as attractive platforms for gas sensing owing to their intrinsically high specific surface area and, more importantly, the ability to tailor microstructural features through controlled thermal conversion^43–45^. Previous studies have shown that the pyrolysis of MOF precursors enables precise regulation of grain size, porosity, and GB density in the resulting metal oxides, providing a versatile route to engineer charge transport pathways and surface reactivity. However, despite these advantages, the role of GB density as an active and tunable parameter governing gas-sensing performance has rarely been systematically explored. In parallel, Schottky interfaces between metals and semiconducting oxides are well known to amplify gas-sensing signals by converting surface adsorption-induced electronic perturbations into exponential changes in carrier transport^46–48^. Despite these advances, the GB density has rarely been treated as an active and tunable physical parameter governing Schottky barrier formation and dynamic barrier modulation during gas adsorption. Unlike defect-engineering strategies that primarily target local point defects and adsorption chemistry^20^, GB engineering addresses mesoscopic transport regulation and interfacial Schottky barrier formation/modulation, providing a fundamentally different route for mechanism-driven performance optimization. As a result, how microstructural parameters interact with interfacial electronic transport to determine sensing performance remains insufficiently understood. Motivated by this gap, we identify GB density as a decisive control knob that simultaneously governs the initial Schottky barrier and its modulation efficiency, enabling a mechanism-driven optimization of H_2_S sensing performance.

To overcome these challenges, we draw on the established role of grain boundaries (GBs) in polycrystalline semiconductors to modulate carrier transport and surface reactivity. By deliberately tuning the grain-boundary density in ZnO, additional active sites and controllable carrier pathways are formed, enabling room-temperature operation at low power. In our implementation, MOF precursors are used as a convenient route to obtain polycrystalline ZnO with well-controlled microstructures; this structural control improves charge transport and enables rational modulation of Schottky barriers, effectively combining the microstructural tunability associated with MOF-derived routes with the robust electronic functionality of metal-oxide semiconductors. This approach provides a practical platform for advanced resistive H_2_S sensing under real-world conditions.

While MOF-derived metal oxides have been widely explored for H_2_S sensing, most previous studies primarily focus on material composition optimization or morphological engineering. In contrast, the novelty of this work does not lie in the use of MOF-derived ZnO itself, but in identifying GB density as a decisive and tunable physical parameter that governs both the initial Schottky barrier and its dynamic modulation during gas adsorption. By leveraging the temperature-sensitive microstructural evolution inherent to MOF-derived ZnO, we establish a quantitative correlation between GB density, barrier modulation efficiency, and sensing performance. This mechanism-oriented strategy enables simultaneous enhancement of sensitivity and power efficiency without relying on complex structural designs, offering a generalizable pathway for high-performance H_2_S sensing.

Here, we present a resistive H_2_S sensor based on MOF-derived polycrystalline ZnO, in which engineered GBs and Au-ZnO Schottky interfaces are deliberately designed to modulate charge transport pathways and adsorption kinetics. The polycrystalline structure provides a high density of active GBs that act as efficient adsorption and carrier scattering centers, while the Schottky junctions at the Au contact interfaces enable tunable barrier heights for selective gas modulation. This unique structural and electronic synergy allows the sensor to operate at room temperature with an ultra-low power demand of <0.7 μW, overcoming the milliwatt-to-watt consumption typical of commercial heated metal-oxide sensors. Performance-wise, the device achieves an extraordinary sensing response exceeding 10,000%, a calculated detection limit of 0.328 ppb, and a rapid 7 s response, surpassing both laboratory prototypes and commercial benchmarks that typically require ≥30 s response times and tens of milliwatts of power. The sensor also demonstrates excellent selectivity against common interfering gases and long-term operational stability under atmospheric conditions, highlighting its robustness for real-world deployment.

Beyond laboratory validation, the sensor has been successfully integrated with a low-power wireless module, enabling real-time monitoring in diverse scenarios, including (i) detecting anaerobic H_2_S emissions as indicators of food spoilage, (ii) tracking H_2_S leakage in industrial pipelines and chemical plants, and (iii) assessing environmental air quality in urban and confined environments. Collectively, these results highlight a comprehensive sensing strategy that unites ultra-low power operation, high sensitivity, rapid response, and environmental stability—paving the way toward scalable, next-generation H_2_S monitoring systems for food safety, industrial protection, and environmental sustainability.

## Results

### Sensor fabrication and performance

**Fig. 1:**
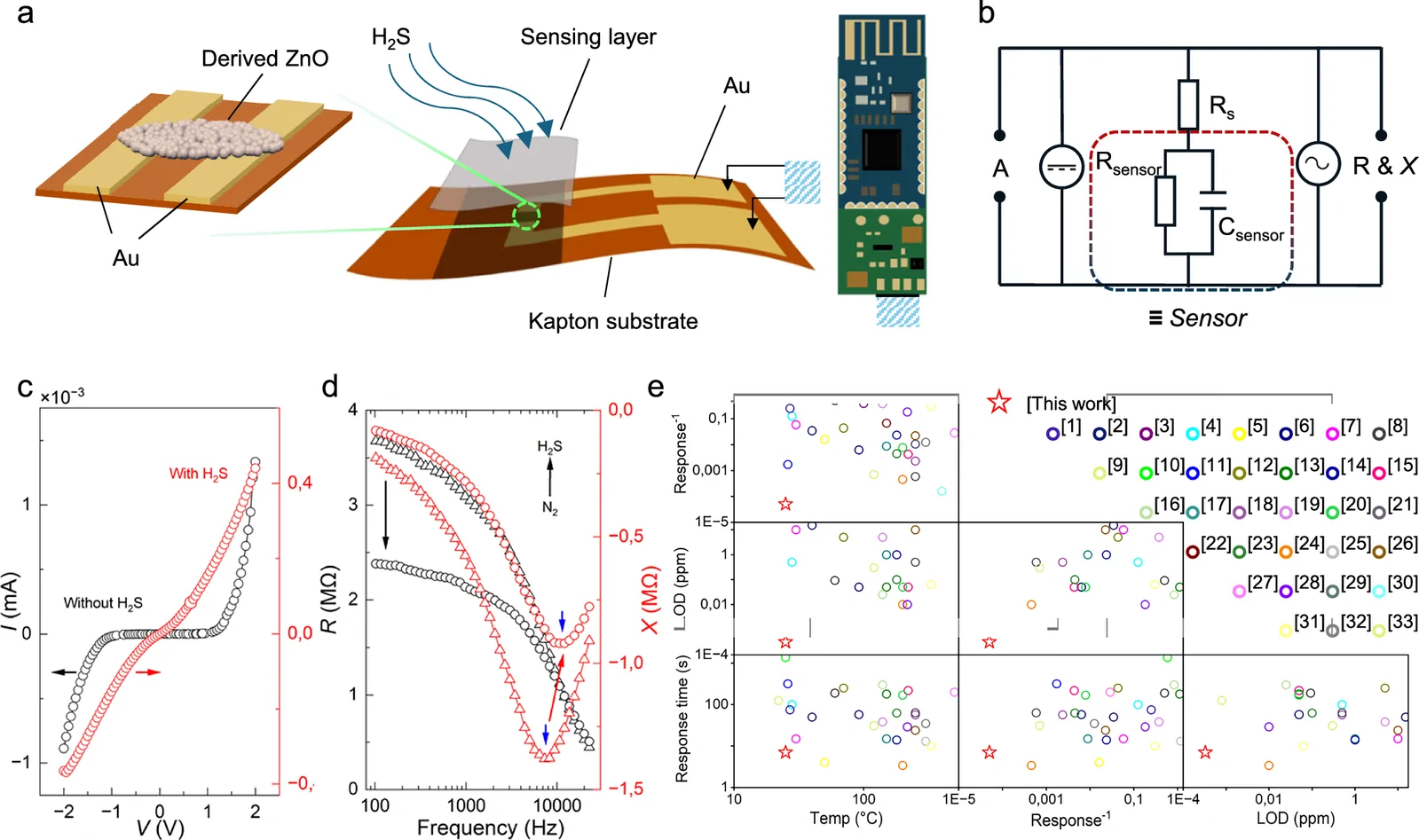
Device architecture and performance metrics of the MOF-derived wireless H_2_S sensor. **a** Schematic illustration of the wireless sensing system, comprising a MOF-derived sensing layer deposited on Au interdigitated electrodes (thickness, 65 nm) supported by a Kapton substrate, integrated with a wireless data acquisition and transmission module. **b** Equivalent circuit model of the sensor: a parallel RC unit connected in series with a resistor (Rs) for DC signal acquisition and for resistance and impedance measurements. **c** Current-voltage (I-V) characteristics before and after exposure to H_2_S. **d** Frequency-dependent resistance and reactance curves of the sensor before and after H_2_S exposure. **e** Comparative performance benchmarking of this work against previous reports, showing key metrics including operating temperature, response magnitude, response time, and LOD.

The architecture and superior performance of the wireless H_2_S sensing device are illustrated in Fig. 1. As shown in Fig. 1a, the sensor consists of a flexible Kapton substrate, 65 nm-thick Au IDE, and a top sensing layer formed by uniformly dispersed MOF-derived ZnO semiconductor material. The Au electrode pads are inserted into the port of a compact, lightweight circuit module, creating a fully integrated wireless smart sensing device. Upon exposure to the target gas H_2_S, electrical signals generated by the sensing layer—via electrodes A and B—are recorded to evaluate sensor performance. Furthermore, resistance changes induced by H_2_S interaction are measured, processed, and wirelessly transmitted via a built-in system integrating an analog-to-digital converter (ADC), microprocessor, and Bluetooth module, enabling real-time remote monitoring of H_2_S under ambient conditions. The gas sensor can be effectively modeled as a parallel resistor-capacitor (RC) circuit (Fig. 1b). To characterize the current-voltage (I-V) behavior, a direct current (DC) source and sampling resistor (Rs) are connected in series and placed in parallel with the sensor. The frequency-dependent impedance was measured with an impedance analyzer (Zurich Instruments MFIA) with an excitation voltage of 0.5 V.

The underlying charge transport mechanism at the material interface fundamentally governs the exceptional sensing performance. The inherent work function difference between Au electrodes (~4.7 eV) and ZnO establishes a Schottky barrier at their interface. This barrier effectively restricts electron transfer from ZnO to Au, maintaining elevated sensor resistance in the baseline state, as evidenced by the characteristic non-linearity in the current-voltage (I-V) characteristics before H_2_S exposure. Upon H_2_S interaction, a significant modulation of the interfacial barrier occurs, manifested by the transition to near-ohmic behavior under forward bias conditions (Fig. 1c). This transition indicates substantial barrier lowering, facilitating enhanced electron injection from ZnO to Au. The consequent reduction in sensor resistance generates the characteristic response signal. The underlying mechanism of this behavior lies in H_2_S-induced surface charge modification, which alters the local electronic environment at the ZnO surface. Following H_2_S removal, the system undergoes complete recovery by re-establishing the initial surface charge distribution. The Schottky barrier height is restored, reinstating the electron transfer limitation and returning the sensor to its baseline state. This reversible barrier modulation mechanism underpins the sensor’s reproducible operation and stable cycling performance.

Systematic electrical measurements were performed to investigate the interfacial barrier modulation, including I-V characterization and frequency-dependent impedance. The impedance spectroscopy results (Fig. 1d) reveal two distinct phenomena upon H_2_S exposure: (1) a significant reduction in frequency-dependent resistance, and (2) a characteristic shift of the reactance extremum toward higher frequencies. These observations indicate a substantial decrease in the relaxation time constant associated with H_2_S adsorption, which is fundamentally governed by the capacitive properties of the Schottky interface. Within the framework of the double-well potential energy model^49^, the observed reduction in relaxation time directly correlates with a decrease in Schottky barrier height.

Following the investigation of sensor fabrication and electron transport modulation—through I-V characteristics, resistance shifts, and impedance spectroscopy—the technological superiority of the device is further underscored by benchmarking its performance against state-of-the-art H_2_S gas sensors. To substantiate the advancement, a meta-analysis was conducted across four key performance metrics: operating temperature, responsivity, response time, and limit of detection (LOD), based on representative literature. As shown in Fig. 1e, pairwise comparisons reveal dual breakthroughs: (1) ultra-trace H_2_S detection down to 0.328 ppb at room temperature, and (2) a responsivity that ranks among the highest reported values to date. To further strengthen the quantitative basis of this benchmarking, we additionally extracted a normalized sensitivity metric (S/LOD, response-to-LOD ratio) directly from the literature data (Supplementary Table. 1), which jointly accounts for response magnitude and detection limit and places the present device among the state-of-the-art H_2_S sensors.

**Fig. 2:**
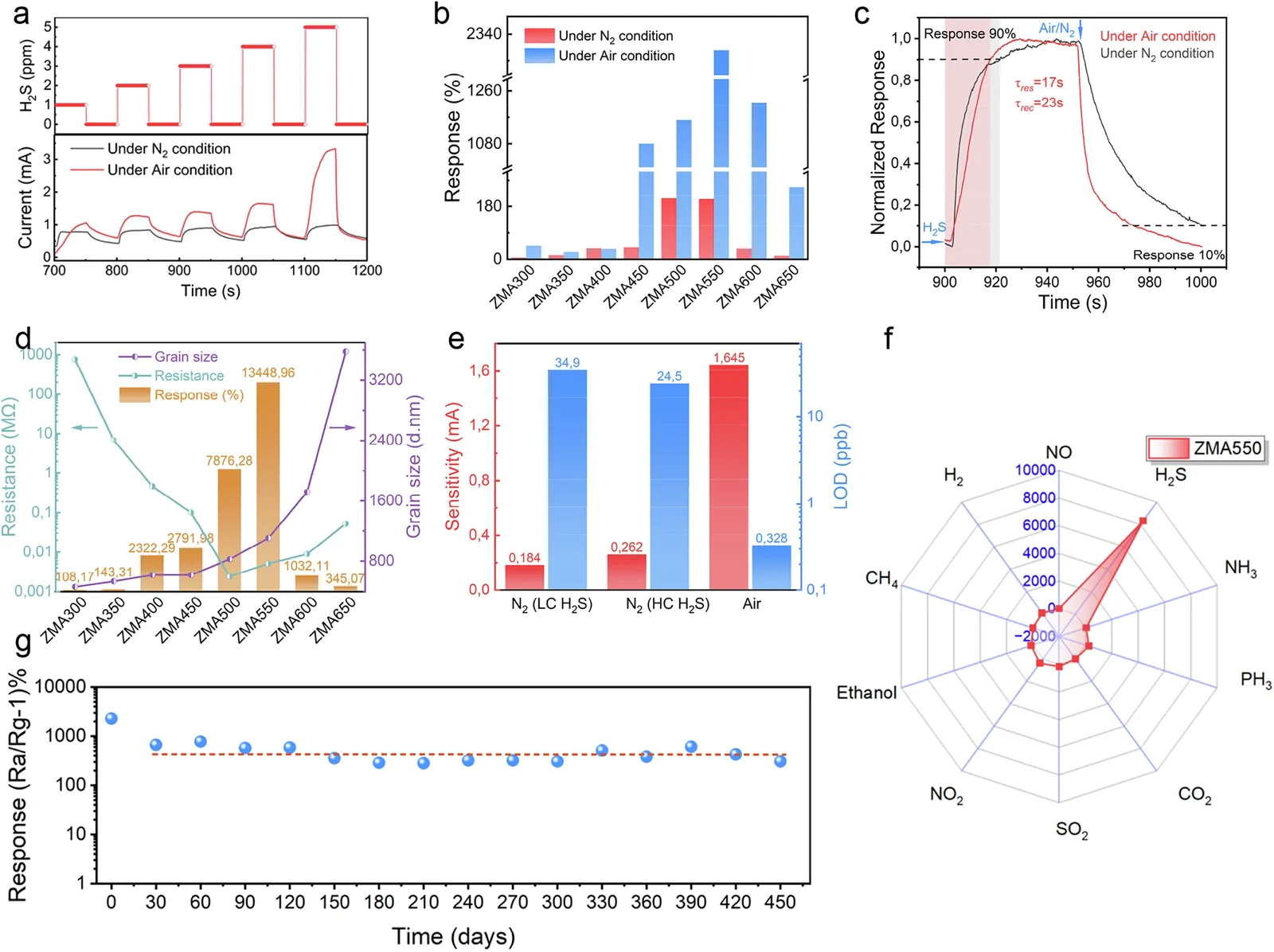
Gas sensing performance of the MOF-derived ZnO-based sensor. **a** Dynamic response and recovery curves of the ZMA550 sensor exposed to varying H_2_S concentrations under N_2_ and air conditions. **b** Comparison of response values to 5 ppm H_2_S across different samples in both N_2_ and air atmospheres. **c** Response and recovery times of the ZMA550 sensor towards 5 ppm H_2_S under N_2_ and air. The red and grey bars indicate the response-time intervals under air and N_2_, respectively, highlighting the faster response of the sensor under air conditions. **d** Correlation among response magnitude, logarithmic resistance, and grain size for all samples under N_2_. **e** Sensitivity and LOD of ZMA550 towards H_2_S in N_2_ and air. **f** Selectivity profile of ZMA550 in the presence of H_2_S (10 ppm), NH_3_ (10 ppm), PH_3_ (5 ppm), CO_2_ (500 ppm), SO_2_ (10 ppm), NO_2_ (10 ppm), C_2_H_6_O (26 ppm), CH_4_ (500 ppm), H_2_ (10 ppm), and NO (10 ppm). **g** Long-term stability of ZMA550 over 450 days, and the red dashed line indicates the approximate stable response level during long-term testing.

The current response and recovery curves for the ZMA550 sample (labeled according to synthesis temperature; see Supporting Information for details) at ambient temperature were obtained under different H_2_S concentrations in both N_2_ and air background environments (Fig. 2a). The response and recovery curves for other samples are presented in Supplementary Fig. 1. It is observed that excellent response characteristics are exhibited in both environments, with superior performance in air. This enhancement is primarily attributed to the abundant O_2_ present in the air background, which continuously supplies more reactive oxygen species to the material’s surface. In contrast, the sensing performance in the N_2_ environment is primarily governed by the intrinsic properties of the material. The sensing performance of samples treated at different temperatures toward 5 ppm H_2_S at ambient temperature was evaluated (Fig. 2b). The ZMA550 sample exhibited the highest response characteristics in both N_2_ and air background environments, indicating that GB concentration is a critical influencing factor. Notably, ZMA450 shows a markedly lower response and less favorable response-recovery dynamics, despite its higher apparent oxygen-vacancy fraction inferred from XPS (see discussion below). This non-monotonic trend indicates that the sensing output is not governed by a single defect indicator but rather by the coupled effects of defect chemistry and charge-transport across GBs. The observed trend is further corroborated by the consistent response patterns across different samples when exposed to higher H_2_S concentrations under an N_2_ atmosphere (Supplementary Fig. 2), suggesting that the intrinsic transport properties dominate the inter-sample differences under oxygen-free conditions. In the case of ZMA450, its finer grain structure and higher grain-boundary density, as revealed by EBSD analysis, create transport bottlenecks, further hindering its sensing performance. This highlights that the balance between defect density and microstructure is crucial for optimizing the material’s response. A baseline correction was applied to the response-recovery curves, with detailed methodological implementation and results presented in Supplementary Fig. 3.

Figure 2c presents the normalized recovery characteristics of the ZMA550 sample’s sensing response to 3 ppm H_2_S under both N_2_ and air background atmospheres. The response curve exhibits a pronounced increase upon H_2_S exposure, indicating a significant rise in sensor current. Conversely, the response curve decreases as the current diminishes. The sensor demonstrates a rapid response time of 17 s (defined as the time required for the response value to attain 90% of its peak value), with over 90% recovery achieved within 23 s. This behavior can be attributed to the chemical interaction between H_2_S and the adsorbed oxygen species on the ZnO surface when exposed to air^32–34^. Significantly, this study reveals that ZnO exhibits a robust H_2_S sensing response even under a pure N_2_ atmosphere, suggesting a direct interaction between the ZnO lattice and H_2_S molecules. In the absence of oxygen, ZnO retains its intrinsic properties, and upon H_2_S exposure, the electron density on the material surface increases, leading to a rise in current and the emergence of a response signal. Conversely, the current recovers as the H_2_S is removed.

Optimal sensing performance is achieved when the GB concentration is at a moderate level. This conclusion is further supported by the relationship between sensing response and grain size at varying H_2_S concentrations under an N_2_ environment (Fig. 2d). Notably, the sensing response of all samples to higher H_2_S concentrations was significantly greater than that observed at lower concentrations (Supplementary Fig. 2). Furtherly, in the extended range of 200-1000 ppm, the response continues to increase with concentration and no plateau is observed up to 1000 ppm, indicating that complete saturation is not reached within the tested window. Detailed results and analysis of the response behavior at these concentrations are provided in the Supplementary Information, including the concentration-response relationship and the observed changes in slope at higher concentrations (Supplementary Fig. 4).

Additionally, the correlation between resistance and response of different sensing materials was investigated. It was observed that resistance initially decreased and then increased with increasing annealing temperature, with the lowest resistance corresponding to samples with moderate grain size. This optimal balance is attributed to the fact that a moderate GB concentration provides sufficient active sites while avoiding excessive restriction of electron transport. This finding is further corroborated by the lowest resistivity observed in the ZMA550 sample (Supplementary Fig. 5).

The sensing performance of the ZMA550 sensor was systematically evaluated through multiple characterization protocols. Comparative analysis of sensitivity and LOD under N_2_ and air atmospheres revealed optimal performance in air, with a calculated LOD of 0.328 ppb for H_2_S detection (Fig. 2e, details shown in Supplementary Fig. 6). Complementary direct exposure measurements at 10–50 ppb H_2_S further support reliable sensing in the ppb-level regime (details shown in Supplementary Fig. 7). A detailed analysis of the differences, including standard deviation comparisons, among sensors from three distinct batches is presented in Supplementary Fig. 8. Selectivity assessment was conducted against a comprehensive panel of ten potentially interfering gases (NO, NH_3_, PH_3_, CO_2_, SO_2_, NO_2_, C_2_H_6_O, CH_4_, H_2_), demonstrating remarkable specificity toward H_2_S (Fig. 2f). The sensor maintains a stable and reversible response to H_2_S across a wide humidity range (15-90% RH) (Supplementary Fig. 9–11) and representative temperatures from −10 to 40 °C (Supplementary Fig. 12), demonstrating robust performance under realistic conditions. Furthermore, the long-term stability was evaluated by periodic measurements over 450 days (monthly tests under identical sensing conditions) rather than continuous operation (Fig. 2g). Remarkably, the monthly stability tests show negligible signal degradation over the entire testing period, underscoring the sensor’s reliability and suitability for long-term deployment in real-world environments. Post-test optical inspection and structural/chemical characterizations (HRTEM, PXRD, XPS, and EDX) confirm the integrity of the electrodes and exclude detectable secondary-phase formation (e.g., ZnS) after the stability campaign (Supplementary Fig. 13). In addition, post-cycling TEM, SEM, and XPS analyses further reveal that the grain morphology and grain-boundary network remain intact after 450-day operation, while only minor surface chemical evolution is observed without inducing measurable performance degradation (Supplementary Fig. 14). Together, these results confirm that the long-term sensing stability arises from both structural robustness and dynamically stable interfacial chemistry.

To assess cross-interference under mixed-gas conditions relevant to practical environments, we performed dynamic co-exposure tests in which H_2_S was introduced together with representative oxidizing gases (NO_2_ and SO_2_) and a reducing gas (NH_3_), both individually and in mixtures. The sensor maintains a stable and reproducible H_2_S response even when oxidizing and reducing interferents coexist simultaneously, demonstrating strong anti-interference capability in complex gas matrices (Supplementary Fig. 15–17). The robustness is particularly relevant to the boiled-egg spoilage headspace tests, where multiple VOCs (e.g., amines, organic acids, and small sulfur-containing species) may be present. The mixed-gas evaluation therefore supports that the response observed in the spoilage demonstration is primarily governed by H_2_S-associated surface/interfacial modulation rather than nonspecific responses to coexisting volatiles.

### Grain boundaries and Schottky barrier

**Fig. 3:**
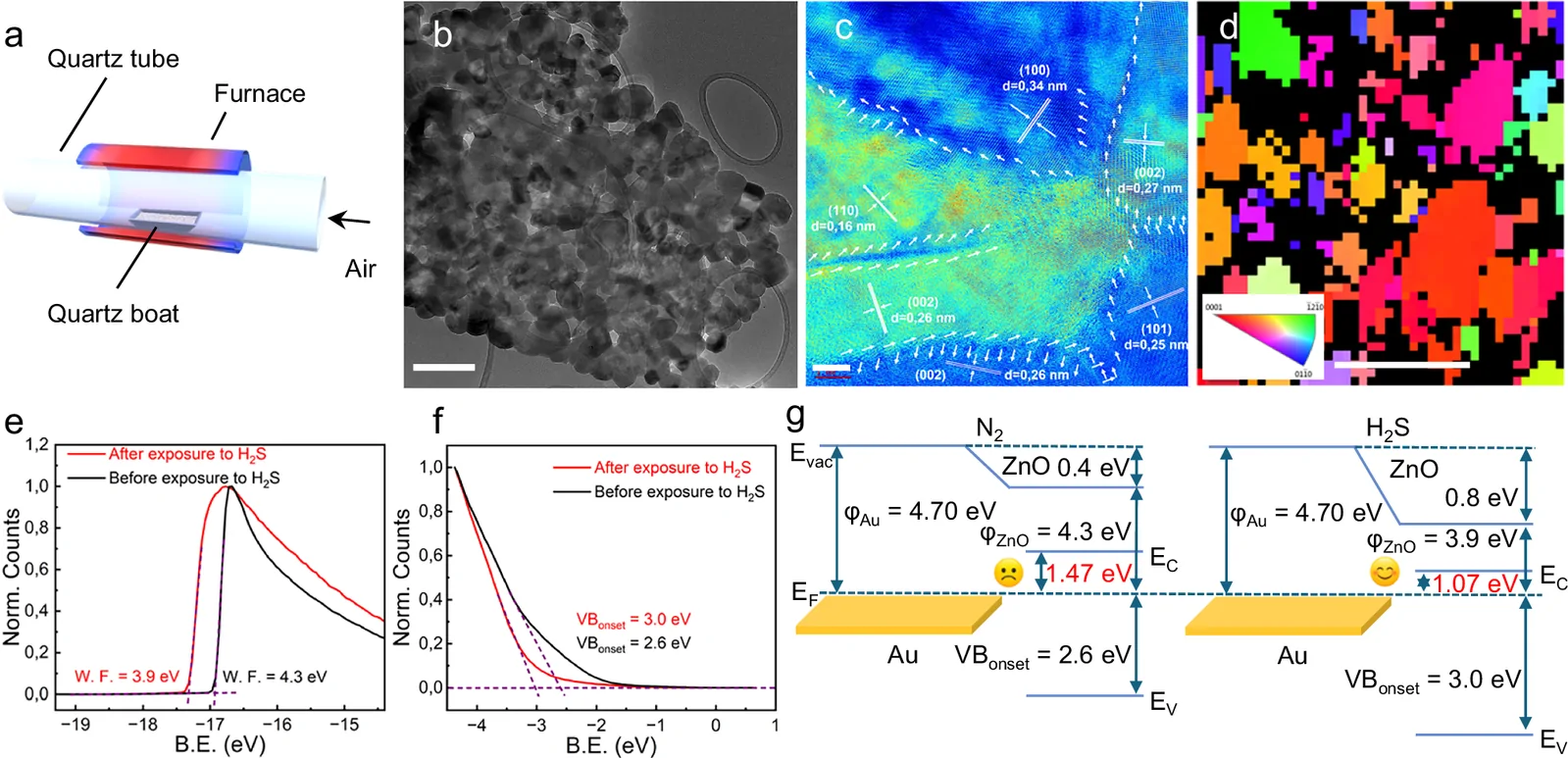
Thermal conversion process and structural analysis of ZnO materials, and Schottky barrier modulation upon H_2_S exposure. **a** Schematic of the tubular furnace setup used for the thermal transformation of Zn MOF under controlled atmosphere. **b** Representative TEM image of the GB-rich ZnO structure derived from the Zn MOF, and similar results were obtained from three independently prepared samples. **c** High-resolution TEM image showing clear lattice fringes, highlighting pronounced GB features. **d** EBSD map illustrating the distribution of grain orientations and boundaries. Scale bars, 200 nm (**b**), 5 nm (c), and 250 nm (**d**). **e** UPS spectra showing the secondary electron cutoff for work function determination. **f** UPS spectra of the valence band region indicating energy level shifts upon H_2_S adsorption. **g** Schematic illustration of the Schottky barrier modulation before and after exposure to H_2_S.

The temperature-sensitive characteristics of the Zn MOF were harnessed to induce structural evolution, enabling controlled modulation of GB density (Fig. 3). As illustrated in Fig. 3a, a schematic of the tubular furnace system used for the thermal conversion of Zn MOF under a controlled atmosphere is presented. The microstructural features of the ZMA550 sample were investigated by transmission electron microscopy (TEM). The results indicate uniformly distributed nanograins, approximately tens of nanometers in size (Fig. 3b), further confirming the homogeneous distribution of GBs within the material. As shown in Fig. 3c, the HRTEM image reveals lattice fringes between neighboring grains with distinct orientation differences, indicating a uniform distribution of GBs. To obtain more conclusive evidence, electron backscatter diffraction (EBSD) analysis was conducted to examine the crystal orientation (Fig. 3d). The EBSD results confirm that adjacent grains exhibit different growth orientations and that GBs are uniformly distributed. These findings demonstrate that precise control over GB density can be achieved by adjusting the pyrolysis temperature of Zn MOF, further underscoring its high sensitivity to temperature variations. Furthermore, an in-depth HRTEM analysis of individual small grains was conducted (Supplementary Fig. 18a). Fourier Transform (FT) performed on selected regions revealed distinct lattice diffraction fringes with a grain face spacing of 0.247 Å (Supplementary Fig. 18b). This spacing precisely corresponds to the (101) crystal plane of ZnO, indicating preferential grain growth along this plane. Selected area electron diffraction (SAED) analysis (Supplementary Fig. 18c) further confirmed this observation, displaying a bright array of diffraction spots characteristic of a single-crystal structure, verifying that ZnO grains selectively grow along the [101] direction.

Furthermore, this interfacial barrier modulation was quantitatively verified by ultraviolet photoelectron spectroscopy (UPS) measurements of ZnO before and after H_2_S exposure (Fig. 3e, f). The UPS spectra show a clear electronic structure shift: the work function (WF) decreases from 4.3 eV to 3.9 eV (Fig. 3e), and the valence band onset (VB_onset_) increases from 2.6 eV to 3.0 eV (Fig. 3f). These variations indicate an upward shift of the Fermi level in ZnO, consistent with electron donation from adsorbed H_2_S. This leads to a narrower surface depletion region and a flatter band profile on the ZnO side. The sensing interface, defined by a Schottky junction between Au and ZnO, exhibits a reconfigured band alignment upon exposure to H_2_S (Fig. 3g), accompanied by a reduction in the interfacial Schottky barrier height. A quantitative analysis combining UPS data and the optical bandgap of ZnO (E_g_, Supplementary Fig. 19) reveals a 0.4 eV decrease in the barrier height (details shown in Supplementary Fig. 20 and Table 2), highlighting the strong influence of surface electronic structure modulation on the Au-ZnO contact. This electronic structure reconfiguration is pivotal in achieving high sensitivity and low detection limits at room temperature.

Modifying the barrier height critically influences charge carrier transport across the metal-semiconductor interface. The charge transport characteristics across the metal-semiconductor interface are fundamentally governed by the Schottky barrier height, which determines the probability of carrier injection. Based on thermionic emission theory^47^, the current density (J) exhibits an exponential dependence on the barrier height (Φ_B_):

1 $$J=A{T}^{2}\propto \exp (-{\varPhi }_{B}{{{\rm{n}}}}/{{{\rm{k}}}}T)$$

where k is the Boltzmann constant (8.617 × 10^−^^5^ eV/K), and T denotes the absolute temperature (T = 300 K). Quantitative analysis based on thermionic emission theory indicates that the 0.4 eV reduction in barrier height corresponds to an increase in current density by approximately 5.2 × 10^6^ folds, highlighting the substantial effect of interfacial modulation on charge transport. This substantial barrier lowering simultaneously reduces the characteristic carrier transit time, accelerating the sensor’s response to H_2_S adsorption/desorption dynamics. The exceptional gas selectivity originates from the unique chemisorption-induced Schottky barrier modulation specific to H_2_S-ZnO/Au interactions. A comparative analysis of barrier modulation characteristics across different sensor architectures reveals the superior performance of the ZnO/Au Schottky configuration. The 0.4 eV barrier height modification significantly surpasses that observed in conventional metal-oxide-semiconductor (MOS) sensors and tunneling-based devices^50,51^, accompanied by a current enhancement factor at least five orders of magnitude greater than alternative sensor designs.

To further elucidate the structural origin of the interfacial barrier characteristics, we establish a direct correlation among grain size, GB density, and Schottky barrier height across samples prepared at different pyrolysis temperatures (Supplementary Fig. 21). EBSD analysis shows that increasing pyrolysis temperature induces progressive grain growth accompanied by a monotonic reduction in GB density (Supplementary Fig. 22 and Table 3). Correspondingly, UPS measurements reveal a systematic decrease in the Schottky barrier height derived from the work function and valence band onset (Supplementary Fig. 20 and Table 2). These results demonstrate that GB density, governed by the grain size evolution, critically determines the interfacial barrier landscape, thereby providing a quantitative structural basis for the synergistic grain-boundary-assisted barrier regulation mechanism. As detailed in Supplementary Fig. 23, the direct transport measurements, together with EBSD-derived grain-boundary statistics and correlation analysis with sensing response, establish a quantitative link between grain boundary density, charge transport, and sensing performance.

### Identification of sensing layer

The sensing layer materials were characterized by scanning electron microscopy (SEM), revealing distinct morphological features and structural evolution during pyrolysis (Supplementary Fig. 24). The material was synthesized via pyrolysis of a Zn MOF precursor (Supplementary Fig. 25), which initially exhibits a spindle-shaped morphology with smooth surfaces. Increasing the pyrolysis temperature led to the formation of tightly packed, uniformly distributed nanoparticles with well-defined GBs, indicating a pronounced temperature-dependent morphological evolution of the Zn MOF. At 300 °C, surface roughening of the MOF was observed (Supplementary Fig. 24a), suggesting localized structural collapse at the early stage of pyrolysis. Upon heating to 400 °C, smaller nanoparticles appeared on the surface with increased GB density (Supplementary Fig. 24b). Further temperature elevation caused nanoparticle growth and gradual reduction in GB density, while the overall spindle-shaped morphology remained intact (Supplementary Fig. 24c–h). Notably, above 550 °C, nanoparticles exceeded 100 nm and GB density decreased markedly (Supplementary Fig. 24f). Grain size distribution analysis (Supplementary Fig. 26) confirmed the progressive increase in grain dimensions, consistent with thermally activated grain growth. Elemental mapping of the ZMA550 sample by EDS (Supplementary Fig. 27) showed homogeneous spatial distribution of Zn and O, supporting uniform structural evolution during pyrolysis.

Phase composition and crystal structure were analyzed using X-ray diffraction (XRD) spectroscopy (Supplementary Fig. 28), revealing that the MOF underwent three phase transitions between 300 °C and 400 °C. The final ZnO phase was confirmed by diffraction peaks at 31.89°, 34.56°, 36.37°, 47.67°, 56.71°, 62.97°, 68.08°, and 69.19°, corresponding to the (100), (002), (101), (102), (110), (103), (112), and (201) planes. Samples treated at 450 °C displayed lower peak intensity and broader full width at half maximum (FWHM), indicating ongoing phase transformation, whereas at 550 °C, peak intensity maximized and FWHM minimized, suggesting completion of the ZnO formation. The ZnO phase remained stable even at 650 °C, without forming additional heterogeneous phases.

Color changes of the samples correlated with pyrolysis temperature (inset at bottom left of Supplementary Fig. 24a1–h1). The MOF appeared milky white initially, turned reddish brown at lower annealing temperatures, and gradually lightened to white at higher temperatures. This evolution reflects quantum size effects of nanoparticles and light scattering variations, as well as contributions from intrinsic defects such as oxygen vacancies (V_O_), zinc vacancies (V_Zn_), and interstitial defects. Raman spectra (Supplementary Fig. 29) further corroborated this, showing the emergence of *E*_2_ (low) and *E*_2_ (high) modes at 99 and 435 cm^−1^ at 450 °C, which decreased in intensity with increasing temperature, indicating reduced zinc vacancies. Additional modes, *A*_1_ (TO) at 382 cm^−1^ and *E*_1_ (LO) at 584 cm^−1^, confirmed the presence of oxygen-related lattice defects^52,53^.

XPS full-spectrum analysis (Supplementary Fig. 30a) revealed surfaces composed mainly of C, N, Zn, and O, with composition varying with annealing temperature. Deconvolution of the O 1*s* spectrum (Supplementary Fig. 30b) identified lattice oxygen (O_1_, ~530.0 eV), oxygen vacancies (O_2_, ~531.1 eV), and loosely bound surface oxygen species (O_3_, ~532.2 eV). With increasing temperature, O_2_ and O_3_ content decreased, while O_1_ dominated, indicating improved crystallinity and reduced defects (Supplementary Fig. 30c). Zn 2*p* peaks at ~1022 eV and ~1044 eV confirmed ZnO formation (Supplementary Fig. 30d), and the gradual disappearance of C 1 *s* and N 1 *s* peaks (Supplementary Fig. 30e, f) suggested effective removal of organic components during pyrolysis. Auger spectral analysis of O KLL and Zn LMM further confirmed the evolution of chemical states and surface defects (Supplementary Fig. 30g, h), with quantitative elemental compositions listed in Supplementary Table 4.

UV-vis absorption spectroscopy was employed to examine optical properties and estimate band gaps (Supplementary Fig. 19). The ZMA350 sample exhibited the largest band gap, ZMA450 the smallest, and higher-temperature samples showed gradual band gap widening. This trend reflects variations in defect concentration, such as oxygen vacancies and GBs, which affect the material’s electronic structure^54–56^. The UV-vis results are consistent with XRD and XPS analyses, confirming that defect concentration and GB density significantly influence both optical properties and crystal structure.

### Mechanism of hydrogen sulfide detection

The microstructural evolution of materials under various thermal treatments significantly influences their sensing characteristics. The initial formation of the ZnO phase yields nanostructures characterized by small grain dimensions and a high GB density. While this morphology provides abundant active sites on the surface, it simultaneously induces substantial electron scattering, thereby limiting the mobility of charge carriers. With increasing thermal treatment temperature, a systematic reduction in GB density is observed, achieving an optimal balance between sufficient active site availability and efficient electron transport. This optimized microstructure facilitates enhanced sensing performance through two synergistic mechanisms: (1) surface charge redistribution upon H_2_S chemisorption, and (2) effective Schottky barrier modulation at the metal-semiconductor interface. These combined effects enable rapid modulation of electrical resistance and consequent current response. Importantly, the sensing performance does not scale monotonically with specific surface area.

Although ZMA450 exhibits the highest BET surface area, its sensing response is inferior to that of ZMA550, indicating that excessive surface area associated with ultra-fine grains does not necessarily translate into effective sensing. Excessive thermal treatment leads to excessive grain growth, resulting in two detrimental effects: (1) significant reduction in surface active sites, and (2) decreased specific surface area, as confirmed by BET measurements (Supplementary Fig. 31). This over-annealing effect ultimately degrades the sensing performance despite improved charge transport characteristics. Together, these results suggest the existence of an optimal microstructure window, where surface area, pore accessibility, and carrier transport are balanced to maximize sensing performance.

The gas sensing mechanism governed by the synergistic effects of Schottky barrier modulation and GB engineering is illustrated. The intrinsic polar nature of ZnO, comprising alternating Zn^2+^ (Lewis acid) and O^2^⁻ (Lewis base) surface sites^57^, establishes a chemically active surface environment. This unique surface chemistry facilitates H_2_S interaction through dual mechanisms: electron acceptor behavior at Zn^2+^ sites, and electron donor characteristics at O^2^⁻ sites. The complementary Lewis acid-base interactions provide abundant active sites for H_2_S adsorption.

The H_2_S molecule, possessing lone electron pairs with electron density localized predominantly on the sulfur atom, functions as a Lewis base. This electronic configuration facilitates strong interaction with Lewis acid sites (Zn^2+^) on the ZnO surface. Furthermore, the GB-rich ZnO structure exhibits enhanced surface reactivity due to the preferential formation of oxygen vacancies (V₀∙∙) at these high-energy sites, with vacancy concentrations significantly exceeding those in bulk regions.

The surface reaction pathway proceeds through two sequential steps: Initially, H_2_S interacts with surface oxygen vacancies through the following redox reaction:

2 $${{{{\rm{V}}}}}_{0}\cdot \cdot+{{{{\rm{H}}}}}_{2}{{{\rm{S}}}}\to {{{\rm{S}}}}{{{{\rm{H}}}}}^{-}+{{{{\rm{H}}}}}^{+}+{{{{\rm{e}}}}}^{-}$$

Subsequently, the generated SH⁻ species (acting as a Lewis base) coordinates with surface Zn^2+^ cations to form stable Zn-SH complexes:

3 $${{{\rm{S}}}}{{{{\rm{H}}}}}^{-}+{{{{\rm{Zn}}}}}^{2+}\to {{{\rm{Zn}}}}-{{{\rm{SH}}}}$$

Concurrently, the liberated protons (H⁺) interact with lattice oxygen anions (O^2^⁻) to generate surface hydroxyl groups:

4 $${{{{\rm{H}}}}}^{+}+{{{{\rm{O}}}}}^{2-}\to {{{\rm{O}}}}{{{{\rm{H}}}}}^{-}$$

In situ FTIR measurements provide experimental support for this surface reaction pathway, revealing reversible perturbations of surface hydroxyl groups, acid-base sites, and Zn-O-related lattice vibrations during H_2_S adsorption, consistent with the formation of sulfur-containing surface intermediates rather than irreversible sulfidation (Supplementary Fig. 32). The DFT calculations show that the dissociative adsorption of H_2_S results in significant electron transfer to the ZnO surface, leading to a reduction of the interfacial barrier (Details discussion can be found in Supplementary Fig. 33). These findings are consistent with the in-situ FT-IR measurements, where surface hydroxyl groups and Zn-O vibrations show dynamic shifts during H_2_S exposure and removal. Together, the DFT results and in situ FT-IR data establish a coherent understanding of how H_2_S adsorption modulates the electronic structure and surface chemistry of ZnO, confirming the reversible nature of the sensing process and its dependence on surface coordination dynamics rather than irreversible chemical transformation.

To further investigate the influence of defects on sensing performance, we performed DFT calculations to evaluate the adsorption of H_2_S at both oxygen and zinc vacancy sites. The results confirm that oxygen vacancies play a more significant role in H_2_S adsorption, and full data and analysis are given in Supplementary Fig. 34 and Tables 5, 6. Although the model represents an idealized local configuration rather than the full structural complexity of polycrystalline ZnO, it effectively captures the key physicochemical interactions, such as charge transfer and electronic structure modulation, that govern the sensing behavior. The trends obtained from the calculations, including the preferential adsorption at oxygen vacancies and the correlation between grain boundary density and sensing performance, are in strong agreement with the experimental observations, providing confidence that the simplified model captures the dominant physics underlying the sensing process. Additionally, the electron paramagnetic resonance spectroscopy (EPR) measurements were performed to directly assess the oxygen-vacancy concentration trends in the samples (*g* ≈ 2.003–2.004) and reveal a monotonic decrease in signal intensity with increasing annealing temperature; full data and analysis are given in Supplementary Fig. 35.

The aforementioned surface reactions induce substantial modifications in the electronic structure of ZnO, altering the surface charge distribution and bulk carrier concentration. XPS, UV-vis, and EPR analyses collectively indicate that oxygen-vacancy-related defects represent a dominant class of surface-active sites in the ZnO matrix, giving rise to a Zn-rich and non-stoichiometric surface. Such defect-rich surfaces exhibit enhanced Lewis acidity and facilitate H_2_S adsorption and activation at the chemical level. However, as demonstrated by the comparative analysis of ZMA450 and ZMA550, the overall sensing performance is ultimately governed by the coupled effects of defect chemistry and charge-transport efficiency.

The electron-donating nature of H_2_S molecules, characterized by their high electron density, contributes additional electrons to the ZnO conduction band. This donor-like behavior increases the majority carrier concentration in n-type ZnO, leading to a pronounced enhancement in electrical conductivity. The electronic structure modifications are quantitatively characterized by several key parameters: (1) reduction in work function from 4.3 eV to 3.9 eV, (2) increase in electron affinity from 2.13 eV to 2.83 eV, and (3) substantial lowering of the Schottky barrier height by approximately 0.4 eV. These collective changes result in a dramatic intensification of the thermionic emission current, directly correlating with the observed enhancement of the sensing response.

GBs exhibit dual functionality in the sensing mechanism, serving simultaneously as carrier scattering centers and preferential adsorption sites. While elevated GB densities enhance electron scattering, thereby reducing carrier mobility and increasing baseline resistance (Supplementary Fig. 1), they simultaneously provide abundant active sites for gas adsorption and facilitate enhanced electron injection upon H_2_S exposure. Conversely, reduced GB concentrations, while improving carrier mobility through diminished scattering, compromise sensing performance by limiting available adsorption sites and weakening the electron injection effect.

Optimal sensing performance is achieved through precise GB engineering, as exemplified by the ZMA550 sample, which reflects a balanced trade-off between carrier transport and surface reactivity rather than a non-monotonic optimization of the barrier height itself. This balance arises from three coupled factors: (1) a sufficient GB density to provide abundant adsorption and charge-trapping sites, (2) moderated GB density to avoid excessive carrier mobility degradation, and (3) the formation of a continuous conductive network that prevents severe carrier localization at intergranular potential barriers. Systematic microstructural analysis combining EBSD and UPS establishes a clear and monotonic structure-barrier relationship across samples pyrolyzed at 500-650 °C (Supplementary Fig. 21). Increasing pyrolysis temperature induces progressive grain growth and a corresponding reduction in GB density, which is accompanied by a systematic decrease in the Schottky barrier height derived from UPS. This trend is consistent with the established picture that GB-rich polycrystalline ZnO hosts stronger depletion and band bending due to a higher density of interfacial trap states. To complement these experimental observations, density functional theory (DFT) simulations were employed to elucidate the mechanistic role of GBs in adsorption-induced barrier modulation. The calculations reveal that GB-associated under-coordinated sites and defect-prone regions facilitate charge transfer and amplify local barrier modulation upon H_2_S adsorption. Notably, the “optimal” sensing behavior arises from a device-level trade-off rather than from a non-monotonic dependence of the barrier height on GB density. Specifically, increasing GB density can enlarge the available barrier-modulation window, whereas an excessively high barrier elevates the baseline resistance and compromises response/recovery kinetics. Therefore, an intermediate GB density provides the most favorable overall performance when sensitivity, transport efficiency, and dynamic characteristics are evaluated in combination. This synergistic interplay between GB-assisted adsorption and transport regulation enables exceptional room-temperature trace H_2_S detection with high sensitivity and operational stability.

### Quantitative hydrogen sulfide sensing in practical scenarios

**Fig. 4:**
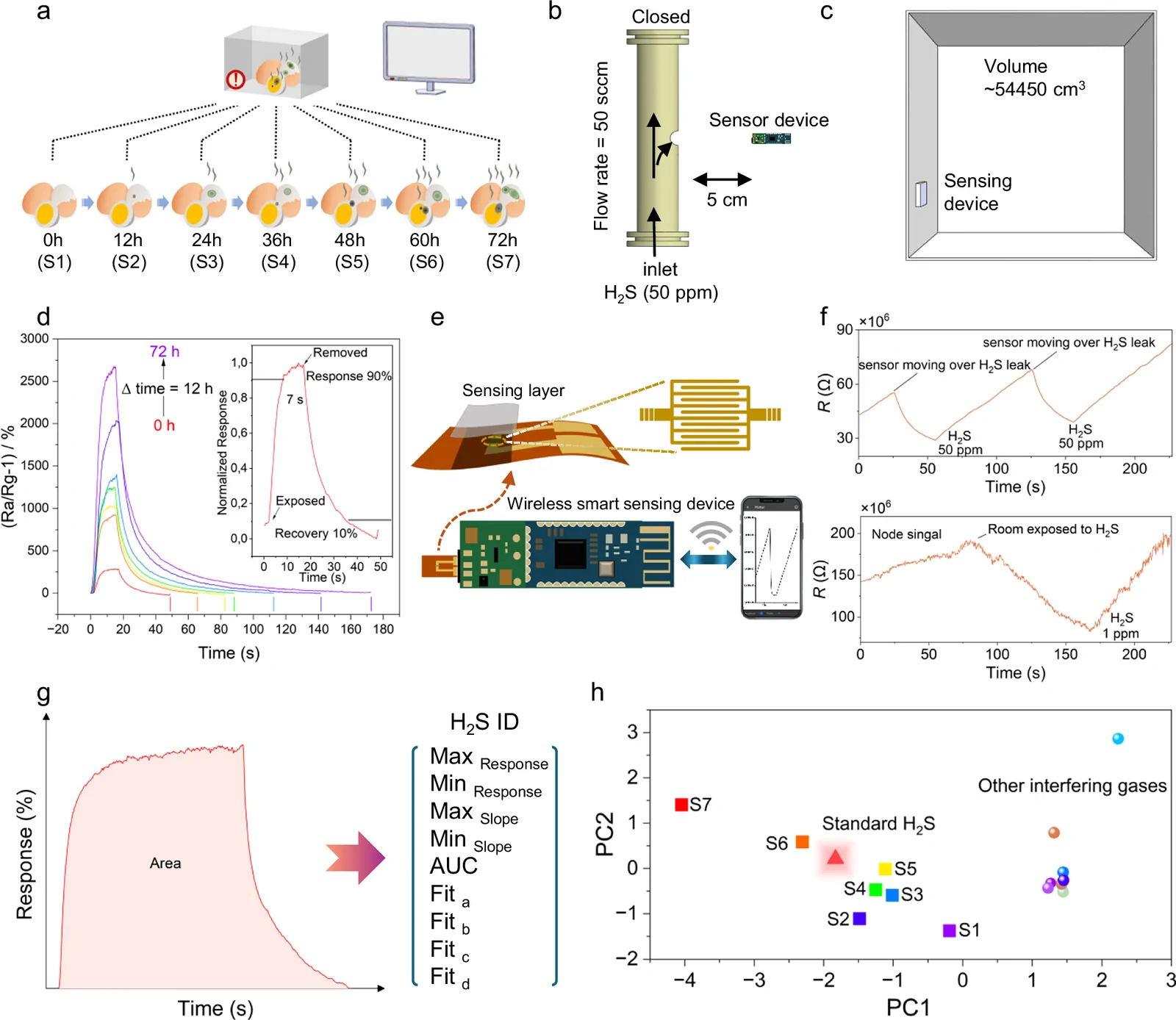
Evaluation of wireless H_2_S sensing performance under diverse practical scenarios. **a** Schematic illustration of the wireless measurement setup for time-resolved detection of H_2_S released during the anaerobic degradation of boiled eggs, simulating food spoilage monitoring. **b** Schematic of the sensing setup for real-time detection of H_2_S leakage from pipelines. **c** Experimental configuration for wireless H_2_S monitoring in a confined space. Approximately 54.45 mL of 1000 ppm H_2_S/N_2_ was released into a sealed 54,450 cm^3^ chamber, corresponding to a final simulated concentration of 1 ppm, mimicking indoor gas accumulation. **d** Time-resolved response and recovery curves of the sensor to egg samples stored for different fermentation durations (0–72 h). Inset: normalized response-recovery curve of the 0 h sample. **e** Schematic illustration of the wireless sensing device, consisting of a sensing layer deposited on gold interdigitated electrodes fabricated on a flexible Kapton substrate. Real-time resistance changes are wirelessly transmitted to a smartphone for monitoring. **f** Real-time response and recovery curves of the sensor during H_2_S leakage detection from a damaged pipeline (top) and within a confined space (bottom). **g** Schematic of the feature extraction strategy used to differentiate between standard H_2_S gas, spoilage-generated H_2_S, and other interfering analyte gases. **h** Principal component analysis (PCA) distribution showing the distinct clustering of responses from standard H_2_S and other interfering gases, confirming high selectivity of the sensor.

To demonstrate the real-world applicability of the developed wireless sensing device, we conducted a series of quantitative H_2_S detection experiments under three practical scenarios (Fig. 4): (1) food spoilage monitoring via anaerobic degradation of boiled eggs (Figs. 4a), (2) leakage detection in H_2_S transport pipelines (Figs. 4b), and (3) high-concentration H_2_S accumulation in confined industrial environments (Fig. 4c) such as sulfur-containing chemical plants—each representing critical domains of safety and environmental relevance. In the first scenario, a sealed anaerobic fermentation process was established by dividing a hard-boiled egg into seven equal parts and storing them individually at room temperature. This setup simulates microbial decomposition-induced H_2_S emission, a common indicator of food spoilage. At selected time intervals, the accumulated gas was exposed to the sensor by briefly opening the sealed container near the sensing layer. Remarkably, a time-dependent increase in sensor response was observed with progressing fermentation, and rapid recovery to baseline occurred within seconds after sample removal (Fig. 4d). The sensor achieved 90% of its peak response in just 7 seconds, demonstrating outstanding temporal resolution.

The response signals were wirelessly transmitted via Bluetooth to a mobile device in real time (Fig. 4e), enabling remote, on-demand food quality monitoring. This hands-free, non-invasive sensing protocol validates the potential of our device for rapid and portable hazard assessment in supply chains. The underlying mechanism involves fast surface interactions between H_2_S molecules released from the sample and the ZnO-based sensing layer, which modulate electron transport pathways and generate measurable resistance shifts. Together, these demonstrations illustrate the device’s capability for precise and timely H_2_S quantification across diverse, safety-critical environments, offering a practical foundation for future deployment in smart sensing networks and industrial Internet of Things (IIoT) frameworks.

In the second scenario, the wireless sensing device was employed to detect H_2_S leakage from a simulated industrial pipeline (Fig. 4b). When the sensor was positioned approximately 5 cm from a small defect in the pipeline, H_2_S gas (50 ppm) began to leak into the ambient environment. A rapid drop in resistance was recorded in real time as the sensor responded to the escaping gas. Upon removing the sensor from the leakage vicinity, the resistance gradually returned to its baseline value, evidencing full recovery (Fig. 4f, top). This test cycle was repeated twice with consistent results, confirming the sensor’s reliable performance in detecting transient, localized H_2_S leaks under dynamic field conditions. To better simulate practical leak-monitoring conditions, the sensor under several realistic scenarios was evaluated. Beyond the original 5 cm condition, the sensor was positioned at 0.50 m, 1.0 m, 3.0 m, and 5.0 m from the leak outlet in still air. To simulate industrial airflow disturbances, the sensor was fixed at 1.0 m from the leak outlet and exposed to weak airflow, weak airflow with dust, and strong airflow. These results emphasize the sensor’s effectiveness in realistic conditions, ensuring early leak detection and reducing safety and economic risks (details in Supplementary Fig. 37).

The third and most critical use case simulated H_2_S accumulation in a confined space—an often overlooked yet lethal hazard in sulfur-processing industries and enclosed infrastructure. A sealed chamber of 54,450 cm^3^ was filled with H_2_S to achieve a uniform concentration of 1 ppm. Upon exposure, the sensor promptly exhibited a measurable decrease in resistance, sustaining the response until the chamber was purged (Fig. 4f, bottom). Notably, the sensor demonstrated full recovery post-exposure, highlighting sensitivity and reversibility in low-oxygen, enclosed environments. To evaluate sensing behavior at high H_2_S concentrations (>100 ppm) in industrial scenarios, the sensor was measured at 200, 400, 600, 800, and 1000 ppm. The baseline returned without irreversible drift, indicating the sensor’s short-term tolerance to high-concentration shocks under the tested conditions (details in Supplementary Fig. 38). Furthermore, the sensor was measured under incremental low concentrations of H_2_S (1, 2, 3, 4, 5 ppm) with short time recovery, as well as under continuously high H_2_S concentrations (10, 50, 100, 500, 1000 ppm) to simulate spatial gradients and slow accumulation rates. The results demonstrate that the sensor reliably handles both spatially non-uniform concentration distributions and slow accumulation rates. (details in Supplementary Fig. 39).

Of particular significance is the device’s energy efficiency. The sensor consistently maintained high resistance values throughout static and active testing conditions, indicative of inherently low current flow. Benefiting from heater-free, room-temperature operation, the sensing element in this work operates in the μW power regime with an average power consumption of 0.7 μW in active mode and 0.3 μW in idle mode (details in Supplementary Table 7), whereas representative commercial MOS H_2_S sensors typically operate in the mW regime (25-1300 mW), as derived from publicly available datasheet specifications. This architecture-level comparison highlights an energy-budget reduction of approximately five orders of magnitude. In addition, the response dynamics are approximately 4-5× faster than those reported for commercial MOS sensors, based on datasheet values and publicly available literature. These comparisons are intended to highlight fundamental differences in sensing architecture, rather than a one-to-one performance comparison under identical testing conditions (details in Supplementary Table 8).

This ultra-low power footprint is a critical enabler for scalable deployment of large-area, autonomous sensing networks where battery life, energy harvesting, and cost-efficiency are key constraints. These real-environment demonstrations collectively underscore the wireless sensing device’s unique integration of sensitivity, reversibility, operational stability, and low-power design, establishing it as a strong candidate for next-generation electronic nose systems in consumer and industrial applications.

It is increasingly recognized that evaluating sensor performance under practical conditions presents far greater complexity than controlled laboratory testing. In practical scenarios, chemical environments typically comprise complex mixtures rather than isolated analytes. A well-known example is the generation of H_2_S during the anaerobic decomposition of boiled eggs, which produces the characteristic “rotten egg” odor. However, this process does not yield H_2_S alone. Eggs naturally contain diverse mineral components and abundant carbon-, nitrogen-, hydrogen-, oxygen-, and sulfur-containing compounds, suggesting the possible co-release of various volatile species during fermentation. This poses a critical challenge: the strong response observed in our previous wireless detection experiments must be rigorously attributed to H_2_S rather than confounding gases.

To address this, we first established a comprehensive response database by exposing the sensor to ten individual standard gases, including H_2_S, and extracting ten characteristic descriptors from each dynamic response profile (Fig. 4g). These descriptors encode features, which together form a multidimensional “fingerprint” for each gas. Subsequently, we applied the same feature extraction strategy to sensor responses obtained from seven boiled egg samples fermented for varying durations (Supplementary Fig. 40). A comparative analysis was then performed to match these empirical response profiles to the reference signatures. The results, illustrated in Fig. 4h, demonstrate that the responses from the decomposing egg samples align most closely and consistently with the standard H_2_S profile, rather than with any of the other tested gases. These findings validate the high selectivity of the sensor in complex and uncontrolled chemical backgrounds, and highlight the robustness of our descriptor-based identification framework. This approach provides a valuable pathway toward deploying machine-readable, intelligent sensing systems that reliably navigate non-ideal real-world conditions.

## Discussion

This work presents a wireless, ultra-low-power H_2_S sensor capable of reliable, real-time monitoring across a wide range of practical scenarios. The device delivers an extraordinary sensing response exceeding 10,000%, a calculated detection limit of 0.328 ppb, and a rapid 7 s response at room temperature, all while consuming less than 0.7 μW—outperforming state-of-the-art gas sensors, representing 4-5× faster response and up to 10^5^-fold lower active sensing power demand compared to a commercial H_2_S sensor. These metrics significantly outperform those of conventional sensing technologies. Its architecture, based on Au-interfaced MOF derivatives, leverages synergistic modulation of Schottky barriers and GBs to maximize carrier transport and adsorption efficiency, achieving a Schottky barrier shift of 0.4 eV and enhancing the current density of the sensing material by more than 10^6^-fold. The demonstrated device effectively addresses diverse H_2_S monitoring scenarios, from food spoilage assessment to pipeline leakage detection and industrial safety control. These findings establish a robust and versatile sensing strategy that combines high performance with long-term stability, underscoring its potential for next-generation smart environmental monitoring systems.

## Methods

### Materials

Zinc nitrate hexahydrate (Zn(NO_3_)_2_·6H_2_O), 2-methylimidazole, and absolute ethanol were purchased from Thermo Fisher Scientific (Germany). All chemicals were of analytical grade and were used without further purification. Deionized water was used throughout the synthesis.

### Synthesis of the Zn MOF precursor

The Zn-based metal–organic framework precursor was synthesized using a room-temperature solution-phase coordination method. Zn(NO_3_)_2_·6H_2_O (12 mmol) and 2-methylimidazole (96 mmol) were separately dissolved in 120 mL of deionized water. The 2-methylimidazole solution was added dropwise to the zinc nitrate solution under continuous magnetic stirring at 600 rpm. The resulting mixture was stirred at room temperature for 24 hours to allow coordination and precipitation. The resulting white precipitate was collected by centrifugation at 11,404 × *g* for 3 min, washed three times with absolute ethanol, and dried overnight under vacuum at 60 °C.

### Preparation of ZnO derivatives with tunable grain-boundary densities

The as-prepared Zn MOF precursor was uniformly distributed in a quartz boat and placed in the center of a horizontal tube furnace. Before heating, compressed air was passed through the furnace at 240 sccm for 30 min to remove residual gases. The precursor was heated to 300, 350, 400, 450, 500, 550, 600 or 650 °C at a heating rate of 2 °C min^−^^1^ and maintained at the selected temperature for 2 hours under continuous airflow. The furnace was subsequently allowed to cool naturally to room temperature. The resulting ZnO-based products were denoted ZMA300, ZMA350, ZMA400, ZMA450, ZMA500, ZMA550, ZMA600 and ZMA650 according to their respective calcination temperatures.

### Fabrication of Au interdigitated electrodes

Au interdigitated electrodes were fabricated on Kapton substrates using standard ultraviolet photolithography. A photoresist layer was coated onto the substrate and soft-baked, followed by ultraviolet exposure through a patterned photomask. After development, Au was deposited onto the patterned substrate. The remaining photoresist and unwanted metal were removed through a lift-off process to form the designed electrode pattern. The resulting interdigitated electrodes had an electrode gap of 4 μm and an electrode width of 5 μm. The patterned electrodes were cleaned and dried before deposition of the sensing materials.

### Fabrication of sensing devices

For sensor fabrication, 6 mg of each sensing material was dispersed in 2 mL of absolute ethanol and sonicated for 10 min to obtain a homogeneous suspension. A 10 μL aliquot was withdrawn from the middle portion of the suspension and drop-cast onto the Au interdigitated electrodes. The coated devices were dried on a hotplate at 40 °C for 10 min and subsequently activated in a vacuum oven at 100 °C for 4 hours. The sensors were stored under dry conditions before measurement. Three independently fabricated devices were prepared for each sensing material to assess device-to-device reproducibility.

### Gas-sensing measurements

Gas-sensing measurements were conducted in a sealed 3D-printed chamber with an internal volume of 100 cm^3^. The sensor was connected to a source meter and operated at a constant bias voltage of 6 V. Electrical signals were recorded at a sampling rate of eight data points per second. Hydrogen sulfide (H_2_S), nitric oxide (NO), ammonia (NH_3_), phosphine (PH_3_), carbon dioxide (CO_2_), nitrogen dioxide (NO_2_), sulfur dioxide (SO_2_), methane (CH_4_), hydrogen (H_2_), and ethanol test gases were obtained from Air Products (UK). The test gases were diluted to the required concentrations using dry nitrogen or oil-free compressed air as the carrier gas. Gas concentrations were controlled using a mass-flow mixing system (GB-103, MCQ Instruments), while the total gas flow rate was maintained at 300 sccm. Unless otherwise specified, the measurements were performed at room temperature. The chamber atmosphere was not actively dehumidified, and the ambient relative humidity was approximately 15%.

The sensing response, (*S*), was calculated as

$$S(\%)=({R}_{{{{\rm{a}}}}}/{R}_{{{{\rm{g}}}}}-1)\times 100$$

where (*R*_a_) is the sensor resistance in the carrier gas and (*R*_g_) is the resistance after exposure to the analyte gas. The response time was defined as the time required for the resistance to reach 90% of its total change after introduction of the analyte gas. The recovery time was defined as the time required for the resistance to recover to 90% of its total change after removal of the analyte gas and reintroduction of the carrier gas.

### Wireless sensing and signal transmission

For wireless measurements, the sensing device was integrated with a low-power signal-acquisition and transmission platform. The resistance-dependent electrical signal from the sensor was acquired by the readout circuit, digitized by the control unit, and transmitted wirelessly to a receiving terminal for real-time visualization and storage. The sensing response was calculated using the same procedure as that used for the source-meter measurements. The wireless system was used for continuous monitoring during the application demonstrations. Detailed circuit architecture, component-level design and system-integration parameters are not disclosed because the device is currently the subject of a pending patent application.

### Egg-spoilage headspace measurements

Fresh eggs were boiled in water for 10 min and cooled to room temperature. The shells were removed to minimize variations arising from shell permeability and surface residues. Seven half-egg samples were individually placed in clean 20 mL glass vials, with one vial assigned to each storage interval. The vials were tightly sealed to minimize air exchange and approximate anaerobic storage conditions. The samples were stored in a closed container under dark conditions at room temperature for 0, 12, 24, 36, 48, 60, or 72 hours. At the selected time point, the corresponding vial was opened, and the sensor was immediately exposed to the released headspace gas at the vial opening. The sensor signal was continuously recorded to evaluate the evolution of H_2_S-related spoilage emissions with storage time.

### Structural and morphological characterization

The crystalline structures and phase compositions of the synthesized materials were examined by X-ray diffraction using Cu Kα radiation. Surface morphology and microstructure were investigated using a field-emission scanning electron microscope (Gemini 500, Zeiss) operated at an accelerating voltage of no more than 1 kV. Electron backscatter diffraction mapping was performed on the same scanning electron microscope at an accelerating voltage of 15.00 kV and a specimen tilt angle of 70°. The measurements were conducted with a hit rate of 43% and a data-acquisition rate of 3.62 Hz. Transmission electron microscopy was performed using a spherical-aberration-corrected FEI Titan 80–300 microscope operated at an accelerating voltage of 300 kV. Elemental compositions and spatial distributions were examined by energy-dispersive X-ray spectroscopy using an Oxford X-MaxN detector with an active area of 150 mm^2^.

### Surface chemical and electronic characterization

Surface elemental compositions and chemical states were analyzed by X-ray photoelectron spectroscopy using a PHI 5600 system equipped with an Al Kα X-ray source operated at 300 W. Ultraviolet photoelectron spectroscopy measurements were conducted using the same photoelectron spectroscopy system. Atomic orbital notations and corresponding XPS labels were formatted with the orbital symbols in italics and all accompanying superscripts and subscripts in roman font.

### Optical and vibrational characterization

Ultraviolet–visible absorption spectra were recorded using a Cary 100 Bio spectrophotometer (Varian) over the wavelength range of 200–400 nm. Raman spectra were recorded using an XPLORA PLUS spectrometer with a 532 nm green excitation laser. Raman mode labels were formatted using italic irreducible representations and roman subscripts. Fourier-transform infrared spectra, including in situ measurements where specified, were recorded using a Bruker Tensor II spectrometer equipped with a universal ZnSe attenuated-total-reflection accessory. The spectra were collected over the range of 400–4000 cm^−1^.

### Particle-size, surface-area and pore-structure measurements

Hydrodynamic particle-size distributions were measured by dynamic light scattering using a Zetasizer Nano-S90 analyser (Malvern Panalytical). Nitrogen adsorption–desorption isotherms were measured at −196 °C using an ASAP 2020 M analyser (Micromeritics). Before measurement, the samples were degassed under vacuum at 100 °C for at least 8 hours. The Brunauer–Emmett–Teller specific surface area (S_BET_) was calculated from the adsorption data in the relative-pressure range (P/P_0_ = 0.05–0.30). Pore-size distributions were determined from the adsorption branch using the Barrett–Joyner–Halenda method. The total pore volume was estimated from the nitrogen uptake at (P/P_0_ = 0.995).

### Electrochemical impedance spectroscopy

Electrochemical impedance spectroscopy measurements were performed using a VMP3 multichannel potentiostat (BioLogic). The impedance spectra were used to compare charge-transfer and interfacial electrical characteristics among the sensing materials.

### Electron paramagnetic resonance spectroscopy

Electron paramagnetic resonance spectra were recorded using an EMXplus-6/1 spectrometer (Bruker, Germany). Measurements were performed at a microwave frequency of 9.4061 GHz, a microwave power of 3.170 mW, a modulation frequency of 100 kHz, a modulation amplitude of 3.0 G, and a sweep width of 100 G.

### Computational methods

All static calculations were performed using the semi-empirical GFN2-xTB method, as implemented in xtb version 6.6.0. The calculations were used to evaluate interactions between gas molecules and the oligomeric ASA structures described in the Supplementary Information. Candidate adsorption configurations were generated using the automated Interaction Site Screening module. The screening procedure combined rigid-body docking based on the xTB-IFF force field with subsequent structural relaxation at the GFN2-xTB level. For each gas–adsorbent system, the energetically most favorable relaxed configuration was selected for further analysis. Long-range dispersion interactions were described using the DFT-D4 scheme. Partial atomic charges were evaluated using Mulliken population analysis.

### Software and data analysis

Electrical sensing and electrochemical impedance data were analyzed using OriginPro 2025. XPS and UPS spectra were processed using PHI MultiPak 9.9.0. SEM, EDS, and EBSD data were acquired and processed using ZEISS SmartSEM 8.0, Oxford Instruments AZtec 6.3 and AZtecCrystal 4.0, respectively. TEM images were processed using Digital Micrograph 3/Gatan Microscopy Suite. Raman, FTIR and EPR data were processed using HORIBA LabSpec 6, Bruker OPUS 9.3 and Bruker Xenon 1.1b60, respectively. DLS and nitrogen adsorption–desorption data were analyzed using Zetasizer Software 8.02 and MicroActive 7.01. Computational calculations were performed using xtb version 6.6.0. Figures were assembled using Microsoft PowerPoint 2024. No custom code was used.

### Statistics and reproducibility

Three independently fabricated devices were prepared for each sensing material to assess reproducibility. Sample sizes and error-bar definitions are provided in the relevant figure legends. The TEM image in Fig. 3b is representative of three independently prepared samples with similar results. No inferential statistical tests were performed.

### Reporting summary

Further information on research design is available in the Nature Portfolio Reporting Summary linked to this article.

## Supplementary information

Supporting Information

Description of Additional Supplementary File

Supplementary Data 1

Reporting summary

Transparent Peer Review file

## Source data

Source-data file

## Data Availability

The source data generated in this study and underlying the figures in the main manuscript and Supplementary Information are provided in the Source Data file. The computational input and output files generated in this study are provided in Supplementary Data 1. Source data are provided with this paper.
