# Supplementary material for: A robust high-performance H2S sensor enabling real-world wireless monitoring
Source: Nat Commun. 2026 Aug 19;17:10251. doi: 10.1038/s41467-026-76707-w (PMC13616922; doi:10.1038/s41467-026-76707-w)
Supplement: Supplementary file 1 [file 41467_2026_76707_MOESM1_ESM.pdf]

Supplementary Information for:

A robust high-performance H_2S sensor enabling real-world wireless monitoring

Wei Wang [1,2], Handan Wang [3], Leif Riemenschneider [1], Chenchen Wang [1], Juntang Dong [4], Shivam Singh [5,6], Yana Vaynzof [5,6], Darius Pohl [7], Markus Löffler [7], Helin Sun [1], Lin He [3], Anping Dong [3], Luis Antonio Panes-Ruiz [1], Bernd Rellinghaus [7], Chuanhui Huang [8]*, Shirong Huang [1,2]*, Gianaurelio Cuniberti [1,2,9]*

1 Institute for Materials Science and Max Bergmann Center for Biomaterials, TUD Dresden University of Technology, 01062 Dresden, Germany

2 Cluster of Excellence CARE, TU Dresden and RWTH Aachen, Germany

3 School of Materials Science and Engineering, Shanghai Jiao Tong University, 200240 Shanghai, PR China

4 School of Physics and Electronic Information, Yan'an University, 716000 Yan'an, PR China

5 Leibniz Institute for Solid State and Materials Research (IFW), 01062 Dresden, Germany

6 Chair for Emerging Electronic Technologies, TUD Dresden University of Technology, 01187 Dresden, Germany.

7 Dresden Center for Nanoanalysis (DCN), Center for Advancing Electronics Dresden (cfaed), TUD Dresden University of Technology, 01069 Dresden, Germany

8 Hangzhou Institute of Medicine (HIM), Chinese Academy of Sciences, 310022 Hangzhou, PR China

9 Dresden Center for Computational Materials Science (DCMS), TUD Dresden University of Technology, 01062 Dresden, Germany

Supplementary Table 1 summarizes representative H_2S sensors reported in the literature using key operating and performance metrics. To enable a more rigorous quantitative comparison beyond response alone, we introduce a normalized sensitivity metric, S/LOD, defined as the reported response (S, using each study's original response definition) divided by the corresponding LOD. This response-to-LOD ratio provides a joint metric reflecting response magnitude and detection limit based solely on literature-reported values, without requiring additional normalization to surface area or device geometry, which are often not\ consistently available across studies. The resulting S/LOD values highlight that the present device is competitive at the top tier among reported H_2S sensors when considering both response and detection limit simultaneously.

Supplementary Table 1 | Literature benchmarking of H_2S sensors with key metrics and normalized sensitivity (S/LOD).

Materials	T (°C)	Conc. (ppm)	Response (%)	LOD (ppm)	Res time	Response def. (S =)	Normalized sensitivity (S/LOD)	Ref
**ZnO nanosheet**	**23**	**100**	**13448.9**	**0.000328**	**7s**	**R_a/R_g-1**	**4.10×10^7**	**This work**
ZnO nanorods	25	0.05	1.7	-	23 min	R_a/R_g	-	[5]
Flower-like ZnO	26	5	581	-	320s	I_g/I_a	-	[6]
ZnO nanobelts	28	10	8	0.5	~100s	R_a/R_g	16	[7]
$Cs_2AgBiCl_6$	RT	10	90.6	0.005	99.6	$(I_g-I_a)/I_a$	1.81×10^4	[8]
Cu_2O/ZnO	RT	10	42	0.01	300	$(I_g-I_a)/I_a$	4.20×10^3	[9]
F_4-TCNQ/C_{10}-DNTT	RT	5	138	0.28	27.2 mins	$(\Delta R/R_0)$	4.93×10^2	[10]
CdS@PPy/rGO	RT	70	98.98	-	128	$(R_g - R_a) / R_a$	-	[11]
Ag@WO_3	RT	10	11420	0.01	7s	R_g/R_a	1.14×10^6	[12]
ZnO dendrites	30	100	17.3	10	15 s	R_a/R_g	1.73	[13]
ZnO nanorods	50	100	61.7	-	4s	-	-	[14]
ZnO nanosheet	70	100	23	5	252s	R_a/R_g	4.6	[15]
ZnO flowers	92	100	157.3	0.05	57.8 s	R_a/R_g	3.15×10^3	[16]
ZnO nanorods	100	100	2.6	-	-	R_a/R_g	-	[17]
SnO_2	125	5	335	0.5	224	R_{air}/R_{gas}	670	[18]
$MgFe_2O_4/MoO_3$	135	7	31.1	-	108	$(R_a-R_g)/ R_a$	-	[19]
ZnO tubes	150	450	15	-	-	R_a/R_g	-	[20]
ZnO nanowire	150	20	0.9	0.05	3min	$(R_a-R_g)/ R_a$	18	[21]
Lettuce-like ZnO	150	100	113	1	15s	R_a/R_g	113	[22]
Ru/SnO_2	160	20	310.1	100	<1	R_{air}/R_{gas}	3.10	[23]
α-Fe_2O_3	175	10	63.55	0.0025	82.7	$(R_a-R_g)/ R_a$	2.54×10^4	[24]
CuO/CeO_2	180	100	2010	10	39	R_{air}/R_{gas}	201	[25]

Materials	T (°C)	Conc. (ppm)	Response (%)	LOD (ppm)	Res time	Response def. (S =)	Normalized sensitivity (S/LOD)	Ref
Flower-like ZnO	180	50	42.2	1	14s	R_a/R_g	42.2	[26]
ZnO nanosheet	200	50	2180	0.01	3.4s	R_a/R_g	2.18×10^5	[27]
Mo doped $BiVO_4$	200	10	43.5	20.5	11	R_g/R_a	2.12	[28]
CuO/ZnO	200	5	83.5	tens	572	R_{air}/R_{gas}	-	[29]
WO_3/SnO_2	200	1	177	-	-	R_{air}/R_{gas}	-	[30]
ZnO hollow tubules	217	50	5.5	0.01	29s	R_a/R_g	550	[31]
Sphere-like ZnO	220	5	230	0.05	219s	R_a/R_g	4.60×10^3	[32]
ZnO nanoflakes	250	100	94	-	-	$(R_a-R_g)/ R_a$	-	[33]
α-Fe_2O_3	300	10	5.31	0.05	30	R_{air}/R_{gas}	106	[34]
ZnO	300	50	0.85	-	13s	$\Delta G/\Delta G_0$	-	[35]
ZnO	300	20	80	-	35s	$(R_a-R_g)/ R_a$	-	[36]
ZnO nanoclusters	330	0.5	3.3	0.0625	10s	R_a/R_g	52.8	[37]
ZnO chains	400	20	6300	5	-	$\Delta G/\Delta G_0$	1.26×10^3	[38]
ZnO nanorods	500	50	35	-	200s	$(I_g-I_a)/I_a$	-	[39]

Note: G = conductance; R = resistance; I = current; Oxygen species+H_2S mean that H_2S reacts with chemical abs oxygen, also react with ZnO to produce ZnS. Normalized sensitivity (S/LOD) is calculated as the reported response divided by the corresponding LOD (both taken directly from the literature), serving as a joint metric reflecting response magnitude and detection limit.

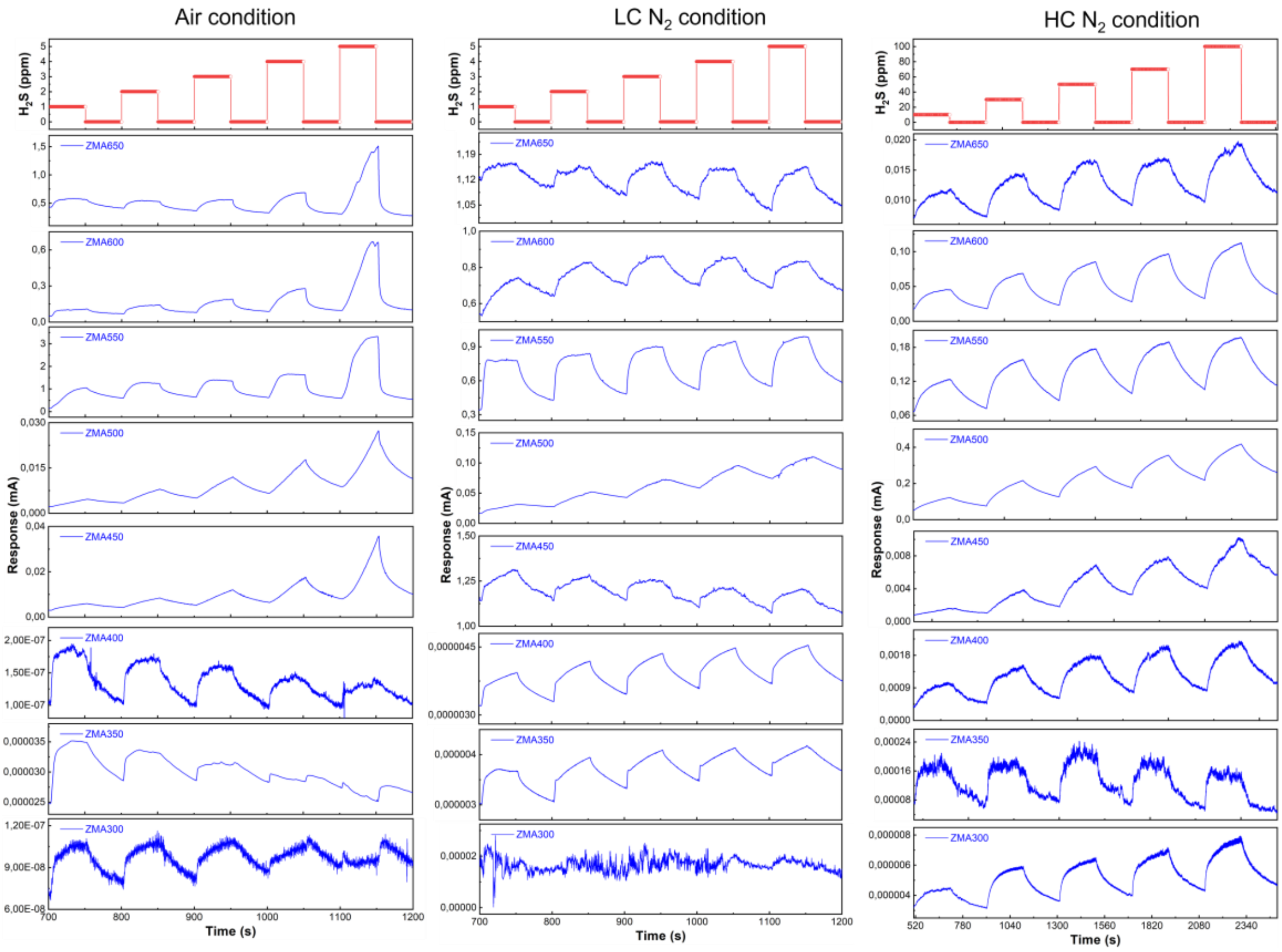

Supplementary Fig. 1 | Response and recovery curves of all samples towards H_2S under air and N_2 atmospheres at high (HC) and low (LC) concentrations.

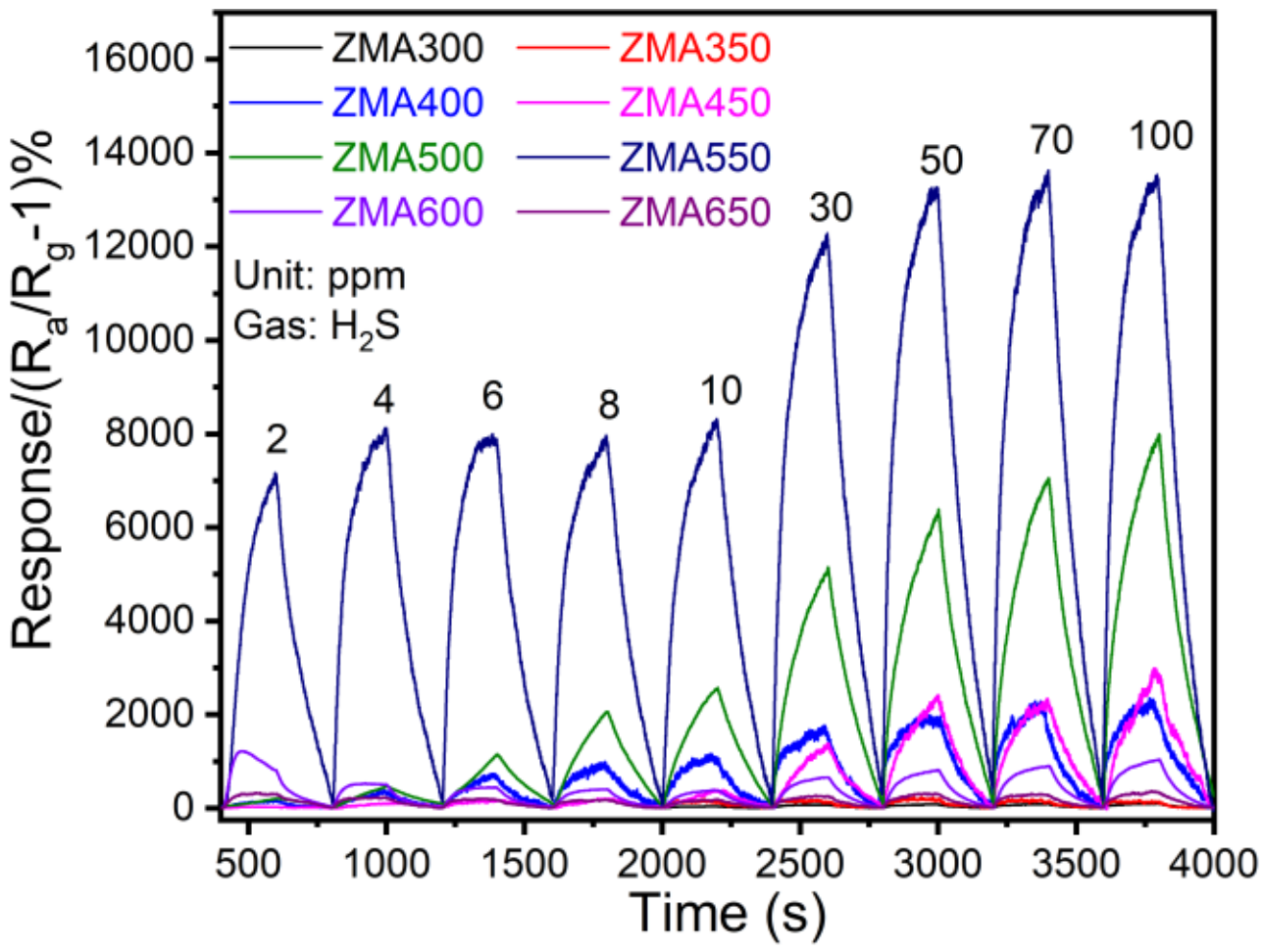

Supplementary Fig. 2 | Response and recovery curves of all samples towards H_2S at high concentrations under N_2 atmosphere.

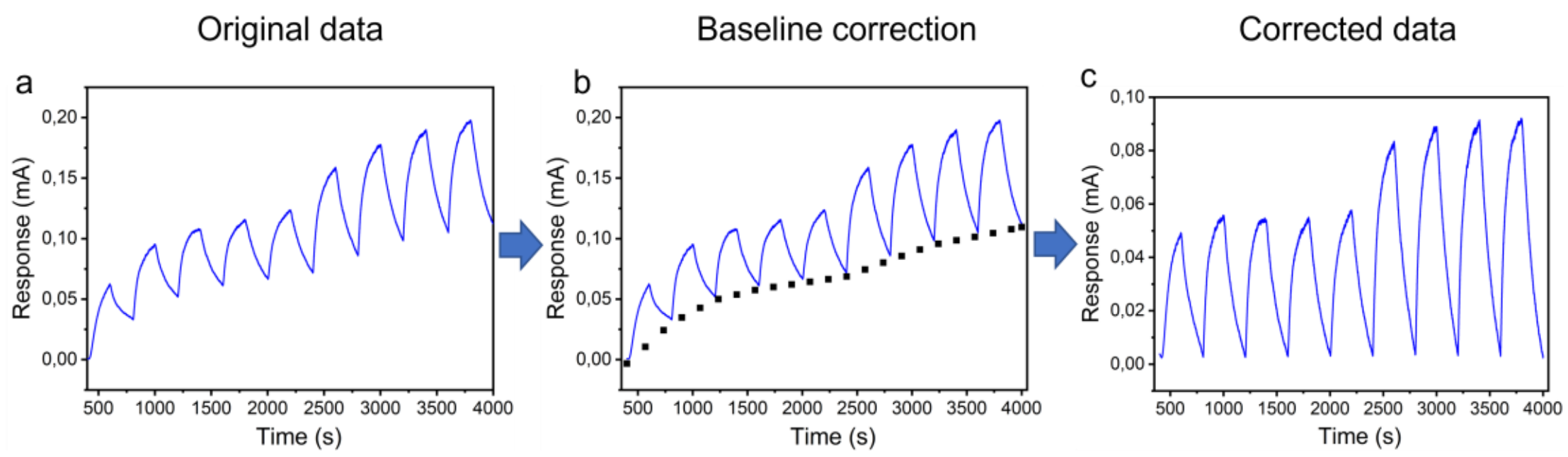

Supplementary Fig. 3 | Baseline Correction: **a,** The raw current response curves of the ZMA550-based sensor to varying concentrations of H_2S at room temperature. **b,** Baseline anchoring of the raw data using zero points from the 2nd derivative. **c,** The current response curves after baseline correction.

In the extended concentration range of 200-1000 ppm, the sensor maintains a linear response up to 900 ppm, with an increased slope observed beyond 900 ppm. At 1000 ppm, the response remains high but exhibits a significantly higher value than expected from the lower concentration range, suggesting that the sensor continues to exhibit a high response without reaching saturation, but with a different linear regime beyond 900 ppm. The change in slope at concentrations above 900 ppm indicates that the sensor's sensitivity is reduced in this range due to the occupation of reactive adsorption sites (Supplementary Fig. 4).

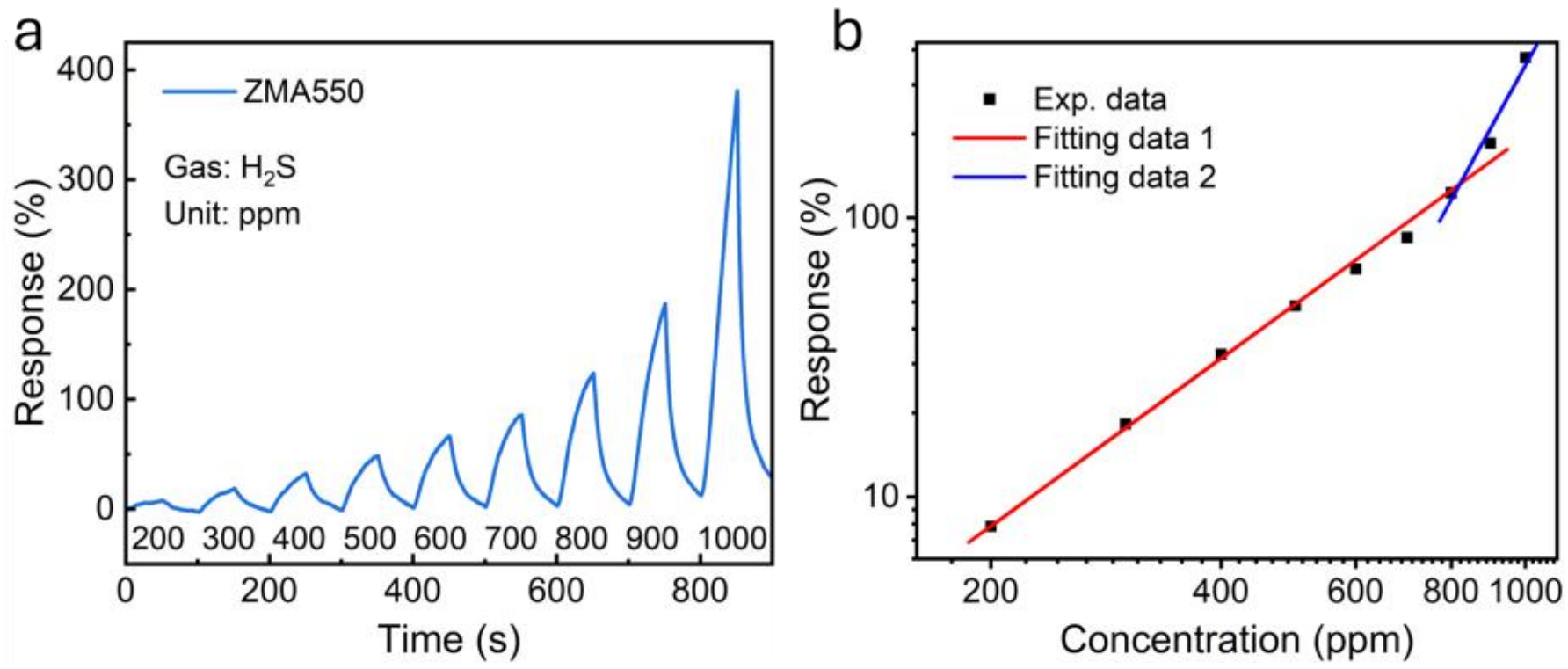

Supplementary Fig. 4 | a, Dynamic response and recovery curves of the ZMA550 sensor exposed to varying high H_2S concentrations (200-1000 ppm) air condition. **b,** Response concentration log-log plots to different concentrations of H_2S.

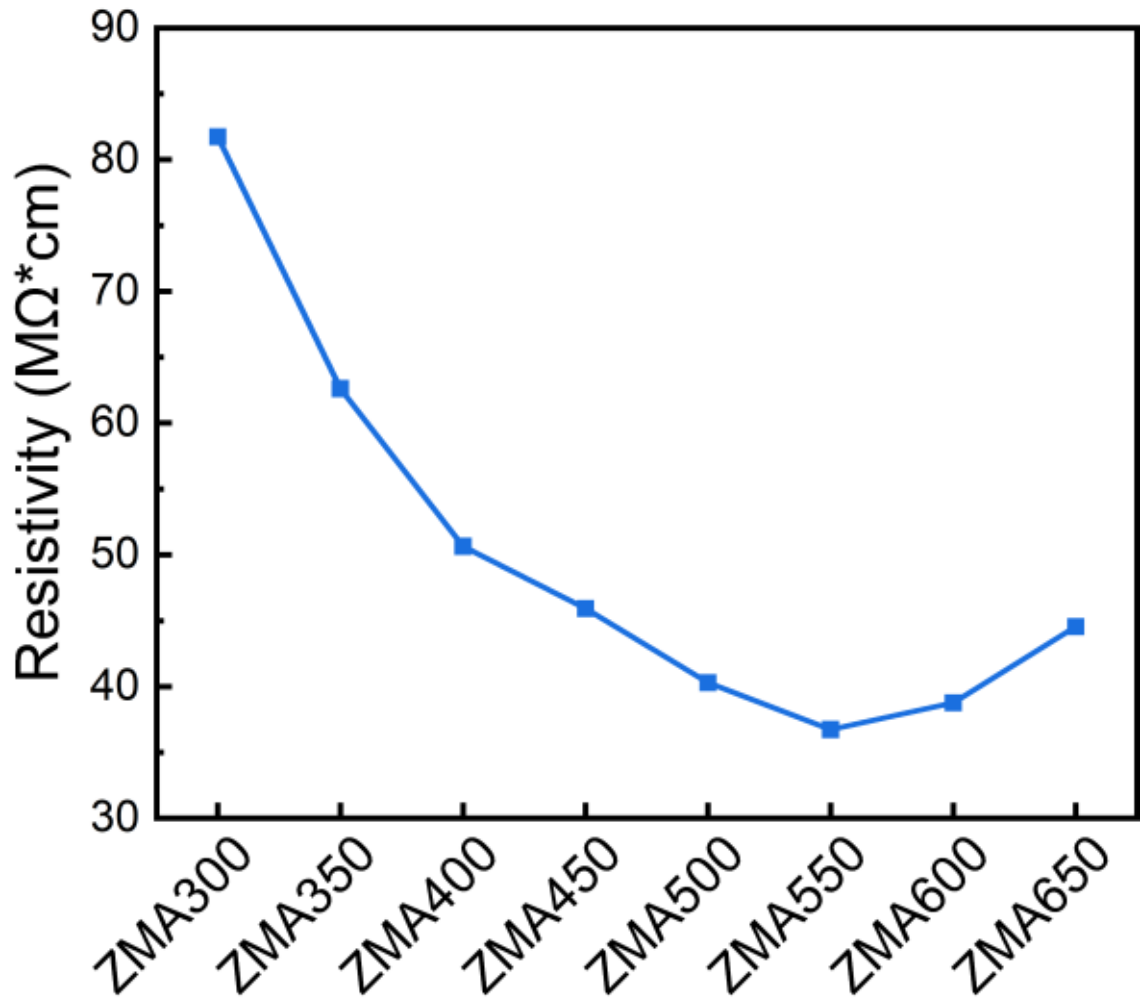

Supplementary Fig. 5 | Electrical resistivity of all as-prepared samples.

The sensor response at each H_2S concentration was obtained from the time-resolved current curves by extracting the maximum response amplitude during H_2S exposure (Supplementary Fig. 6a). The concentration–response relationship was constructed as a log–log plot and fitted using a linear model:

$$\lg(\text{Current}) = k \cdot \lg(C) + b \qquad (2)$$

where C is the H_2S concentration and k is the slope (sensitivity in the log–log domain) used for LOD estimation. The baseline noise (RMS) was quantified from the current–time signal recorded under air conditions. To remove slow instrumental drift without affecting high-frequency noise, the baseline was fitted using a low-order (5th-order) polynomial, and the RMS was calculated from the residuals ($Y_i - Y_{fit}$) (Supplementary Fig. 6b). This procedure serves only to isolate stochastic noise and does not involve derivative-based baseline anchoring [40]. The LOD is then calculated using the 3σ method:

$$\text{LOD} = 3.3\ \delta / S \qquad (3)$$

where δ represents the standard deviation of the baseline noise extracted from the sensor signal under air background gas at room temperature, and S denotes the sensitivity, defined as the slope of the linear fitting of the sensor response versus H_2S concentration in the low-concentration regime. This approach ensures that the LOD is intrinsic to the sensor's response to H_2S, with no dependence on arbitrary baseline correction procedures.

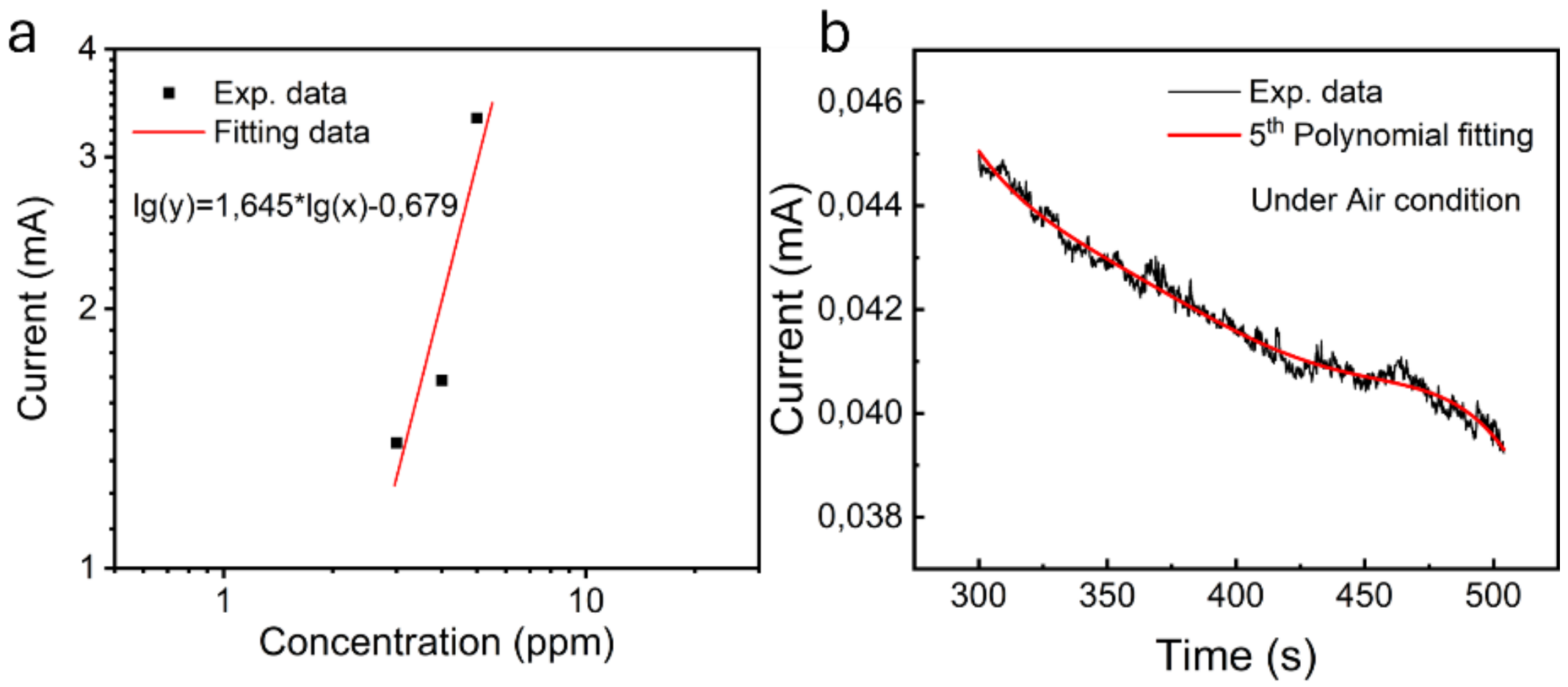

Supplementary Fig. 6 | a, Response concentration log-log plots to different concentrations of H_2S under air condition. **b,** RMS noise levels of response as a function of time under air conditions.

To complement the calculated LOD, direct H_2S exposure measurements were performed at low concentrations of 10, 20, 30, 40, and 50 ppb, corresponding to the lowest concentrations that could be reproducibly generated under the current gas-mixing conditions.

As shown in Supplementary Fig. 7, the sensor exhibits clear and distinguishable response–recovery behavior across the entire tested concentration range. Notably, even at 10 ppb, the response signal remains significantly above the baseline noise level, indicating reliable signal discrimination at the ppb level.

Furthermore, the sensor response shows a consistent increase with increasing H_2S concentration, demonstrating a well-defined concentration-dependent behavior in the low-concentration regime.

To evaluate repeatability, 8 consecutive response–recovery cycles were conducted at 30 ppb. The results show highly reproducible response amplitudes and stable baseline recovery without noticeable signal drift, confirming the robustness and stability of the sensor under repeated low-concentration exposure.

These results provide direct experimental validation of the sensor's ppb-level sensing capability. It should be noted that the previously reported value of 0.328 ppb represents a calculated LOD derived from baseline noise and calibration sensitivity, whereas the present measurements define the experimentally accessible detection range under practical operating conditions.

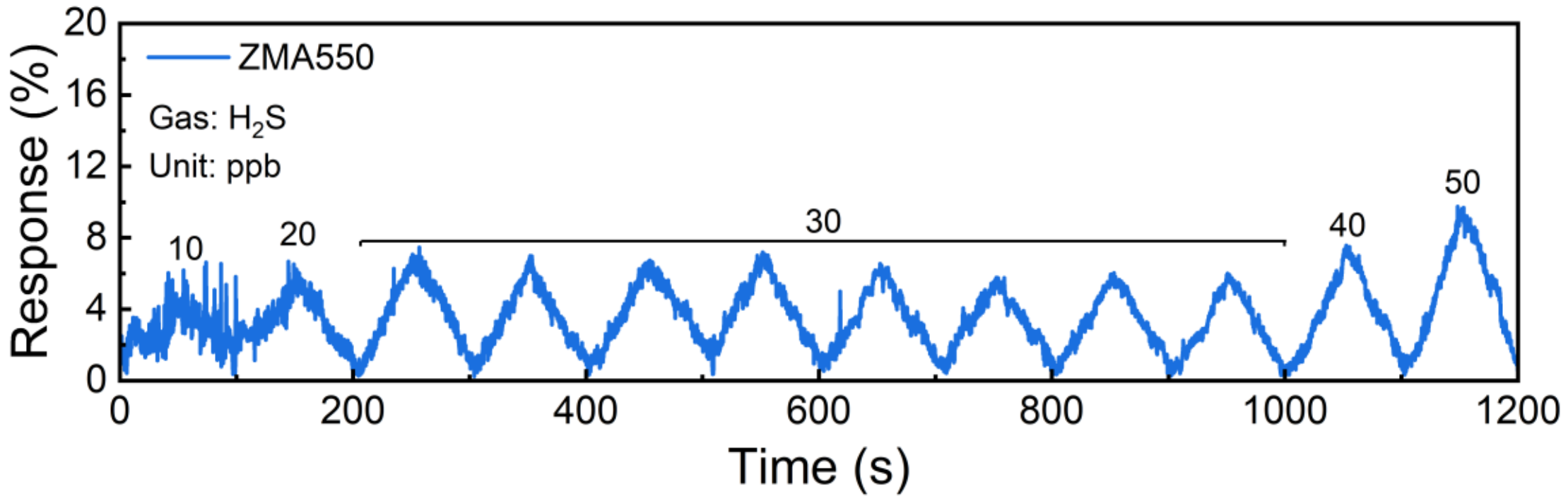

Supplementary Fig. 7 | Dynamic response and recovery curves of the ZMA550 sensor exposed to varying lower H_2S concentrations (10-50 ppb) under air condition.

The statistical analysis of three independently fabricated sensor batches is summarized below, including the response values, LODs and corresponding standard deviations. All fabrication and measurement conditions were kept consistent. One independently fabricated sensor from each batch was evaluated, giving a total sample size of n = 3 sensors.

The response values for the three sensor batches are as follows: 529.52%, 629.75%, 548.04%

The standard deviation for these values is calculated to be 43.55.

The LOD for each batch of sensors was calculated as follows: 0.0909 ppb, 0.0356 ppb, 0.0729 ppb

The standard deviation for these LOD values is 0.0230 ppb.

The response values show a reasonable variation between the three sensor batches, with a standard deviation of 43.55. This reflects the inherent variation that can occur between different batches of sensors, even when experimental conditions are held constant.

The LOD values, calculated based on the baseline stability and response sensitivity via using the same methodology, show a similar trend, with a standard deviation of 0.0230 ppb. These values are lower than the previous LOD of 0.328 ppb. This decrease can be attributed to improved baseline stability and enhanced response sensitivity. The baseline stability was improved by fitting the baseline data with a 5th-order polynomial, which minimized fluctuations and noise, resulting in smaller residuals. This allowed for more precise detection of small signal variations. Additionally, the increased response sensitivity, reflected in a steeper slope of the response-concentration curve, indicates that the sensor is now more responsive to small changes in concentration. These improvements in baseline stability and response sensitivity together contributed to the lower LOD values. Error bars representing the standard deviations for the response values and LOD values are shown in Supplementary Fig. 8. The error bars visually indicate the variations across the three sensor batches, providing a clear representation of the data's uncertainty.

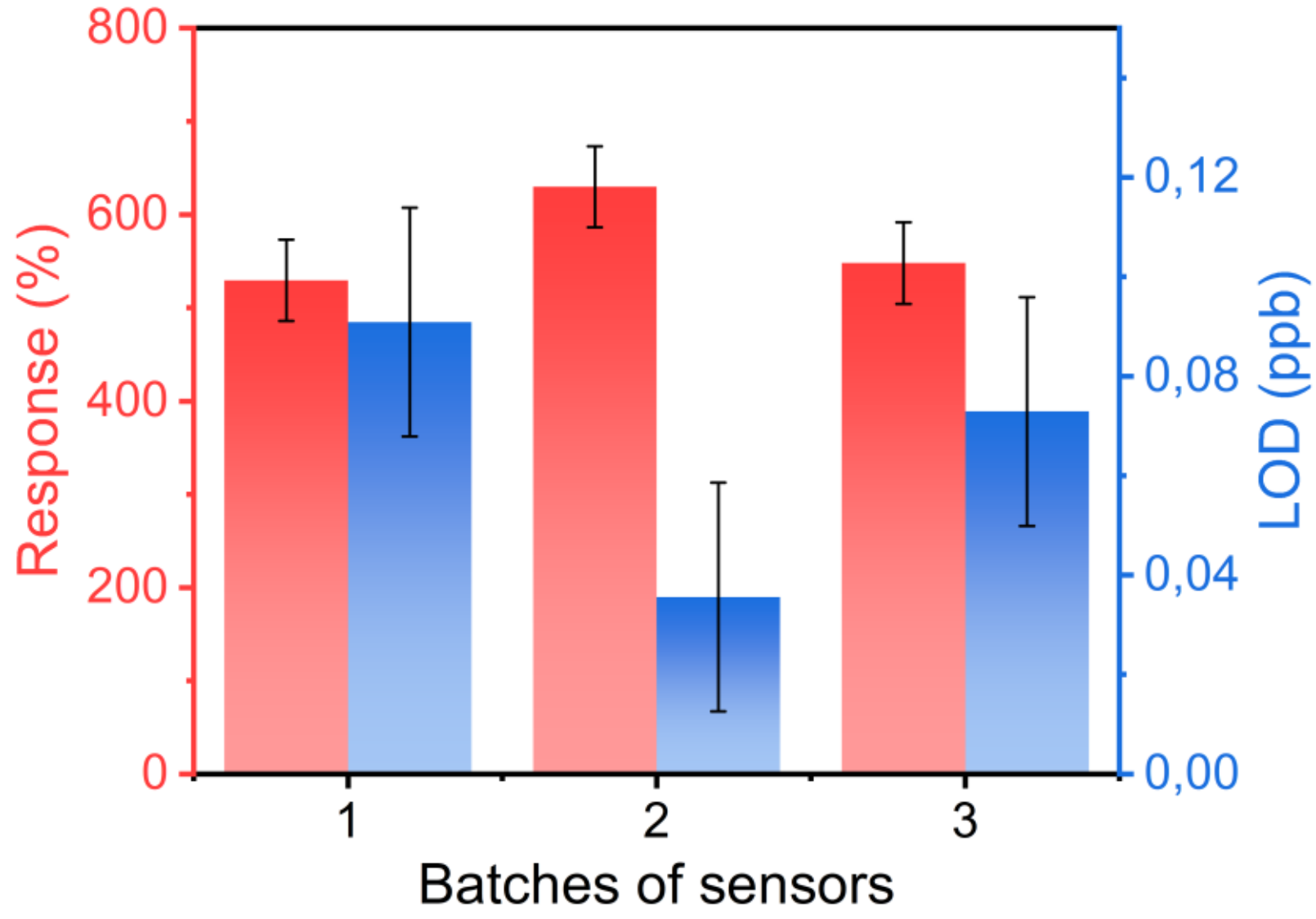

Supplementary Fig. 8 | Reproducibility of the sensor response and limit of detection across three independently prepared sensor batches. Bar chart with of sensor response (left Y-axis) and LOD (right Y-axis) for three sensor batches. Error bars denote s.d. (n = 3 independent sensor batches, one sensor per batch).

The humidity was continuously monitored using a calibrated Bosch BME280 sensor, and a schematic of the humidity-controlled sensing setup has been added (Supplementary Fig. 9). It is worth noting that the response amplitude of the sensor to 1 ppm H_2S exhibits a gradual increase with increasing relative humidity, as consistently observed over repeated response–recovery cycles at each humidity level (Supplementary Fig. 10). This trend suggests that moderate humidity may facilitate the surface reaction kinetics or charge transfer processes involved in H_2S sensing.

To elucidate the underlying mechanism, XPS measurements were performed on the sensing layers after operation at different humidity levels, with particular focus on the O 1*s* region (Supplementary Fig. 11). The analysis reveals no significant change in the lattice oxygen (O_1) peak, while the intensity of the hydroxyl-related oxygen species (O_3) peak increases with humidity. This indicates that water vapor primarily influences the formation of hydroxyl-related oxygen species on the surface, rather than substantial modification of the lattice oxygen or irreversible water-induced surface reconstruction. Thus, humidity modulates the sensing response through surface hydroxylation, without affecting the core Schottky-barrier-modulated sensing mechanism.

In the pristine samples, the O 1*s* envelope was deconvoluted into lattice oxygen (O_1, ~530.0 eV), oxygen vacancies (O_2, ~531.1 eV), and loosely bound surface oxygen species (O_3, ~532.2 eV) (Supplementary Fig. 28b and c). Following sensing tests under varying humidity conditions, the O 1*s* spectrum was dominated by O_1 (lattice oxygen, ~530.9 eV) and O_3 (hydroxyl/adsorbed water-related oxygen species, ~532.9 eV). The emergence of O_3 correlates with humidity-induced surface hydroxylation and the formation of H_2O-related species, whereas the relative intensity of the oxygen vacancy-related signal (O_2) is not explicitly observed due to the overlap with hydroxylated oxygen species. Importantly, these changes do not diminish the central role of oxygen vacancies in the sensing mechanism. Oxygen vacancies remain the primary reactive sites for H_2S adsorption, and the increased O_3 component highlights the surface modification that occurs in the presence of humidity.

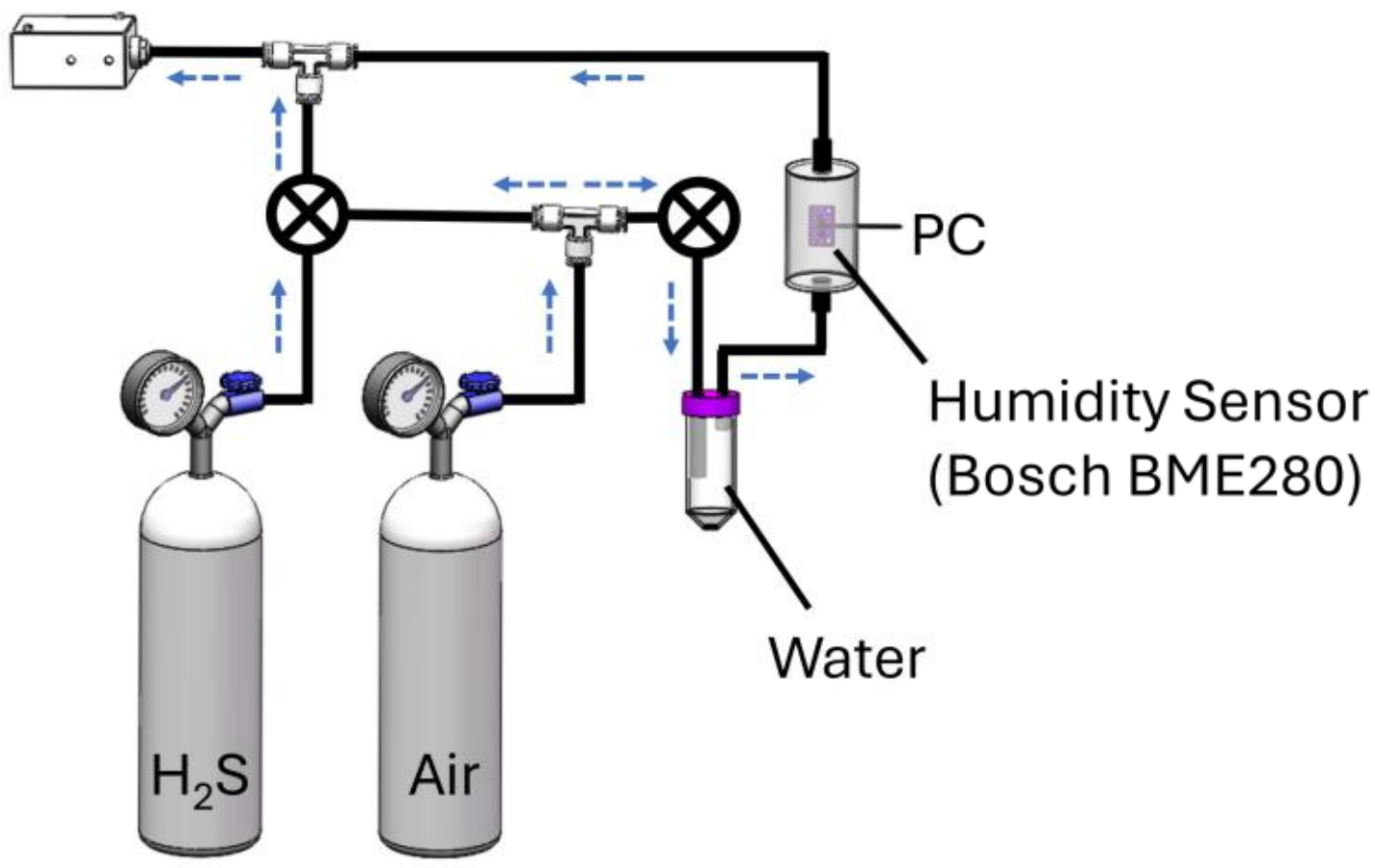

Supplementary Fig. 9 | Schematic diagram of humidity introduction in sensing tests.

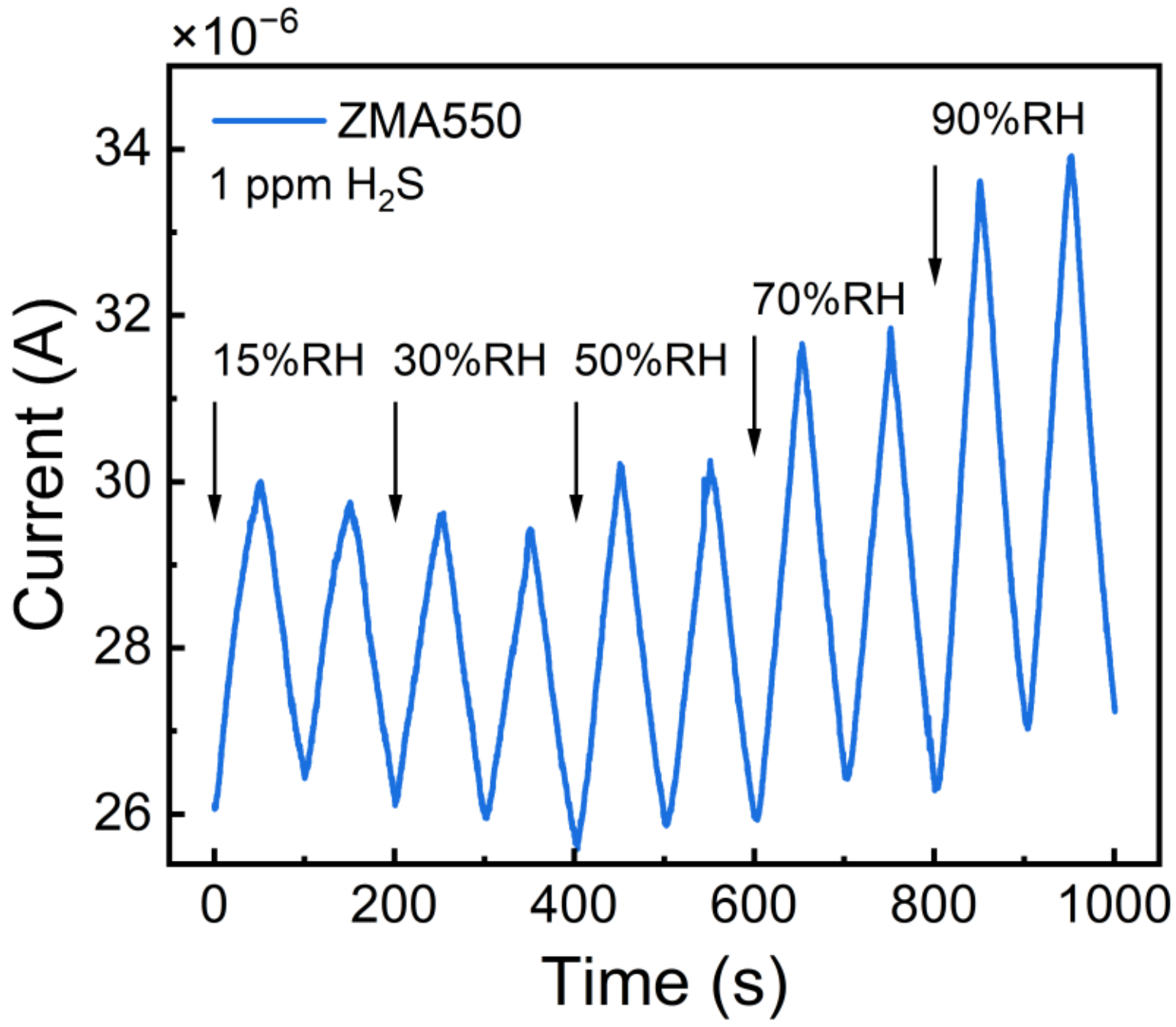

Supplementary Fig. 10 | Current response and recovery curves of ZMA550 sensor towards 1 ppm H_2S at different humidity levels from 15%RH-90%RH.

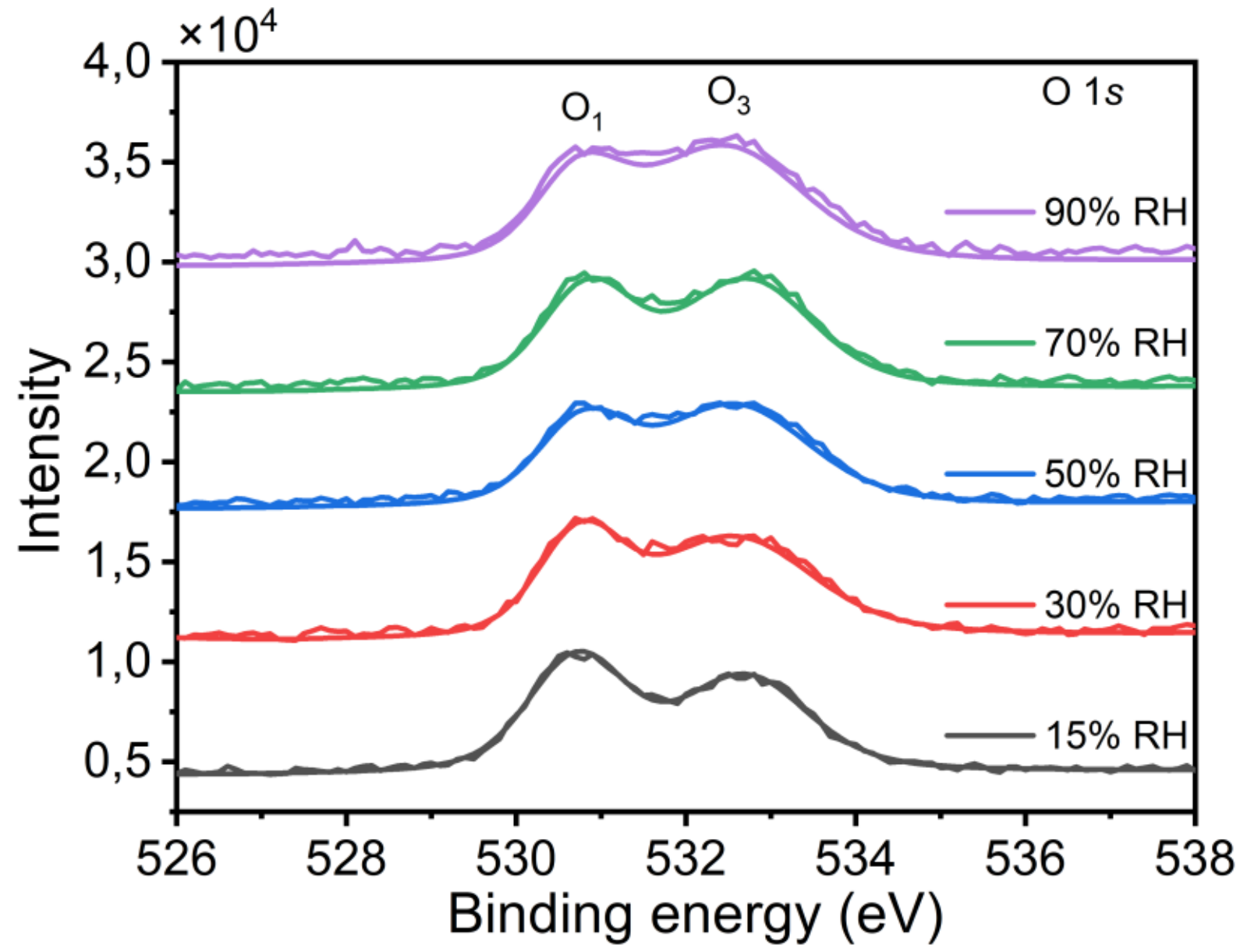

Supplementary Fig. 11 | O 1*s* XPS results of sensing tests under different humidity levels.

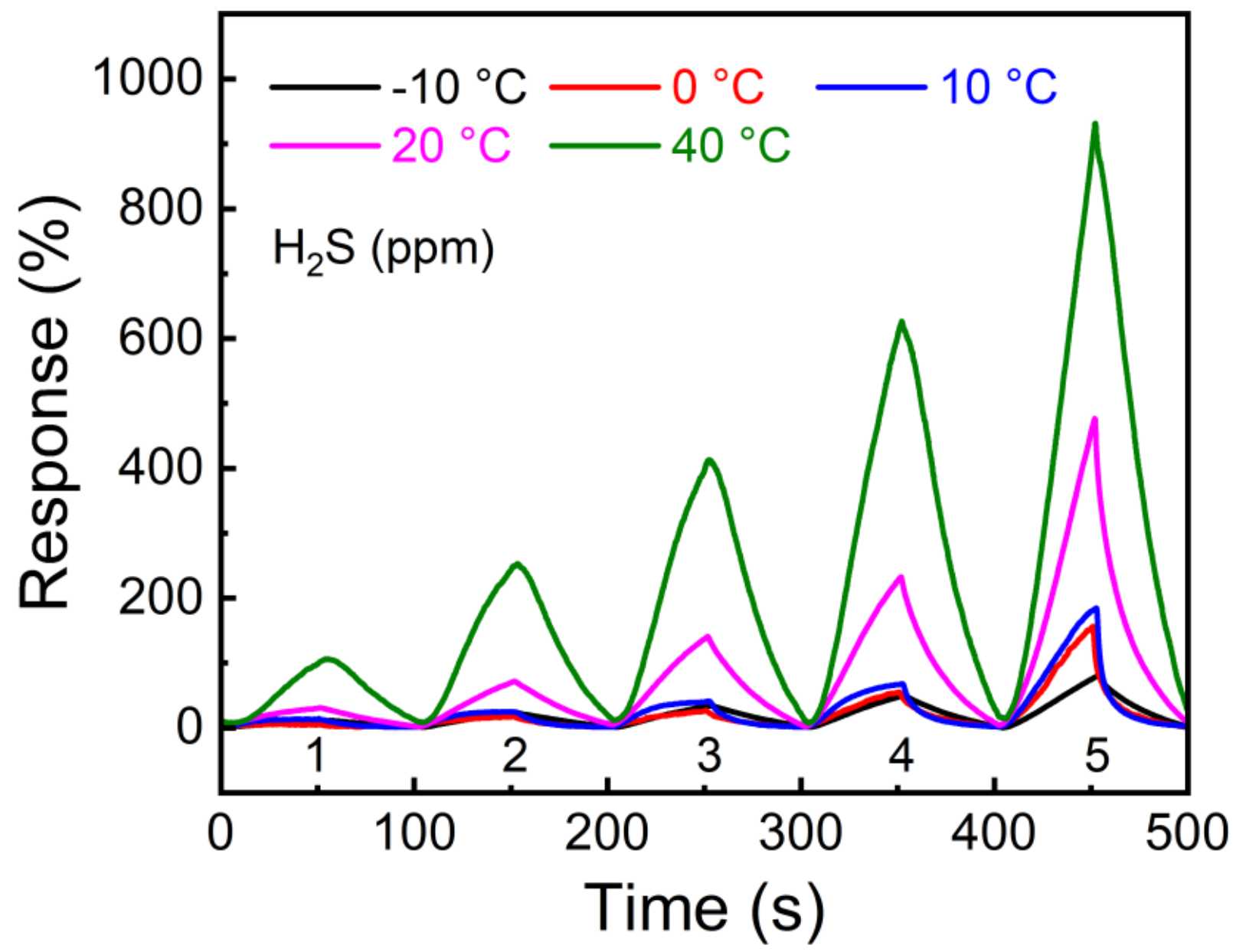

Supplementary Fig. 12| Response and recovery curves of ZMA550 sensor exposed to varying high H_2S concentrations (1-5 ppm) under different operation temperature.

To examine whether long-term operation induces sulfidation or secondary-phase formation, the sensing layer after the stability campaign was characterized by HRTEM, PXRD, XPS, and EDX elemental mapping. HRTEM images revealed clear lattice fringes characteristic of ZnO and no ZnS crystalline domains, confirming that the ZnO structure remains intact at the nanoscale (Supplementary Fig. 13a). PXRD patterns did not show any additional peaks corresponding to ZnS, excluding the formation of the ZnS crystalline phase (Supplementary Fig. 13b). XPS analysis revealed a weak sulfur signal in the S 2*p* region at 169 eV, characteristic of SO_4^{2-} species, but no peaks indicative of ZnS formation were observed (Supplementary Fig. 13c). EDX mapping confirmed the absence of sulfur-rich ZnS domains or phase segregation (Supplementary Fig. 13d). These results collectively confirm that the long-term sensing process under our testing conditions is not accompanied by detectable bulk ZnS formation.

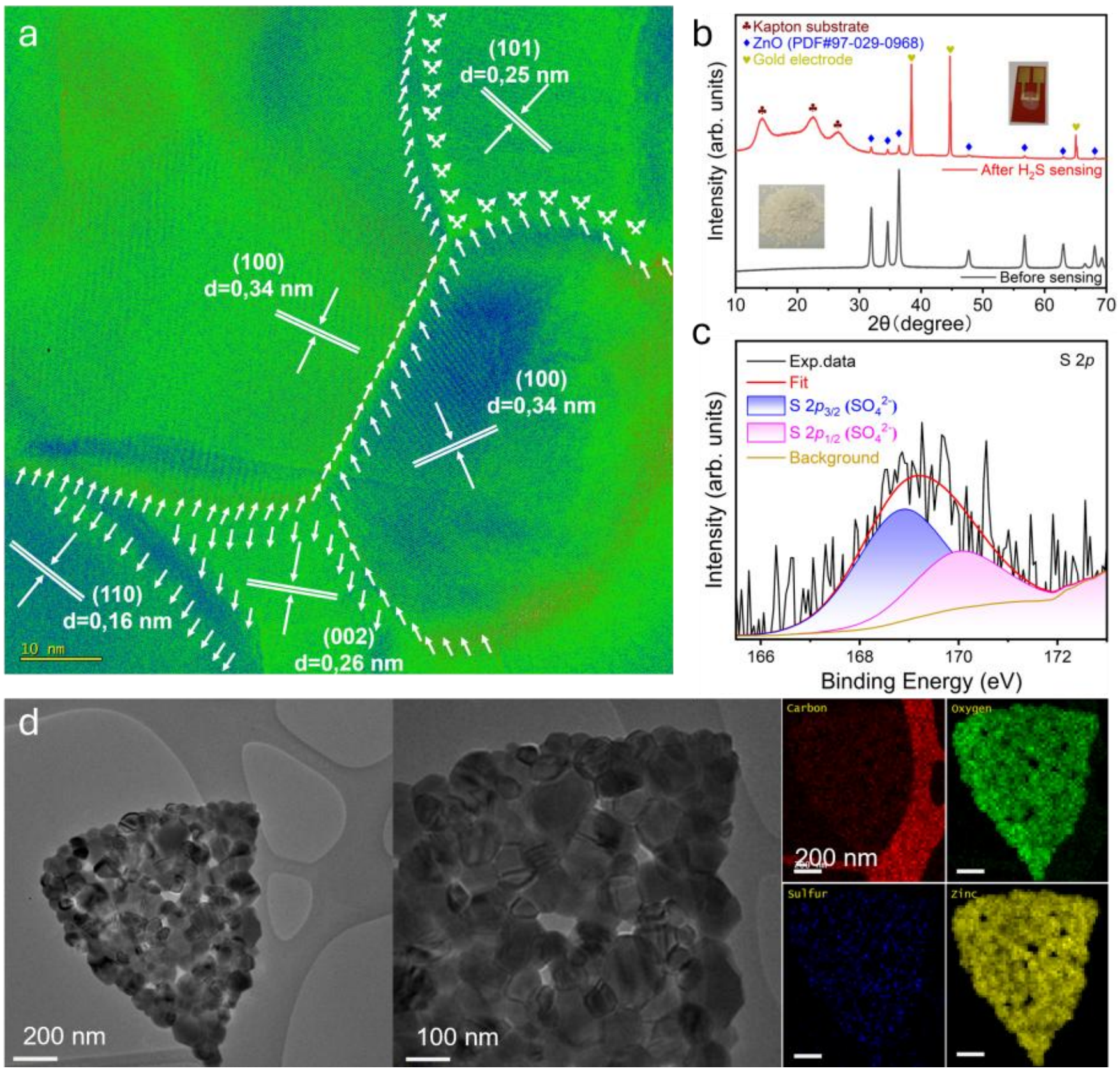

Supplementary Fig. 13 | HRTEM, PXRD, XPS, and EDX characterization of the sensing layer after long-term exposure to H_2S. **a,** HRTEM image showing lattice fringes characteristic of ZnO without ZnS formation. **b,** PXRD pattern confirming no ZnS phase formation. **c,** XPS S 2*p* spectrum revealing SO_4^{2-} species and the absence of ZnS peaks. **d,** EDX elemental mapping showing homogeneous elemental distribution without sulfur-rich ZnS domains.

The structural and surface evolution of the sensing layer after long-term cycling was systematically investigated. TEM, SEM and EDX images of the sensing layer after 450-day cycling tests (Supplementary Figs. 14a-e) show that the grain morphology and grain boundary network are well preserved, with no evidence of grain coarsening, boundary relaxation, or structural degradation. This indicates that the microstructural framework responsible for carrier transport remains stable during prolonged operation.

XPS analysis of the cycled samples (Supplementary Figs. 14f-h) further reveals detectable changes in the S 2*p*, O 1*s*, and Zn 2*p* spectra after long-term cycling. The S 2*p* spectra show variations in sulfur-related surface species, while the O 1*s* spectra indicate modulation of oxygen-related surface states. Meanwhile, slight changes in the Zn 2*p* spectra suggest a corresponding adjustment of the local Zn chemical environment. Together, these results point to mild surface chemical evolution during repeated sensing cycles, rather than severe irreversible chemical degradation.

Notably, despite the detectable accumulation of sulfur-containing species, the sensing performance remains highly stable over 450 days. This suggests that these species do not form an irreversible passivation layer or block active sites. Instead, they are likely weakly bound or dynamically exchangeable under operating conditions, maintaining access to reactive sites and preserving charge transport pathways.

Therefore, the sensing interface can be described as a quasi-reversible system, in which surface chemical states undergo continuous yet non-destructive evolution. The structural stability of grain boundaries, combined with the dynamic equilibrium of surface adsorbates, ensures that the interfacial barrier modulation and carrier transport mechanism remain functional during long-term operation.

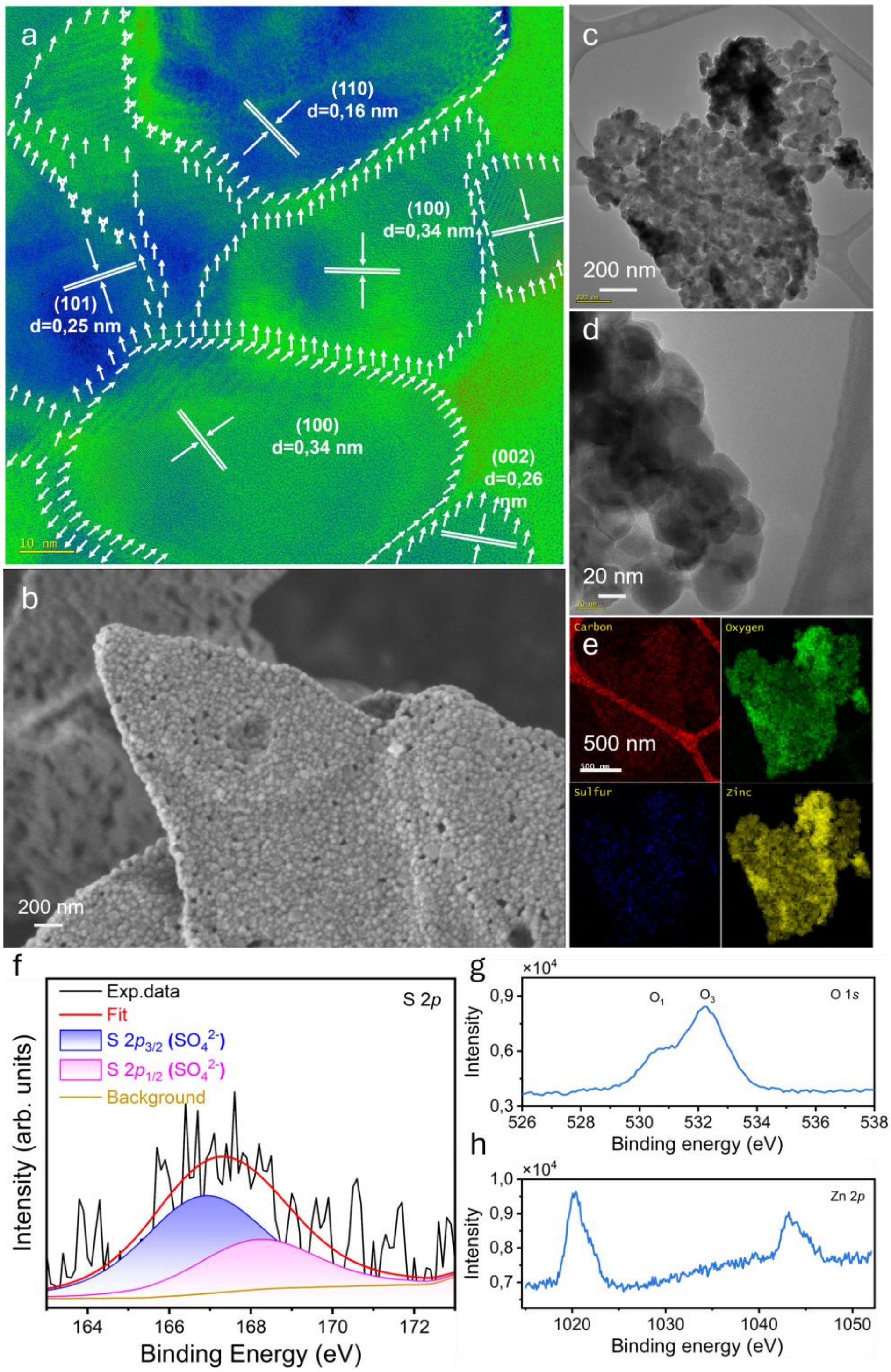

Supplementary Fig. 14 | HRTEM, SEM, EDX and XPS characterization of the sensing layer after the 450-day long-term cycling test. **a,** HRTEM image showing lattice fringes characteristic of ZnO. **b,** SEM images still keep nanosheet structure composed of ZnO particles. **c-e,** TEM and EDX elemental mapping showing homogeneous elemental distribution. **f-h,** XPS S 2*p*, O 1*s*, and Zn 2*p* spectra revealing reversibility and structural stability of the sensing layer under long-term use.

To evaluate the robustness of the H_2S sensing signal under realistic multi gas environments, we conducted a dynamic interference stability test by repeatedly exposing the sensor to 3 ppm H_2S over 18 consecutive response–recovery cycles. During each cycle, one set of interfering gases was introduced simultaneously with H_2S, while all other experimental parameters (e.g., concentration control, total flow rate, temperature, and bias/measurement settings) were kept constant. In particular, to directly address the coexistence of oxidizing and reducing species, we tested representative oxidizing gases (NO_2, SO_2) and a reducing gas (NH_3) both individually and in mixtures, including NO_2 + SO_2, NO_2 + NH_3, SO_2 + NH_3, and NO_2 + SO_2 + NH_3. The sensor exhibits highly reproducible H_2S response amplitudes with negligible baseline drift across all cycles, indicating that the sensing output is dominated by H_2S-triggered interfacial modulation rather than transient cross-interference from coexisting gases (Supplementary Fig. 15). A schematic of the gas-introduction protocol and the overall setup is provided in Supplementary Fig. 16, and a photograph of the actual experimental setup is shown in Supplementary Fig. 17 to ensure transparency and reproducibility.

In addition to the inorganic interferents above, we further evaluated potential cross-sensitivity to representative VOC interferents that are commonly reported in food spoilage headspaces, particularly during egg fermentation. These VOCs include amines (triethylamine, propylamine, butylamine), organic acids (acetic acid and butyric acid), sulfur-containing species (dimethyl sulfide), and small oxygenated molecules (formaldehyde, ethanol, and acetone). The sensor maintains stable and reversible H_2S responses under these VOC interference conditions as well (Supplementary Fig. 15), further supporting its robustness in complex gas matrices.

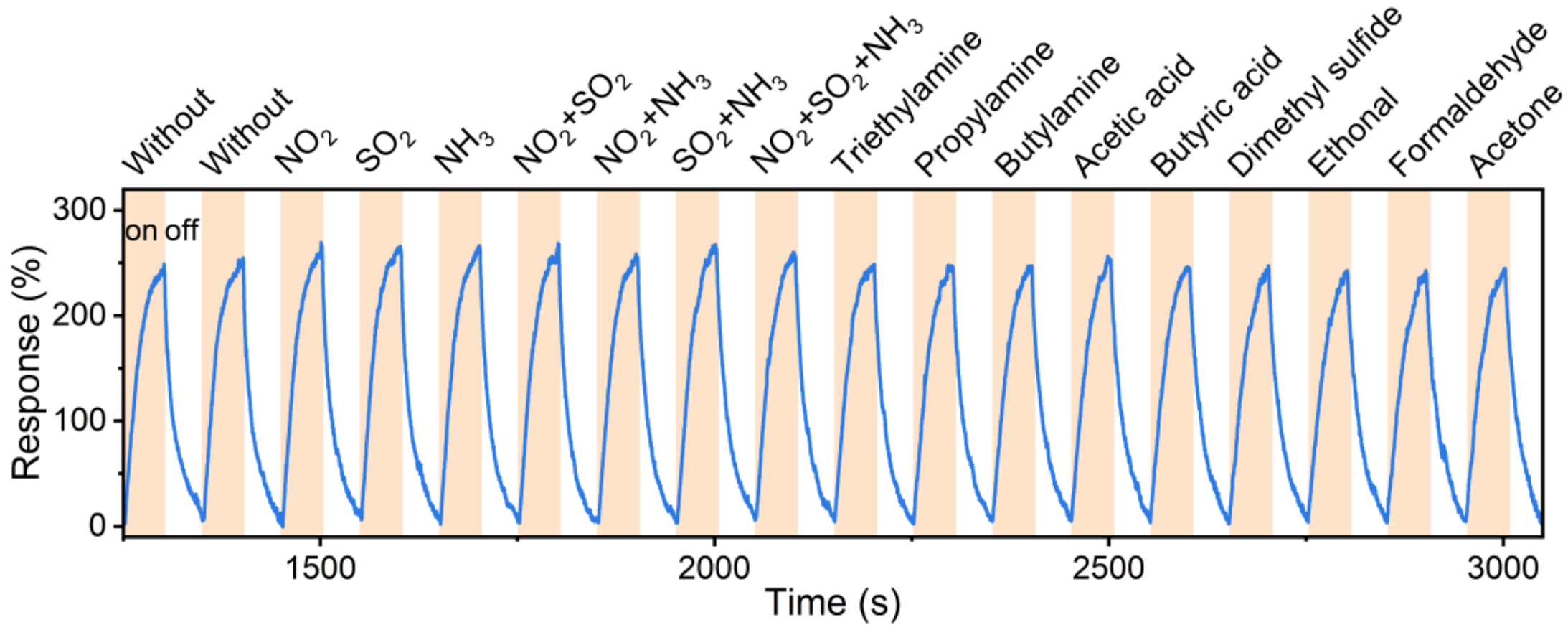

Supplementary Fig. 15 | Dynamic interference stability of the H_2S sensor.

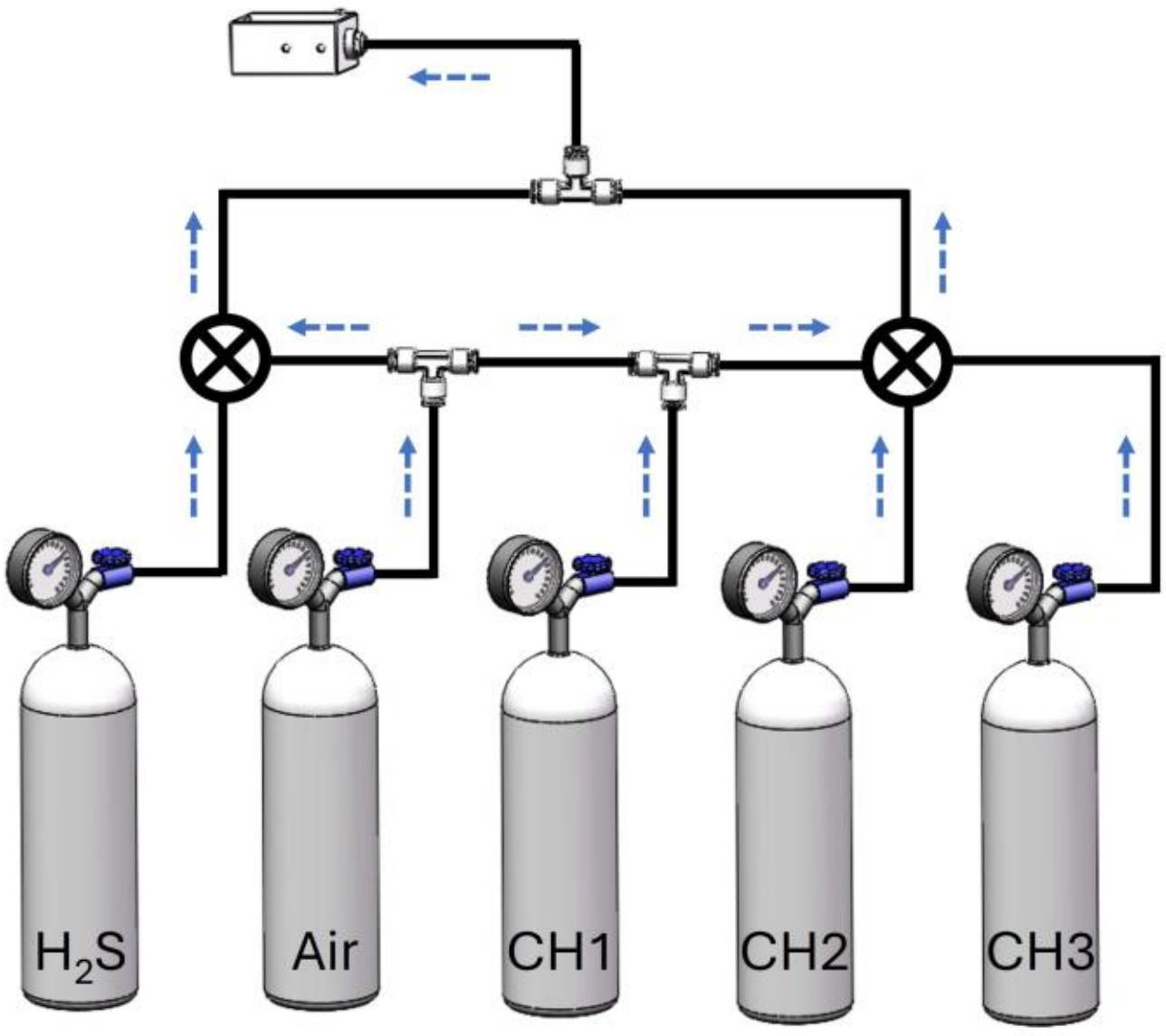

Supplementary Fig. 16 | Experimental setup for dynamic interference stability measurements.

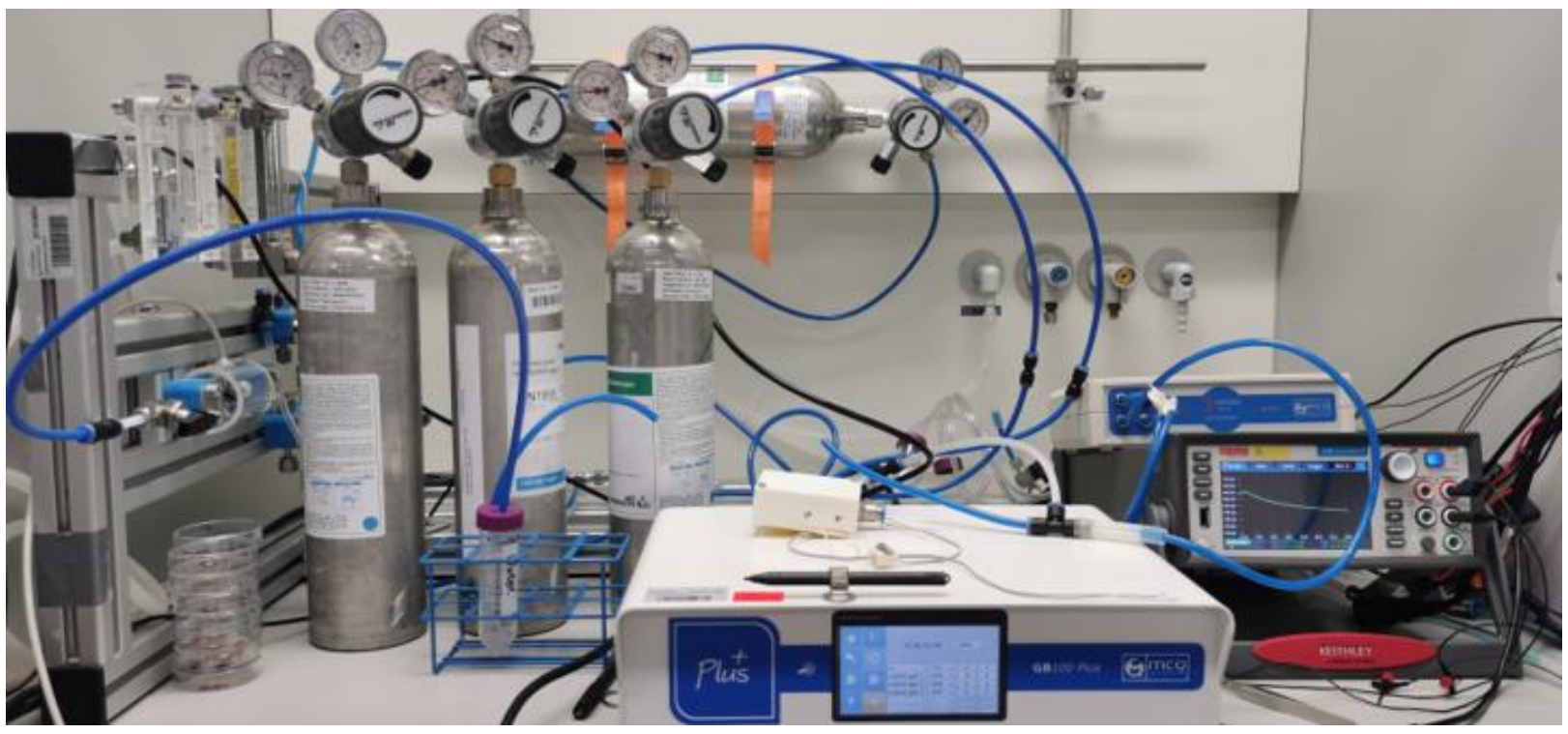

Supplementary Fig. 17 | Photograph of the actual experimental setup.

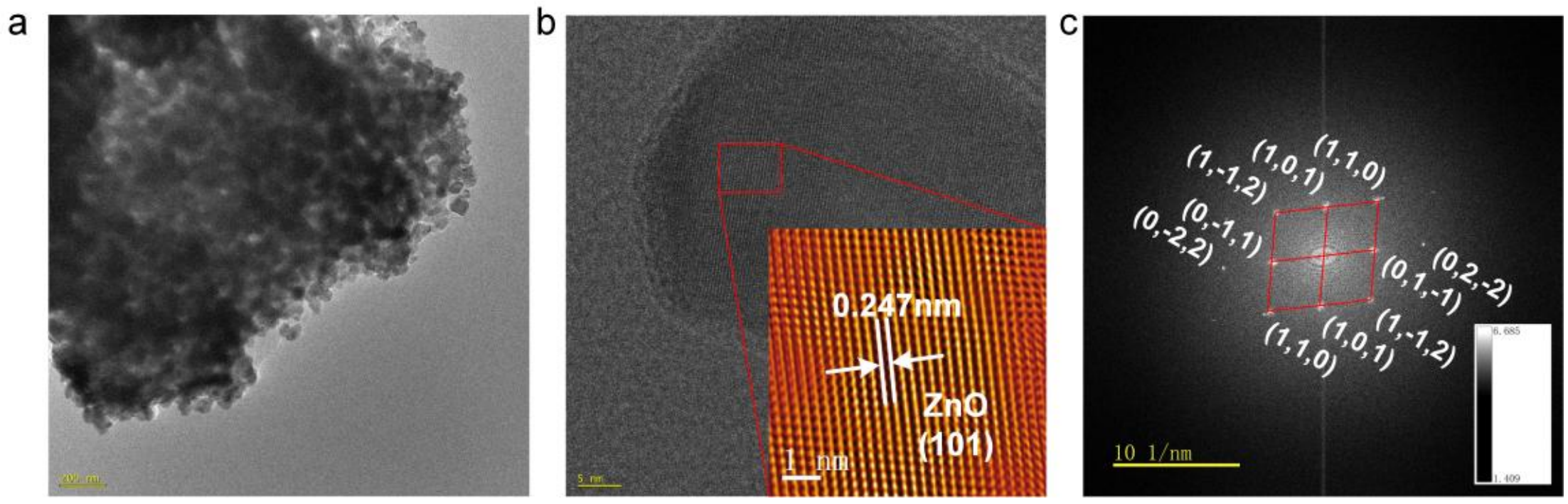

Supplementary Fig. 18 | Structural analysis of ZMA550: **a,** TEM image, **b,** high-resolution TEM (HRTEM) image, and **c,** FFT of HRTEM image.

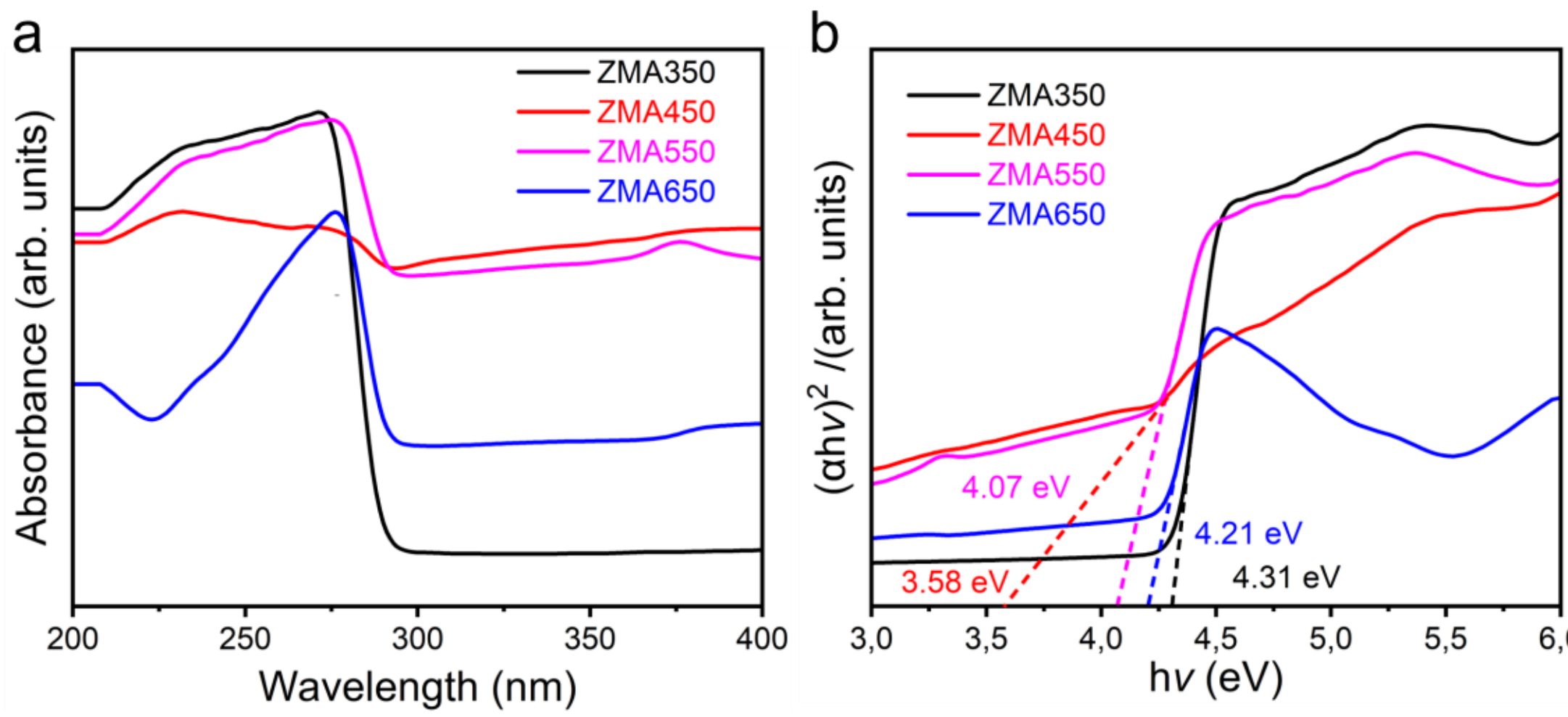

Supplementary Fig. 19 | UV–visible absorption spectra and optical band-gap analysis of ZMA350, ZMA450, ZMA550, ZMA650. **a**, UV–visible absorption spectra, **b**, Corresponding Tauc plots of $(\alpha h\nu)^2$ versus photon energy hν used to estimate the direct optical band gaps of the samples.

Ultraviolet photoelectron spectroscopy (UPS, He I, $h\nu$ = 21.22 eV) provides two key observables for the ZnO sensing layer:

(i) Work function (WF, Φ) from the secondary-electron cutoff

$$\Phi = h\nu - (E_{cutoff} - E_F) \qquad (4)$$

where E_{cutoff} is the secondary-electron cutoff energy and E_F is the Fermi level (set to 0 eV in the UPS spectra).

(ii) Valence band onset relative to E_F

The valence band onset is determined by linear extrapolation of the leading edge of the valence band:

$$\Delta E_V = E_F - E_{VBM} \qquad (5)$$

where E_{VBM} is the valence band maximum (VBM). In this work, ΔE_V corresponds to the reported VB_{onset} values in Fig. 3f.

From Fig. 3e-f, the measured values are:

- Before H_2S exposure: Φ=4.3 eV; ΔE_V=2.6 eV
- After H_2S exposure: Φ=3.9 eV; ΔE_V=3.0 eV

These two UPS observables together constrain both the vacuum-level position (via Φ) and the band-edge position relative to E_F (via ΔE_V).

Band-edge positions and the "effective barrier" on the ZnO side:

For an n-type semiconductor, the energy separation between the conduction band minimum (CBM) and the Fermi level at/near the surface is:

$$\Delta E_C = E_{CBM} - E_F \qquad (6)$$

Using the optical bandgap of ZnO (taken as E_g=4.07 in this work), and the UPS-derived Formula (5), we obtain:

$$E_{CBM} - E_F = E_g - (E_F - E_{VBM}) = E_g - \Delta E_V \qquad (7)$$

Before H_2S exposure:

$$\Delta E_C^{before} = 4.07 - 2.6 = 1.47 \text{ eV} \qquad (8)$$

After H_2S exposure:

$$\Delta E_C^{after} = 4.07 - 3.0 = 1.07 \text{ eV} \qquad (9)$$

Thus, the surface/near-interface electron barrier on the ZnO side decreases by:

$$\Delta(\Delta E_C) = 1.47 - 1.07 = 0.4 \text{ eV} \qquad (10)$$

Formula (6) is a direct energetic descriptor for electron depletion at the ZnO surface/near-interface region: a smaller ΔE_C implies a less depleted (more electron-rich) near-surface region, a narrower depletion width, and a reduced energetic penalty for interfacial electron transport. Under operating conditions of a metal/n-ZnO junction, this reduction manifests as an effective lowering of the interfacial transport barrier, consistent with the observed conductance increase upon H_2S exposure. The reported 0.4 eV barrier reduction is extracted from the band-edge alignment change relative to E_F (via ΔE_V and E_g), which is from the ZnO-side depletion/transport barrier, rather than relying solely on an ideal Schottky-Mott absolute barrier value.

Assessment of alternative explanations:

(i) Interface states

To assess whether new interfacial electronic states dominate the energetics shift, the valence-band spectra were inspected on a semi-log scale (Supplementary Fig. 20 and Table 2). No pronounced additional in-gap tailing or emergent valence-band components beyond those attributable to ZnO and Au are observed. This argues against strong interface-state formation as the primary origin of the barrier modulation inferred from ΔE_V. However, we acknowledge that interface states may still play a minor role in the overall energetics shift.

(ii) Surface dipoles

Surface dipoles can shift the vacuum level and therefore modify the work function (Φ). However, a pure dipole-driven vacuum-level shift would not necessarily produce a systematic change in Formula (5). In contrast, in this work ΔE_V changes by 0.4 eV (2.6→3.0 eV), which directly alters Formula (6) and yields the extracted 0.4 eV effective barrier reduction. Therefore, while dipoles may contribute to the work function shift, the band-edge alignment change relative to E_F indicates that charge redistribution and band-bending modulation accompany any vacuum-level effect and directly impact the effective transport barrier under sensing conditions. While UPS does not provide the microscopic origin of the vacuum level shift, it does give absolute energetic positions, supporting the observed shift in barrier modulation.

(iii) Defect redistribution

Defect-related surface chemistry may influence the magnitude of the UPS shifts. Nevertheless, the key quantity used to quantify the effective barrier modulation here is Formula (7), derived

from the band-edge position relative to E_F. The concerted, systematic shifts in UPS observables before/after gas exposure support a barrier-modulation scenario consistent with the device response, rather than requiring a dominant irreversible defect redistribution mechanism. Any defect-chemistry contribution would need to produce comparable and systematic shifts in both work function and band-edge positions, which is not observed in the current system.

In addition, the Schottky barrier height is not directly measured by UPS, but derived from the UPS-extracted work function and valence band onset. The work function was obtained from the secondary electron cutoff using linear extrapolation of the cutoff edge to the baseline, and the valence band onset was determined by linear extrapolation of the leading edge of the valence band spectrum. Because both parameters rely on edge extrapolation and energy referencing, small session-to-session variations for ZMA550 (WF: 3.9 → 3.8 eV; VB onset: 3.0 → 3.1 eV) can arise from fitting-window selection and surface sensitivity. Consequently, minor differences in the derived barrier height may originate from the extraction procedure, while the relative trend across the sample series remains unaffected.

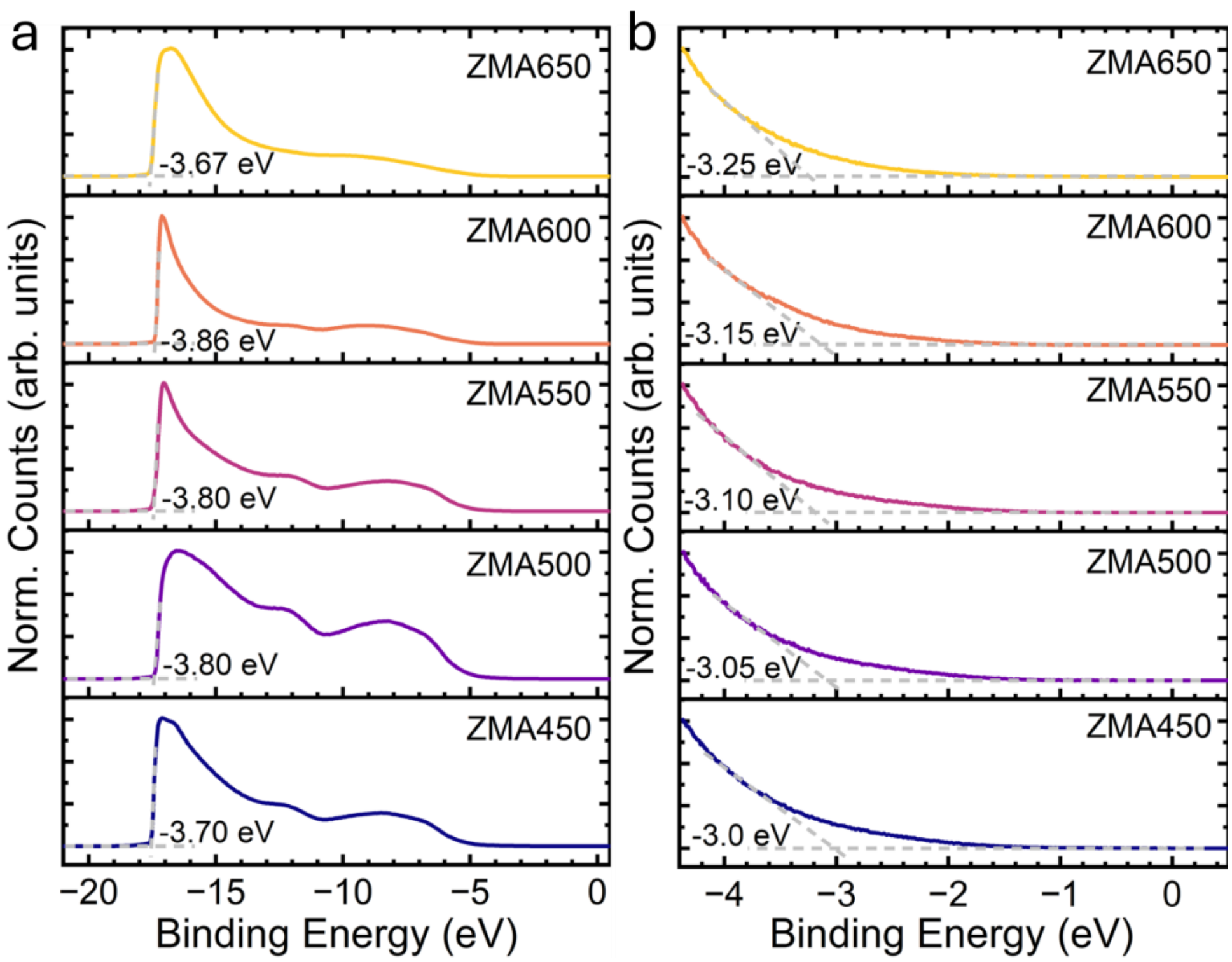

Supplementary Fig. 20 | a, UPS spectra in the secondary electron cutoff region for work-function determination (by linear extrapolation of the cutoff edge). **b,** UPS valence-band spectra showing the valence-band onset for samples pyrolyzed at 450-650 °C after H_2S sensing tests.

Supplementary Table 2 | Comparison of barrier parameters for samples pyrolyzed at 450-650 °C after H_2S sensing tests.

Samples	φ_{Au} (eV)	φ_{ZnO} (eV)	VB_{onset} (eV)	Barrier (eV)
ZMA 450		3.70	3.00	1.07
ZMA 500		3.80	3.05	1.02
ZMA 550	4.7	3.80	3.10	0.97
ZMA 600		3.86	3.15	0.92
ZMA 650		3.67	3.25	0.82

A direct correlation among grain size, GB density, and Schottky barrier height across samples prepared at different pyrolysis temperatures (Supplementary Fig. 21). The EBSD analysis was employed to quantify the grain size and grain-boundary density of ZnO sensing layers pyrolyzed at 450–650 °C. Increasing pyrolysis temperature induces progressive grain growth accompanied by a monotonic reduction in GB density (Supplementary Fig. 22 and Table 3). Samples pyrolyzed at ≤450 °C were excluded from quantitative EBSD analysis due to insufficient spatial resolution and indexing reliability (Supplementary Fig. 22).

UPS measurements (Supplementary Fig. 20) performed on the same set of samples reveal a systematic decrease in the Schottky barrier height derived from the work function and valence band onset with decreasing GB density (Supplementary Table 3). By correlating EBSD-derived microstructural parameters with UPS-derived electronic structure metrics, we establish a quantitative structure–barrier relationship across the temperature series.

It should be noted that UPS-derived barrier values reflect the interfacial electronic structure under pristine surface conditions and are used here to establish inter-sample trends. In contrast, the barrier modulation discussed in the sensing operation corresponds to the effective operating-state barrier of the Au–ZnO junction under gas exposure. While absolute barrier values under these conditions are not expected to coincide, they provide complementary insights into the structure–property relationship governing the sensing mechanism.

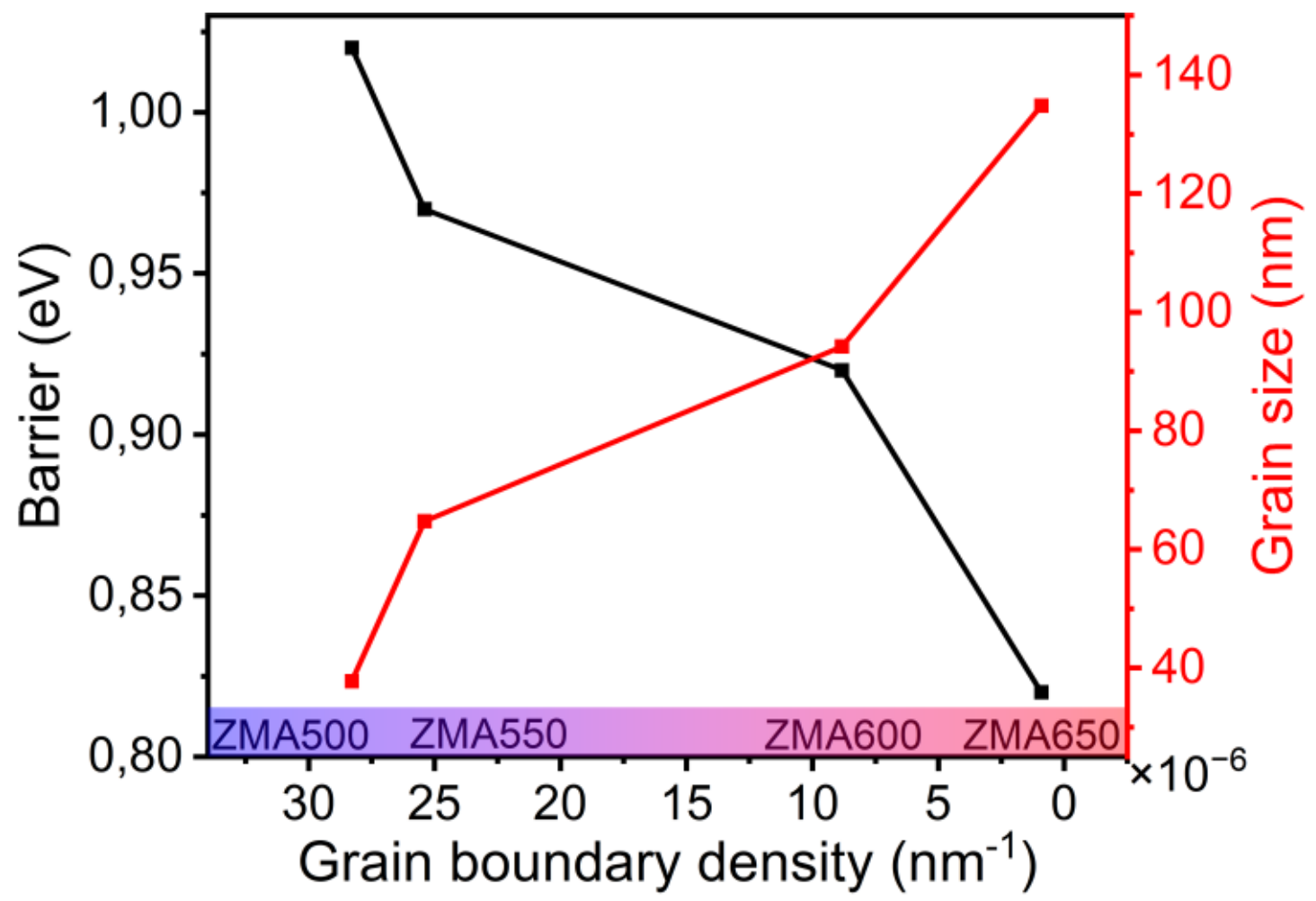

Supplementary Fig. 21 | Grain size and grain boundary density extracted from EBSD, and Schottky barrier height derived from UPS for samples pyrolyzed at 500–650 °C.

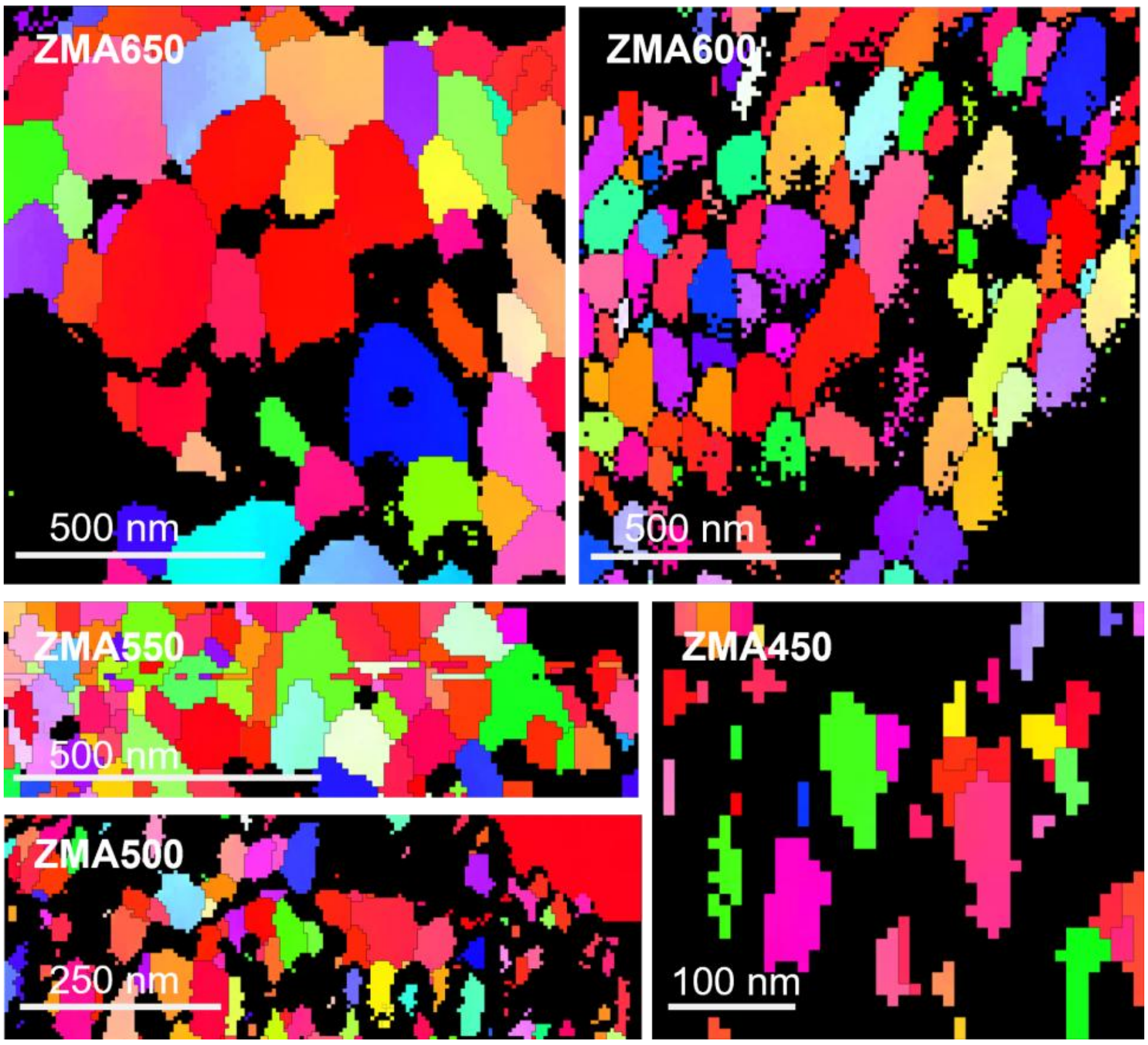

Supplementary Fig. 22 | EBSD images of samples pyrolyzed at 450–650 °C.

Supplementary Table 3 | Grain boundary concentration and density of samples pyrolyzed at 500–650 °C.

Samples	Total length of GB/nm	Area /nm^2	GB density/nm^{-1}
ZMA 500	6.08148	215024.9500	2.82827×10^{-5}
ZMA 550	10.14060	399185.0975	2.54033×10^{-5}
ZMA 600	11.96009	1355545.3960	8.82308×10^{-6}
ZMA 650	11.50177	12590887.6200	9.13500×10^{-7}

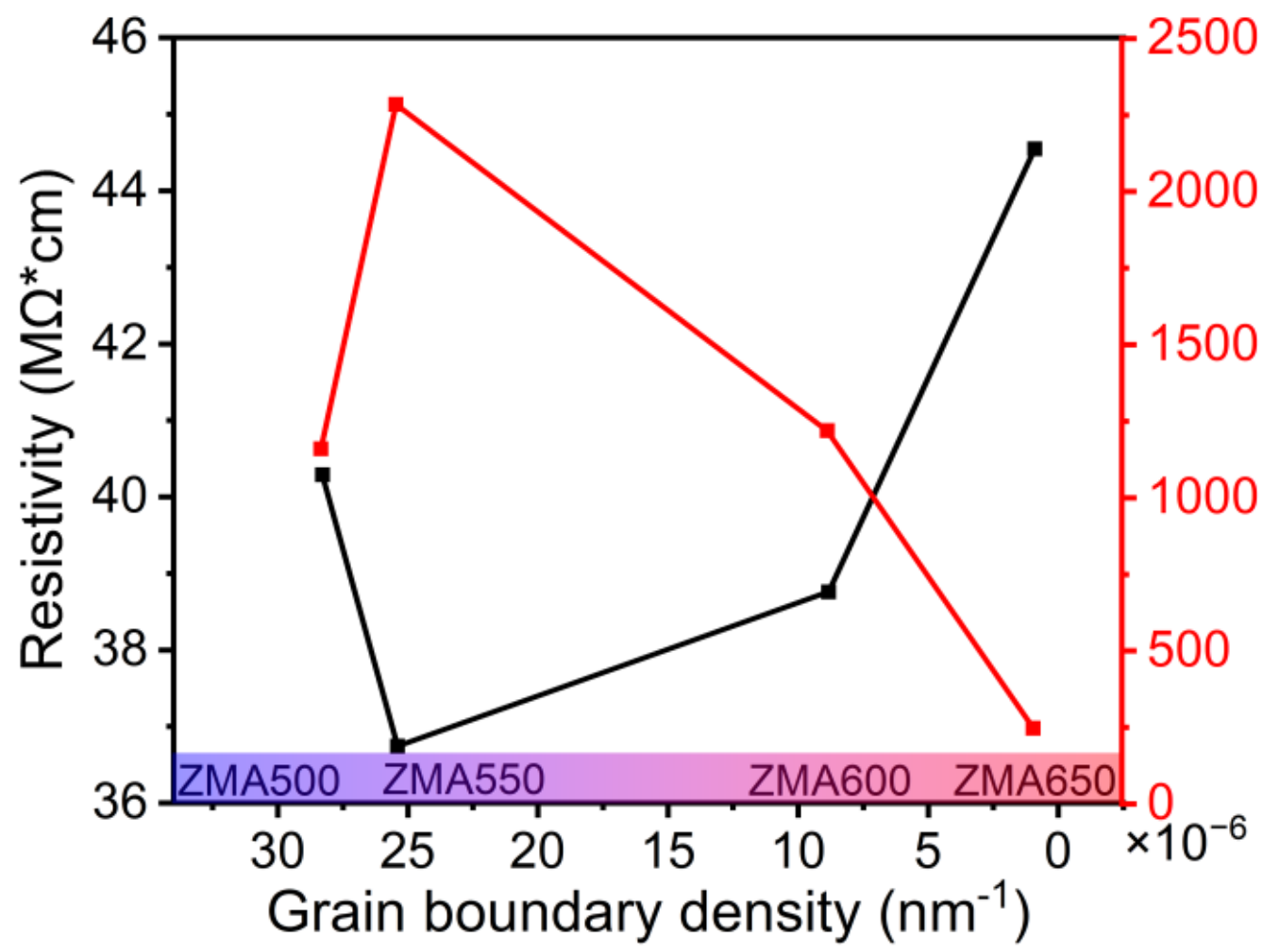

Supplementary Fig. 23 | Correlation between grain-boundary density, electrical resistivity, and H_2S sensing response.

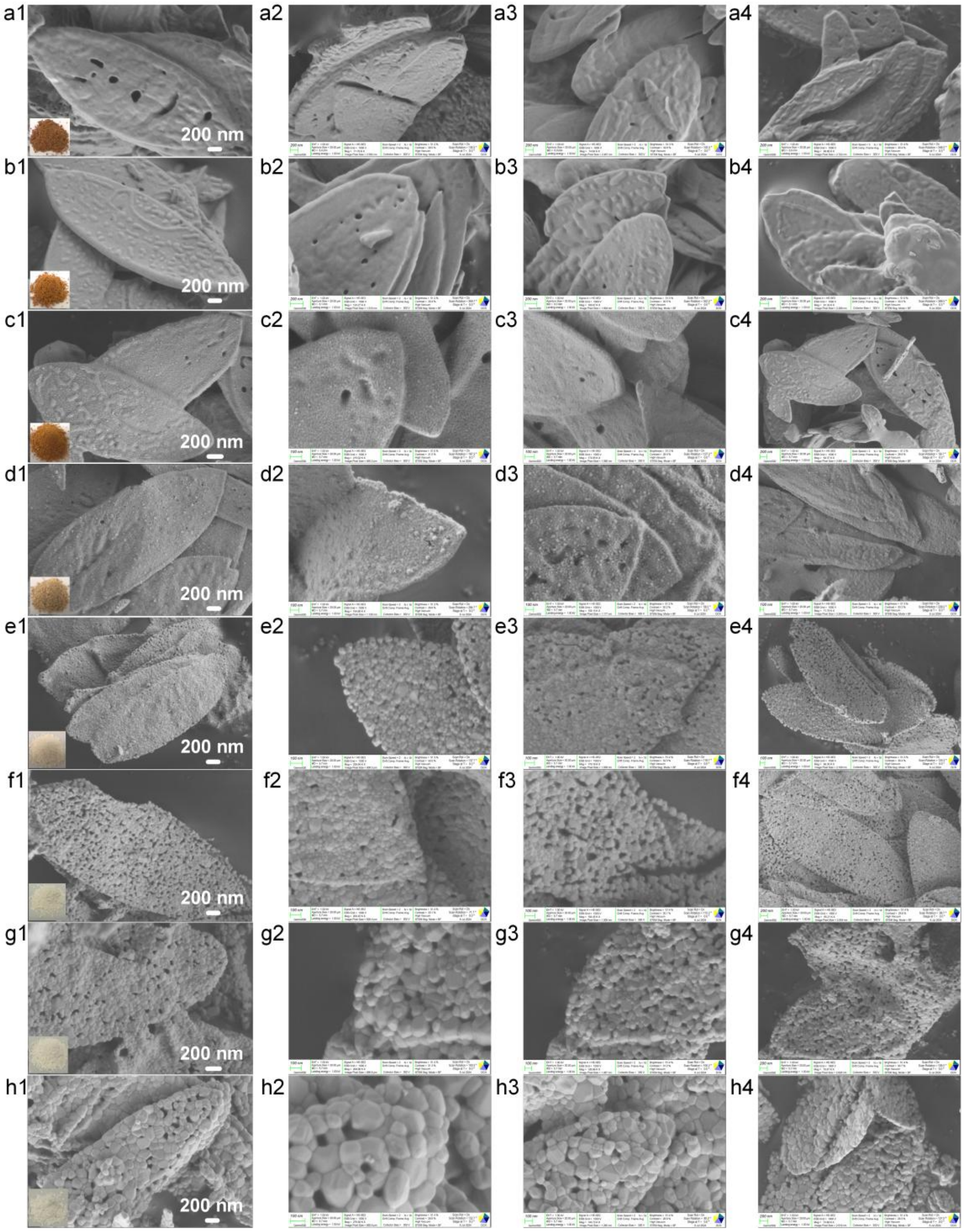

Supplementary Fig. 24 | SEM images of samples synthesized at various temperatures: **a,** ZMA300; **b,** ZMA350; **c,** ZMA400; **d,** ZMA450; **e,** ZMA500; **f,** ZMA550; **g,** ZMA600; **h,** ZMA650.

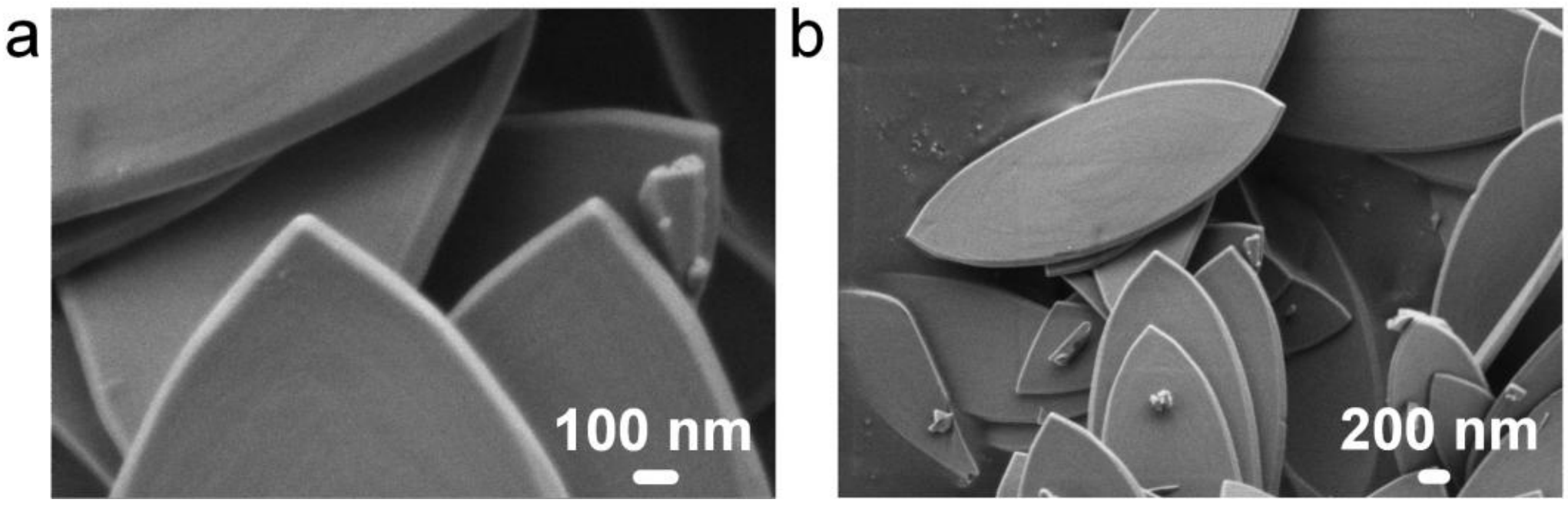

Supplementary Fig. 25 | a-b, SEM images of the Zn MOF precursor.

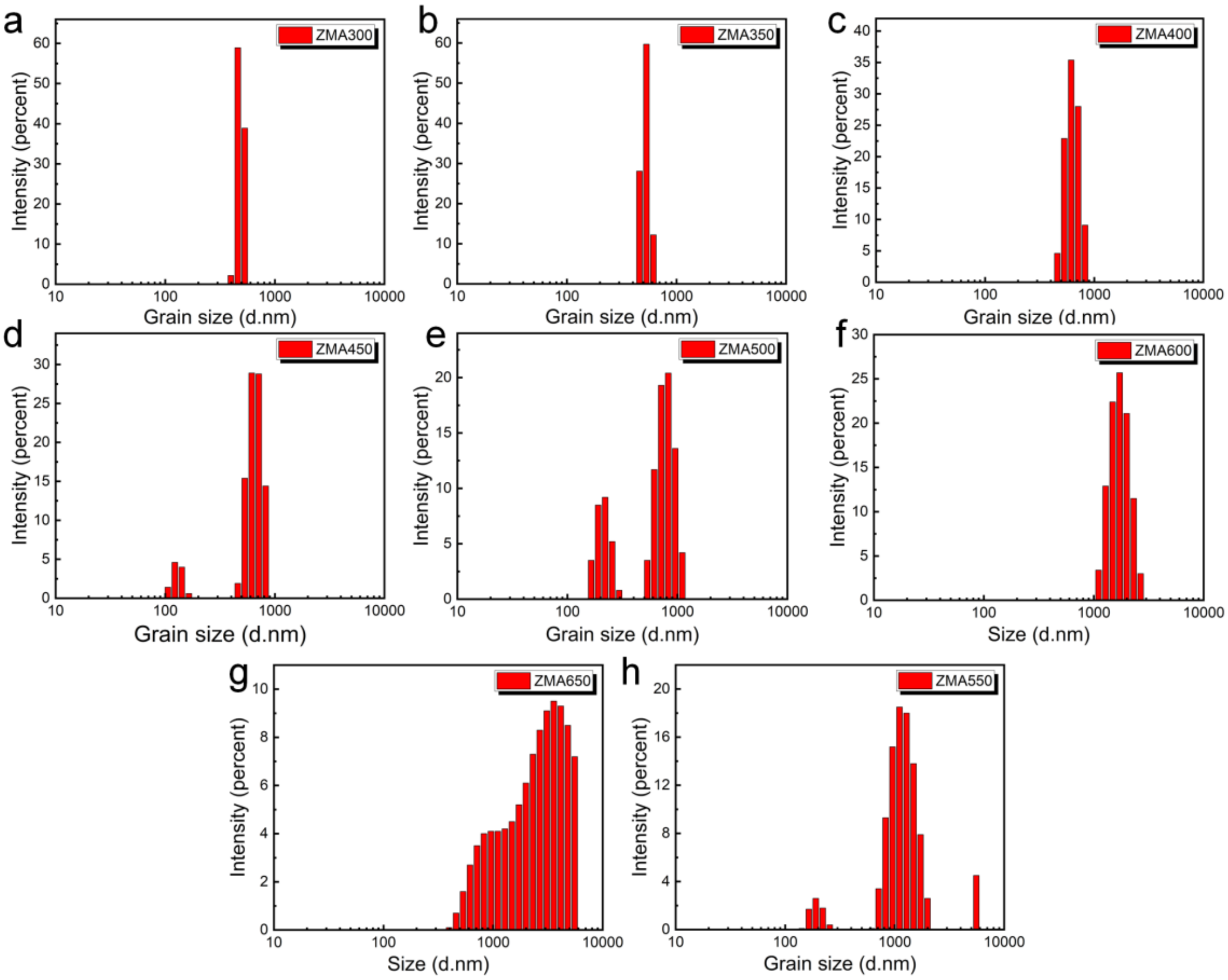

Supplementary Fig. 26 | Grain size distribution: **a,** ZMA300, **b,** ZMA350, **c,** ZMA400, **d,** ZMA450, **e,** ZMA500, **f,** ZMA550, **g,** ZMA600, and **h,** ZMA650.

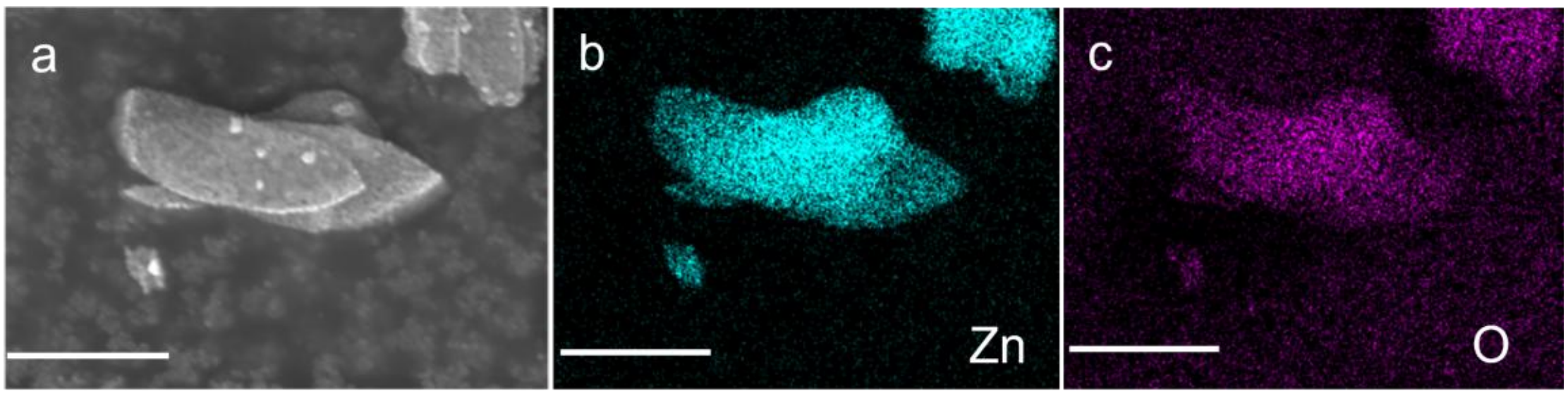

Supplementary Fig. 27 | Elemental mapping of ZnO derived from spindle-like Zn MOF: **a,** SEM image, **b,** Zn element, **c,** O element. The scale bar in corresponds to 2.5 μm.

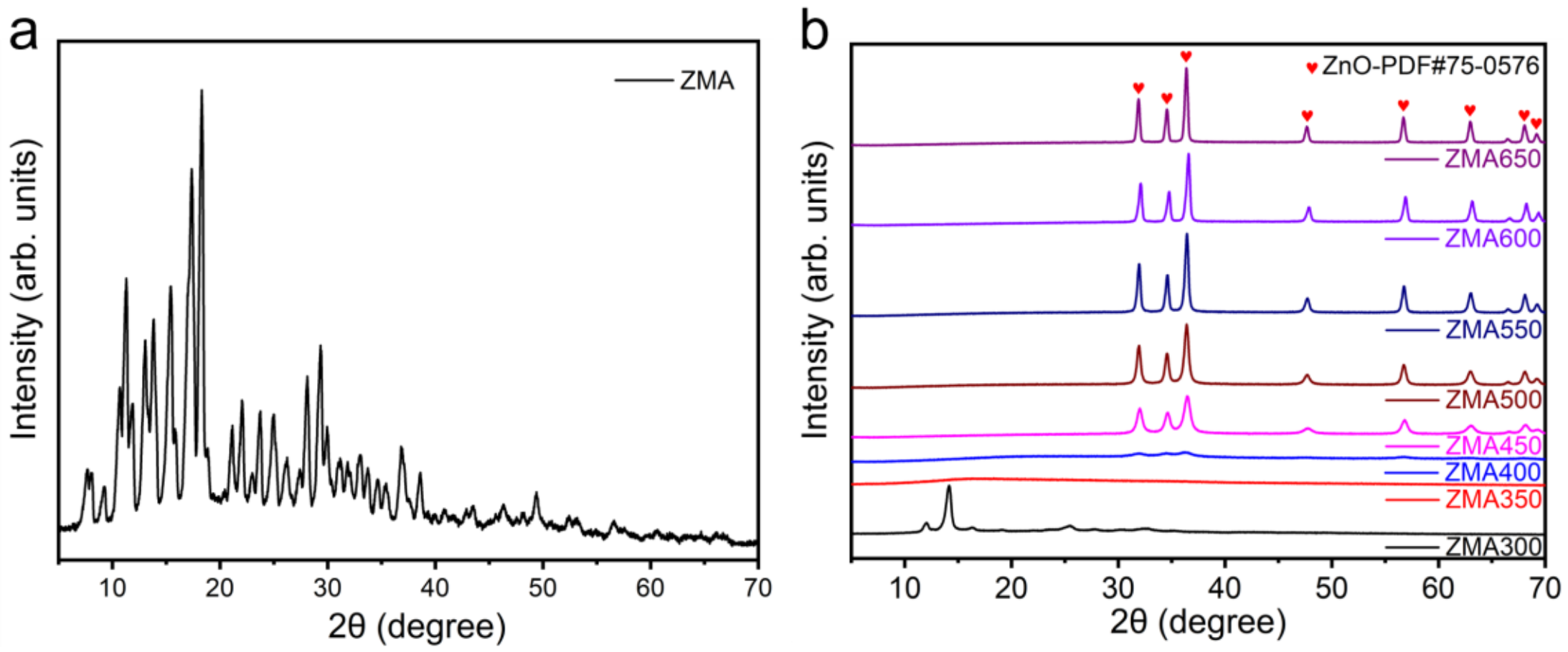

Supplementary Fig. 28 | XRD patterns: **a,** Zn MOF precursor (ZMA) and **b,** all thermally treated samples.

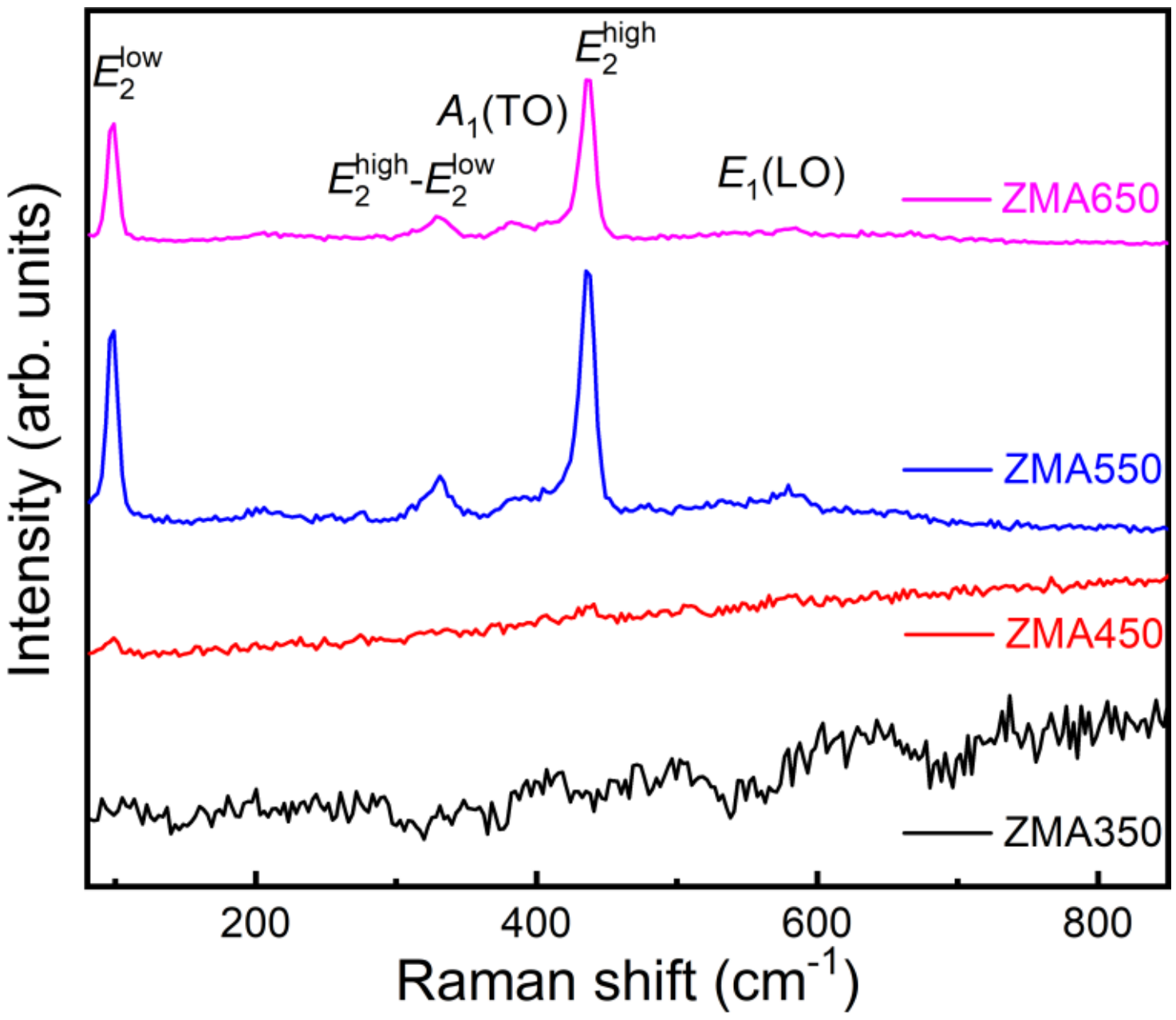

Supplementary Fig. 29 | Raman spectra of ZMA350, ZMA450, ZMA550, ZMA650.

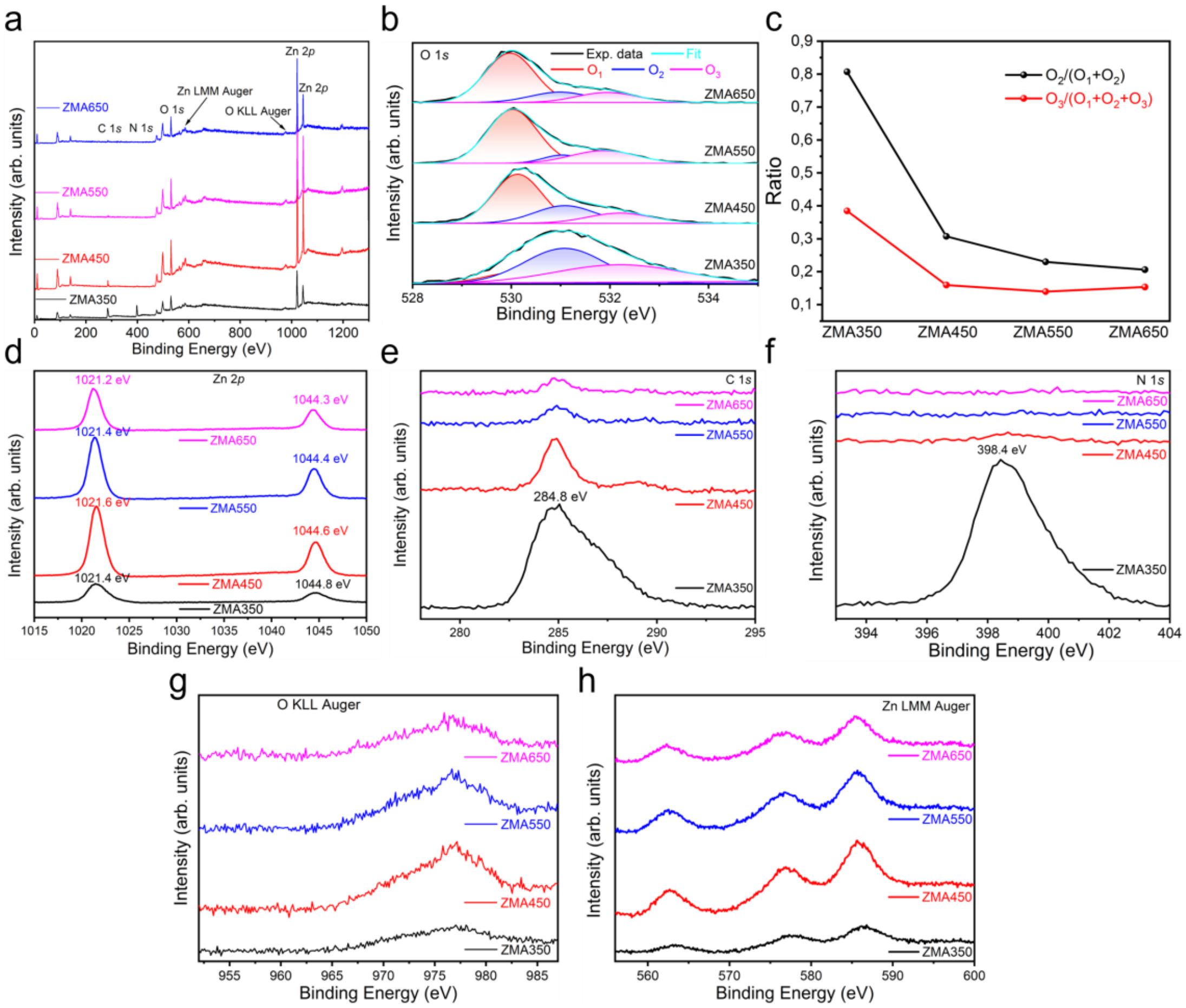

Supplementary Fig. 30 | XPS analysis of ZMA350, ZMA450, ZMA550, ZMA650: **a,** XPS survey, **b,** O 1*s* spectrum, **c,** A ratio comparison of O_1, O_2, and O_3 species, **d,** Zn 2*p* spectrum, **e,** C 1*s* spectrum, **f,** N 1*s* spectrum, **g,** O KLL auger spectrum, and **h,** Zn LMM auger spectrum.

Supplementary Table 4 | Atomic concentration of ZMA350, ZMA450, ZMA550, ZMA650 based on XPS.

Samples#	Elements Content (at%)			
	C	N	O	Zn
ZMA350	46.12	22.62	18.38	12.89
ZMA450	12.78	1.99	41.71	43.52
ZMA550	4.65	0.00	44.78	50.57
ZMA650	3.78	0.00	48.42	47.80

Nitrogen adsorption-desorption measurements were performed to investigate the structural and surface modifications induced by phase transitions of MOF-derived materials at different temperatures. Supplementary Fig. 31a displays the N_2 adsorption-desorption isotherms of as-prepared samples within a relative pressure range of 0.0-1.0. The ZMA450 sample exhibits the largest hysteresis loop area, indicating superior N_2 adsorption-desorption capacity, which correlates with its highest BET surface area (27 m^2/g) among the investigated samples. In contrast, samples prepared at lower (350 °C) and higher (650 °C) temperatures demonstrate significantly reduced N_2 adsorption-desorption capacities, corresponding to minimal BET surface areas and pore volumes (Supplementary Fig. 31b). Pore size distribution analysis is shown in Supplementary Fig. 31c. Notably, despite its highest BET surface area, ZMA450 exhibits a relatively narrow and monomodal pore-size distribution centered at ~20 nm, which may limit effective gas diffusion and active-site accessibility under dynamic sensing conditions. In contrast, ZMA550 features a bimodal mesoporous structure with larger characteristic pore sizes (~40 and 70 nm), which is more favorable for rapid gas transport and efficient utilization of surface active sites. These structural differences help rationalize why ZMA550 outperforms ZMA450 in sensing performance despite a lower overall BET surface area. ZMA350 and ZMA650 show negligible well-defined porosity, indicating collapsed pore structures.

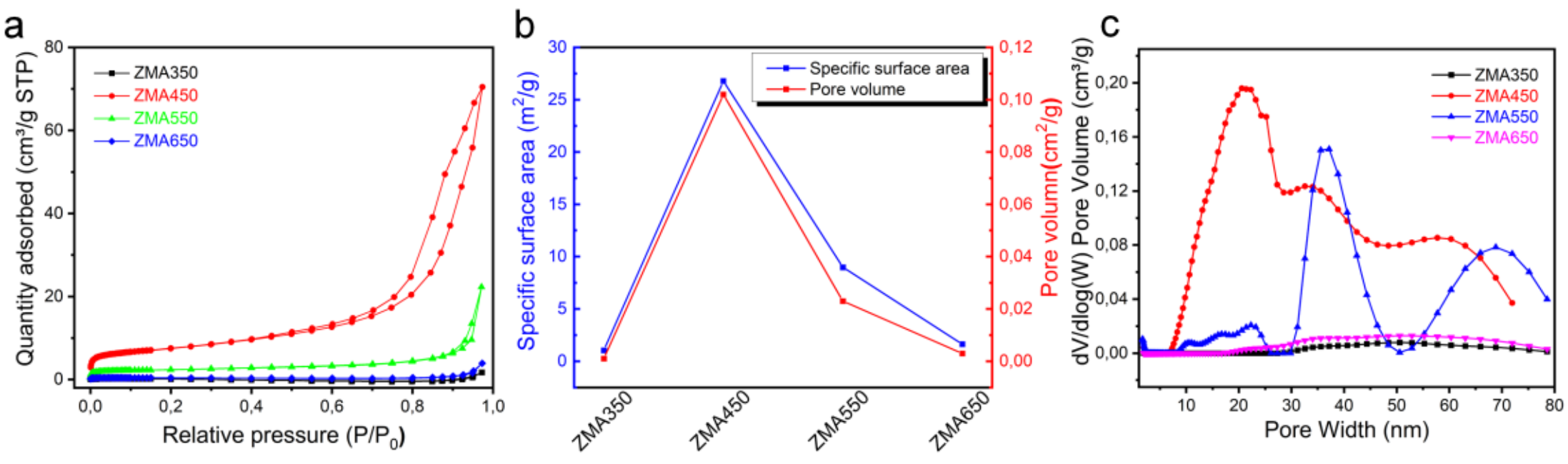

Supplementary Fig. 31 | a, N_2 adsorption–desorption isotherms. **b,** BET specific surface area and pore volume. **c,** pore size distributions calculated using DFT models for ZMA350, ZMA450, ZMA550, and ZMA650.

In situ Fourier-transform infrared (FTIR) spectroscopy was employed to probe the dynamic surface chemical evolution of ZnO during H_2S exposure, aiming to bridge static electronic structure characterization with real-time sensing behavior.

During H_2S exposure, no pronounced S–H stretching vibration was observed in the 2550–2600 cm^{-1} region. This observation is consistent with previous studies reporting that H_2S preferentially undergoes dissociative adsorption on ZnO surfaces, forming strongly coordinated sulfur-containing species (e.g., HS or S) bound to surface Zn sites. The characteristic vibrational modes of such Zn–S(H)-related intermediates are typically located in the low-wavenumber region (<500 cm^{-1}), rendering the S–H stretching mode weak or undetectable in the present measurements. Therefore, the absence of an S–H band does not preclude the formation of Zn–S(H)-type surface intermediates.

Several surface-related vibrational features exhibit pronounced and reversible intensity changes during H_2S on/off cycles (Supplementary Fig. 32 right). Specifically, the band at ~3680 cm^{-1}, assigned to terminal or weakly hydrogen-bonded Zn-OH groups, together with hydrogen-bond-associated bands near ~3000 cm^{-1}, shows reversible attenuation and recovery upon H_2S exposure and removal. In addition, paired bands at ~1400 and ~890 cm^{-1}, characteristic of surface carbonate/bicarbonate species, display correlated reversible variations [41-44]. These observations indicate that H_2S adsorption dynamically perturbs surface hydroxyl groups, acid-base sites, and adsorption equilibria on ZnO, rather than inducing permanent chemical transformation. The reversibility of these spectral changes strongly suggests that the sensing process is governed by surface coordination and adsorption-desorption dynamics instead of irreversible sulfidation.

In the low-wavenumber region, a broad absorption band spanning 400-600 cm^{-1} with an inflection near ~520 cm^{-1} is assigned to Zn-O lattice vibrations. A reversible band at ~1060 cm^{-1} is observed upon H_2S exposure/removal, which is attributed to S-O stretching of surface Zn-SO_x species formed during the sensing process (Supplementary Fig. 32 left) [41-42, 45]. The modulation of these features during H_2S exposure reflects surface-sensitive perturbations of the Zn-O framework associated with adsorption-induced electronic and structural rearrangements. Importantly, post-sensing TEM, EDX, XPS, and PXRD analyses do not reveal the formation

of ZnS secondary phases (Supplementary Fig. 13), confirming that the interaction between H_2S and ZnO is confined to reversible surface coordination rather than bulk-phase sulfidation.

Taken together, the in situ FTIR results provide direct evidence that H_2S adsorption induces reversible surface chemical and coordination changes on ZnO, dynamically modulating surface hydroxyls, acid–base equilibria, and Zn–O-related lattice environments. These surface processes are inherently coupled to electronic structure reconfiguration, thereby providing dynamic experimental support for the barrier-tunable sensing mechanism inferred from static electronic structure analysis.

Density functional theory (DFT) calculations were carried out to rationalize the energetics and electronic structure perturbations associated with H_2S-derived surface intermediates inferred from the in situ FTIR observations.

Representative adsorption configurations of H_2S and its dissociation products (HS and S) on the ZnO surface were considered. The calculated adsorption energies indicate that dissociative adsorption configurations involving Zn–S(H)-type coordination are energetically more favorable than molecularly adsorbed H_2S, consistent with the absence of a detectable S–H stretching vibration in the in situ FTIR spectra.

Charge density difference and Bader charge analyses reveal noticeable electron transfer from the adsorbed sulfur-containing species to the ZnO surface, resulting in increased electron density localized near surface Zn sites. This charge redistribution is consistent with the experimentally observed upward shift of the Fermi level and reduction of the interfacial barrier upon H_2S exposure.

The DFT results provide electronic structure and energetic support for the surface intermediates and adsorption configurations inferred from in situ FTIR measurements. Together, these complementary experimental and theoretical results establish a coherent picture in which dissociative H_2S adsorption and reversible Zn–S(H)-type surface coordination dynamically modulate the electronic structure of ZnO, underpinning the observed barrier-tunable sensing behavior.

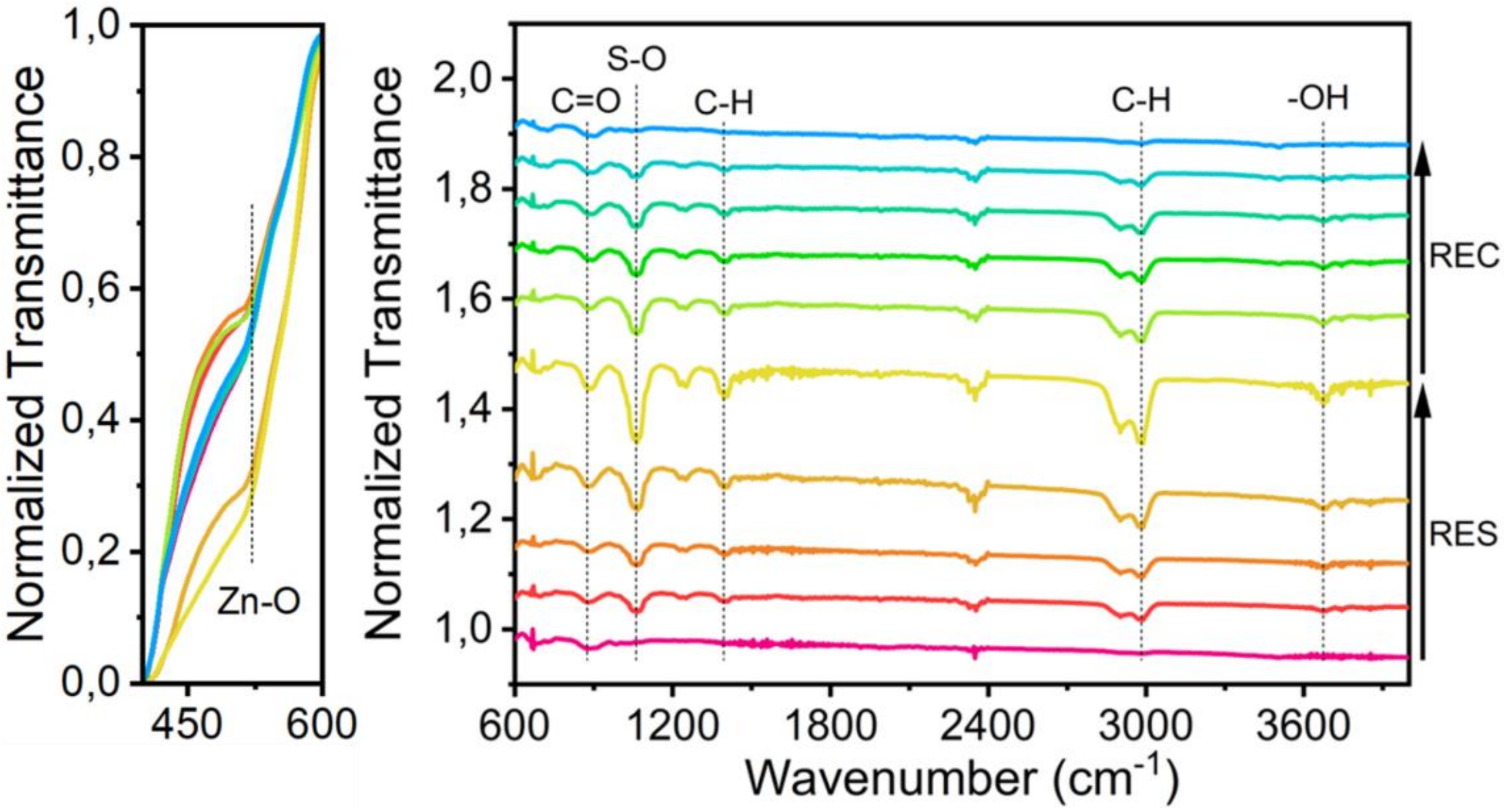

Supplementary Fig. 32 | In situ FTIR spectra of ZMA550 during H_2S adsorption and desorption.

To establish a theoretical foundation for the experimental observations, density functional theory (DFT) simulations were performed to systematically investigate the influence of grain boundary density on sensor performance. Four distinct ZnO configurations were modeled, corresponding to grain boundary densities of 0, 0.5, 1, and 2 (denoted as GB0, GB0.5, GB1, and GB2, respectively) (Supplementary Fig. 33). The simplified model, although not fully representative of the structural complexity of polycrystalline ZnO, captures the key interactions occurring at grain boundaries and vacancy sites. The Figure of Merit (FOM) analysis revealed significant variations in sensing characteristics across different configurations, and comparative evaluation of FOM differences before and after H_2S exposure demonstrated that the GB1 system exhibited minimal FOM variation, indicating optimal Schottky barrier characteristics. These computational findings are in excellent agreement with experimental results, confirming that an intermediate grain boundary density (GB1) maximizes sensing performance toward trace H_2S detection. Both excessive (GB2) and insufficient (GB0, GB0.5) grain boundary densities were found to elevate the Schottky barrier height, thereby impeding electron transport and degrading sensing performance. This non-monotonic relationship between grain boundary density and sensor performance establishes an important design principle for optimizing metal oxide gas sensors. While the model does not explicitly account for all structural variations found in real polycrystalline materials, it provides a physically meaningful and representative description of the dominant mechanisms governing the sensing process. The agreement between the theoretical results and experimental data supports the validity of the simplified model in describing the essential interactions responsible for H_2S sensing.

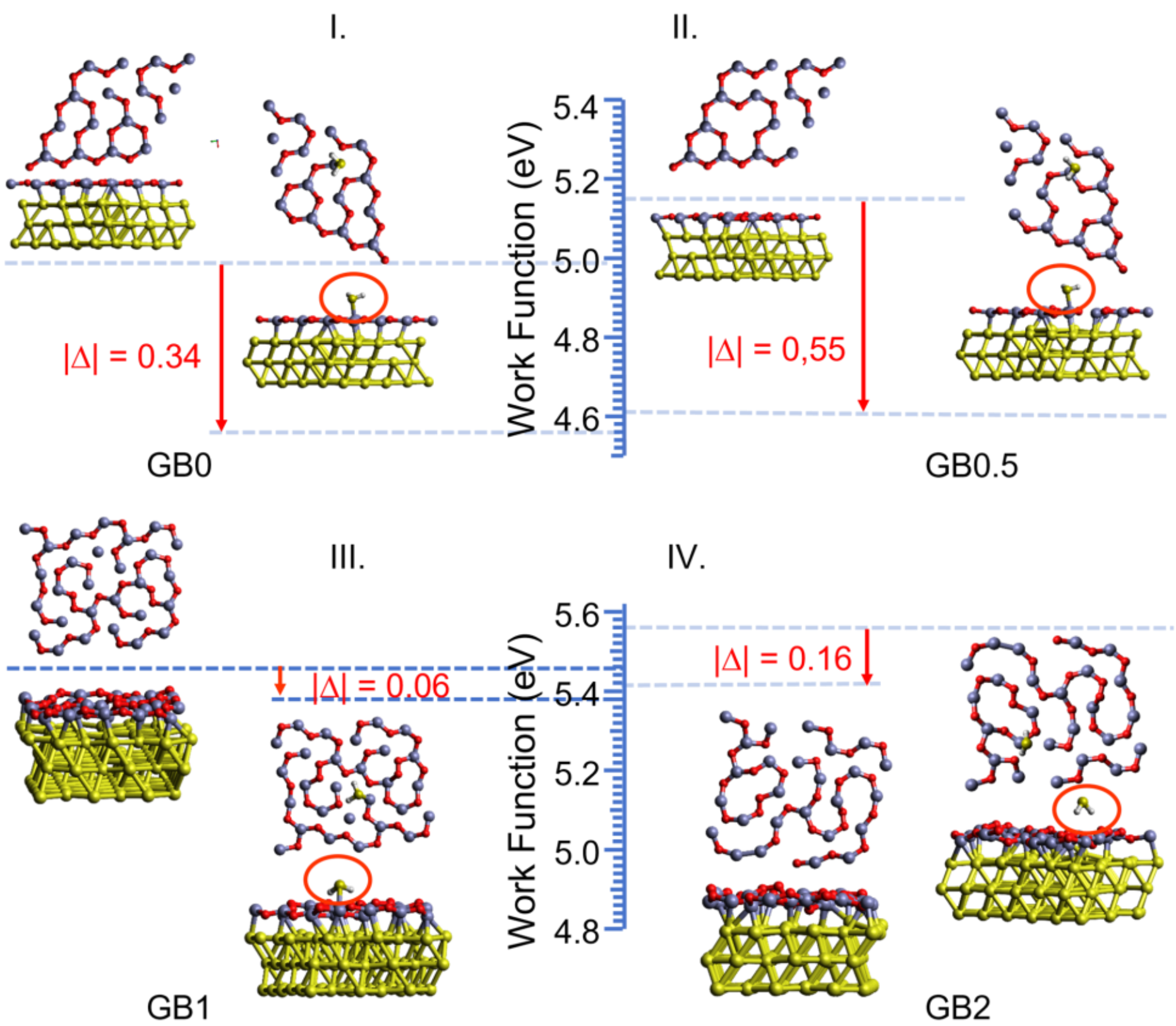

Supplementary Fig. 33 | DFT-simulated models of ZnO with varying grain boundary concentrations (GB0, GB0.5, GB1, GB2) before and after H_2S exposure, and the corresponding variation in calculated work function.

To investigate the influence of oxygen and zinc vacancies on H_2S adsorption and sensor performance, we performed DFT calculations to evaluate the adsorption energies and charge distributions at both vacancy sites (Supplementary Fig. 34). Although the model simplifies the structural complexity of polycrystalline ZnO, focusing on local grain boundary and vacancy sites, it effectively captures the key interactions governing the sensing mechanism. Specifically, we find that H_2S preferentially adsorbs at oxygen vacancy sites, with greater charge transfer to the material compared to zinc vacancies. This is consistent with experimental findings that oxygen vacancies play a more significant role in H_2S adsorption.

The adsorption energies for H_2S at oxygen and zinc vacancy sites are -0.3096 eV and -0.1304 eV, respectively (Supplementary Table 5). The differential charge density analysis (Supplementary Fig. 34c and f) provides further insight into the charge transfer upon adsorption. As shown in the differential charge density, the blue region represents the depletion of charge, while the yellow region indicates the accumulation of charge. Notably, for the oxygen vacancy, a large blue region is observed, indicating a significant amount of charge is transferred from the H_2S molecule to the material surface. This suggests that oxygen vacancies are the primary adsorption sites, where a large amount of charge is accumulated at the interface. In contrast, the zinc vacancy shows a smaller blue region, indicating a lesser degree of charge transfer, further supporting the conclusion that oxygen vacancies play a more prominent role in H_2S adsorption. The results of Bader charge analysis (Supplementary Table 6) confirm that oxygen vacancies contribute more charge to the adsorbed H_2S molecule (-0.1658), compared to zinc vacancies (-0.1370), highlighting the role of oxygen vacancies as the primary adsorption site.

These findings support the experimental observation that ZMA450, despite having a higher oxygen-vacancy concentration, exhibits weaker sensing performance due to its smaller grain size and higher grain-boundary density, which limit charge transport and reduce response efficiency. The grain boundary limitations in ZMA450 hinder its ability to effectively translate surface charge modulation into a macroscopic conductance change, thus impairing its sensing performance.

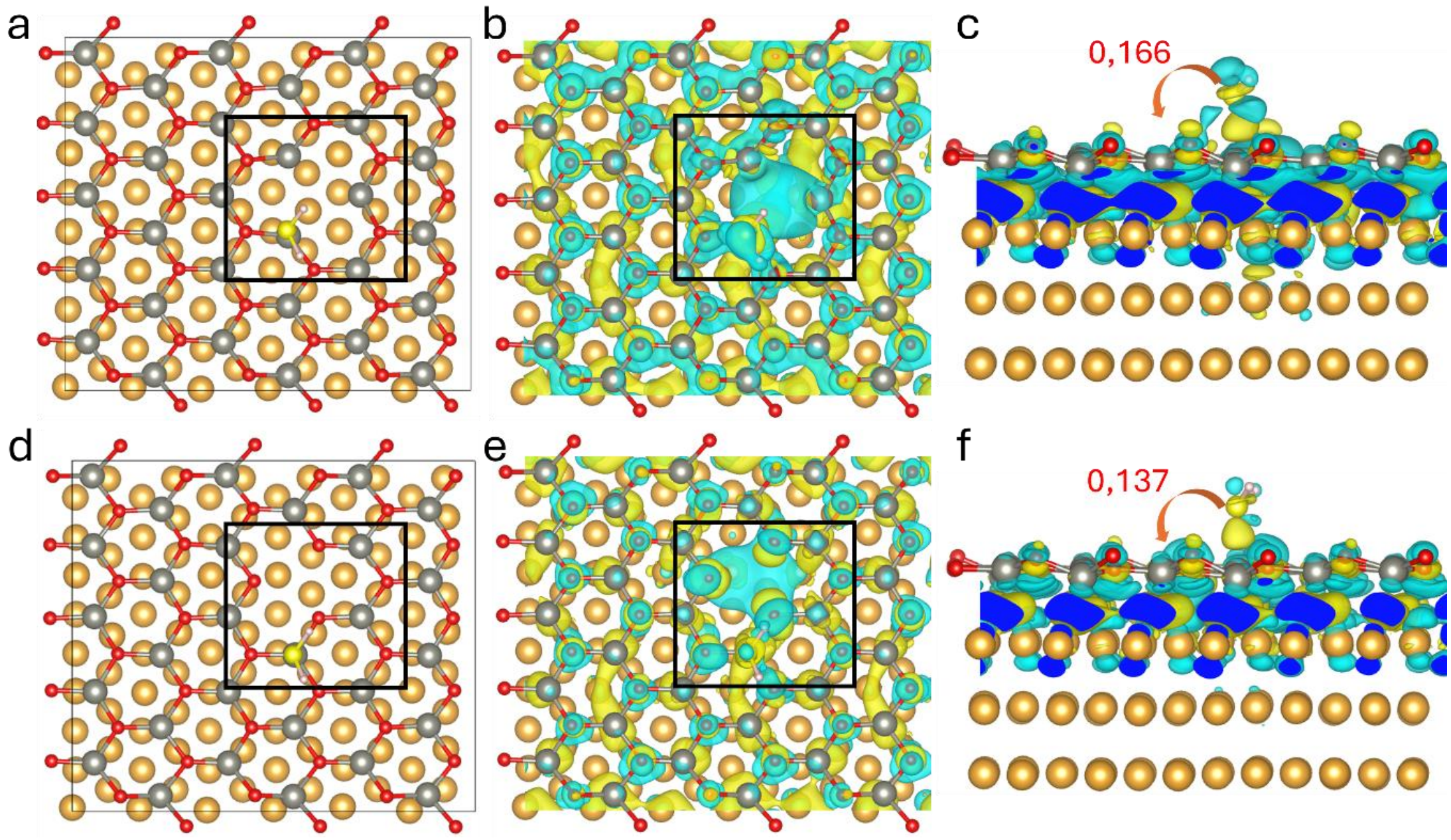

Supplementary Fig. 34 | DFT-based analysis of H_2S adsorption on oxygen and zinc vacancy sites. **a-c,** Oxygen vacancy: adsorption structure, differential charge density, and charge transfer analysis. **d-f,** Zinc vacancy: adsorption structure, differential charge density, and charge transfer analysis.

Supplementary Table 5 | Adsorption energy comparison (E_{bind}) of H_2S on oxygen and zinc vacancy sites.

Defects type	E_{ads}	$E_{surface}$	E_{gas}	E_{bind}
V_O	-649.49783489	-637.99504020	-11.19318950	-0.30960519
V_{Zn}	-650.35992171	-639.03633234	-11.19318950	-0.13039987

Note: $E_{bind}=E_{(surface+gas)}-E_{surface}-E_{gas}$

Supplementary Table 6 | Bader charge transfer for H_2S adsorption at oxygen and zinc vacancy sites.

Species	Q_S	Q_{H1}	Q_{H2}	Q_{sum}	ΔQ
H_2S @ Oxygen Vacancy	5.021889	1.509254	1.303025	7,834168	-0,165833
H_2S @ Zinc Vacancy	5.196020	1.083239	1.034088	7,862959	-0,137042
Pure H_2S	5.882674	1.368198	1.298741	8,000001	/

Note: $\Delta Q_i = Q_i^{adsorbed} - Q_i^{clean}$

EPR was employed as an independent probe to validate defect-related signals beyond XPS deconvolution. All samples exhibit a distinct EPR resonance at g ≈ 2.003-2.004, which is characteristic of defect-related unpaired electrons in ZnO and is commonly associated with oxygen-vacancy-related paramagnetic centers. The g values remain within a narrow range across samples, indicating that the dominant paramagnetic defect type is comparable, whereas the relative defect population is reflected by the signal intensity.

To quantify the temperature-dependent trend, the EPR intensity of the g ≈ 2.003-2.004 feature was evaluated under identical measurement conditions (microwave power, modulation amplitude, receiver gain; intensity normalized by sample mass). The results show that the EPR intensity decreases monotonically with increasing pyrolysis temperature, indicating a progressive reduction of vacancy-related paramagnetic centers at higher temperatures. This behavior is consistent with thermally assisted defect relaxation and enhanced oxygen re-coordination during high-temperature treatment. Together with the microstructure/transport results in the Supplementary Fig. 23, the EPR results support the conclusion that vacancy-related defects contribute to surface activation, whereas peak sensing performance requires an optimal balance between defect density and charge-transport efficiency.

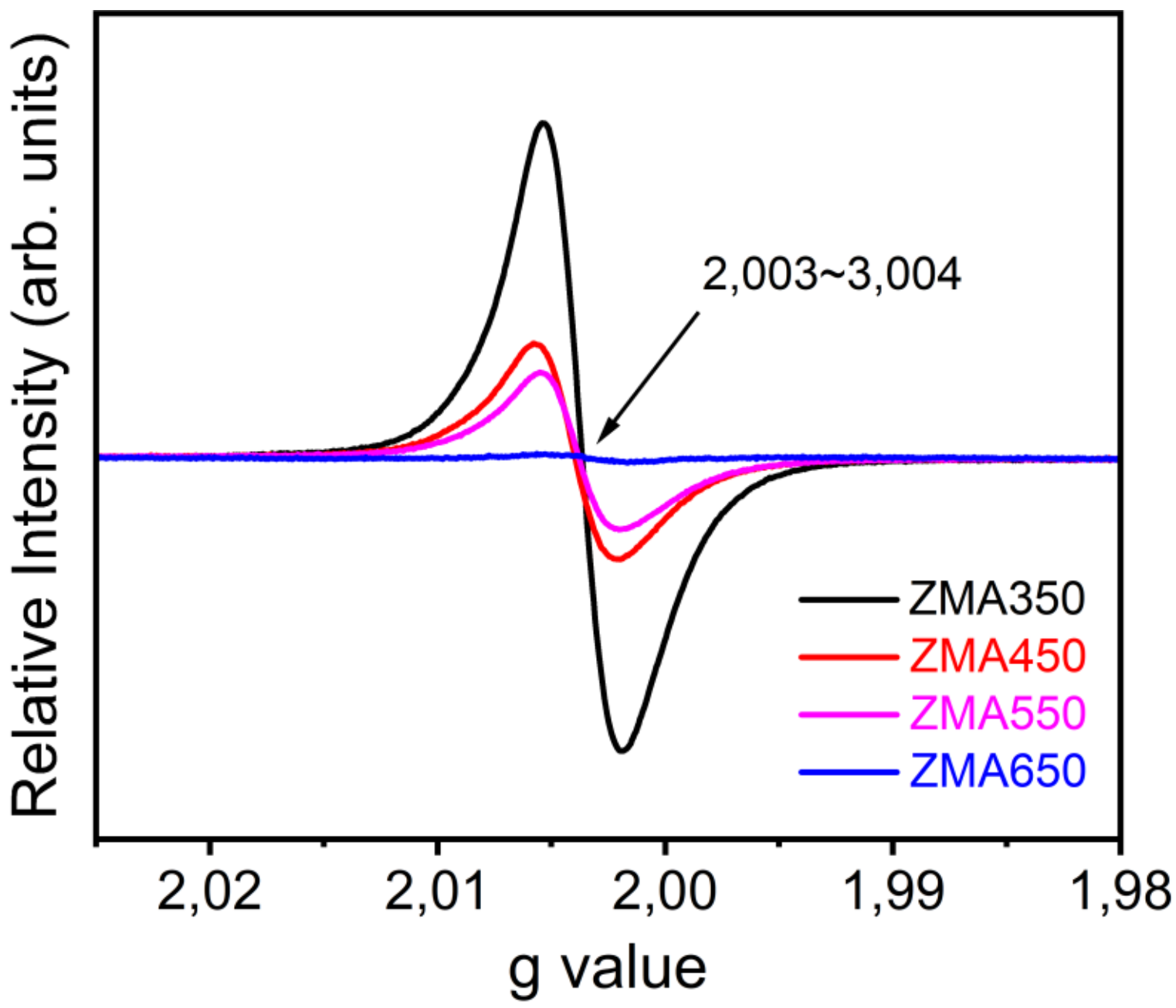

Supplementary Fig. 35 | EPR spectra of ZMA350, ZMA450, ZMA550, ZMA650 samples shown as relative intensity versus g value.

To directly link grain-boundary density to charge transport and sensing performance, we combined impedance characterization, four-probe resistivity measurements, and EBSD-based grain-boundary statistics. Electrochemical impedance spectroscopy (EIS) of the powder-based sensing layers does not show a resolvable semicircle and is dominated by a linear response, indicating that conventional EIS fitting cannot reliably extract intrinsic grain/boundary resistance components in this highly resistive system (Supplementary Fig. 36). We therefore employ four-probe resistivity as a direct transport descriptor, which are widely used as a direct and quantitative descriptor of electrical transport in solids [46-47]. The resistivity exhibits a non-monotonic dependence on pyrolysis temperature, decreasing first and then increasing, with ZMA550 showing the lowest resistivity among all samples (Supplementary Fig. 5).

To correlate microstructure with transport and sensing, EBSD statistics were used to estimate grain size and grain-boundary density. For samples with extremely fine grains (notably ZMA450 and other low-temperature samples), the EBSD-based boundary quantification shows large uncertainty and is thus not included in the quantitative correlation plot. Accordingly, the correlation analysis focuses on ZMA500, ZMA550, ZMA600, and ZMA650, where EBSD statistics are reliable (Supplementary Fig. 22-23 and Table 3). Across these samples, increasing pyrolysis temperature results in grain growth and reduced grain-boundary density, accompanied by a non-monotonic evolution of resistivity (minimum at ZMA550) and a corresponding non-monotonic sensing response (maximum at ZMA550), highlighting an optimal grain-boundary density window that balances charge transport and sensing transduction (Supplementary Fig. 23).

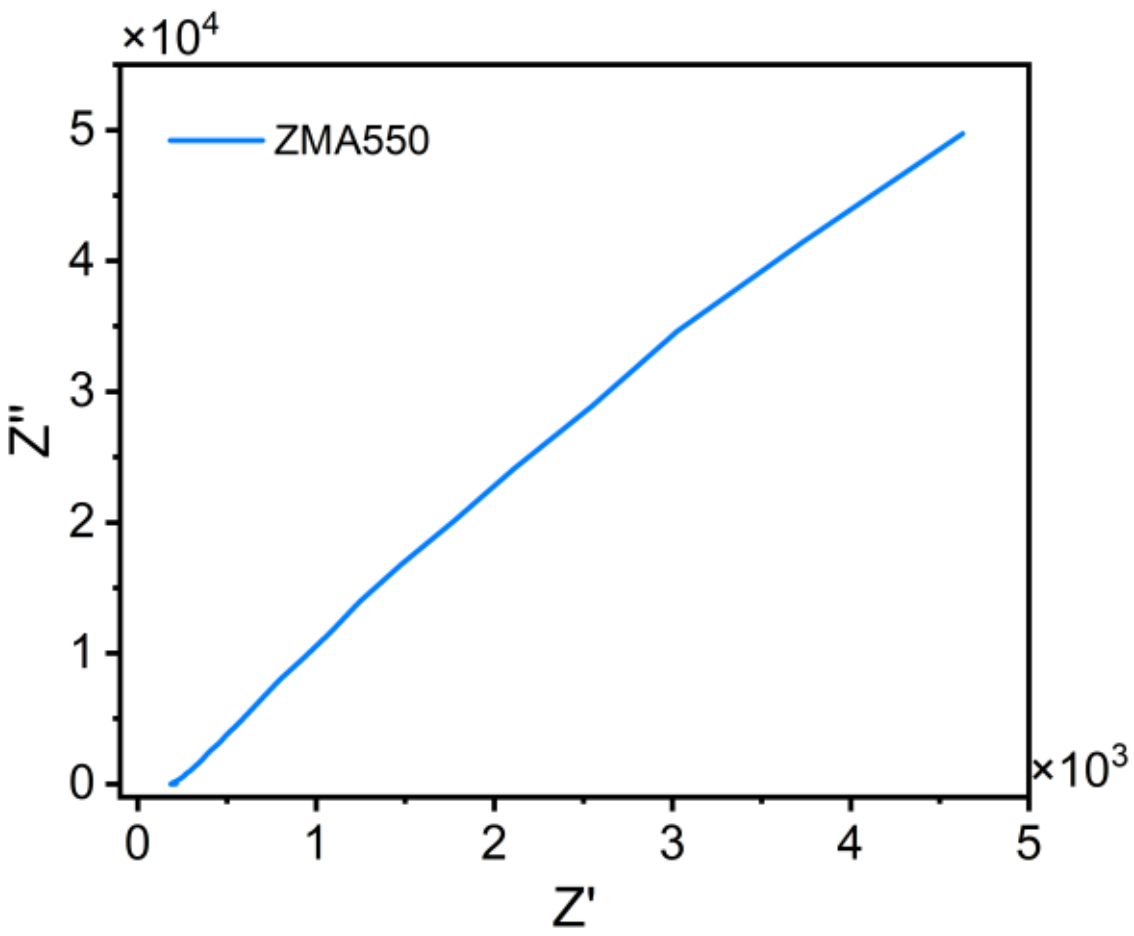

Supplementary Fig. 36 | Electrochemical impedance spectroscopy (EIS) of the ZMA550.

To better simulate practical leak-monitoring conditions, the sensor under several realistic scenarios were evaluated, including (i) long-distance detection (0.50-5.0 m), (ii) airflow and dust disturbances under forced convection, and (iii) high-concentration H_2S robustness (200-1000 ppm) to assess saturation and irreversible damage.

Long-distance detection was examined by positioning the sensor at 0.50 m, 1.0 m, 3.0 m, and 5.0 m from the leak outlet under still-air conditions (fan off), while keeping all other operating parameters unchanged. As expected, the response decreased with distance, but a noticeable response was still observed at 5.0 m, demonstrating the sensor's ability to detect leaks over meter-scale distances (Supplementary Fig. 37a-d).

To simulate industrial airflow perturbations, we fixed the sensor-to-leak distance at 1.0 m and four sequential conditions were applied: (i) still-air baseline; (ii) weak forced convection generated by a miniature DC fan powered at 8 V; (iii) weak convection with dust challenge, implemented by placing 20 mg of dried, sieved soil powder at the fan inlet for natural entrainment (30 s per pulse, two pulses, separated by baseline recovery); and (iv) strong forced convection generated by increasing the fan voltage to 15 V. Airflow disturbances were introduced using an axial fan (diameter ~10 cm), placed between the sensor and the gas source, operating at two voltage levels (8 V and 15 V) to simulate weak and strong convection. Specifically, at 8 V, the fan generates a weak convection velocity of approximately $v_{\text{weak}} = 0.5$ m/s, and at 15 V, a strong convection velocity of $v_{\text{strong}} = 1.2$ m/s, with time averages taken over ≥30 s.

Under weak airflow-only conditions, baseline fluctuations increase and the apparent response amplitude is partially reduced; the strongest airflow produces the largest noise. For dust interference, 20 mg dried, sieved soil powder was placed at the fan inlet and naturally entrained under weak airflow (30 s × 2 pulses). Despite increased noise during dust exposure, the response and recovery features remained identifiable, indicating the sensor’s robustness against combined airflow and dust disturbances (Supplementary Fig. 37e-g), indicating robustness against combined airflow and dust disturbances.

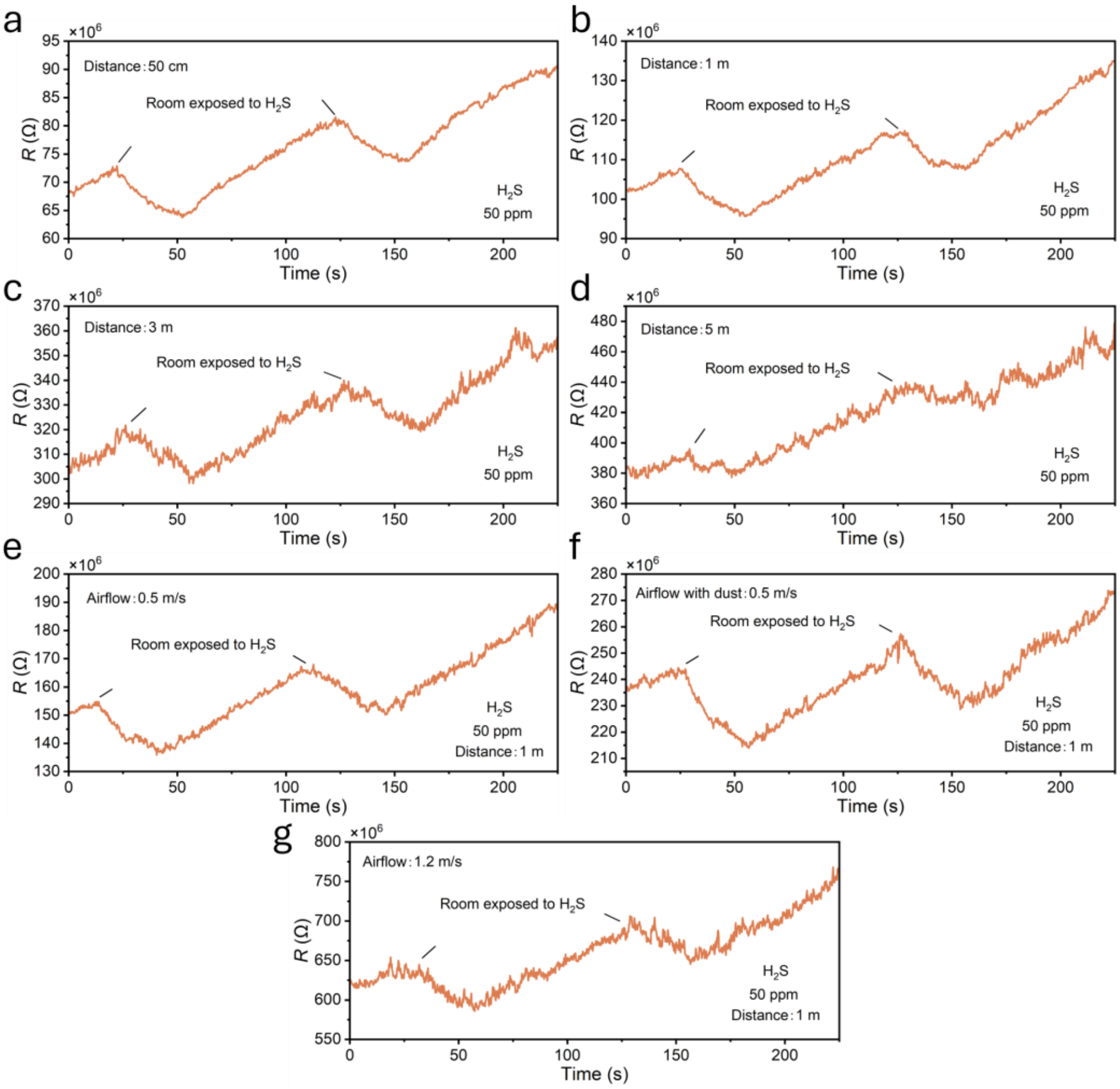

Supplementary Fig. 37 | a-d, Response and recovery curves at different distances from the leak outlet: 0.50 m, 1.0 m, 3.0 m, and 5.0 m. **e-g,** Response and recovery curves under different airflow and dust disturbance conditions at a fixed distance of 1.0 m from the leak outlet: **e,** under 0.5 m/s weak airflow; **f,** under 0.5 m/s weak airflow with dust interference (20 mg dried, sieved soil powder); **g,** under 1.2 m/s strong airflow.

High-concentration robustness was evaluated by exposing the sensor to 200, 400, 600, 800, and 1000 ppm H_2S. The sensor exhibited rapid and reversible response/recovery across this range, with an improved signal-to-noise ratio at higher concentrations and no saturation up to 1000 ppm. The baseline returned without irreversible drift, indicating the sensor's short-term tolerance to high-concentration shocks under the tested conditions (Supplementary Fig. 38).

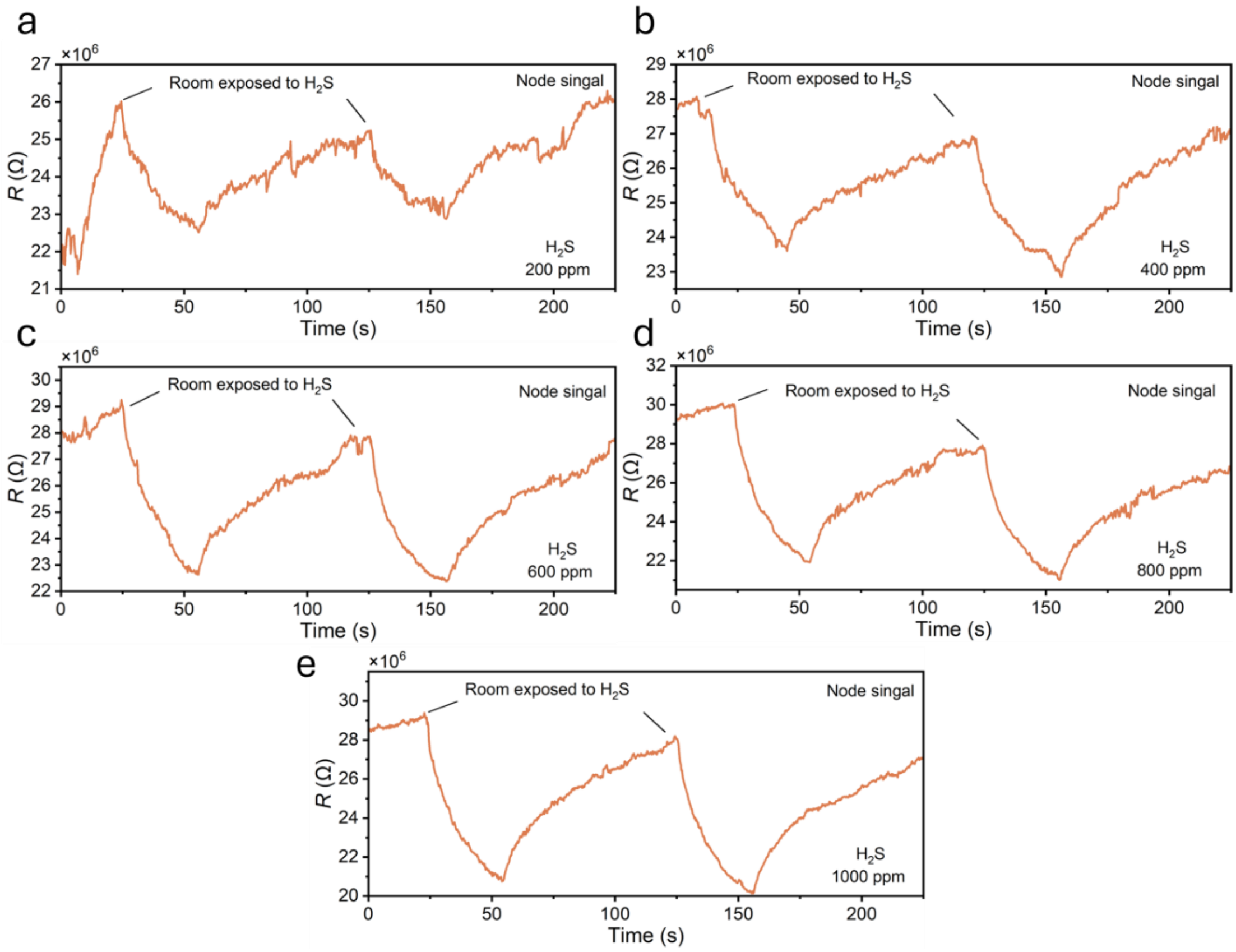

Supplementary Fig. 38 | a-e, Response and recovery curves under high concentrations of H_2S: 200 ppm, 400 ppm, 600 ppm, 800 ppm, 1000 ppm.

The sensor was tested under incremental low concentrations of H_2S (1, 2, 3, 4, 5 ppm) with short recovery times, as well as under continuously increasing high concentrations (10, 50, 100, 500, 1000 ppm) to simulate spatial gradients and slow accumulation rates. In the first experiment, incremental low concentrations of H_2S (1, 2, 3, 4, 5 ppm) were introduced sequentially, each for 30 s, followed by a short recovery period, with the sensor's response and recovery monitored after each exposure. As shown in Supplementary Fig. 39a, the sensor demonstrated clear response and recovery characteristics, with a decrease in noise as the concentration increased, suggesting an improvement in the signal-to-noise ratio. In the second experiment, the sensor was exposed to continuously increasing high concentrations of H_2S (10 ppm, 50 ppm, 100 ppm, 500 ppm, 1000 ppm). As shown in Supplementary Fig. 39b, the sensor exhibited stepwise changes in response signal with increasing concentrations, reaching a stable response after exposure to 1000 ppm. The baseline returned after the exposure without irreversible drift, further confirming that the sensor remains unaffected by high concentrations of H_2S and does not exhibit saturation or irreversible damage.

These results confirm that the sensor effectively detects both spatial concentration gradients and slow accumulation rates, with no signs of saturation at high concentrations. The sensor's performance under these conditions validates its robustness and suitability for real-world industrial leak detection, where spatially non-uniform gas distributions and gradual accumulation are common.

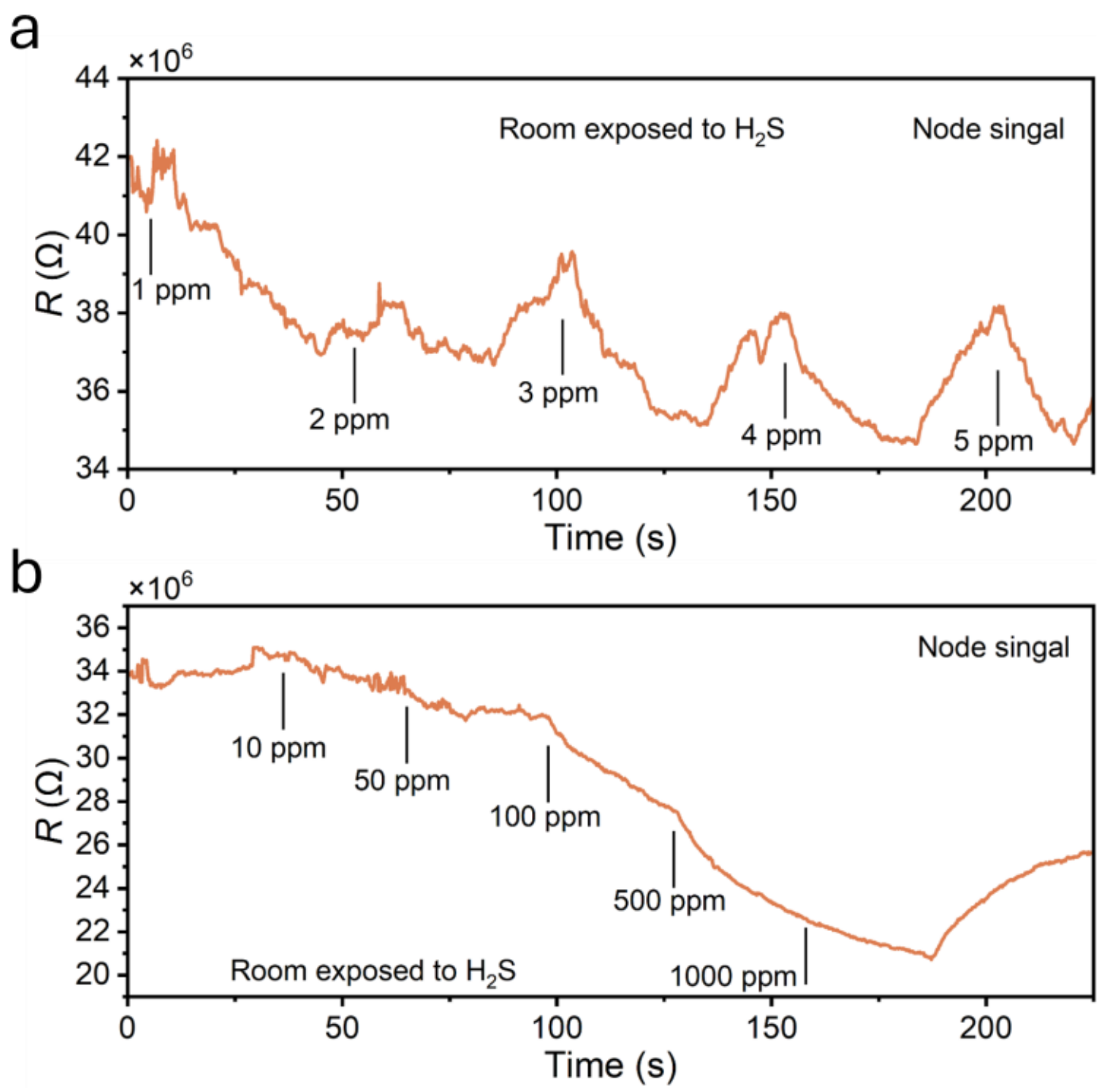

Supplementary Fig. 39 | a, Response and recovery characteristics of the sensor under incremental low concentrations of H_2S (1, 2, 3, 4, 5 ppm) with short recovery periods. **b,** Stepwise response of the sensor to continuously increasing H_2S concentrations (10, 50, 100, 500, 1000 ppm).

Supplementary Table 7 | Power consumption parameters and calculations for sensing unit.

Scenario	Operating State	Resistance (MΩ)	Operating Voltage (V)	Current (μA)	Power (μW)
Food monitoring	Standby (Idle)	170.75	3.725	0.0218	0.1264
	Sensing (Active)	11.33		0.3288	1.25495
Leakage monitoring	Standby (Idle)	55.3		0.0674	0.25091
	Sensing (Active)	29.09		0.1281	0.47691
Indoor monitoring	Standby (Idle)	189.72		0.0196	0.07314
	Sensing (Active)	85.73		0.0435	0.16186

Note: Power consumption is calculated using P=V×I, where I=V/R. The resistance values were extracted from real-time sensing data for idle and active states, respectively. The voltage of 3.725 V corresponds to the operating voltage supplied to the sensor unit during wireless measurement.

The sensing-element power reported in the main text (0.7 μW in active mode and 0.3 μW in idle mode) was calculated from the applied bias voltage and the instantaneous sensor resistance/current according to

$$P_{sensor} = V_{bias} \times I_{sensor} \qquad (11)$$

This value reflects only the intrinsic power consumption of the sensing layer.

To quantify the system-level power consumption, the total supply current of the electronics and wireless communication module was measured under the same operating conditions and data-transmission settings as used in the experiments. With a regulated supply voltage of 2.5 V, the module draws an average current of 8.19 mA, corresponding to a total power consumption of approximately 20.48 mW. This value inherently includes the duty-cycled operation of ADC sampling, microcontroller processing, and Bluetooth wireless transmission. Accordingly, the sensing-element power represents only a negligible fraction of the overall device power budget, while the dominant contribution arises from the communication and control electronics.

Power consumption for the commercial sensors listed in Supplementary Table 8 was extracted from publicly available manufacturer datasheets. These datasheets report the overall power consumption of the device, which includes both the sensing element and any additional components such as heating elements or signal conditioning circuits. Commercial datasheets generally do not provide a full breakdown of power consumption across individual modules (sensing element vs. heater vs. signal processing), so these values should be interpreted as a product-level benchmark rather than a direct one-to-one comparison under identical experimental conditions.

Supplementary Table 8 | Power Consumption and Testing Parameters for Sensor and Commercial Sensors.

Sensor Type	Model	T90 (s)	LOD (ppm)	Operating Voltage (V)	Current (A)	Power (W)	Power Source Breakdown	Source	Notes
**This work**	**H_2S**	**7**	**0.000328**	**3.725**	**0.1668 µA**	**0.6312 µW**	**Sensor**	**SI**	**Sensing element: µW-level, wireless + ADC = mW-level**
				**2.5**	**0.00819**	**0.0205**	**Wireless**	**SI**	**SI**
Commercial 1	SGX-4H_2S-100	30	/	1.3	1	1.3	Sensor	Data sheet	W-level, heat requiremtn, heat power, etc
Commercial 2	H_2S Sensor V1.0	20	0.5	3.3-5	0.02	0.066	Sensor	Data sheet	mW-level, heat requiremtn, heat power, etc
Commercial 3	2X MQ136	30	1	5	0.18	0.9	Sensor	Data sheet	mW-level, heat requiremtn, heat power, etc
Commercial 4	S-H_2S-01	30	0.1	3.3-5.5	0.05	0.025	Sensor	Data sheet	mW-level, heat requiremtn, heat power, wire transmission etc

Commercial Sensor Datasheet Sources:

Commercial Sensor 1: RS Components GmbH, Industrial Hydrogen Sulphide (H_2S) Sensor, Datasheet, available at [https://docs.rs-online.com/50d2/A700000009288588.pdf], accessed [21.01.2026].

Commercial Sensor 2: DFRobot, Fermion: H_2S Gas Sensor, Datasheet, available at [https://www.dfrobot.com/product-2702.html?srsltid=AfmBOoo57XTXfPiknsJnx7tR_0KkWUVBd00iB-ZgpHbOBsSuZ9ynozD1], accessed [21.01.2026].

Commercial Sensor 3: HUABAN, 1 x MQ-136 MQ136 Hydrogen Sulfide H_2S, Datasheet, available at [https://www.amazon.de/HUABAN-MQ-136-MQ136-Schwefelwasserstoff-H2S-Detektionsgassensormodul-Alarmsignallampe/dp/B089SQDQFJ?source=ps-sl-shoppingads-lpcontext&ref_=fplfs&smid=A169MKB0ILTINE&th=1], accessed [21.01.2026].

Commercial Sensor 4: Seeed Technology, S-H_2S-01, Datasheet, available at [https://files.seeedstudio.com/products/101990863/RES/RS485%20H2S%20Sensor%20-%20Datasheet.pdf], accessed [21.01.2026].

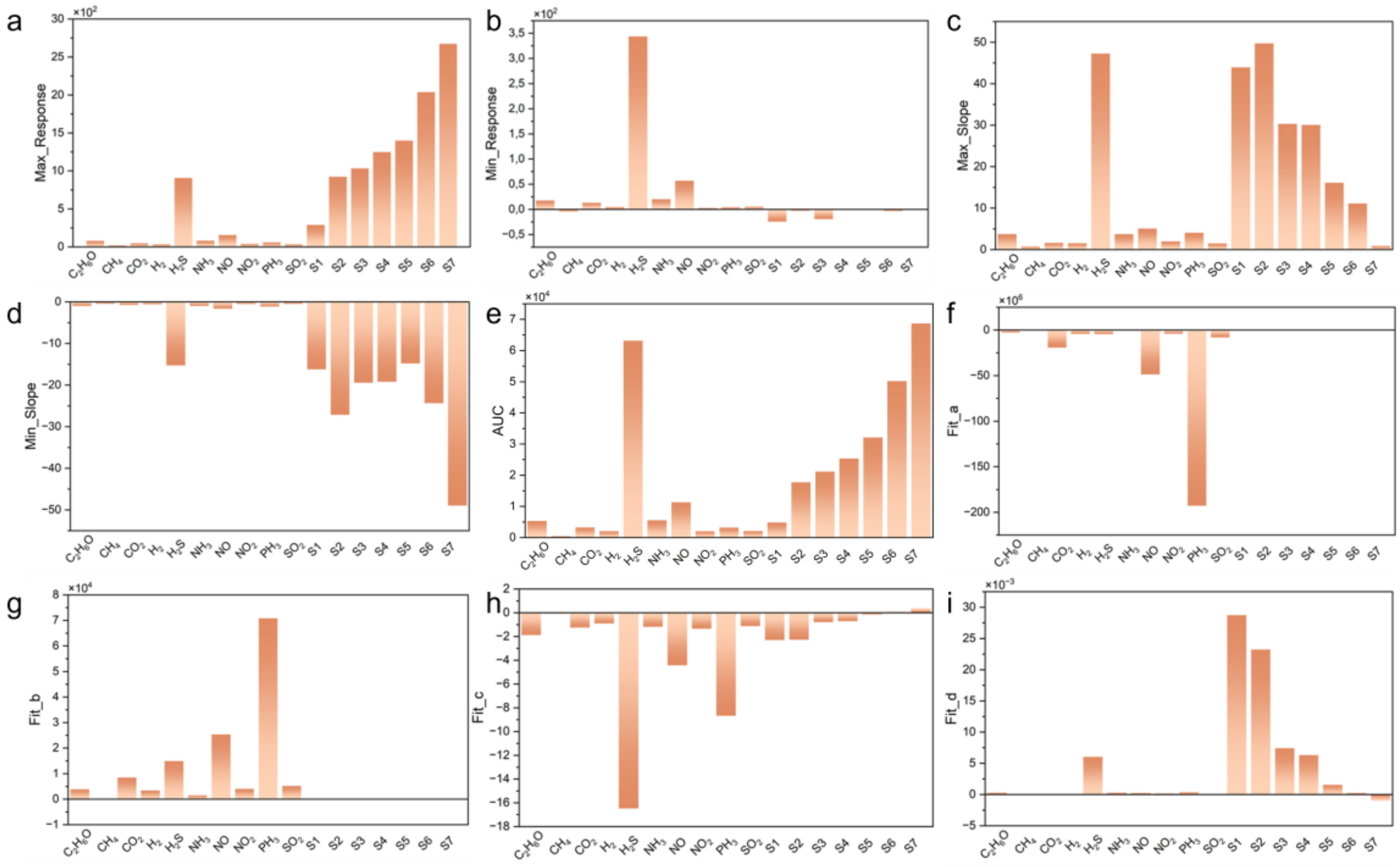

Supplementary Fig. 40 | Feature comparison of sensor responses to seven boiled egg samples with varying fermentation durations. Relative feature values derived from the sensor responses to ten different gases are compared across the seven samples. **a**, Maximum response value. **b**, Minimum response value. **c**, Maximum response slope. **d**, Minimum response slope. **e**, Area under the response curve (AUC). **f–i**, Fitted parameters Fita, Fitb, Fitc and Fitd, respectively. All features were extracted using the same analysis procedure to enable multidimensional comparison among the samples.

Supplementary References

1. Bannwarth, C., Ehlert, S. & Grimme, S. $GFN_{2-x}TB$—An accurate and broadly parametrized self-consistent tight-binding quantum chemical method with multipole electrostatics and density-dependent dispersion contributions. *J. Chem. Theory Comput.* **15**, 1652–1671 (2019).
2. Plett, C. & Grimme, S. Automated and efficient generation of general molecular aggregate structures. *Angew. Chem. Int. Ed.* **62**, e202214477 (2022).
3. Reed, A. E., Weinstock, R. B. & Weinhold, F. Natural population analysis. *J. Chem. Phys.* **83**, 735–746 (1985).
4. Caldeweyher, E., Ehlert, S., Hansen, A., Neugebauer, H. & Spicher, S. A generally applicable atomic-charge dependent London dispersion correction. *J. Chem. Phys.* **150**, 154122 (2019).
5. Wang, C., Chu, X. & Wu, M. Detection of H_2S down to ppb levels at room temperature using sensors based on ZnO nanorods. *Sens. Actuators B Chem.* **113**, 320–323 (2006).
6. Hosseini, Z. S. & Mortezaali, A. Room temperature H_2S gas sensor based on rather aligned ZnO nanorods with flower-like structures. *Sens. Actuators B Chem.* **207**, 865–871 (2015).
7. Kaur, M., Kailasaganapathi, S., Ramgir, N., Datta, N. & Kumar, S. Gas dependent sensing mechanism in ZnO nanobelt sensor. *Appl. Surf. Sci.* **394**, 258–266 (2017).
8. Li, M. L., Ye, W., Ruan, J. Z., Ren, Q. Y. & Dong, S. H. Lead-free halide double perovskite $Cs_2AgBiCl_6$ for H_2S trace detection at room temperature. *ACS Sens.* **10**, 2224–2233 (2025).
9. Chen, H. J., Lv, L., Xue, K. F., Zhang, P. H. & Du, L. L. Oral exhalation H_2S sensor based on Cu_2O/ZnO heterostructures. *ACS Sens.* **10**, 2579–2588 (2025).
10. Guo, J. H., Baek, J. W., Kang, Y., Kim, D. & Park, C. Molecular sensitizer-loaded monolayer organic semiconductors for high-performance H_2S sensors. *ACS Nano* **19**, 16175–16187 (2025).
11. Ragab, H. M., Diab, N. S., Aleid, G. M., Ab Aziz, R. & Obeidat, S. T. Selective H_2S sensor with CdS@PPy/rGO nanocomposite for sustainable air quality monitoring. *Diam. Relat. Mater.* **154**, 112155 (2025).

12. Wang, Y., Li, J., Zhang, D. X., Zhou, T. H. & Sun, M. L. Hydrothermal synthesis of Ag-doped WO_3-based H_2S room temperature sensors: Unprecedented high and fast response. *Sens. Actuators B Chem.* **438**, 137811 (2025).
13. Zhang, N., Yu, K., Li, Q., Zhu, Z. Q. & Wan, Q. Room-temperature high-sensitivity H_2S gas sensor based on dendritic ZnO nanostructures with macroscale in appearance. *J. Appl. Phys.* **103**, 103 (2008).
14. Shinde, S. D., Patil, G. E., Kajale, D. D., Gaikwad, V. B. & Jain, G. H. Synthesis of ZnO nanorods by spray pyrolysis for H_2S gas sensor. *J. Alloys Compd.* **528**, 109–114 (2012).
15. Wang, M., Luo, Q., Hussain, S., Liu, G. & Qiao, G. Sharply-precipitated spherical assembly of ZnO nanosheets for low temperature H_2S gas sensing performances. *Mater. Sci. Semicond. Process.* **100**, 283–289 (2019).
16. Zhang, W. Z., Song, L. Y., Zhao, D., Liu, T. Y. & Jiang, H. Y. Construction of hierarchical ZnO flower-like structure for boost H_2S detection at low temperature. *Sens. Actuators B Chem.* **385**, 133728 (2023).
17. Liao, L., Lu, H. B., Li, J. C., He, H. & Wang, D. F. Size dependence of gas sensitivity of ZnO nanorods. *J. Phys. Chem. C* **111**, 1900–1903 (2007).
18. Mei, L., Chen, Y. J. & Ma, J. M. Gas sensing of SnO_2 nanocrystals revisited: Developing ultra-sensitive sensors for detecting the H_2S leakage of biogas. *Sci. Rep.* **4**, 6028 (2014).
19. Uma, S., Vignesh, D. & Shobana, M. K. Synthesis of heterostructured nanocomposite $MgFe_2O_4/MoO_3$ for H_2S sensing: Experimental and theoretical approach by DFT. *Sens. Actuators B Chem.* **425**, 136950 (2025).
20. Onkar, S. G., Nagdeote, S. B., Wadatkar, A. S. & Kharat, P. B. Gas sensing behavior of ZnO thick film sensor towards H_2S, NH_3, LPG and CO_2. *J. Phys. Conf. Ser.* **1644**, 012060 (2020).
21. Huang, H., Xu, P., Zheng, D., Chen, C. & Li, X. Sulfuration–desulfuration reaction sensing effect of intrinsic ZnO nanowires for high-performance H_2S detection. *J. Mater. Chem. A* **3**, 6330–6339 (2015).
22. Yu, Z. Y., Gao, J., Xu, L. X., Liu, T. Y. & Liu, Y. Y. Fabrication of lettuce-like ZnO gas sensor with enhanced H_2S gas sensitivity. *Crystals* **10**, 145 (2020).
23. Zheng, M. J., Cheng, Y. D., Zhang, X. L., Liu, H. N. & Xu, H. Y. Atomic Ru species driven

SnO_2-based sensor for highly sensitive and selective detection of H_2S in the ppb-level. *ACS Sens.* **10**, 1093–1104 (2025).

24. Sheokand, A., Kumar, V., Sindhu, S., Bulla, M. & Dahiya, R. Development of low detection limit ultra-sensitive H_2S sensor based on MIL-88B(Fe) derived α-Fe_2O_3. *Sens. Actuators A Phys.* **382**, 116121 (2025).
25. Wang, D., Tan, R., Yu, H. B., Chen, H. & Ma, X. High response H_2S chemiresistive gas sensor based on multidimensional CuO/CeO_2: The application for real-time portable alarm device. *Sens. Actuators B Chem.* **424**, 136884 (2025).
26. Fan, C., Sun, F., Wang, X., Huang, Z. & Keshvardoostchokami, M. Synthesis of ZnO hierarchical structures and their gas sensing properties. *Nanomaterials* **9**, 1277 (2019).
27. Zhu, Y. D., Wang, Y. Y., Duan, G. T., Zhang, H. W. & Li, Y. In situ growth of porous ZnO nanosheet-built network film as high-performance gas sensor. *Sens. Actuators B Chem.* **221**, 350–356 (2015).
28. Xi, J. C., Liang, K., Yao, N., Yuan, H. Y. & Cui, J. Y. Mo-doping induced mixed phase $BiVO_4$ with hierarchical structure for enhanced H_2S sensing. *Sens. Actuators B Chem.* **423**, 136710 (2025).
29. Vuong, N. M., Chinh, N. D., Huy, B. T. & Lee, Y.-I. CuO-decorated ZnO hierarchical nanostructures as efficient and established sensing materials for H_2S gas sensors. *Sci. Rep.* **6**, 26736 (2016).
30. Hoa, T. T. N., Le, D. T. T., Toan, N. V., Duy, N. V. & Hung, C. M. Highly selective H_2S gas sensor based on WO_3-coated SnO_2 nanowires. *Mater. Today Commun.* **26**, 102094 (2021).
31. Na, H. B., Zhang, X. F., Deng, Z. P., Xu, Y. M. & Huo, L. H. Large-scale synthesis of hierarchically porous ZnO hollow tubule for fast response to ppb-level H_2S gas. *ACS Appl. Mater. Interfaces* **11**, 11627–11635 (2019).
32. Diao, K., Zhou, M., Zhang, J., Tang, Y. & Wang, S. High response to H_2S gas with facile synthesized hierarchical ZnO microstructures. *Sens. Actuators B Chem.* **219**, 30–37 (2015).
33. Nakate, U. T., Yu, Y. T. & Park, S. Hydrothermal synthesis of ZnO nanoflakes composed of fine nanoparticles for H_2S gas sensing application. *Ceram. Int.* **48**, 28822–28829 (2022).
34. Li, Z. J., Huang, Y. W., Zhang, S. C., Chen, W. M. & Kuang, Z. A fast response & recovery

H_2S gas sensor based on α-Fe_2O_3 nanoparticles with ppb level detection limit. *J. Hazard. Mater.* **300**, 167–174 (2015).

35. Shewale, P. S., Agawane, G. L., Shin, S. W., Moholkar, A. V. & Lee, J. Thickness dependent H_2S sensing properties of nanocrystalline ZnO thin films derived by advanced spray pyrolysis. *Sens. Actuators B Chem.* **177**, 695–702 (2013).
36. Mortezaali, A. & Moradi, R. The correlation between the substrate temperature and morphological ZnO nanostructures for H_2S gas sensors. *Sens. Actuators A Phys.* **206**, 30–34 (2014).
37. Zhao, G. D., Xuan, J. Y., Liu, X. L., Jia, F. C. & Sun, Y. P. Low-cost and high-performance ZnO nanoclusters gas sensor based on new-type FTO electrode for the low-concentration H_2S gas detection. *Nanomaterials* **9**, 435 (2019).
38. Galstyan, V., Poli, N. & Comini, E. Highly sensitive and selective H_2S chemical sensor based on ZnO nanomaterial. *Appl. Sci.* **9**, 1167 (2019).
39. Kim, J. & Yong, K. Mechanism study of ZnO nanorod-bundle sensors for H_2S gas sensing. *J. Phys. Chem. C* **115**, 7218–7224 (2011).
40. Su, T. Y., Chen, Y. Z., Wang, Y. C., Tang, S. Y., Shih, Y. C., Cheng, F. L., Wang, Z. M. M., Lin, H. N. & Chueh, Y. L. Highly sensitive, selective and stable NO_2 gas sensors with a ppb-level detection limit on 2D-platinum diselenide films. *J. Mater. Chem. C* **8**(14), 4851–4858 (2020).
41. Kaiser, A., Mauritz, T., Bansmann, J., Biskupek, J., Herr, U., & Thonke, K. Mechanism of optical and electrical H_2S gas sensing of pristine and surface functionalized ZnO nanowires. *ACS Omega* **9**(51), 50188–50200 (2024).
42. Ahmad, T., Pandey, V., Husain, M. S., & Munjal, S. Structural and spectroscopic analysis of pure phase hexagonal wurtzite ZnO nanoparticles synthesized by sol-gel. *Mater. Today: Proc.* **49**, 1694-1697 (2022).
43. da Silva-Neto, M. L., de Oliveira, M. C. A., Dominguez, C. T., Lins, R. E. M., Rakov, N., de Araújo, C. B., de Souza Menezes, L., de Oliveira, H. P., & Gomes, A. S. L. UV random laser emission from flexible ZnO–Ag-enriched electrospun cellulose acetate fiber matrix. *Sci. Rep.* **9**, 11765 (2019).
44. Vivek, C., Balraj, B. & Thangavel, S. Structural, optical and electrical behavior of

ZnO@Ag core–shell nanocomposite synthesized via novel plasmon-green mediated approach. *J. Mater. Sci. Mater. Electron.* **30**, 11220–11230 (2019).

45. Matai, I., Sachdev, A., Dubey, P., Kumar, S. U., Bhushan, B., & Gopinath, P. Antibacterial activity and mechanism of Ag–ZnO nanocomposite on *S. aureus* and GFP-expressing antibiotic-resistant *E. coli*. *Colloids Surf. B Biointerfaces* **115**, 359-367 (2014).
46. Alves, L. M. S., Oliveira, F. S., Romão, E. C., da Luz, M. S. & dos Santos, C. A. M. A simple and precise way to determine electrical resistivity of isotropic conductors: Simplifying the four-probe method. *AIP Adv.* **13**(3) (2023).
47. Instrument, K. Four-probe resistively and Hall voltage measurements with the Model 4200-SCS. *Plan Canada* **41**, 56-58 (2002).
