# Supplementary material for: A robust high-performance H2S sensor enabling real-world wireless monitoring
Source: Nat Commun. 2026 Aug 19;17:10251. doi: 10.1038/s41467-026-76707-w (PMC13616922; doi:10.1038/s41467-026-76707-w)
Supplement: Supplementary file 2 [file 41467_2026_76707_MOESM2_ESM.pdf]

## The Description of Additional Supplementary Files

**Title:** Supplementary Data 1

**Description:**

Supplementary Data 1 contains the computational input and output files generated in this study for structural modelling and first-principles calculations. The data package is organised according to the model configuration and calculation type and includes the files required to reproduce and independently verify the principal computational results reported in the manuscript.

1. **Grain-boundary model calculations.** Atomic structural models and the associated calculation files are provided for the four model sets labelled GB0, GB0.5, GB1 and GB2 in the manuscript. These files include the corresponding initial and optimised structures, calculation settings and relevant output files used to evaluate the influence of the different grain-boundary configurations.
2. **Work-function calculations.** The package contains the surface or slab models and all relevant input and output files used for the work-function analysis, including the optimised structures, electronic-structure calculation outputs, electrostatic-potential data, Fermi-level information and the files used to determine the vacuum level and calculate the work function.
3. **Vacancy-containing models and adsorption-energy calculations.** Computational files are included for the pristine structures and for structures containing oxygen vacancies and Zn vacancies. For each relevant adsorption system, the package provides the model structures and total-energy calculation files for the isolated adsorbate reference, the clean or defective substrate and the corresponding adsorbed configuration. These files contain the energies and structural information required to reproduce the reported adsorption-energy calculations.
4. **Bader charge analysis.** The charge-density files, Bader-analysis outputs and atom-resolved charge data are provided for the relevant pristine and vacancy-containing adsorption systems. These files support the calculation and verification of charge redistribution and net charge transfer between the adsorbates and the model surfaces.

The file and folder organisation identifies the corresponding structural model, defect type, adsorption configuration and calculation category, thereby enabling direct linkage between the deposited files and the computational analyses described in the manuscript. The numerical data underlying the plots shown in the main and supplementary figures are provided separately in the Source Data Excel file.
