# Supplementary material for: A robust high-performance H2S sensor enabling real-world wireless monitoring
Source: Nat Commun. 2026 Aug 19;17:10251. doi: 10.1038/s41467-026-76707-w (PMC13616922; doi:10.1038/s41467-026-76707-w)
Supplement: Supplementary file 4 [file 41467_2026_76707_MOESM5_ESM.pdf]

nature portfolio

Peer Review File

A robust high-performance H2S sensor enabling real-world wireless monitoring

Corresponding Author: Professor Gianaurelio Cuniberti

This file contains all reviewer reports in order by version, followed by all author rebuttals in order by version.

Version 0:

Reviewer comments:

Reviewer #1

(Remarks to the Author)
This paper focuses on the development and application of a high-performance wireless H_2S sensor, systematically presenting the sensor's core value in material design, performance breakthroughs, and practical implementation.
1. The main manuscript proposes that grain boundaries and Schottky barriers synergistically regulate carrier transport, but does not explicitly establish the correlation between grain boundary density and barrier height for samples at different pyrolysis temperatures. It is recommended to supplement relevant quantitative data by integrating the EBSD grain boundary statistics and UPS test results from the supplementary materials.
2. The authors should supplement the study with data on the dynamic evolution of electronic structures during H_2S adsorption. It is recommended to combine the DFT simulations in the supplementary materials with new in situ characterization experiments, such as in situ Raman spectroscopy, in situ FT-IR spectroscopy, or in situ impedance spectroscopy, to bridge the gap between static characterization and dynamic performance evidence.
3. Since the authors emphasize the sensor's monitoring capability in real-world conditions, evaluating the impact of humidity variations on performance is crucial. In practical applications, humidity fluctuations can significantly interfere with the response of metal oxide sensors. It is recommended to add humidity gradient experiments (e.g., 15%, 30%, 50%, 70% RH) to test the ZMA550's response values to 1 ppm H_2S at different humidity levels. Combine this with XPS measurements of surface hydroxyl content changes to analyze the mechanism by which humidity affects performance through “competitive adsorption” or “electron transfer interference.”
4. Nanomaterials based on metal-organic frameworks (MOFs) are frequently employed for H2S gas sensors; the structural design of the materials in this work is not novel.
5. The introduction does not elaborate on how the current limitations of sensors specifically impact monitoring requirements in practical application scenarios, such as anaerobic environments for food preservation or industrial enclosed spaces.
6. Given the focus of this study, the authors have not addressed the necessity of two core innovative strategies in the introduction: regulating grain boundary density within metal-organic framework derivative materials and modulating the Schottky barrier. It is recommended that the introduction be supplemented with existing research highlighting the advantages of metal-organic framework derivative materials in terms of controllable microstructure and high specific surface area. Alternatively, an explanation could be provided on how the Schottky interface achieves high selectivity/high sensitivity in gas sensing. This approach would both prevent the novel points from appearing abrupt and help readers swiftly grasp the core logic of the research design, thereby paving the way for a more natural transition to the technical details in the subsequent main text.
7. How did the authors arrive at the detection limit of 0.328 ppb for the sensor? Was this obtained through actual testing or calculated?
8. The author should supplement performance stability data under different interfering gases to enhance the completeness of the comparison.
9. The manuscript states that the sensor's average power consumption is below 0.7 μW, but does not specify the test scenario for this power consumption data (e.g., static monitoring state, dynamic response state, or power consumption variations under different H_2S concentrations). The power consumption data for commercial sensors (66–100 mW) also fails to clarify whether the test conditions align with this study (e.g., whether the same operating temperature and response trigger threshold were used). It is recommended to supplement the power consumption testing with specific experimental parameters (e.g., test temperature, gas concentration, data acquisition frequency). Additionally, the source literature and testing conditions for the commercial sensor power consumption data should be cited to ensure fair comparison and data reproducibility.

10. The manuscript defines sensor response as ‘S = (Ra/Rg-1) × 100%’. However, Table S1 in the supplementary materials defines response as ‘Ra/Rg’. Such inconsistencies may compromise the fairness of comparative results.

Reviewer #2

(Remarks to the Author)
This study proposes an ultra-low-power wireless hydrogen sulfide sensor based on metal-organic framework-derived ZnO, featuring exceptional sensitivity and rapid response time for trace gas detection. Although this approach addresses significant challenges in gas sensing and demonstrates remarkable performance metrics, the novelty of the sensor design—particularly the integration of Schottky barrier and grain boundary engineering—has been extensively documented in the literature for similar MOF-derived sensors and Schottky barrier modification techniques. This paper fails to sufficiently articulate how this specific solution significantly advances the state of the art.

1. MOF-derived metal oxides for H_2S sensing have been a research hotspot in recent years, but this paper fails to clearly articulate its innovative differentiation from similar work, resulting in insufficient novelty. In 2024, Montoro et al. (Composites Part B: Engineering Volume 283, 2024, 111637) reported “defect-engineered ZnO derived from MOFs for low-concentration H_2S detection.” The “grain boundary engineering” approach in this paper essentially extends the concept of “defect engineering” and does not propose novel structural design strategies.
2. The experiment used “anaerobic fermentation of boiled eggs” to simulate food spoilage, but did not specify the storage conditions of the eggs (such as whether they were protected from light or whether there were differences in microbial contamination). It also failed to measure the concentrations of other volatile organic compounds (such as amines and organic acids) during fermentation, making it impossible to rule out interference from sensor responses to other gases.
3. Pipe leak detection was tested only under ideal conditions of 5 cm distance and 50 ppm H2S, failing to simulate long-distance detection (e.g.,1-5 m), airflow disturbances, or dust interference in actual industrial environments. Confined space testing used only a sealed chamber with 1 ppm H_2S, neglecting the irreversible damage high-concentration H2S (e.g., above 100 ppm) inflicts on sensors in industrial scenarios.
4. XPS and Raman spectroscopy detected oxygen vacancies and zinc vacancies respectively, but the contribution of these two defects to H_2S adsorption was not compared via DFT calculations. The statement that oxygen vacancies are the primary defect is too general to explain why ZMA450 exhibits weaker sensing performance despite having a higher oxygen vacancy concentration (greater proportion in XPS measurements) than ZMA550.
5. The paper claims the sensor responds 4-5 times faster than commercial sensors with 10^5 times lower power consumption. However, Table S1 lacks specific commercial sensor models, testing conditions (e.g., humidity, interfering gas concentrations), and key parameters (e.g., typical detection limits of 0.1-1 ppm for commercial sensors). Relying solely on textual descriptions makes it impossible to verify the authenticity of these performance advantages.
6. BET data shows ZMA450 has the highest specific surface area among all samples, yet its sensing response is significantly lower than ZMA550. The article fails to analyze the trade-off between specific surface area, active site efficacy, and carrier mobility, leaving unexplained why high surface area did not yield high response.
7. Performance testing was conducted only at ~15% RH. The impact of high humidity (e.g., RH>60%, common in food refrigeration and industrial damp environments) and temperature fluctuations (e.g., -10~40°C) on sensor response was not evaluated.

Reviewer #3

(Remarks to the Author)
The manuscript presents an ultra-low-power wireless H2S sensor based on MOF-derived ZnO with engineered grain boundaries and Au/ZnO Schottky interfaces, enabling exceptional room-temperature sensitivity, a 0.328-ppb detection limit, and a rapid 7-second response. Comprehensive structural, electronic, and theoretical analyses validate the synergistic sensing mechanism, while real-world demonstrations- such as food spoilage detection, pipeline leakage monitoring, and confined-space safety-highlight the device’s practical applicability. Besides these, there are still some aspects of the detection methods and characterisations that need clarification; therefore, this paper may require major revision. We hope that the authors will give serious consideration to comments or suggestions.

1. The authors claim an optimal grain boundary density yields peak performance. What is the correlation between grain boundary density (from TEM/BET) and charge transport parameters? Can you provide a direct measurement (e.g., EIS fitting, mobility estimations) linking grain boundary density to transport and sensing response?
2. They attribute the exceptional sensing performance to a ~0.4 eV reduction in the Au/ZnO Schottky barrier. How was the barrier change extracted from UPS, and can alternative explanations (surface dipoles, interface states, defect redistribution) be excluded?
3. The sensing mechanism heavily relies on oxygen vacancies. Is there direct evidence (e.g., EPR, photoluminescence spectroscopy) confirming vacancy concentration trends with temperature beyond XPS deconvolution?
4. The authors propose the formation of SH- and Zn-SH complexes. Do you have spectroscopic evidence (e.g., FTIR, in situ Raman) to support the chemical reaction pathways?
5. All tests were performed at ~15% RH. How does the sensor behave across a realistic humidity range (30–90% RH) relevant for industrial and food-storage environments?
6. The 270-day stability data show little drift, which is unusual for ZnO in ambient conditions. Was the device continuously operated or periodically tested? How is contamination, electrode corrosion, or ZnS formation ruled out over long exposure cycles?
7. Selectivity is shown for 10 gases, but cross-interference under mixtures is unclear. How does the sensor respond when H2S coexists with oxidizing gases (e.g., NO2, SO2) or reductive gases (e.g., NH3) simultaneously?
8. Baseline anchoring using second-derivative zero points may artificially influence ultra-low concentration detection. Can

you demonstrate that the LOD (0.328 ppb) is not dependent on the correction protocol?
9. The authors state a 4-5× faster response and 105-fold lower power consumption compared to commercial sensors. Which specific commercial models were used as benchmarks, and under what conditions?
10. The upper concentration limit is not clearly defined. Does the sensor saturate beyond 100 ppm, and what is the linearity range?
11. Power values are calculated from instantaneous resistance and voltage. What is the total power, including the wireless module's duty cycle, ADC sampling, and Bluetooth transmission? Is the 0.7 μW value just the sensing layer, or the entire system?
12. Does H2S exposure affect the stability of the electronics (corrosion, signal noise, leakage currents)?
13. The leakage test uses a localized defect at 50 ppm release. How does the sensor behave at realistic diffusion gradients or turbulent flow conditions?
14. The confined chamber demonstration uses a uniform 1 ppm distribution. Can the sensor detect spatial gradients or very slow accumulation rates typical of real industrial settings?
15. The grain-boundary models in DFT represent small oligomeric structures. How do these simplified structures reflect the realistic energetics of ZnO grain boundary? Have you validated the computational predictions with experimental barrier measurements at varying grain boundary densities?
16. Some materials in Tab. S1 show comparable high responses. Can the authors more rigorously justify the claim of being state-of-the-art, perhaps by normalizing metrics (response per surface area, per power, per LOD)?
17. H2S can convert ZnO to ZnS. Has the material been examined post-measurement (XRD, XPS) to ensure no irreversible ZnS formation occurred?
18. The paper states sensors were fabricated "in triplicate." Can you provide statistical variation (standard deviation, error bars) for response values, LOD, and power consumption across multiple sensors?

Version 1:

Reviewer comments:

Reviewer #1

(Remarks to the Author)
There is still some minor issues,

1. Could the authors please provide additional systematic characterization results, such as TEM and XPS, for the samples after the 450-day long-term cycling test? This would help verify whether phenomena such as surface sulfate accumulation, irreversible changes in oxygen vacancy concentration, and grain boundary relaxation occur during long-term cycling, thereby further confirming the reversibility and structural stability of the sensing mechanism under long-term use.
2. How did the authors assess the impact of structural differences between the simplified grain boundary/defect model and actual polycrystalline ZnO on the computational results?
3. The authors emphasize a detection limit of 0.328 ppb. Could you please provide direct exposure test data for H2S at sub-ppb levels (e.g., 0.1 ppb, 0.3 ppb, 0.5 ppb), including complete response/recovery curves and multi-cycle repeatability results, to experimentally demonstrate the sensor's actual ultra-low concentration detection capability, rather than relying solely on theoretical calculations?

Reviewer #3

(Remarks to the Author)
Author has incorporated all the queries.

Version 2:

Reviewer comments:

Reviewer #1

(Remarks to the Author)
The authors have answer all the issues, and it is ready to be accepted.

Open Access This Peer Review File is licensed under a Creative Commons Attribution 4.0 International License, which permits use, sharing, adaptation, distribution and reproduction in any medium or format, as long as you give appropriate credit to the original author(s) and the source, provide a link to the Creative Commons license, and indicate if changes were made.
In cases where reviewers are anonymous, credit should be given to 'Anonymous Referee' and the source.
The images or other third party material in this Peer Review File are included in the article’s Creative Commons license, unless indicated otherwise in a credit line to the material. If material is not included in the article’s Creative Commons license and your intended use is not permitted by statutory regulation or exceeds the permitted use, you will need to obtain permission directly from the copyright holder.
To view a copy of this license, visit https://creativecommons.org/licenses/by/4.0/

Responses to the reviewers' comments

Manuscript ID: NCOMMS-25-71902-T

Title: A robust high-performance H_2S sensor enabling real-world wireless monitoring

***Editor's comment**:* Thank you again for submitting your manuscript "A robust high-performance H_2S sensor enabling real-world wireless monitoring" to Nature Communications. We have now received reports from 3 reviewers and, after careful consideration, we have decided to invite a major revision of the manuscript.

As you will see from the reports copied below, the reviewers raise important concerns. We find that these concerns limit the strength of the study, and therefore we ask you to address them with additional work. Without substantial revisions, we will be unlikely to send the paper back to review. In particular, it seems that deeper characterization, experimental validation, and evidence supporting conclusions; operation at varied humidity and with interfering gases, including cross-interference; and understanding of the contribution of O vs Zn vacancies will be needed.

If you feel that you are able to comprehensively address the reviewers' concerns, please provide a point-by-point response to these comments along with your revision. Please show all changes in the manuscript text file with track changes or colour highlighting. If you are unable to address specific reviewer requests or find any points invalid, please explain why in the point-by-point response.

Reply: Thank you for your constructive feedback on our manuscript titled *"A robust high-performance H_2S sensor enabling real-world wireless monitoring"*. We appreciate the detailed insights provided by both the editor and the reviewers. We have carefully studied the reviewers' comments and fully recognized the importance of the issues raised, particularly regarding: **the need for deeper material characterization and mechanistic validation, extended experimental evidence supporting key conclusions, sensor performance under variable humidity and interfering gases, including cross-interference, and clarification of the relative contributions of O vs Zn vacancies**.

We fully agree that addressing these points will significantly strengthen scientific rigor and the broader relevance of the work. We confirm that our team can comprehensively address all major concerns raised by the reviewers. Specifically, we have already conducted the following additional experiments and analyses:

1. Deeper characterization and experimental validation:

[1]. We conducted HRTEM, PXRD, XPS, and EDX characterization of the sensing layer after long-term exposure to H_2S.

[2]. A bar chart was created to show the sensor response on the left Y-axis and the LOD on the right Y-axis for three sensor batches, with error bars included.

[3]. Deep UPS spectra were recorded to show the secondary electron cutoff, which was used to determine the work function of the samples, and the Schottky barrier height was derived from UPS for samples pyrolyzed at 500–650 °C.

[4]. EBSD images were obtained for samples pyrolyzed at temperatures ranging from 450–650 °C.

[5]. We analyzed the grain boundary concentration and density of samples pyrolyzed at 450–650 °C.

[6]. In situ FTIR spectra were collected for ZMA550 during H_2S adsorption and desorption processes.

[7]. EPR spectra were measured for ZMA350, ZMA450, ZMA550, and ZMA650 samples, showing relative intensity versus the g value.

[8]. We recorded response and recovery curves at different distances from the leak outlet.

[9]. Response and recovery curves were obtained under various airflow and dust disturbance conditions at a fixed distance of 1.0 m from the leak outlet.

[10]. We tested the response and recovery curves under high concentrations of H_2S, including 200 ppm, 400 ppm, 600 ppm, 800 ppm, and 1000 ppm.

[11]. The sensor's response and recovery characteristics were tested under incremental low concentrations of H_2S (1, 2, 3, 4, 5 ppm) with short recovery periods.

[12]. The sensor's stepwise response was evaluated by continuously increasing H_2S concentrations (10, 50, 100, 500, 1000 ppm).

[13]. Power consumption parameters were measured, and calculations for the sensing unit were performed.

[14]. Power consumption and testing parameters were compared between the sensor and commercial sensors.

[15]. Since the initial submission, we have extended the sensor stability testing period to 450 days, with an additional 6 months of data, and have updated the manuscript to reflect this longer testing period.

2. Operation at varied humidity and with interfering gases:

[1]. We performed sensing verification at different humidity levels, ranging from 15% RH to 90% RH.

[2]. XPS measurements were performed on the sensing layers after operation at various humidity levels to elucidate the underlying mechanism.

[3]. XPS results were analyzed to verify the amount of surface hydroxylation under different humidity conditions.

[4]. We conducted a dynamic interference stability test by exposing the sensor to 3 ppm H_2S over 18 consecutive response–recovery cycles to evaluate the robustness of the H_2S sensing signal in realistic multi-gas environments.

[5]. During each cycle, one set of interfering gases was introduced simultaneously with H_2S, while all other experimental parameters (*e.g.,* concentration, total flow rate, temperature, and bias/measurement settings) were kept constant.

3. Understanding the contribution of O vs Zn vacancies:

[1]. DFT calculations were conducted to evaluate the adsorption of H_2S at both oxygen and zinc vacancy sites, including the adsorption structures, differential charge densities, and charge-transfer analysis.

[2]. Adsorption energy comparisons (E_{bind}) and Bader charge-transfer analyses were conducted to understand the interaction strength at these sites.

[3]. EPR analysis was performed to investigate the role of oxygen vacancies in the sensing mechanism.

[4]. A comprehensive analysis combining XPS and UV-vis spectroscopy was conducted to further characterize the material's surface properties and electronic structure.

We have revised the manuscript substantially to incorporate these results. A point-by-point response document is provided below, addressing the reviewers' comments with detailed explanations. All modifications in the manuscript and supporting information have clearly highlighted in red. We appreciate the constructive guidance from you and the reviewers. We are confident that the revised version has addressed all concerns comprehensively and significantly strengthened the manuscript.

To Reviewer: 1

General comments: This paper focuses on the development and application of a high-performance wireless H_2S sensor, systematically presenting the sensor's core value in material design, performance breakthroughs, and practical implementation.

Reply: We sincerely thank the reviewer for the overall positive evaluation of our work and for recognizing the value of our efforts in developing a high-performance wireless H_2S sensor that integrates rational material design, significant performance advances, and practical real-world implementation. We appreciate your constructive insights, which have helped us further strengthen the depth and rigor of the manuscript. In the revised version, we have carefully addressed all your comments in a detailed point-by-point response that thoroughly explains how each issue has been resolved. All revisions in the manuscript have been highlighted in red. We are grateful for your thoughtful comments, which have substantially improved the quality and clarity of our work.

Comment 1: The main manuscript proposes that grain boundaries and Schottky barriers synergistically regulate carrier transport, but does not explicitly establish the correlation between grain boundary density and barrier height for samples at different pyrolysis temperatures. It is recommended to supplement relevant quantitative data by integrating the EBSD grain boundary statistics and UPS test results from the supplementary materials.

Reply: We sincerely thank the reviewer for this insightful comment regarding the correlation between grain boundary density and Schottky barrier characteristics across samples prepared at different pyrolysis temperatures.

In response, we have systematically integrated EBSD grain boundary statistics and UPS-derived electronic structure parameters for representative samples pyrolyzed at 450, 500, 550, 600, and 650 °C. As shown in the newly added Supplementary Fig. 18-21 and Tab. 2-3, EBSD analysis reveals a clear microstructural evolution with increasing pyrolysis temperature: the average grain size progressively increases, accompanied by a monotonic decrease in grain boundary density. We note that for samples pyrolyzed at lower temperatures (≤450 °C), the grain size becomes too small to be reliably resolved by EBSD, leading to significant indexing uncertainty; therefore, these samples were intentionally excluded from quantitative grain boundary analysis to ensure data reliability and avoid overinterpretation.

Correspondingly, UPS measurements were performed on the same set of samples (450-650 °C) after H_2S sensing tests to extract the work function and valence band onset, from which the effective Schottky barrier height was determined (Supplementary Fig. 18 and Tab. 2). The UPS results demonstrate a consistent reduction in barrier height with increasing pyrolysis temperature, in good agreement with the observed decrease in grain boundary density. By directly correlating the EBSD-derived grain size and grain boundary density with the UPS-derived Schottky barrier height, these results establish a quantitative relationship among grain size, grain boundary density, and interfacial barrier height across the temperature series. This directly substantiates the proposed synergistic role of grain boundaries and Schottky barriers in regulating carrier transport.

We further clarify that the UPS-derived barrier values are sensitive to the interfacial state of the sensing layer under specific conditions. In this work, the structure–barrier correlation across the pyrolysis-temperature series is established using UPS measurements performed on the sensing layers after H_2S sensing tests, enabling a direct comparison with EBSD-derived grain boundary statistics for the same set of samples. In contrast, the gas-induced barrier modulation discussed in the manuscript is obtained from the same sensing device before and after gas exposure, reflecting the effective operating-state barrier at the Au–ZnO junction.

In addition, the Schottky barrier height is not directly measured by UPS, but derived from the UPS-extracted work function and valence band onset. The work function was obtained from the secondary electron cutoff using linear extrapolation of the cutoff edge to the baseline, and the valence band onset was determined by linear extrapolation of the leading edge of the valence band spectrum. Because both parameters rely on edge extrapolation and energy referencing, small session-to-session variations for ZMA550 (WF: 3.9 → 3.8 eV; VB onset: 3.0 → 3.1 eV) can arise from fitting-window selection and surface sensitivity. Consequently, minor differences in the derived barrier height may originate from the extraction procedure, while the relative trend across the sample series remains unaffected.

These new results and the associated discussion have been added to the Supplementary Information (Supplementary Fig. 18-21 and Tab. 2-3) and explicitly referenced in the revised manuscript (Page 11, Line 319-330) and are also highlighted in red accordingly, thereby strengthening the experimental basis of the proposed grain-boundary/Schottky-barrier synergistic mechanism.

Revised parts of the manuscript are as follows:

To further elucidate the structural origin of the interfacial barrier characteristics, we establish a direct correlation among grain size, GB density, and Schottky barrier height across samples prepared at different

pyrolysis temperatures (Supplementary Fig. 19). EBSD analysis shows that increasing pyrolysis temperature induces progressive grain growth accompanied by a monotonic reduction in GB density (Supplementary Fig. 20 and Tab. 3). Correspondingly, UPS measurements reveal a systematic decrease in the Schottky barrier height derived from the work function and valence band onset (Supplementary Fig. 18 and Tab. 2). These results demonstrate that GB density, governed by the grain size evolution, critically determines the interfacial barrier landscape, thereby providing a quantitative structural basis for the synergistic grain-boundary-assisted barrier regulation mechanism.

Additional revised parts of manuscript are as following:

As detailed in Supplementary Fig. 21, the direct transport measurements, together with EBSD-derived grain-boundary statistics and correlation analysis with sensing response, establish a quantitative link between grain boundary density, charge transport, and sensing performance.

Revised parts of SI are as following:

A direct correlation among grain size, GB density, and Schottky barrier height across samples prepared at different pyrolysis temperatures (Supplementary Fig. 19). The EBSD analysis was employed to quantify the grain size and grain-boundary density of ZnO sensing layers pyrolyzed at 450–650 °C. Increasing pyrolysis temperature induces progressive grain growth accompanied by a monotonic reduction in GB density (Supplementary Fig. 20 and Tab. 3). Samples pyrolyzed at ≤450 °C were excluded from quantitative EBSD analysis due to insufficient spatial resolution and indexing reliability (Supplementary Fig. 20).

UPS measurements (Supplementary Fig. 18) performed on the same set of samples reveal a systematic decrease in the Schottky barrier height derived from the work function and valence band onset with decreasing GB density (Supplementary Tab. 3). By correlating EBSD-derived microstructural parameters with UPS-derived electronic structure metrics, we establish a quantitative structure–barrier relationship across the temperature series.

It should be noted that UPS-derived barrier values reflect the interfacial electronic structure under pristine surface conditions and are used here to establish inter-sample trends. In contrast, the barrier modulation discussed in the sensing operation corresponds to the effective operating-state barrier of the Au–ZnO junction under gas exposure. While absolute barrier values under these conditions are not expected to coincide, they provide complementary insights into the structure–property relationship governing the sensing mechanism.

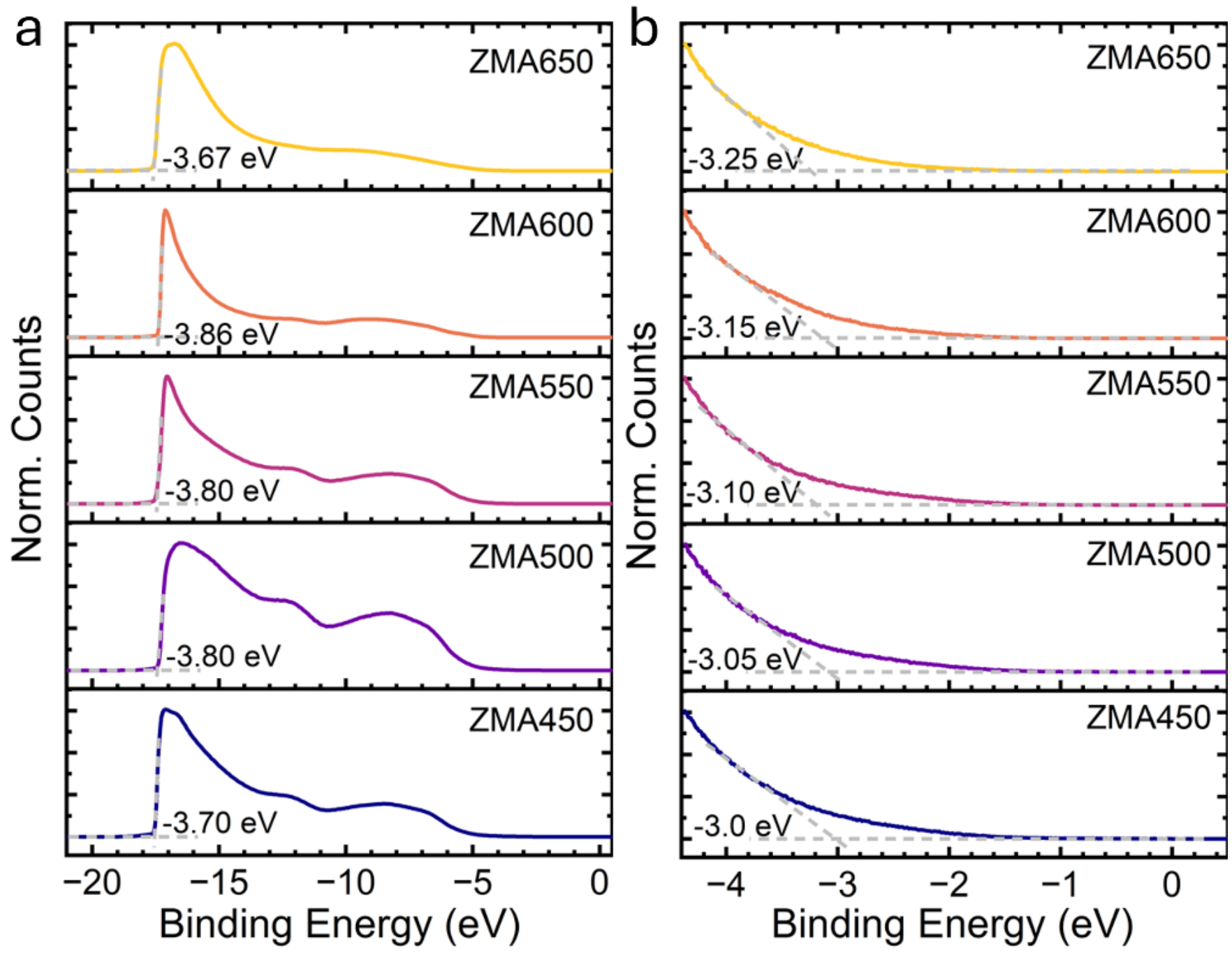

Supplementary Fig. 18 | a, UPS spectra in the secondary electron cutoff region for work-function determination (by linear extrapolation of the cutoff edge). **b,** UPS valence-band spectra showing the valence-band onset for samples pyrolyzed at 450-650 °C after H_2S sensing tests.

Supplementary Tab. 2 | Comparison of barrier parameters for samples pyrolyzed at 450-650 °C after H_2S sensing tests.

Samples	φ_{Au} (eV)	φ_{ZnO} (eV)	VB_{onset} (eV)	Barrier (eV)
ZMA 450		3.70	3.00	1.07
ZMA 500		3.80	3.05	1.02
ZMA 550	4.7	3.80	3.10	0.97
ZMA 600		3.86	3.15	0.92
ZMA 650		3.67	3.25	0.82

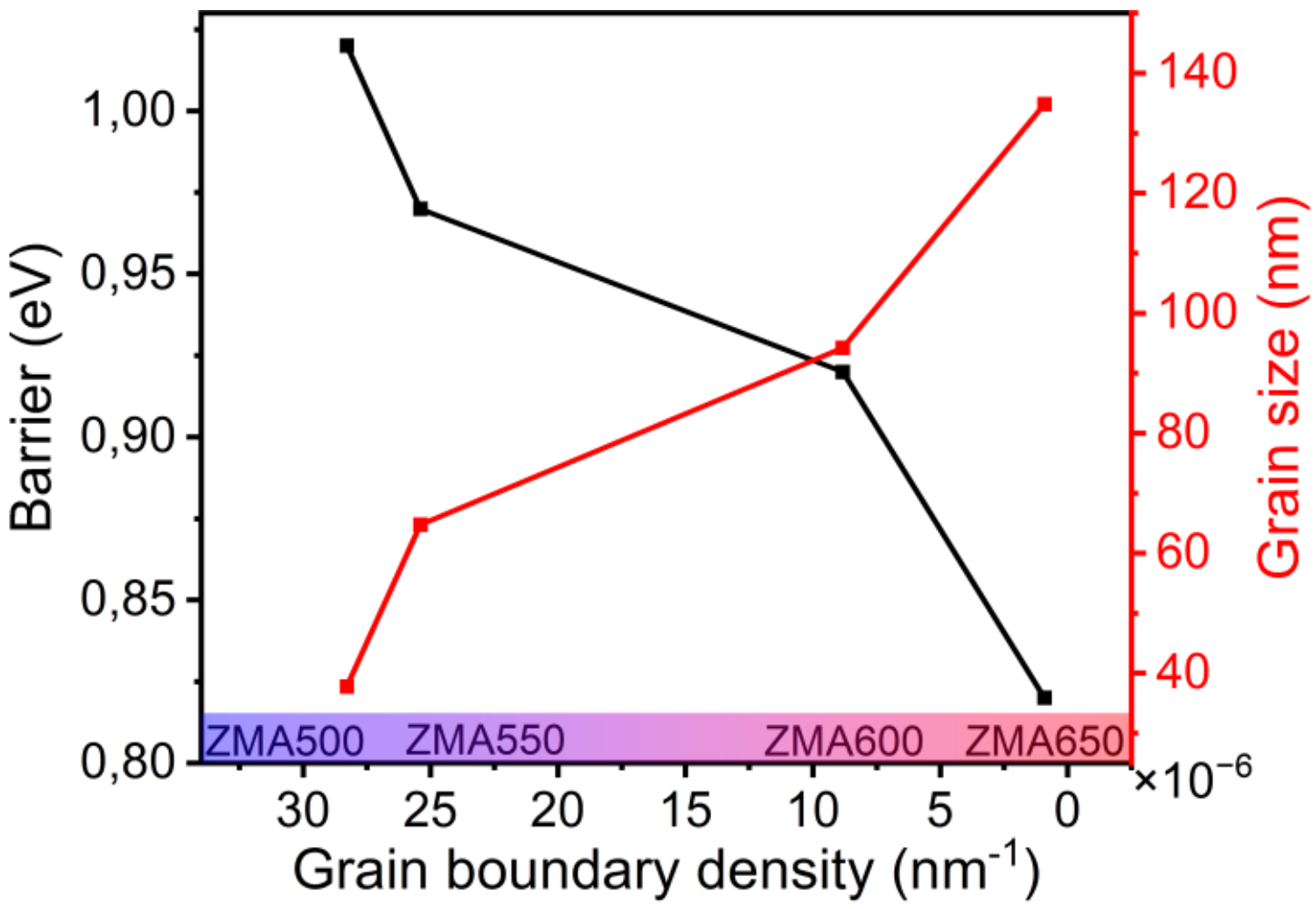

Supplementary Fig. 19 | Grain size and grain boundary density extracted from EBSD, and Schottky barrier height derived from UPS for samples pyrolyzed at 500–650 °C.

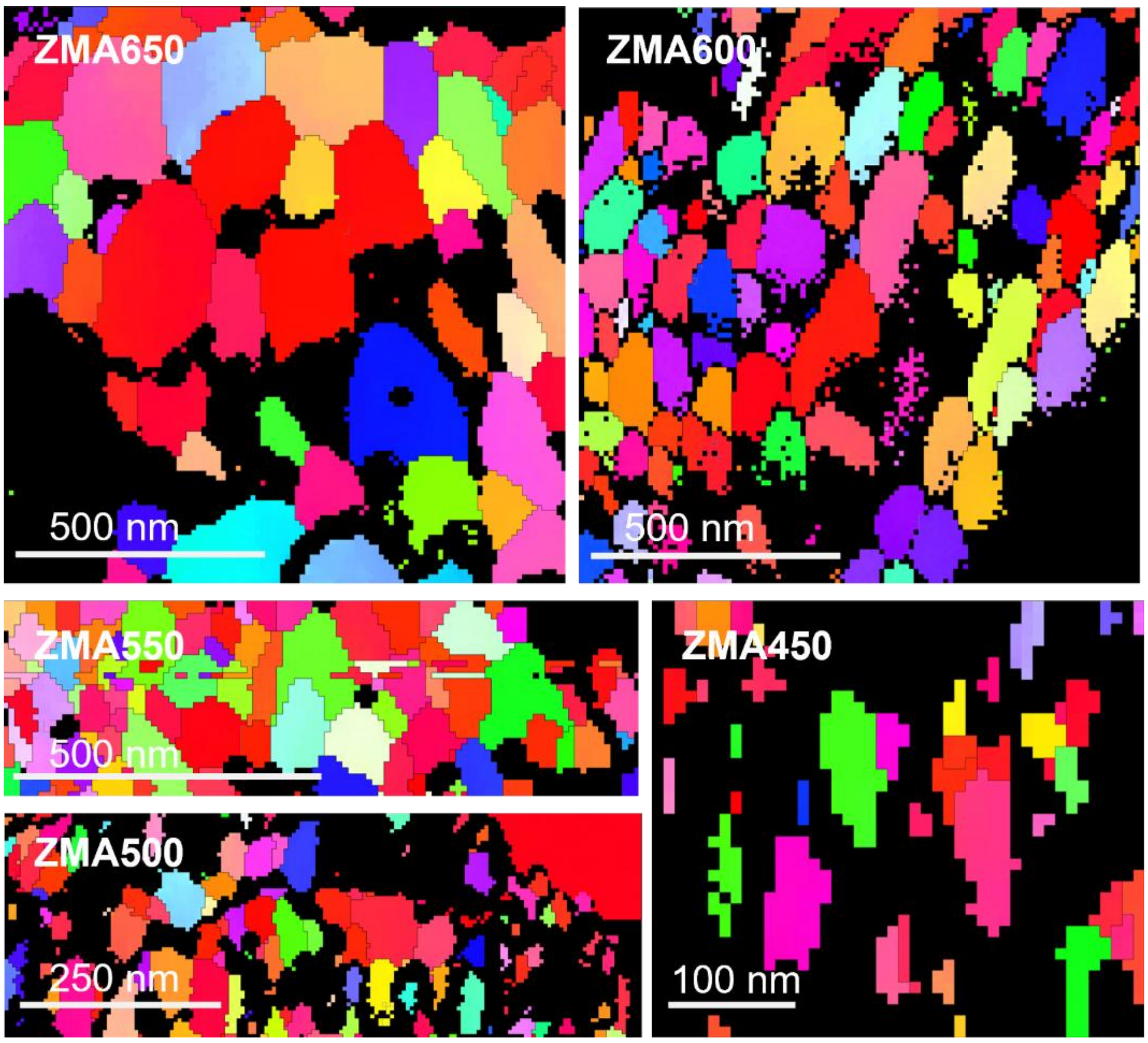

Supplementary Fig. 20 | EBSD images of samples pyrolyzed at 450–650 °C.

Supplementary Tab. 3 | Grain boundary concentration and density of samples pyrolyzed at 500–650 °C.

Samples	Total length of GB/nm	Area /nm^2	GB density/nm^{-1}
ZMA 500	6.08148	215024.9500	2.82827×10^{-5}
ZMA 550	10.14060	399185.0975	2.54033×10^{-5}
ZMA 600	11.96009	1355545.3960	8.82308×10^{-6}

ZMA 650	11.50177	12590887.6200	9.13500×10^{-7}

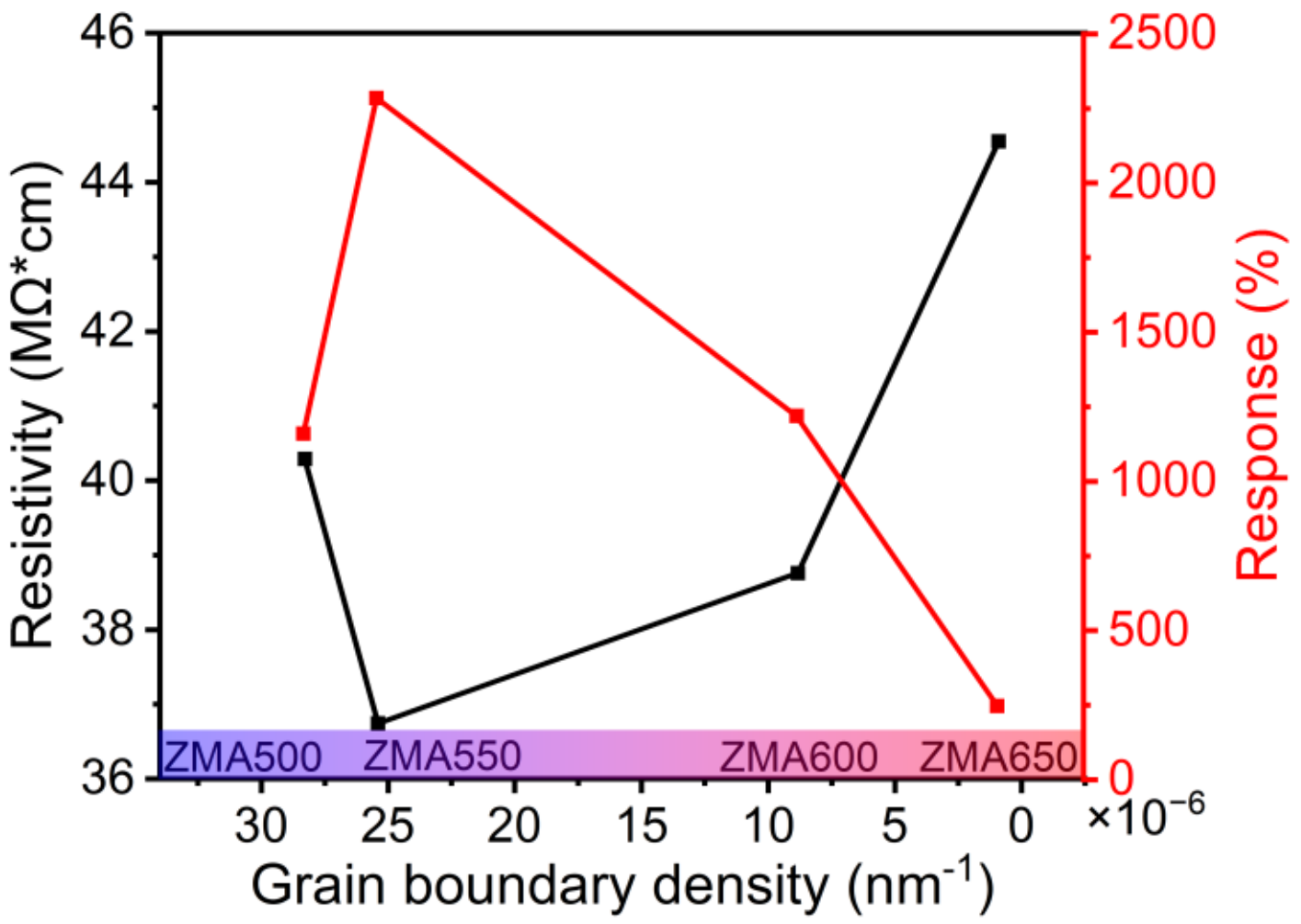

Supplementary Fig. 21 | Correlation between grain-boundary density, electrical resistivity, and H_2S sensing response.

Comment 2: The authors should supplement the study with data on the dynamic evolution of electronic structures during H_2S adsorption. It is recommended to combine the DFT simulations in the supplementary materials with new in situ characterization experiments, such as in situ Raman spectroscopy, in situ FT-IR spectroscopy, or in situ impedance spectroscopy, to bridge the gap between static characterization and dynamic performance evidence.

Reply: We sincerely thank the reviewer for this important suggestion regarding the dynamic evolution of electronic structures during H_2S adsorption and the need to bridge static characterization with sensing performance.

In response, we performed in situ FT-IR measurements to directly track the surface chemical evolution of ZnO during H_2S exposure. Notably, no pronounced S-H stretching vibration was observed in the 2550-2600 cm^{-1} region, which is consistent with previous reports that H_2S preferentially undergoes dissociative adsorption on ZnO surfaces, forming strongly coordinated sulfur-containing species (e.g., HS/S or Zn-S(H)/Zn-S^- complexes) whose characteristic vibrations are located in the low-wavenumber region (<500 cm^{-1}). Therefore, the absence of S-H stretching does not preclude the presence of Zn-S(H)-related surface intermediates.

Meanwhile, several surface-related vibrational features, including the terminal/weakly hydrogen-bonded Zn-OH band at ~3680 cm^{-1}, hydrogen-bond-associated bands near ~3000 cm^{-1}, and paired bands at

~1400 and ~890 cm^{-1} assigned to carbonate/bicarbonate species, exhibit pronounced and reversible intensity changes during H_2S on/off cycles (Supplementary Fig. 30). These reversible spectral responses indicate that H_2S exposure dynamically modulates surface hydroxyl groups, acid-base sites, and adsorption equilibria, rather than inducing irreversible sulfidation.

In addition, a broad absorption band spanning 400-600 cm^{-1} with an inflection near ~520 cm^{-1} is assigned to Zn-O lattice vibrations. A reversible band at ~1060 cm^{-1} is observed upon H_2S exposure/removal, which is attributed to S-O stretching of surface Zn-SO_x species formed during the sensing process. Importantly, post-sensing TEM, EDX, XPS, and PXRD analyses do not reveal the formation of ZnS secondary phases (Supplementary Fig. 12), confirming that the sensing process is dominated by reversible surface coordination/adsorption rather than bulk-phase transformation. These in situ observations provide direct dynamic evidence that H_2S adsorption induces reversible surface electronic and chemical state modulation, which underpins the barrier-tunable sensing behavior. The DFT calculations, presented in the Supplementary Information, are used to rationalize the energetics and electronic structure perturbations associated with the grain boundary density-dependent variations in the Schottky barrier height during H_2S exposure, associated with the Zn-S(H)-related adsorption configurations inferred from the in situ FT-IR results, thereby linking the experimentally observed surface chemistry with the electronic structure modulation responsible for the sensing response.

Additionally, we performed detailed DFT calculations to compare the contributions of oxygen vacancies and zinc vacancies to H_2S adsorption and the associated defect energetics. DFT calculations show that oxygen vacancies have a significantly higher H_2S adsorption energy (-0.3096 eV) compared to zinc vacancies (-0.1304 eV), confirming that oxygen vacancies are the primary adsorption sites for H_2S. These findings suggest that oxygen vacancies play a key role in the adsorption mechanism, and are likely the most active defect sites for H_2S interaction. In comparison, the zinc vacancies, despite their presence, contribute less to H_2S adsorption. Additionally, we further confirmed the charge distribution upon H_2S adsorption at the oxygen and zinc vacancy sites by calculating the differential charge density. To quantify this, we performed Bader charge calculations, which clearly show the amount of charge transferred from the H_2S molecule to the material. Specifically, we found that when H_2S adsorbs at the oxygen vacancy, a charge of -0.1658 is transferred to the material, while at the zinc vacancy, the transferred charge is -0.1370. This further supports the dominant role of oxygen vacancies in the adsorption and reaction processes.

These new results and the associated discussion have been added to the Supplementary Information (Supplementary Fig. 30-33 and Tab. 5-6) and explicitly referenced in the revised manuscript (Page 14, Line 429-453) and are also highlighted in red accordingly, thereby strengthening the experimental basis of the proposed grain-boundary/Schottky-barrier synergistic mechanism.

Revised parts of manuscript are as following:

In situ FTIR measurements provide experimental support for this surface reaction pathway, revealing reversible perturbations of surface hydroxyl groups, acid-base sites, and Zn-O-related lattice vibrations during H_2S adsorption, consistent with the formation of sulfur-containing surface intermediates rather than irreversible sulfidation (Supplementary Fig. 30). The DFT calculations show that the dissociative adsorption of H_2S results in significant electron transfer to the ZnO surface, leading to a reduction of the interfacial barrier (Details discussion can be found in (Supplementary Fig. 31). These findings are consistent with the in situ FT-IR measurements, where surface hydroxyl groups and Zn-O vibrations show dynamic shifts during H_2S exposure and removal. Together, the DFT results and in situ FT-IR data establish a coherent understanding of how H_2S adsorption modulates the electronic structure and surface chemistry of ZnO, confirming the reversible nature of the sensing process and its dependence on surface coordination dynamics rather than irreversible chemical transformation.

To further investigate the influence of defects on sensing performance, we performed DFT calculations to evaluate the adsorption of H_2S at both oxygen and zinc vacancy sites. The results confirm that oxygen vacancies play a more significant role in H_2S adsorption, and full data and analysis are given in Supplementary Fig. 32 and Tab. 5-6. Additionally, the electron paramagnetic resonance spectroscopy (EPR) measurements were performed to directly assess the oxygen-vacancy concentration trends in the samples ($g \approx 2.003$-2.004) and reveal a monotonic decrease in signal intensity with increasing annealing temperature, and full data and analysis are given in Supplementary Fig. 33.

Revised parts of SI are as following:

In situ Fourier-transform infrared (FT-IR) spectroscopy was employed to probe the dynamic surface chemical evolution of ZnO during H_2S exposure, aiming to bridge static electronic structure characterization with real-time sensing behavior.

During H_2S exposure, no pronounced S-H stretching vibration was observed in the 2550-2600 cm^{-1} region. This observation is consistent with previous studies reporting that H_2S preferentially undergoes dissociative adsorption on ZnO surfaces, forming strongly coordinated sulfur-containing species (e.g., HS

or S) bound to surface Zn sites. The characteristic vibrational modes of such Zn-S(H)-related intermediates are typically located in the low-wavenumber region (<500 cm^{-1}), rendering the S-H stretching mode weak or undetectable in the present measurements. Therefore, the absence of an S-H band does not preclude the formation of Zn-S(H)-type surface intermediates.

Several surface-related vibrational features exhibit pronounced and reversible intensity changes during H_2S on/off cycles (Supplementary Fig. 30 right). Specifically, the band at ~3680 cm^{-1}, assigned to terminal or weakly hydrogen-bonded Zn-OH groups, together with hydrogen-bond-associated bands near ~3000 cm^{-1}, shows reversible attenuation and recovery upon H_2S exposure and removal. In addition, paired bands at ~1400 and ~890 cm^{-1}, characteristic of surface carbonate/bicarbonate species, display correlated reversible variations [41-44]. These observations indicate that H_2S adsorption dynamically perturbs surface hydroxyl groups, acid-base sites, and adsorption equilibria on ZnO, rather than inducing permanent chemical transformation. The reversibility of these spectral changes strongly suggests that the sensing process is governed by surface coordination and adsorption-desorption dynamics instead of irreversible sulfidation.

In the low-wavenumber region, a broad absorption band spanning 400-600 cm^{-1} with an inflection near ~520 cm^{-1} is assigned to Zn-O lattice vibrations. A reversible band at ~1060 cm^{-1} is observed upon H_2S exposure/removal, which is attributed to S-O stretching of surface Zn-SO_x species formed during the sensing process (Supplementary Fig. 30 left) [41-42, 45]. The modulation of these features during H_2S exposure reflects surface-sensitive perturbations of the Zn-O framework associated with adsorption-induced electronic and structural rearrangements. Importantly, post-sensing TEM, EDX, XPS, and PXRD analyses do not reveal the formation of ZnS secondary phases (Supplementary Fig. 12), confirming that the interaction between H_2S and ZnO is confined to reversible surface coordination rather than bulk-phase sulfidation.

Taken together, the in situ FT-IR results provide direct evidence that H_2S adsorption induces reversible surface chemical and coordination changes on ZnO, dynamically modulating surface hydroxyls, acid-base equilibria, and Zn-O-related lattice environments. These surface processes are inherently coupled to electronic structure reconfiguration, thereby providing dynamic experimental support for the barrier-tunable sensing mechanism inferred from static electronic structure analysis.

Density functional theory (DFT) calculations were carried out to rationalize the energetics and electronic structure perturbations associated with H_2S-derived surface intermediates inferred from the in situ FT-IR observations.

Representative adsorption configurations of H_2S and its dissociation products (HS and S) on the ZnO surface were considered. The calculated adsorption energies indicate that dissociative adsorption configurations involving Zn-S(H)-type coordination are energetically more favorable than molecularly adsorbed H_2S, consistent with the absence of a detectable S-H stretching vibration in the in situ FT-IR spectra.

Charge density difference and Bader charge analyses reveal noticeable electron transfer from the adsorbed sulfur-containing species to the ZnO surface, resulting in increased electron density localized near surface Zn sites. This charge redistribution is consistent with the experimentally observed upward shift of the Fermi level and reduction of the interfacial barrier upon H_2S exposure.

The DFT results provide electronic structure and energetic support for the surface intermediates and adsorption configurations inferred from in situ FT-IR measurements. Together, these complementary experimental and theoretical results establish a coherent picture in which dissociative H_2S adsorption and reversible Zn-S(H)-type surface coordination dynamically modulate the electronic structure of ZnO, underpinning the observed barrier-tunable sensing behavior.

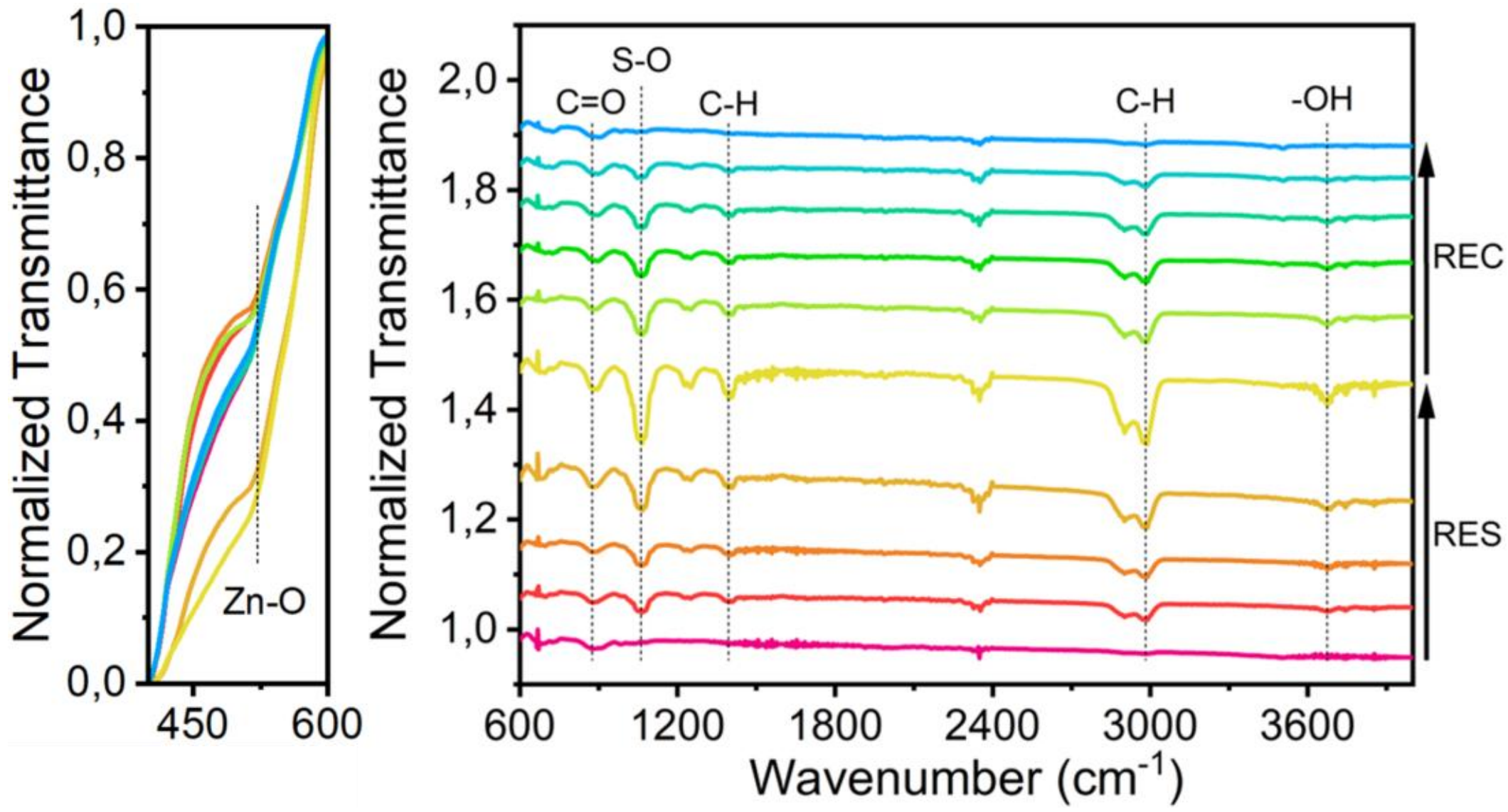

Supplementary Fig. 30 | In situ FTIR spectra of ZMA550 during H_2S adsorption and desorption.

Comment 3. Since the authors emphasize the sensor's monitoring capability in real-world conditions, evaluating the impact of humidity variations on performance is crucial. In practical applications, humidity fluctuations can significantly interfere with the response of metal oxide sensors. It is recommended to add humidity gradient experiments (e.g., 15%, 30%, 50%, 70% RH) to test the ZMA550's response values to 1 ppm H_2S at different humidity levels. Combine this with XPS measurements of surface hydroxyl content

changes to analyze the mechanism by which humidity affects performance through "competitive adsorption" or "electron transfer interference."

Reply: We sincerely thank the reviewer for highlighting the importance of humidity effects for evaluating the sensor performance under realistic operating conditions.

In response, we conducted systematic humidity-dependent sensing measurements by exposing the ZMA550 sensor to 1 ppm H_2S under controlled relative humidity levels of 15%, 30%, 50%, 70%, and 90% RH (Supplementary Fig. 9). The humidity was continuously monitored using a calibrated Bosch BME280 sensor, and a schematic of the humidity-controlled sensing setup has been added (Supplementary Fig. 8). The response-recovery curves demonstrate that the sensor maintains a clear and reversible response to H_2S across the entire humidity range, indicating robust sensing behavior under varying ambient humidity conditions.

To elucidate the underlying mechanism, XPS measurements were performed on the sensing layers after operation at different humidity levels, with particular focus on the O 1s region (Supplementary Fig. 10). In the XPS analysis, with increasing humidity, we observe no significant change in the lattice oxygen (O_I) peak, while the intensity of the hydroxyl-related oxygen species (O_{III}) peak increases. This indicates that water vapor primarily influences the formation of hydroxyl-related oxygen species without significantly altering lattice oxygen content or the overall oxygen chemical states of ZnO.

These results suggest that the influence of humidity on sensor performance is limited and does not originate from strong competitive adsorption or pronounced electron transfer interference associated with surface hydroxyl accumulation. Instead, the Schottky-barrier-modulated sensing mechanism remains largely intact under humid conditions, supporting the applicability of the sensor for real-world monitoring scenarios.

These new results and the associated discussion have been added to the Supplementary Information (Supplementary Fig. 8-11) and explicitly referenced in the revised manuscript (Page 8, Line 241-243) and are also highlighted in red accordingly.

Revised parts of manuscript are as following:

The sensor maintains a stable and reversible response to H_2S across a wide humidity range (15-90% RH) (Supplementary Fig. 8-10) and representative temperatures from -10 to 40 °C (Supplementary Fig. 11), demonstrating robust performance under realistic conditions.

Revised parts of SI are as following:

The humidity was continuously monitored using a calibrated Bosch BME280 sensor, and a schematic of the humidity-controlled sensing setup has been added (Supplementary Fig. 8). It is worth noting that the response amplitude of the sensor to 1 ppm H_2S exhibits a gradual increase with increasing relative humidity, as consistently observed over repeated response-recovery cycles at each humidity level (Supplementary Fig. 9). This trend suggests that moderate humidity may facilitate the surface reaction kinetics or charge transfer processes involved in H_2S sensing.

To elucidate the underlying mechanism, XPS measurements were performed on the sensing layers after operation at different humidity levels, with particular focus on the O 1s region (Supplementary Fig. 10). The analysis reveals no significant change in the lattice oxygen (O_I) peak, while the intensity of the hydroxyl-related oxygen species (O_{III}) peak increases with humidity. This indicates that water vapor primarily influences the formation of hydroxyl-related oxygen species on the surface, rather than substantial modification of the lattice oxygen or irreversible water-induced surface reconstruction. Thus, humidity modulates the sensing response through surface hydroxylation, without affecting the core Schottky-barrier-modulated sensing mechanism.

In the pristine samples, the O 1s envelope was deconvoluted into lattice oxygen (O_I, ~530.0 eV), oxygen vacancies (O_{II}, ~531.1 eV), and loosely bound surface oxygen species (O_{III}, ~532.2 eV) (Supplementary Fig. 28b and c). Following sensing tests under varying humidity conditions, the O 1s spectrum was dominated by O_I (lattice oxygen, ~530.9 eV) and O_{III} (hydroxyl/adsorbed water-related oxygen species, ~532.9 eV). The emergence of O_{III} correlates with humidity-induced surface hydroxylation and the formation of H_2O-related species, whereas the relative intensity of the oxygen vacancy-related signal (O_{II}) is not explicitly observed due to the overlap with hydroxylated oxygen species. Importantly, these changes do not diminish the central role of oxygen vacancies in the sensing mechanism. Oxygen vacancies remain the primary reactive sites for H_2S adsorption, and the increased O_{III} component highlights the surface modification that occurs in the presence of humidity.

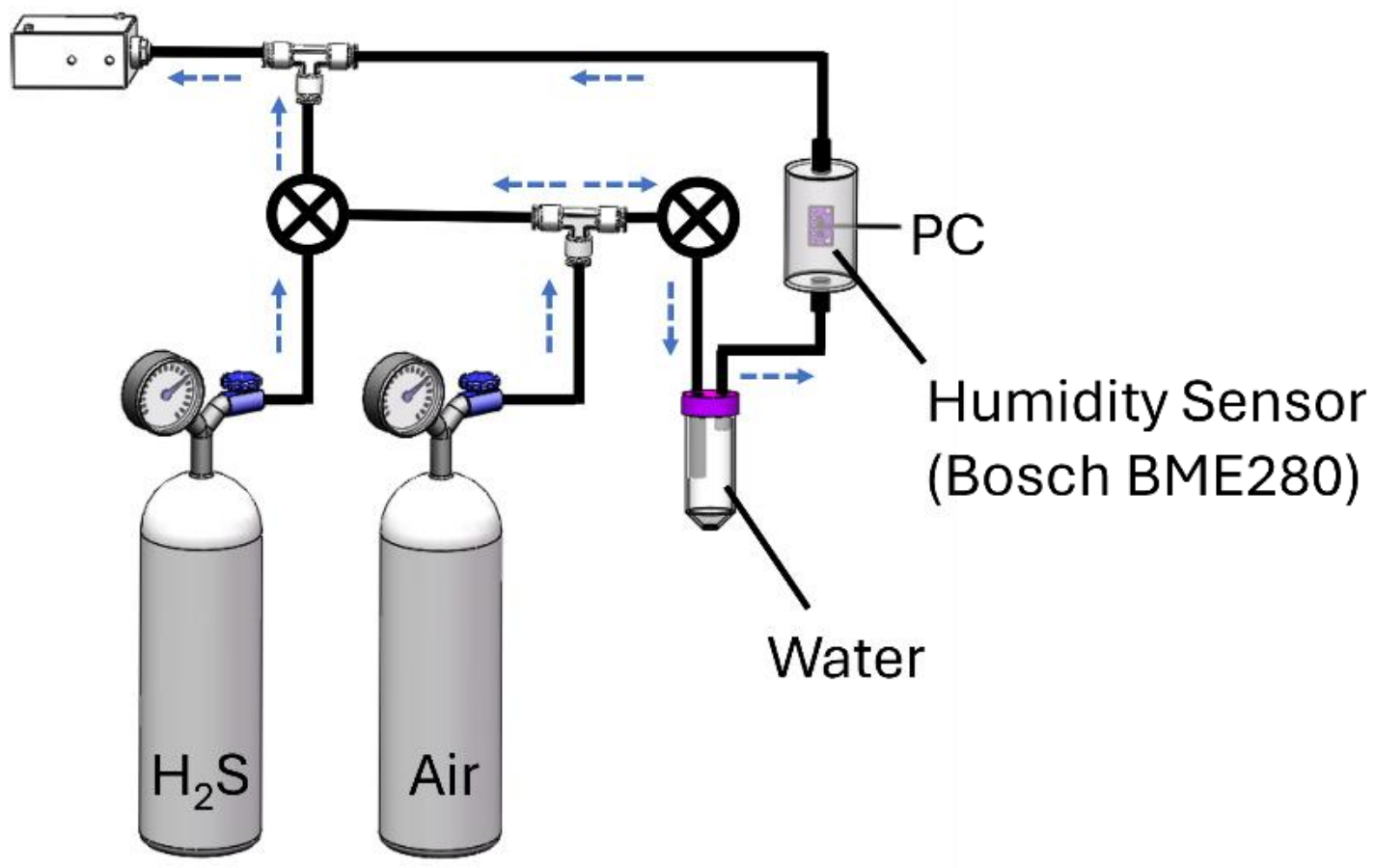

Supplementary Fig. 8 | Schematic diagram of humidity introduction in sensing tests.

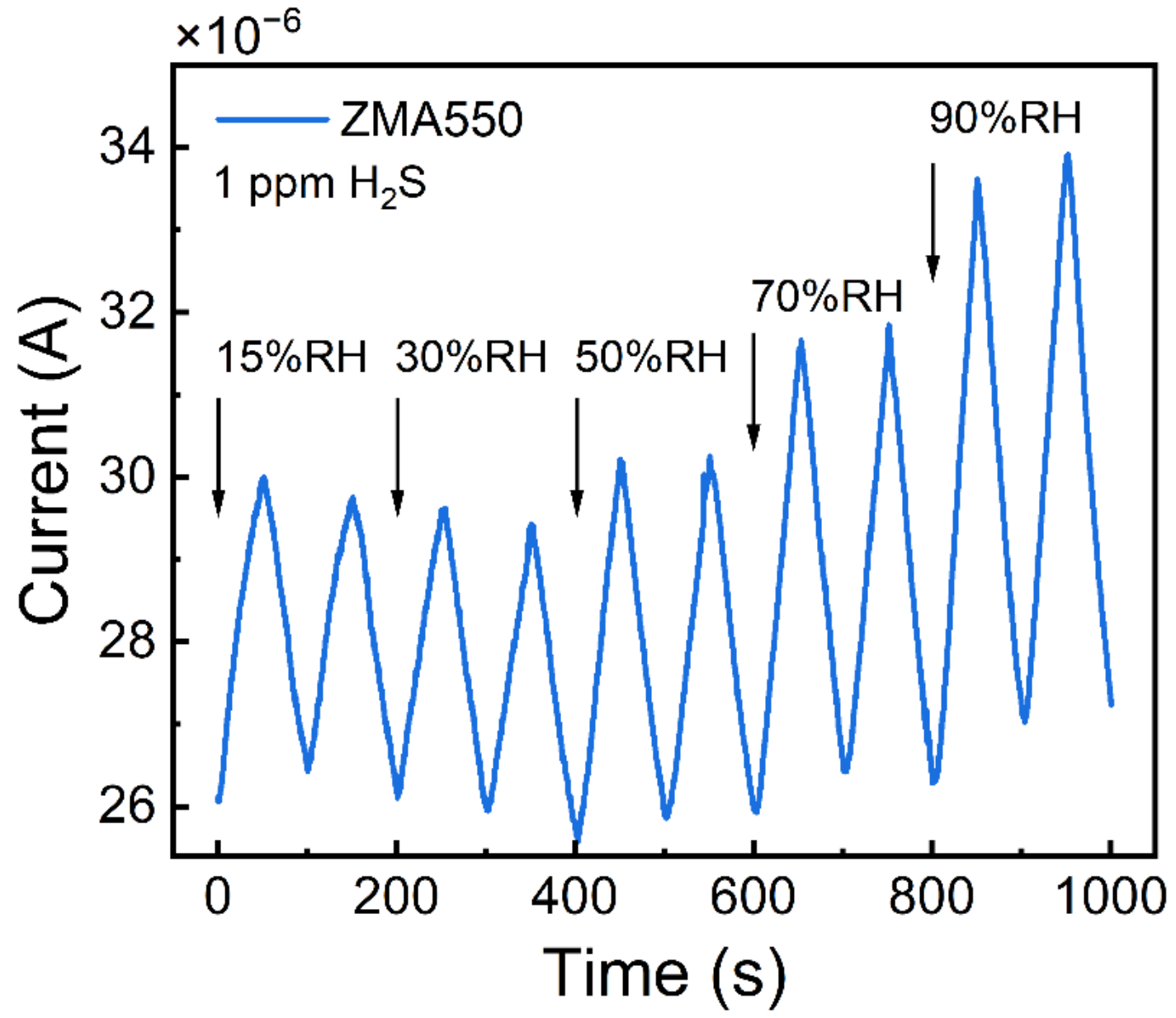

Supplementary Fig. 9 | Current response and recovery curves of ZMA550 sensor towards 1 ppm H_2S at different humidity levels from 15%RH-90%RH.

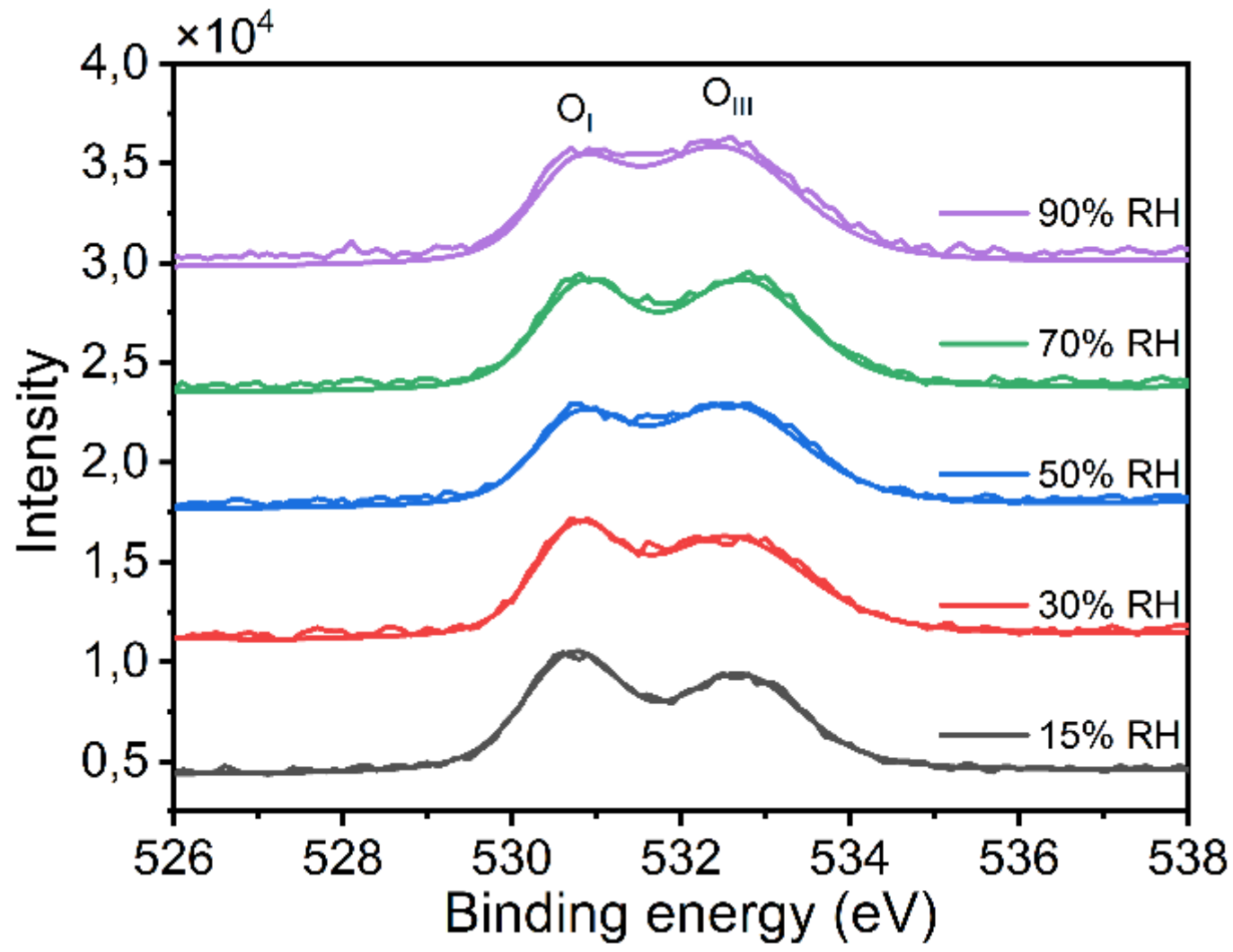

Supplementary Fig. 10 | O 1s XPS results of sensing tests under different humidity levels.

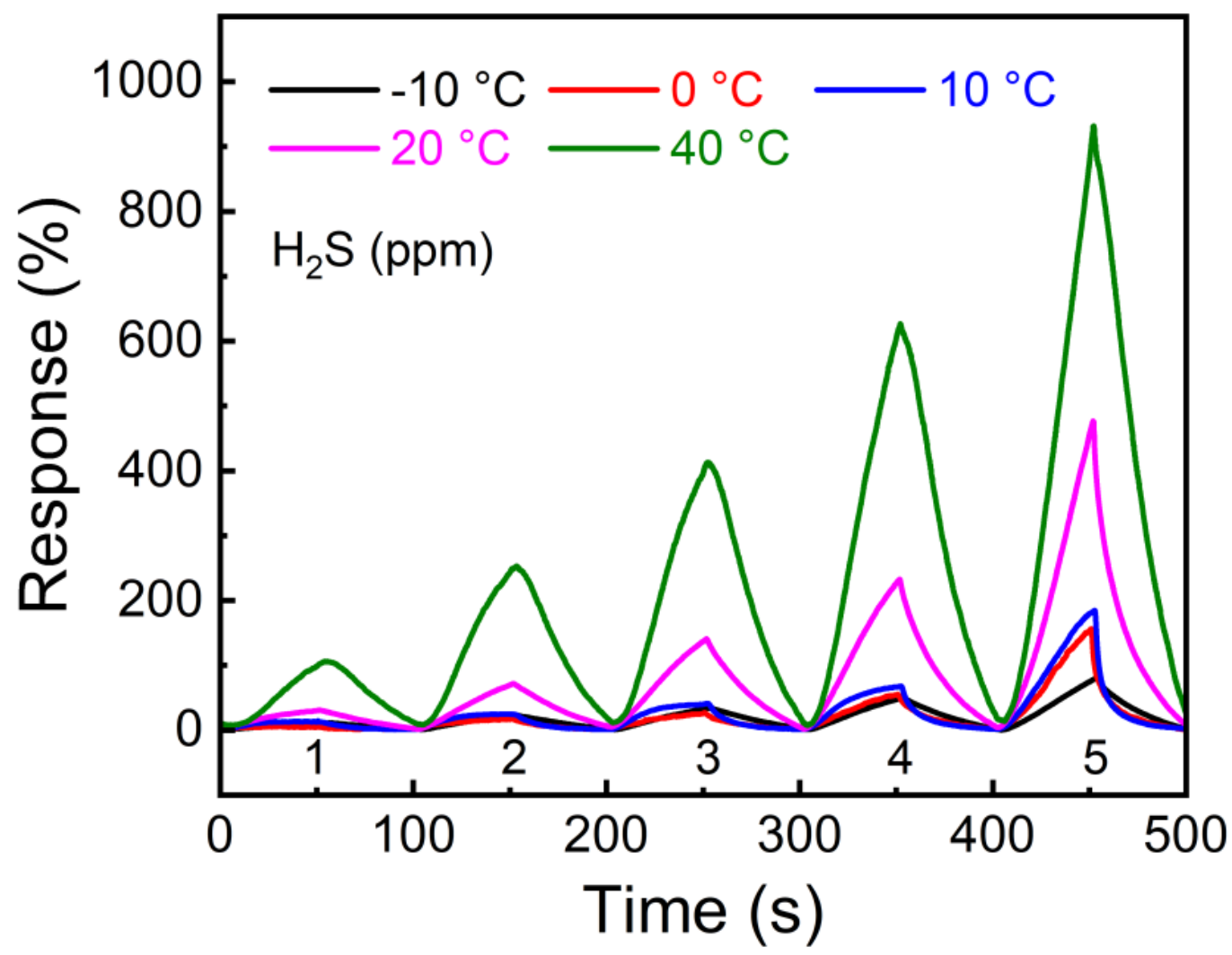

Supplementary Fig. 11 | Response and recovery curves of ZMA550 sensor exposed to varying high H_2S concentrations (1-5 ppm) under different operation temperature.

Comment 4. Nanomaterials based on metal-organic frameworks (MOFs) are frequently employed for H_2S gas sensors; the structural design of the materials in this work is not novel.

Reply: We sincerely thank the reviewer for this comment regarding the structural novelty of MOF-derived nanomaterials for H_2S sensing. We agree that MOF-based and MOF-derived nanomaterials have been extensively explored for gas sensing applications, particularly for H_2S detection, and the structural motif itself is not the primary novelty of this work.

The key innovation of this study does not lie in introducing a new MOF-derived architecture, but rather in establishing a fundamentally different structure-property-function control paradigm. Specifically, we

demonstrate that the intrinsic temperature sensitivity of MOF-derived ZnO can be exploited as a precise and scalable physical parameter to regulate grain size and grain boundary density, which in turn governs both the initial Schottky barrier height and its dynamic modulation upon gas adsorption.

By systematically correlating grain boundary density, interfacial barrier characteristics, and sensing performance, this work reveals that an optimized intermediate grain boundary density—rather than extreme grain refinement or grain growth—maximizes the barrier modulation efficiency and charge transport regulation. This grain-boundary-assisted Schottky barrier tuning enables record-level H_2S sensing performance and ultra-low power operation, without relying on complex material compositions or hierarchical structural designs.

Therefore, the novelty of this work resides in the mechanism-driven performance optimization strategy, which is generalizable beyond the specific MOF precursor used here and provides a transferable framework for designing next-generation low-power gas sensors.

These further discussions have been added to the revised manuscript (Page 3, Line 101-109) and are also highlighted in red accordingly.

Revised parts of manuscript are as following:

While MOF-derived metal oxides have been widely explored for H_2S sensing, most previous studies primarily focus on material composition optimization or morphological engineering. In contrast, the novelty of this work does not lie in the use of MOF-derived ZnO itself, but in identifying grain boundary density as a decisive and tunable physical parameter that governs both the initial Schottky barrier and its dynamic modulation during gas adsorption. By leveraging the temperature-sensitive microstructural evolution inherent to MOF-derived ZnO, we establish a quantitative correlation between grain boundary density, barrier modulation efficiency, and sensing performance. This mechanism-oriented strategy enables simultaneous enhancement of sensitivity and power efficiency without relying on complex structural designs, offering a generalizable pathway for high-performance H_2S sensing.

Comment 5. The introduction does not elaborate on how the current limitations of sensors specifically impact monitoring requirements in practical application scenarios, such as anaerobic environments for food preservation or industrial enclosed spaces.

Reply: We sincerely thank the reviewer for this valuable comment. In the revised Introduction, we have explicitly elaborated on how the limitations of conventional H_2S sensors—such as high operating

temperature, elevated power consumption, and dependence on oxygen-assisted surface reactions—directly constrain monitoring in practical scenarios, including anaerobic food preservation systems and enclosed industrial environments. This added discussion clarifies the application-driven motivation of this work and highlights how the proposed grain-boundary-assisted Schottky barrier modulation strategy directly addresses these real-world monitoring requirements.

These further discussions have been added to the revised manuscript (Page 2, Line 60-73) and are also highlighted in red accordingly.

Revised parts of manuscript are as following:

In practical application scenarios, such as anaerobic food preservation systems and enclosed industrial environments (e.g., storage tanks, pipelines, and confined workspaces), the monitoring requirements for H_2S are particularly stringent [39-40]. In anaerobic or low-oxygen food preservation conditions, trace-level H_2S released during early-stage spoilage must be detected under high humidity and limited oxygen availability, where conventional metal-oxide sensors often exhibit sluggish response, poor reversibility, and elevated power consumption. In these settings, the elevated operating temperatures commonly required by traditional metal-oxide sensors not only increase power consumption but also pose safety risks and limit long-term, battery-powered deployment. Moreover, the reliance on oxygen-assisted surface reactions and unstable interfacial charge transport frequently leads to signal drift and reduced reliability under low-oxygen or confined conditions [41-43]. These practical constraints highlight the need for H_2S sensors that meet stringent real-world monitoring requirements, including room-temperature operation, ultra-low power consumption, and stable performance under low-oxygen and confined conditions. Addressing these application-driven challenges requires a sensing mechanism that fundamentally decouples performance from thermal activation and instead relies on robust interfacial electronic modulation.

Comment 6. Given the focus of this study, the authors have not addressed the necessity of two core innovative strategies in the introduction: regulating grain boundary density within metal-organic framework derivative materials and modulating the Schottky barrier. It is recommended that the introduction be supplemented with existing research highlighting the advantages of metal-organic framework derivative materials in terms of controllable microstructure and high specific surface area. Alternatively, an explanation could be provided on how the Schottky interface achieves high selectivity/high sensitivity in gas sensing. This approach would both prevent the novel points from appearing abrupt and help readers swiftly grasp the

core logic of the research design, thereby paving the way for a more natural transition to the technical details in the subsequent manuscript.

Reply: We sincerely thank the reviewer for this insightful suggestion. In the revised Introduction, we have explicitly highlighted the two core strategies of this work—grain boundary density regulation in MOF-derived materials and Schottky barrier modulation—and provided contextual discussion on why these approaches are advantageous for gas sensing. Specifically, we added a paragraph summarizing prior studies on the microstructural tunability of MOF-derived metal oxides and another paragraph explaining how Schottky interfaces enhance sensitivity and selectivity via interfacial barrier modulation. These additions ensure that the novelty and rationale of the proposed design are clearly introduced and that the transition to the subsequent technical sections is more natural.

These further discussions have been added to the revised manuscript (Page 3, Line 74-91) and are also highlighted in red accordingly.

Revised parts of manuscript are as following:

Metal-organic framework (MOF)-derived metal oxides have emerged as attractive platforms for gas sensing owing to their intrinsically high specific surface area and, more importantly, the ability to tailor microstructural features through controlled thermal conversion [44-46]. Previous studies have shown that the pyrolysis of MOF precursors enables precise regulation of grain size, porosity, and grain boundary density in the resulting metal oxides, providing a versatile route to engineer charge transport pathways and surface reactivity. However, despite these advantages, the role of grain boundary density as an active and tunable parameter governing gas-sensing performance has rarely been systematically explored. In parallel, Schottky interfaces between metals and semiconducting oxides are well known to amplify gas-sensing signals by converting surface adsorption-induced electronic perturbations into exponential changes in carrier transport [47-49]. Despite these advances, the grain boundary density has rarely been treated as an active and tunable physical parameter governing Schottky barrier formation and dynamic barrier modulation during gas adsorption. As a result, how microstructural parameters interact with interfacial electronic transport to determine sensing performance remains insufficiently understood. Motivated by this gap, we identify grain boundary density as a decisive control knob that simultaneously governs the initial Schottky barrier and its modulation efficiency, enabling a mechanism-driven optimization of H_2S sensing performance.

Comment 7. How did the authors arrive at the detection limit of 0.328 ppb for the sensor? Was this obtained through actual testing or calculated?

Reply: We sincerely thank the reviewer for raising this important point regarding the determination of the detection limit. The reported limit of detection (LOD) of 0.328 ppb was not obtained from direct exposure to a calibrated sub-ppb H_2S atmosphere, but was calculated following the widely adopted analytical definition for chemiresistive gas sensors. Specifically, the LOD was estimated using the widely accepted IUPAC formula:

$$LOD = 3.3\ \delta / S$$

where δ represents the standard deviation of the baseline noise extracted from the sensor signal under air background gas at room temperature, and S denotes the sensitivity, defined as the slope of the linear fitting of the sensor response versus H_2S concentration in the low-concentration regime. Both δ and S were experimentally determined from repeated measurements, ensuring statistical reliability. This approach is commonly used in gas sensing literature to estimate the theoretical detection capability of sensors when direct sub-ppb gas calibration is not readily accessible. We have clarified this point in the revised manuscript and provided additional details in the Supplementary Information.

These new results and the associated discussion have been added to the Supplementary Information (Supplementary Fig. 6) and are also highlighted in red accordingly.

Revised parts of SI are as following:

The sensor response at each H_2S concentration was obtained from the time-resolved current curves by extracting the maximum response amplitude during H_2S exposure (Supplementary Fig. 6a). The concentration-response relationship was constructed as a log-log plot and fitted using a linear model:

$$\lg(\text{Current}) = k \cdot \lg(C) + b$$

where C is the H_2S concentration and k is the slope (sensitivity in the log-log domain) used for LOD estimation. The baseline noise (RMS) was quantified from the current-time signal recorded under air conditions. To remove slow instrumental drift without affecting high-frequency noise, the baseline was fitted using a low-order (5th-order) polynomial, and the RMS was calculated from the residuals ($Y_i - Y_{fit}$) (Supplementary Fig. 6b). This procedure serves only to isolate stochastic noise and does not involve derivative-based baseline anchoring [40]. Specifically, the LOD was estimated using the widely accepted IUPAC formula:

$$LOD = 3.3\ \delta / S$$

where δ represents the standard deviation of the baseline noise extracted from the sensor signal under air background gas at room temperature, and S denotes the sensitivity, defined as the slope of the linear fitting of the sensor response versus H_2S concentration in the low-concentration regime. This approach ensures that the LOD is intrinsic to the sensor's response to H_2S, with no dependence on arbitrary baseline correction procedures.

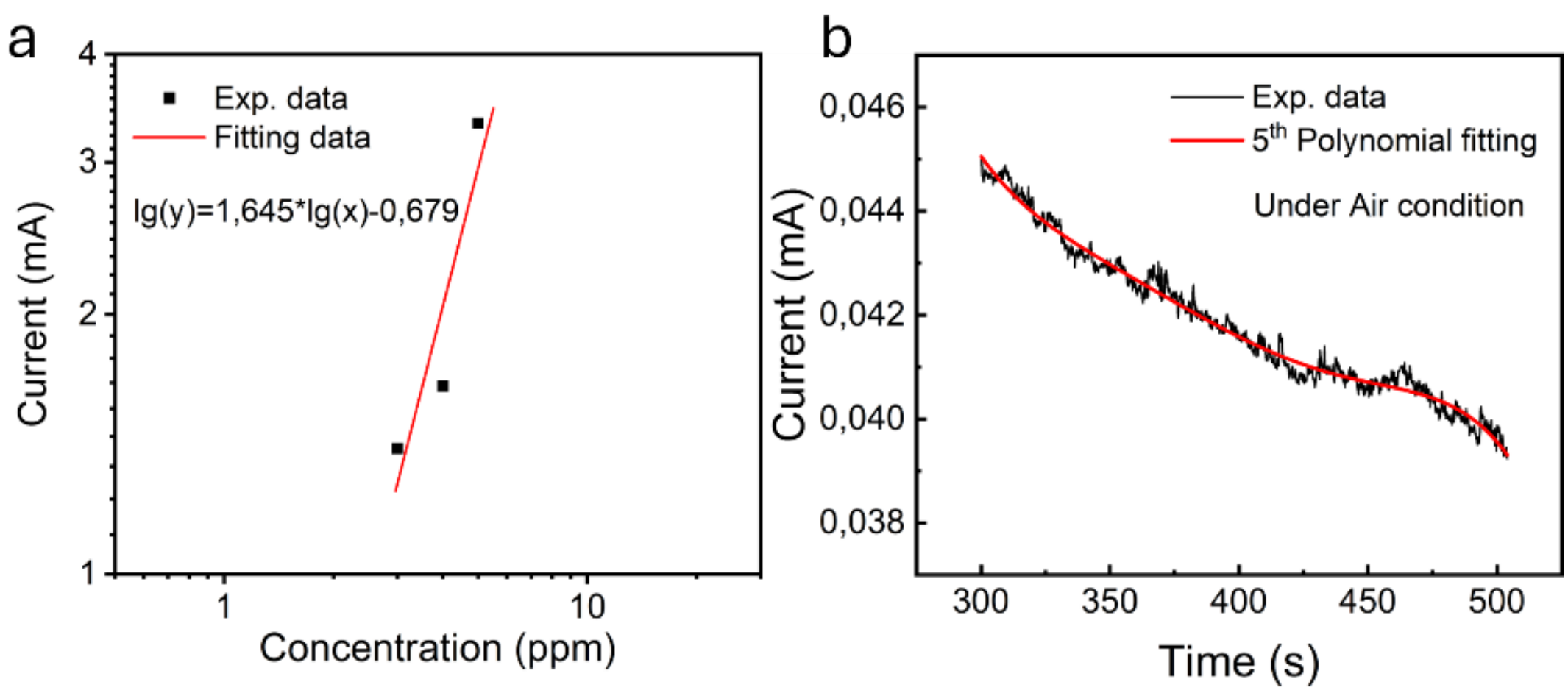

Supplementary Fig. 6 | a, Response concentration log-log plots to different concentrations of H_2S under air condition. **b,** RMS noise levels of response as a function of time under air conditions.

Comment 8. The author should supplement performance stability data under different interfering gases to enhance the completeness of the comparison.

Reply: We sincerely thank the reviewer for this valuable suggestion regarding the stability of sensor performance under interfering gases. In response, we have performed a comprehensive dynamic interference stability test by subjecting the sensor to repeated H_2S sensing cycles in the presence of various interfering gases. Specifically, the response and recovery behavior toward 3 ppm H_2S was continuously monitored over 18 successive cycles, where a different interfering gas was intentionally introduced during each individual sensing cycle.

As shown in the newly added Supplementary Fig. 13, the sensor maintains a highly stable and reproducible response to H_2S throughout all cycles, despite the presence of diverse interfering gas species. No noticeable degradation, baseline drift, or response attenuation was observed, demonstrating that the H_2S sensing performance is robust against transient interference.

To further ensure experimental transparency and reproducibility, photographs of the actual testing setup and a schematic illustration detailing the gas introduction protocol are provided in Supplementary Fig. 14-

15. These results collectively confirm the excellent anti-interference stability and operational reliability of the sensor under realistic multi gas environments. We have incorporated these data and discussions into the revised Supplementary Information.

These new results and the associated discussion have been added to the Supplementary Information (Supplementary Fig. 13-15) and explicitly referenced in the revised manuscript (Page 8, Line 250-258) and are also highlighted in red accordingly.

Revised parts of manuscript are as following:

To assess cross-interference under mixed-gas conditions relevant to practical environments, we performed dynamic co-exposure tests in which H_2S was introduced together with representative oxidizing gases (NO_2 and SO_2) and a reducing gas (NH_3), both individually and in mixtures. The sensor maintains a stable and reproducible H_2S response even when oxidizing and reducing interferents coexist simultaneously, demonstrating strong anti-interference capability in complex gas matrices (Supplementary Fig. 13-15). The robustness is particularly relevant to the boiled-egg spoilage headspace tests, where multiple VOCs (e.g., amines, organic acids, and small sulfur-containing species) may be present. The mixed-gas evaluation therefore supports that the response observed in the spoilage demonstration is primarily governed by H_2S-associated surface/interfacial modulation rather than nonspecific responses to coexisting volatiles.

Revised parts of SI are as following:

To evaluate the robustness of the H_2S sensing signal under realistic multi gas environments, we conducted a dynamic interference stability test by repeatedly exposing the sensor to 3 ppm H_2S over 18 consecutive response-recovery cycles. During each cycle, one set of interfering gases was introduced simultaneously with H_2S, while all other experimental parameters (e.g., concentration control, total flow rate, temperature, and bias/measurement settings) were kept constant. In particular, to directly address the coexistence of oxidizing and reducing species, we tested representative oxidizing gases (NO_2, SO_2) and a reducing gas (NH_3) both individually and in mixtures, including $NO_2 + SO_2$, $NO_2 + NH_3$, $SO_2 + NH_3$, and $NO_2 + SO_2 + NH_3$. The sensor exhibits highly reproducible H_2S response amplitudes with negligible baseline drift across all cycles, indicating that the sensing output is dominated by H_2S-triggered interfacial modulation rather than transient cross-interference from coexisting gases (Supplementary Fig. 13). A schematic of the gas-introduction protocol and the overall setup is provided in Supplementary Fig. 14, and a photograph of the actual experimental setup is shown in Supplementary Fig. 15 to ensure transparency and reproducibility.

In addition to the inorganic interferents above, we further evaluated potential cross-sensitivity to representative VOC interferents that are commonly reported in food spoilage headspaces, particularly during egg fermentation. These VOCs include amines (triethylamine, propylamine, butylamine), organic acids (acetic acid and butyric acid), sulfur-containing species (dimethyl sulfide), and small oxygenated molecules (formaldehyde, ethanol, and acetone). The sensor maintains stable and reversible H_2S responses under these VOC interference conditions as well (Supplementary Fig. 13), further supporting its robustness in complex gas matrices.

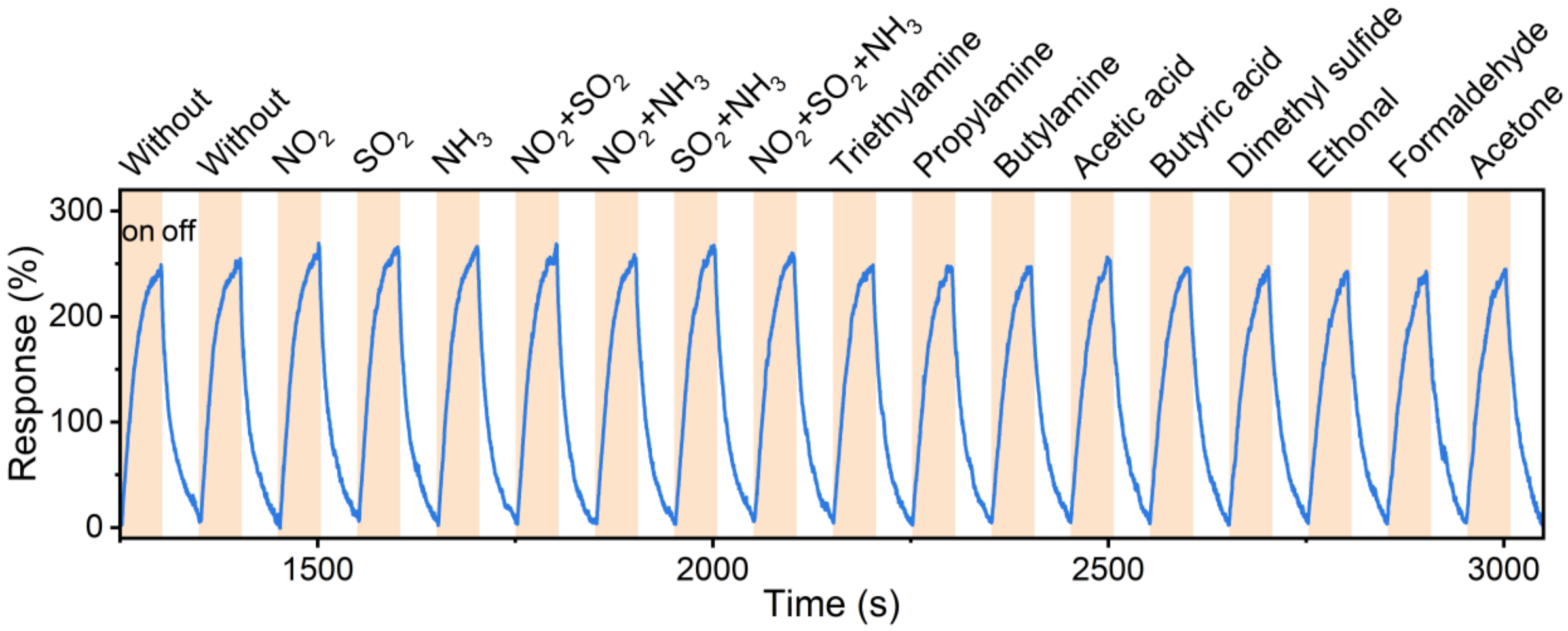

Supplementary Fig. 13 | Dynamic interference stability of the H_2S sensor.

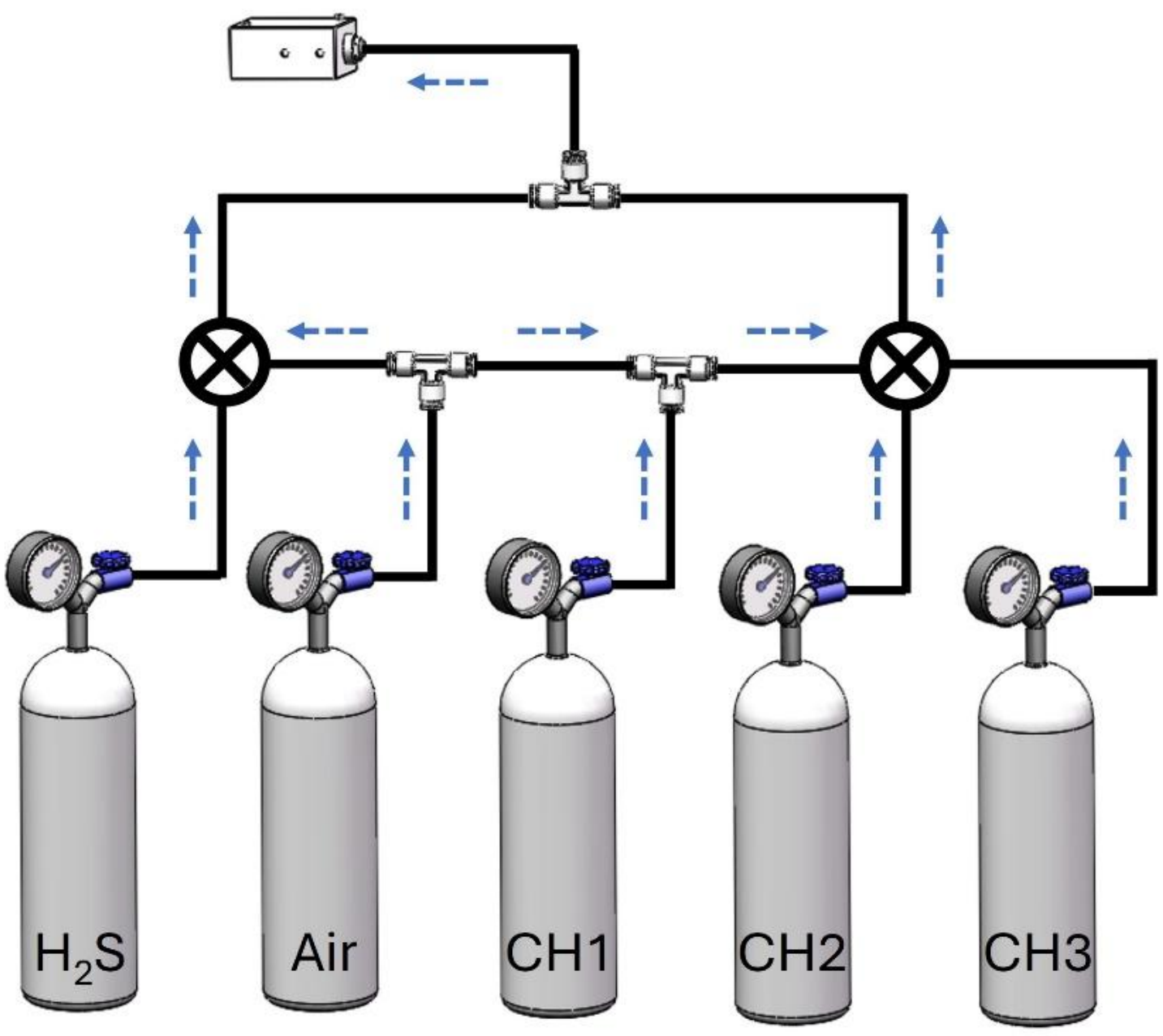

Supplementary Fig. 14 | Experimental setup for dynamic interference stability measurements.

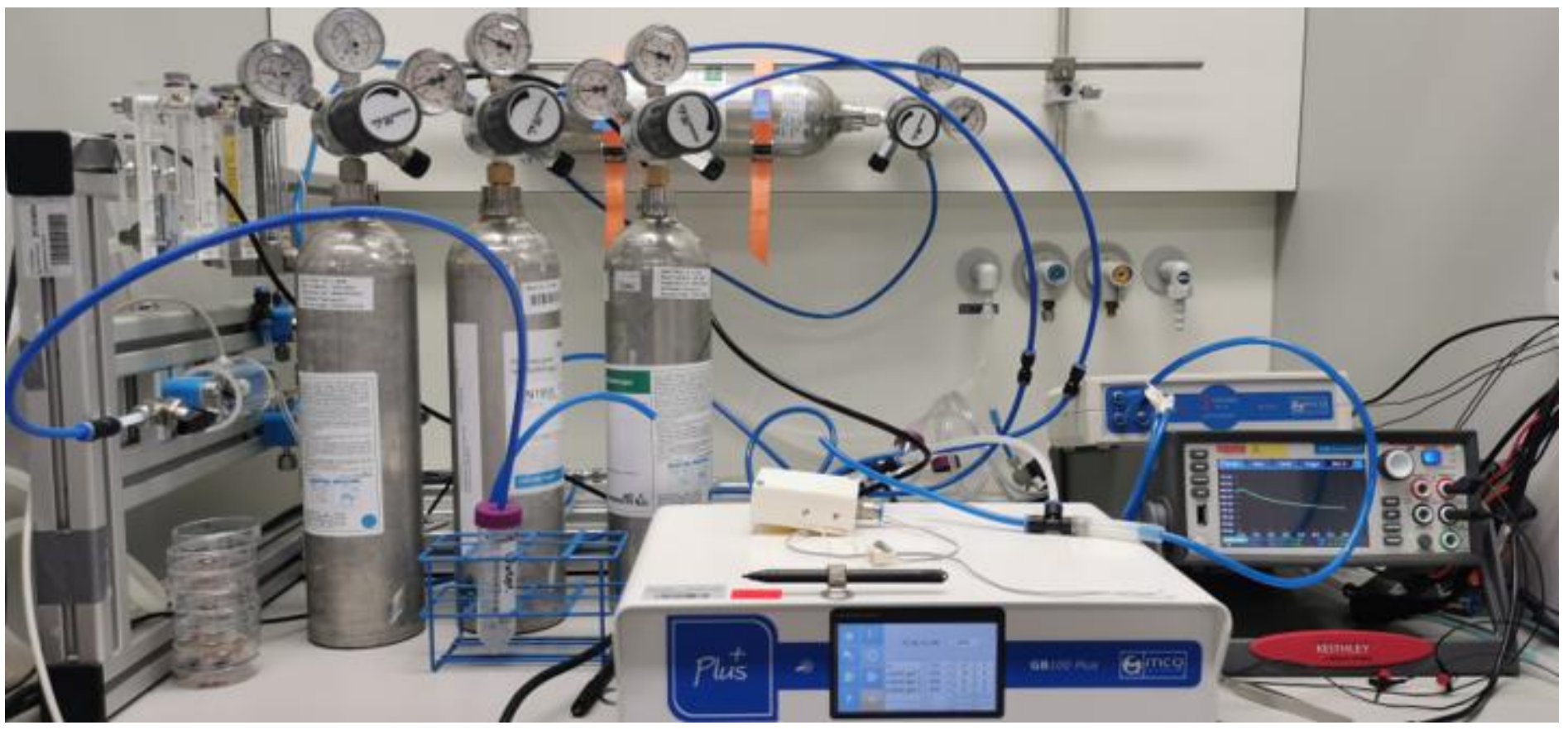

Supplementary Fig. 15 | Photograph of the actual experimental setup.

Comment 9. The manuscript states that the sensor's average power consumption is below 0.7 μW, but does not specify the test scenario for this power consumption data (e.g., static monitoring state, dynamic response state, or power consumption variations under different H_2S concentrations). The power consumption data for commercial sensors (66-100 mW) also fails to clarify whether the test conditions align with this study (e.g., whether the same operating temperature and response trigger threshold were used). It is recommended to supplement the power consumption testing with specific experimental parameters (e.g., test temperature, gas concentration, data acquisition frequency). Additionally, the source literature and testing conditions for the commercial sensor power consumption data should be cited to ensure fair comparison and data reproducibility.

Reply: We appreciate the reviewer's concern regarding the power-consumption scenario and the fairness of comparison with commercial sensors. In the revised manuscript, we explicitly define two operating states for our device: (i) an active sensing state during H_2S exposure, where the average power consumption is <0.7 μW, and (ii) a standby state under pure carrier gas without target-gas exposure, where the power consumption is <0.3 μW (Supplementary Tab. 7). Power consumption was calculated using $P=VI$ from the fixed bias voltage and the time-resolved current recorded during sensing measurements; key experimental parameters (test temperature, H_2S concentration, and data acquisition settings) have been added to the Methods/Supplementary Information to ensure reproducibility.

Regarding commercial sensors (25-1300 mW), we did not perform direct measurements under identical conditions. Instead, these values were taken from publicly available manufacturer datasheets/manuals of representative heated MOS H_2S sensors, where the dominant power budget is associated with the integrated

micro-heater required for elevated-temperature operation. We agree that exact operating conditions and response thresholds may vary across products and may not fully match our testing protocol. Therefore, we present this comparison strictly as an architecture-level energy-budget benchmark (heater-free, room-temperature chemiresistive sensing vs. heater-driven MOS sensing), rather than a one-to-one performance comparison under identical conditions.

To avoid overinterpretation, we have revised the text to clarify the scope and limitations of the datasheet-based comparison and added the corresponding datasheet sources and conditions (where provided) in the Supplementary Information. Even under this conservative framing, the heater-free design in this work reduces the power requirement by orders of magnitude relative to representative commercial heated MOS sensors, highlighting its suitability for continuous and battery-powered deployment.

These new results and the associated discussion have been added to the Supplementary Information (Supplementary Tab. 8) and explicitly referenced in the revised manuscript (Page 17, Line 541-550) and are also highlighted in red accordingly.

Revised parts of manuscript are as following:

Benefiting from heater-free, room-temperature operation, the sensing element in this work operates in the μW power regime with an average power consumption of 0.7 μW in active mode and 0.3 μW in idle mode (details in Supplementary Tab. 7), whereas representative commercial MOS H_2S sensors typically operate in the mW regime (25-1300 mW), as derived from publicly available datasheet specifications. This architecture-level comparison highlights an energy-budget reduction of approximately five orders of magnitude. In addition, the response dynamics are approximately 4-5× faster than those reported for commercial MOS sensors, based on datasheet values and publicly available literature. These comparisons are intended to highlight fundamental differences in sensing architecture, rather than a one-to-one performance comparison under identical testing conditions (details in Supplementary Tab. 8).

Revised parts of SI are as following:

The sensing-element power reported in the manuscript (0.7 μW in active mode and 0.3 μW in idle mode) was calculated from the applied bias voltage and the instantaneous sensor resistance/current according to $P_{sensor} = V_{bias} \times I_{sensor}$ (Supplementary Tab. 7). This value reflects only the intrinsic power consumption of the sensing layer.

To quantify the system-level power consumption, the total supply current of the electronics and wireless communication module was measured under the same operating conditions and data-transmission settings

as used in the experiments. With a regulated supply voltage of 2.5 V, the module draws an average current of 8.19 mA, corresponding to a total power consumption of approximately 20.48 mW. This value inherently includes the duty-cycled operation of ADC sampling, microcontroller processing, and Bluetooth wireless transmission. Accordingly, the sensing-element power represents only a negligible fraction of the overall device power budget, while the dominant contribution arises from the communication and control electronics.

Power consumption for the commercial sensors listed in Supplementary Tab. 8 was extracted from publicly available manufacturer datasheets. These datasheets report the overall power consumption of the device, which includes both the sensing element and any additional components such as heating elements or signal conditioning circuits. Commercial datasheets generally do not provide a full breakdown of power consumption across individual modules (sensing element vs. heater vs. signal processing), so these values should be interpreted as a product-level benchmark rather than a direct one-to-one comparison under identical experimental conditions.

Supplementary Tab. 8 | Power Consumption and Testing Parameters for Sensor and Commercial Sensors.

Sensor Type	Model	T_{90} (s)	LOD (ppm)	Operating Voltage (V)	Current (A)	Power (W)	Power Source Breakdown	Source	Notes
**This work**	**H_2S**	**7**	**0.000328**	**3.725**	**0.1668 µA**	**0.6312 µW**	**Sensor**	**SI**	**Sensing element: µW-level, wireless + ADC = mW-level**
				**2.5**	**0.00819**	**0.0205**	**Wireless**	**SI**	**SI**
Commercial 1	SGX-4H_2S-100	30	/	1.3	1	1.3	Sensor	datasheet	W-level, heat requiremtn, heat power, etc
Commercial 2	H_2S Sensor V1.0	20	0.5	3.3-5	0.02	0.066	Sensor	datasheet	mW-level, heat requiremtn, heat power, etc
Commercial 3	2X MQ136	30	1	5	0.18	0.9	Sensor	datasheet	mW-level, heat requiremtn, heat power, etc
Commercial 4	S-H_2S-01	30	0.1	3.3-5.5	0.05	0.025	Sensor & Wire	datasheet	mW-level, heat requiremtn, heat power, wire transmission etc

Commercial Sensor Datasheet Sources:

Commercial Sensor 1: RS Components GmbH, Industrial Hydrogen Sulphide (H_2S) Sensor, Datasheet, available at [https://docs.rs-online.com/50d2/A700000009288588.pdf], accessed [21.01.2026].

Commercial Sensor 2: DFRobot, Fermion: H_2S Gas Sensor, Datasheet, available at [https://www.dfrobot.com/product-2702.html?srsltid=AfmBOoo57XTXfPiknsJnx7tR_0KkWUVBd00iB-ZgpHbOBsSuZ9ynozD1], accessed [21.01.2026].

Commercial Sensor 3: HUABAN, 1 x MQ-136 MQ136 Hydrogen Sulfide H_2S, Datasheet, available at [https://www.amazon.de/HUABAN-MQ-136-MQ136-Schwefelwasserstoff-H2S-Detektionsgassensormodul-Alarmsignallampe/dp/B089SQDQFJ?source=ps-sl-shoppingads-lpcontext&ref_=fplfs&smid=A169MKB0ILTINE&th=1], accessed [21.01.2026].

Commercial Sensor 4: Seeed Technology, S-H_2S-01, Datasheet, available at [https://files.seeedstudio.com/products/101990863/RES/RS485%20H2S%20Sensor%20-%20Datasheet.pdf], accessed [21.01.2026].

Comment 10. The manuscript defines sensor response as 'S = (Ra/Rg-1) × 100%'. However, Table S1 in the supplementary materials defines response as 'Ra/Rg'. Such inconsistencies may compromise the fairness of comparative results.

Reply: We sincerely thank the reviewer for pointing out the inconsistency between the response formula used in the manuscript and the one presented in Table S1 of the supplementary information. Upon reviewing the manuscript, we realized that the sensor response was defined in the manuscript as:

$$S = (R_a/R_g-1) \times 100\%$$

where R_a and R_g are the resistance values of the sensor in the presence of target gas (H_2S) and background gas, respectively. However, in Table S1, the response was mistakenly listed as $S = R_a/R_g$ (without subtracting 1 and multiplying by 100%). This inconsistency was due to a formatting error in Supplementary Tab. 1. We have corrected this issue by updating Supplementary Tab. 1 to reflect the correct formula and have ensured consistency between the formula used in the manuscript and the data presented in the supplementary information.

We have revised the manuscript and supplementary information in accordance with all comments, and we hope these all address any confusion.

To Reviewer: 2

General comments: This study proposes an ultra-low-power wireless hydrogen sulfide sensor based on metal-organic framework-derived ZnO, featuring exceptional sensitivity and rapid response time for trace gas detection. Although this approach addresses significant challenges in gas sensing and demonstrates remarkable performance metrics, the novelty of the sensor design—particularly the integration of Schottky barrier and grain boundary engineering—has been extensively documented in the literature for similar MOF-derived sensors and Schottky barrier modification techniques. This paper fails to sufficiently articulate how this specific solution significantly advances the state of the art.

Reply: We sincerely thank the reviewer for the thoughtful evaluation of our work and for acknowledging the significance of developing an ultra-low-power wireless H_2S sensor capable of trace-level detection with rapid response. We fully appreciate the reviewer's concerns regarding the novelty of our approach, particularly the role of Schottky-barrier modulation and grain-boundary engineering in MOF-derived ZnO systems. In the revised manuscript, we have substantially strengthened the articulation of our contributions by clarifying how the simultaneous and dynamic coupling of Au-ZnO Schottky modulation with defect-engineered grain-boundary transport represents a distinct advancement beyond previously reported strategies. We further provide new mechanistic evidence, extended characterization, and comparative analyses that explicitly differentiate our system from prior MOF-derived sensors and existing Schottky-based designs.

We appreciate your constructive insights, which have encouraged us to refine the novelty statement and deepen mechanistic explanations. In the revised version, we have carefully addressed all your comments in a detailed point-by-point response that thoroughly explains how each issue has been resolved. All changes in the manuscript have been highlighted in red. We are grateful for your comments, which have significantly improved the clarity, rigor, and contextual positioning of our work.

Comment 1. MOF-derived metal oxides for H_2S sensing have been a research hotspot in recent years, but this paper fails to clearly articulate its innovative differentiation from similar work, resulting in insufficient novelty. In 2024, Montoro et al. (Composites Part B: Engineering Volume 283, 2024, 111637) reported "defect-engineered ZnO derived from MOFs for low-concentration H_2S detection." The "grain boundary engineering" approach in this paper essentially extends the concept of "defect engineering" and does not propose novel structural design strategies.

Reply: We thank the reviewer for this important and constructive comment regarding the novelty of our work, and for explicitly pointing out the recent study by Montoro et al. (Composites Part B: Engineering, 2024), which reports defect-engineered MOF-derived ZnO for low-concentration H_2S detection. We fully acknowledge that MOF-derived metal oxides and defect engineering strategies have become an active research hotspot in recent years.

We would like to clarify, however, that the central innovation of the present work does not lie in proposing a new MOF-derived material or extending defect engineering concepts. Instead, our work introduces grain boundary density (GBD) as a distinct, mesoscopic, and quantitatively tunable control parameter that governs charge transport and interfacial barrier behavior at the device level. While the study by Montoro et al. primarily focuses on local point defects (e.g., oxygen vacancies) and defect-mediated adsorption chemistry, our approach treats grain boundaries as a transport-regulating structural entity, which determines both the initial Schottky barrier and its dynamic modulation upon H_2S adsorption.

Importantly, we establish a direct and quantitative correlation between grain boundary density, Schottky barrier height/modulation amplitude, and sensing performance, thereby revealing a transport- and interface-governed sensing mechanism that goes beyond defect-mediated adsorption enhancement. To the best of our knowledge, such a grain boundary density-regulated Schottky barrier modulation framework has not been systematically reported for MOF-derived ZnO-based H_2S sensors.

To address this concern more comprehensively and to avoid any ambiguity regarding novelty, we have substantially revised the Introduction, which also responds to Reviewer 1's Comments. Specifically, we have added:

(i) a clearer articulation that MOF-derived routes are employed as a microstructure-tunable platform rather than a novelty in material composition;

(ii) a focused discussion on the advantages of MOF-derived metal oxides in enabling controllable grain boundary density and mesoscopic transport regulation;

(iii) an explicit explanation of how Schottky interfaces amplify sensing signals through barrier modulation, thereby providing high sensitivity and selectivity under room-temperature operation; and

(iv) a clear distinction between defect engineering strategies (with Montoro et al. cited as a representative example) and our grain boundary density-regulated, mechanism-driven sensing architecture.

These revisions ensure that the two core innovative strategies of this work—grain boundary density regulation and Schottky barrier modulation—are clearly motivated, logically introduced, and differentiated

from prior defect-focused studies, allowing readers to grasp the novelty and design logic before entering the technical details of the manuscript.

These further discussions have been added to the revised manuscript (Page 2-3, Line 60-91, and Line 101-109) and are also highlighted in red accordingly.

Revised parts of manuscript are as following:

Unlike defect-engineering strategies that primarily target local point defects and adsorption chemistry [50], grain boundary engineering addresses mesoscopic transport regulation and interfacial Schottky barrier formation/modulation, providing a fundamentally different route for mechanism-driven performance optimization.

Additionally revised parts of manuscript are as following:

In practical application scenarios, such as anaerobic food preservation systems and enclosed industrial environments (e.g., storage tanks, pipelines, and confined workspaces), the monitoring requirements for H_2S are particularly stringent [39-40]. In anaerobic or low-oxygen food preservation conditions, trace-level H_2S released during early-stage spoilage must be detected under high humidity and limited oxygen availability, where conventional metal-oxide sensors often exhibit sluggish response, poor reversibility, and elevated power consumption. In these settings, the elevated operating temperatures commonly required by traditional metal-oxide sensors not only increase power consumption but also pose safety risks and limit long-term, battery-powered deployment. Moreover, the reliance on oxygen-assisted surface reactions and unstable interfacial charge transport frequently leads to signal drift and reduced reliability under low-oxygen or confined conditions [41-43]. These practical constraints highlight the need for H_2S sensors that meet stringent real-world monitoring requirements, including room-temperature operation, ultra-low power consumption, and stable performance under low-oxygen and confined conditions. Addressing these application-driven challenges requires a sensing mechanism that fundamentally decouples performance from thermal activation and instead relies on robust interfacial electronic modulation.

Metal-organic framework (MOF)-derived metal oxides have emerged as attractive platforms for gas sensing owing to their intrinsically high specific surface area and, more importantly, the ability to tailor microstructural features through controlled thermal conversion [44-46]. Previous studies have shown that the pyrolysis of MOF precursors enables precise regulation of grain size, porosity, and grain boundary density in the resulting metal oxides, providing a versatile route to engineer charge transport pathways and surface reactivity. However, despite these advantages, the role of grain boundary density as an active and

tunable parameter governing gas-sensing performance has rarely been systematically explored. In parallel, Schottky interfaces between metals and semiconducting oxides are well known to amplify gas-sensing signals by converting surface adsorption-induced electronic perturbations into exponential changes in carrier transport [47-49]. Despite these advances, the grain boundary density has rarely been treated as an active and tunable physical parameter governing Schottky barrier formation and dynamic barrier modulation during gas adsorption. Unlike defect-engineering strategies that primarily target local point defects and adsorption chemistry [50], grain boundary engineering addresses mesoscopic transport regulation and interfacial Schottky barrier formation/modulation, providing a fundamentally different route for mechanism-driven performance optimization. As a result, how microstructural parameters interact with interfacial electronic transport to determine sensing performance remains insufficiently understood. Motivated by this gap, we identify grain boundary density as a decisive control knob that simultaneously governs the initial Schottky barrier and its modulation efficiency, enabling a mechanism-driven optimization of H_2S sensing performance.

While MOF-derived metal oxides have been widely explored for H_2S sensing, most previous studies primarily focus on material composition optimization or morphological engineering. In contrast, the novelty of this work does not lie in the use of MOF-derived ZnO itself, but in identifying grain boundary density as a decisive and tunable physical parameter that governs both the initial Schottky barrier and its dynamic modulation during gas adsorption. By leveraging the temperature-sensitive microstructural evolution inherent to MOF-derived ZnO, we establish a quantitative correlation between grain boundary density, barrier modulation efficiency, and sensing performance. This mechanism-oriented strategy enables simultaneous enhancement of sensitivity and power efficiency without relying on complex structural designs, offering a generalizable pathway for high-performance H_2S sensing.

Comment 2. The experiment used "anaerobic fermentation of boiled eggs" to simulate food spoilage, but did not specify the storage conditions of the eggs (such as whether they were protected from light or whether there were differences in microbial contamination). It also failed to measure the concentrations of other volatile organic compounds (such as amines and organic acids) during fermentation, making it impossible to rule out interference from sensor responses to other gases.

Reply: We thank the reviewer for the comments on the anaerobic boiled-egg spoilage model and potential interference from co-existing volatiles. In the revised manuscript, we have clarified the storage protocol to improve reproducibility: eggs were boiled for 10 min, cooled to room temperature, peeled, adjusted to

identical mass, individually sealed in clean glass vials with tightened caps to minimize air exchange, and stored in a closed container under dark conditions at ambient temperature for predefined durations before headspace testing.

We recognize that spoilage headspace can contain multiple volatile species (such as amines and organic acids). To address possible cross-sensitivity, we added a stringent dynamic interference stability evaluation in which the H_2S response was monitored over repeated cycles while other interfering volatile organic compounds (methylamine, dimethylamine, trimethylamine, acetic acid, butyric acid, methyl mercaptan, formaldehyde, ethanol and acetone) and gases (including oxidizing and reducing species and their mixtures) were co-introduced. The sensor maintains a stable and reproducible H_2S response with negligible baseline drift under these conditions (Supplementary Fig. 13). These results, along with a detailed description of the experimental setup and gas introduction protocol, have been included in Supplementary Fig. 14-15.

These new results and the associated discussion have been added to the Supplementary Information (Supplementary Fig. 13-15) and explicitly referenced in the revised manuscript (Page 8, Line 250-258) and are also highlighted in red accordingly.

Revised parts of method are as following:

Fresh eggs were boiled in water for 10 min and cooled to room temperature. The shells were removed to minimize variability associated with shell permeability and surface residues. The peeled eggs were adjusted to identical mass, placed individually into clean glass vials, and tightly capped to minimize air exchange (approximating anaerobic storage). The vials were stored in a closed container under dark conditions at ambient temperature for predefined durations to generate headspace samples representing different spoilage stages. After the specified storage time, the vials were opened and the headspace was measured.

Revised parts of the manuscript are as follows:

To assess cross-interference under mixed-gas conditions relevant to practical environments, we performed dynamic co-exposure tests in which H_2S was introduced together with representative oxidizing gases (NO_2 and SO_2) and a reducing gas (NH_3), both individually and in mixtures. The sensor maintains a stable and reproducible H_2S response even when oxidizing and reducing interferents coexist simultaneously, demonstrating strong anti-interference capability in complex gas matrices (Supplementary Fig. 13-15). The robustness is particularly relevant to the boiled-egg spoilage headspace tests, where multiple VOCs (e.g., amines, organic acids, and small sulfur-containing species) may be present. The mixed-gas evaluation

therefore supports that the response observed in the spoilage demonstration is primarily governed by H_2S-associated surface/interfacial modulation rather than nonspecific responses to coexisting volatiles.

Revised parts of SI are as follows:

To evaluate the robustness of the H_2S sensing signal under realistic multi gas environments, we conducted a dynamic interference stability test by repeatedly exposing the sensor to 3 ppm H_2S over 18 consecutive response–recovery cycles. During each cycle, one set of interfering gases was introduced simultaneously with H_2S, while all other experimental parameters (e.g., concentration control, total flow rate, temperature, and bias/measurement settings) were kept constant. In particular, to directly address the coexistence of oxidizing and reducing species, we tested representative oxidizing gases (NO_2, SO_2) and a reducing gas (NH_3) both individually and in mixtures, including NO_2 + SO_2, NO_2 + NH_3, SO_2 + NH_3, and NO_2 + SO_2 + NH_3. The sensor exhibits highly reproducible H_2S response amplitudes with negligible baseline drift across all cycles, indicating that the sensing output is dominated by H_2S-triggered interfacial modulation rather than transient cross-interference from coexisting gases (Supplementary Fig. 13). A schematic of the gas-introduction protocol and the overall setup is provided in Supplementary Fig. 14, and a photograph of the actual experimental setup is shown in Supplementary Fig. 15 to ensure transparency and reproducibility.

In addition to the inorganic interferents above, we further evaluated potential cross-sensitivity to representative VOC interferents that are commonly reported in food spoilage headspaces, particularly during egg fermentation. These VOCs include amines (triethylamine, propylamine, butylamine), organic acids (acetic acid and butyric acid), sulfur-containing species (dimethyl sulfide), and small oxygenated molecules (formaldehyde, ethanol, and acetone). The sensor maintains stable and reversible H_2S responses under these VOC interference conditions as well (Supplementary Fig. 13), further supporting its robustness in complex gas matrices.

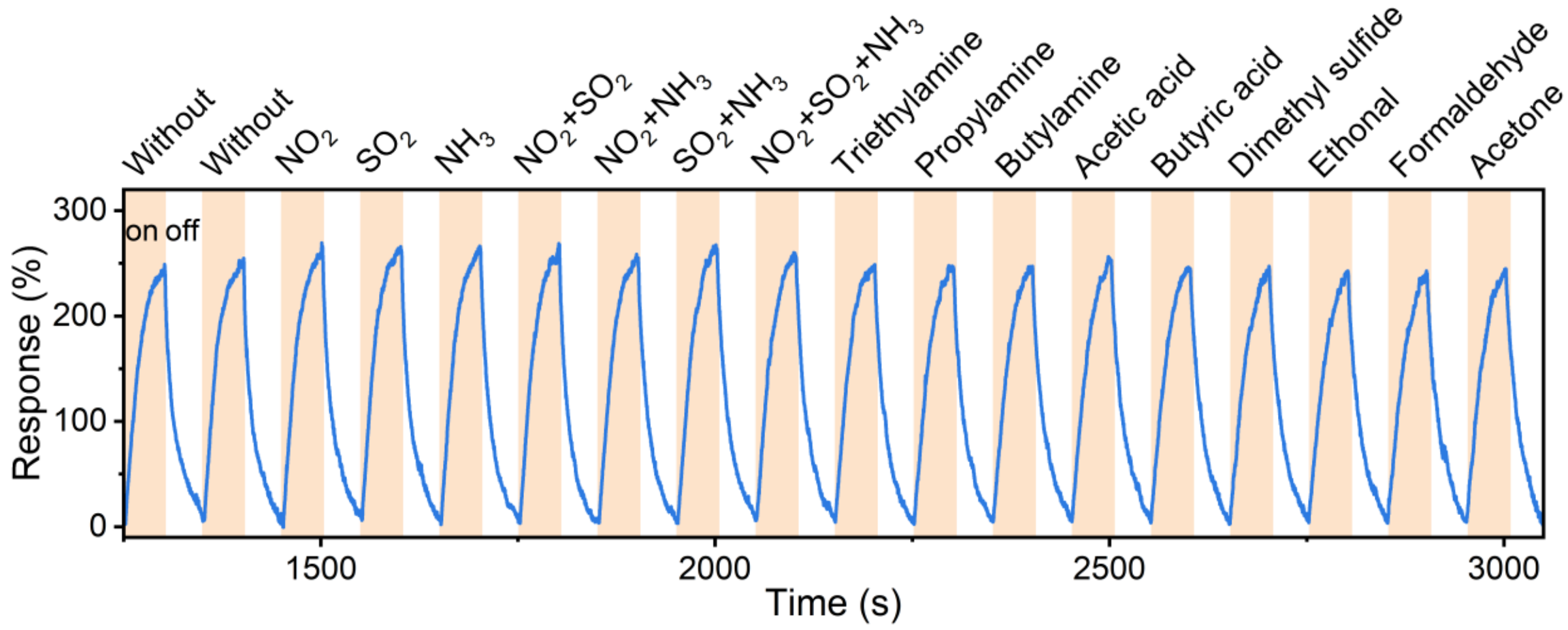

Supplementary Fig. 13 | Dynamic interference stability of the H_2S sensor.

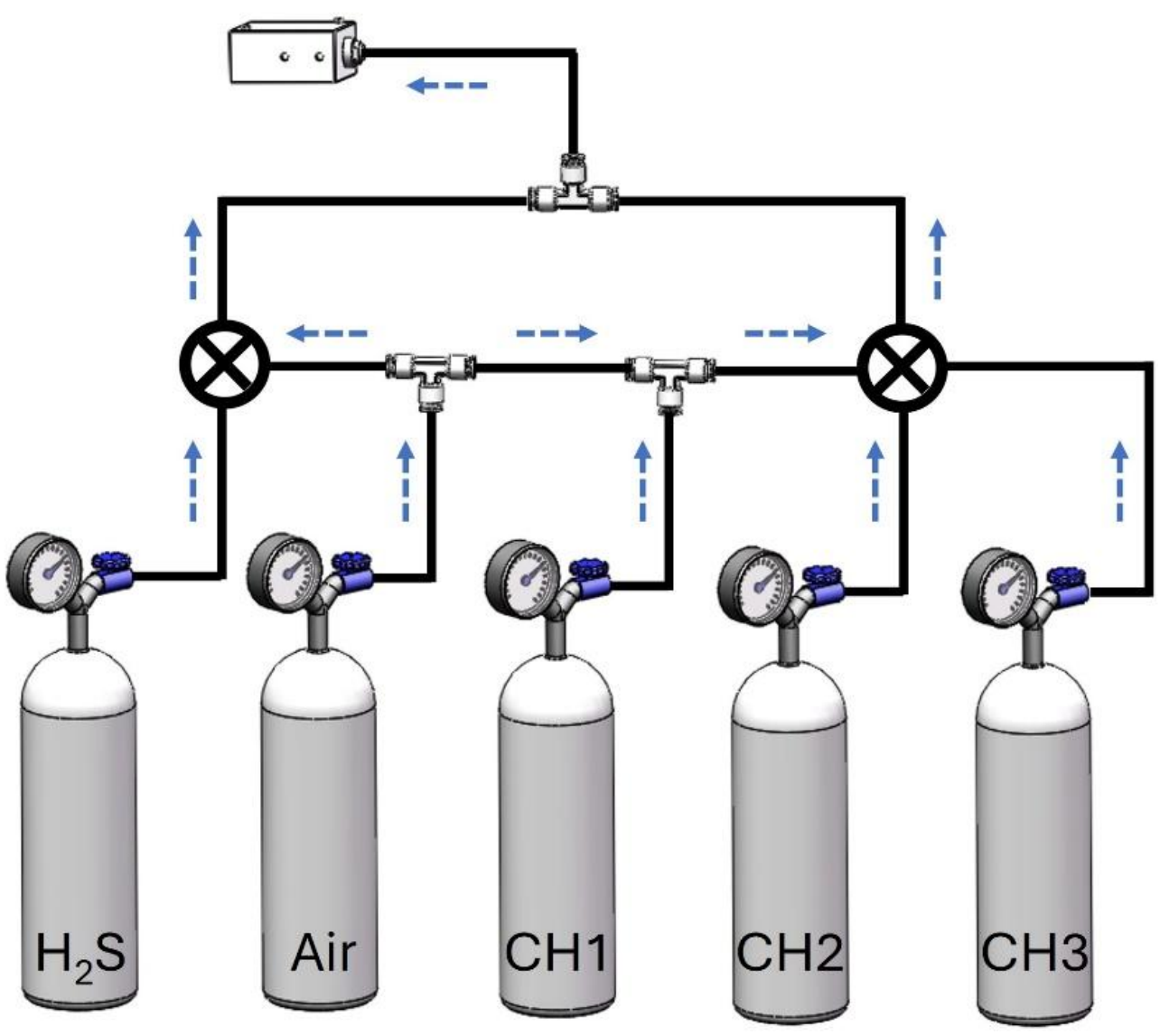

Supplementary Fig. 14 | Experimental setup for dynamic interference stability measurements.

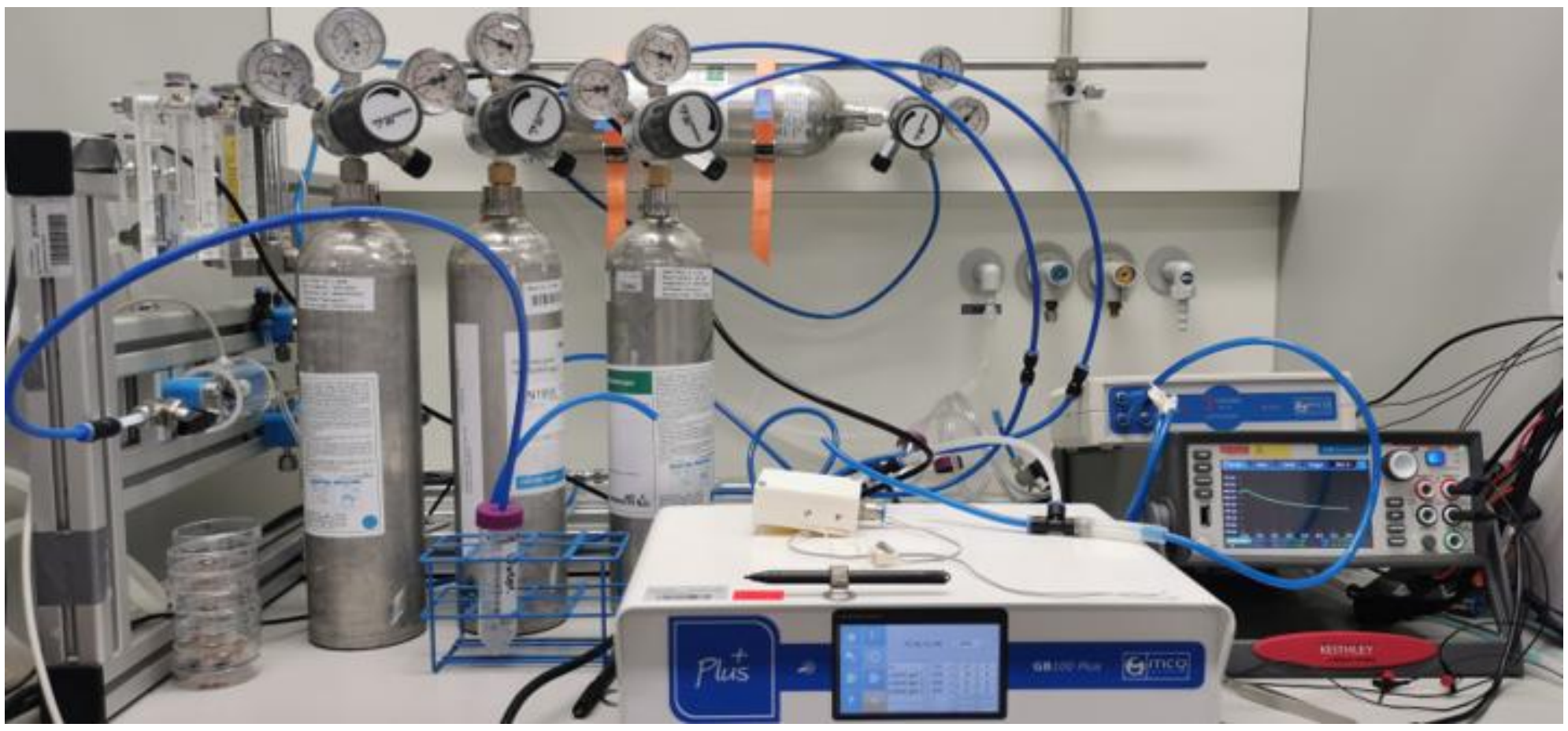

Supplementary Fig. 15 | Photograph of the actual experimental setup.

Comment 3. Pipe leak detection was tested only under ideal conditions of 5 cm distance and 50 ppm H_2S, failing to simulate long-distance detection (e.g.,1-5 m), airflow disturbances, or dust interference in actual industrial environments. Confined space testing used only a sealed chamber with 1 ppm H_2S, neglecting the irreversible damage high-concentration H_2S (e.g., above 100 ppm) inflicts on sensors in industrial scenarios.

Reply: We sincerely thank the reviewer for highlighting the gap between idealized tests and practical industrial leak-monitoring conditions. In the revised manuscript, we have expanded our evaluation to better simulate industrial leak-monitoring scenarios that involve airflow perturbations and high-concentration H_2S exposure.

Beyond the original 5 cm condition, we expanded the distance testing to evaluate the sensor's performance over a more realistic range of distances. The sensor was placed at 0.50 m, 1.0 m, 3.0 m, and 5.0 m from the leak outlet in still air, with all other parameters kept constant. As expected, the response decreased with increasing distance due to dilution, but even at 5 m, a weak yet identifiable response was still observed, demonstrating the sensor's ability to detect leakage over meter-scale distances (details in Supplementary Fig. 35a-d).

Regarding the concern about airflow disturbances, we simulated industrial airflow perturbations by fixing the sensor-to-leak distance at 1.0 m and applied sequential conditions: still air → weak airflow → weak airflow + dust → strong airflow. An axial fan (diameter ~10 cm) was placed between the sensor and the gas source, operating at two voltage levels (8 V and 15 V) to simulate weak and strong convection. Specifically, at 8 V, the fan generates a weak convection velocity of approximately v_{weak} = 0.5 m/s, and at 15 V, a strong convection velocity of v_{strong} = 1.2 m/s, with time averages taken over ≥30 s. Under weak airflow-only conditions, baseline fluctuations increase and the apparent response amplitude is partially reduced; the strongest airflow produces the largest noise. For dust interference, 20 mg dried, sieved soil powder was placed at the fan inlet and naturally entrained under weak airflow (30 s × 2 pulses). Although the curves exhibit larger noise during dust exposure, clear response and recovery features remain identifiable (details in Supplementary Fig. 35e-f), indicating robustness against combined airflow and dust disturbances.

To assess the high-concentration H_2S robustness, we exposed the sensor to 200, 400, 600, 800, and 1000 ppm H_2S and recorded the corresponding response/recovery curves. The sensor exhibited reversible responses without observable saturation up to 1000 ppm, with higher concentrations producing larger response amplitudes and improved signal-to-noise ratios. Importantly, no irreversible baseline drift was

observed, indicating that the sensor is resistant to short-term high-concentration shocks under practical conditions (details in Supplementary Fig. 36).

These new results and the associated discussion have been added to the Supplementary Information (Supplementary Fig. 35-36) and explicitly referenced in the revised manuscript (Page 16, Line 520-526), highlighted in red accordingly.

Revised parts of manuscript are as follows:

To better simulate practical leak-monitoring conditions, the sensor under several realistic scenarios were evaluated. Beyond the original 5 cm condition, the sensor was positioned at 0.50 m, 1.0 m, 3.0 m, and 5.0 m from the leak outlet in still air. To simulate industrial airflow disturbances, the sensor was fixed at 1.0 m from the leak outlet and exposed to weak airflow, weak airflow with dust, and strong airflow. These results emphasize the sensor's effectiveness in realistic conditions, ensuring early leak detection and reducing safety and economic risks (details in Supplementary Fig. 35).

To evaluate sensing behavior at high H_2S concentrations (>100 ppm) in industrial scenarios, the sensor at 200, 400, 600, 800, and 1000 ppm was measured. The baseline returned without irreversible drift, indicating the sensor's short-term tolerance to high-concentration shocks under the tested conditions (details in Supplementary Fig. 36).

Revised parts of SI are as follows:

To better simulate practical leak-monitoring conditions, the sensor under several realistic scenarios were evaluated, including (i) long-distance detection (0.50-5.0 m), (ii) airflow and dust disturbances under forced convection, and (iii) high-concentration H_2S robustness (200-1000 ppm) to assess saturation and irreversible damage.

Long-distance detection was examined by positioning the sensor at 0.50 m, 1.0 m, 3.0 m, and 5.0 m from the leak outlet under still-air conditions (fan off), while keeping all other operating parameters unchanged. As expected, the response decreased with distance, but a noticeable response was still observed at 5.0 m, demonstrating the sensor's ability to detect leaks over meter-scale distances (Supplementary Fig. 35a-d).

To simulate industrial airflow perturbations, we fixed the sensor-to-leak distance at 1.0 m and four sequential conditions were applied: (i) still-air baseline; (ii) weak forced convection generated by a miniature DC fan powered at 8 V; (iii) weak convection with dust challenge, implemented by placing 20 mg of dried, sieved soil powder at the fan inlet for natural entrainment (30 s per pulse, two pulses, separated by baseline

recovery); and (iv) strong forced convection generated by increasing the fan voltage to 15 V. Airflow disturbances were introduced using an axial fan (diameter ~10 cm), placed between the sensor and the gas source, operating at two voltage levels (8 V and 15 V) to simulate weak and strong convection. Specifically, at 8 V, the fan generates a weak convection velocity of approximately v_{weak} = 0.5 m/s, and at 15 V, a strong convection velocity of v_{strong} = 1.2 m/s, with time averages taken over ≥30 s.

Under weak airflow-only conditions, baseline fluctuations increase and the apparent response amplitude is partially reduced; the strongest airflow produces the largest noise. For dust interference, 20 mg dried, sieved soil powder was placed at the fan inlet and naturally entrained under weak airflow (30 s × 2 pulses). Despite increased noise during dust exposure, the response and recovery features remained identifiable, indicating the sensor's robustness against combined airflow and dust disturbances (Supplementary Fig. 35e-g), indicating robustness against combined airflow and dust disturbances.

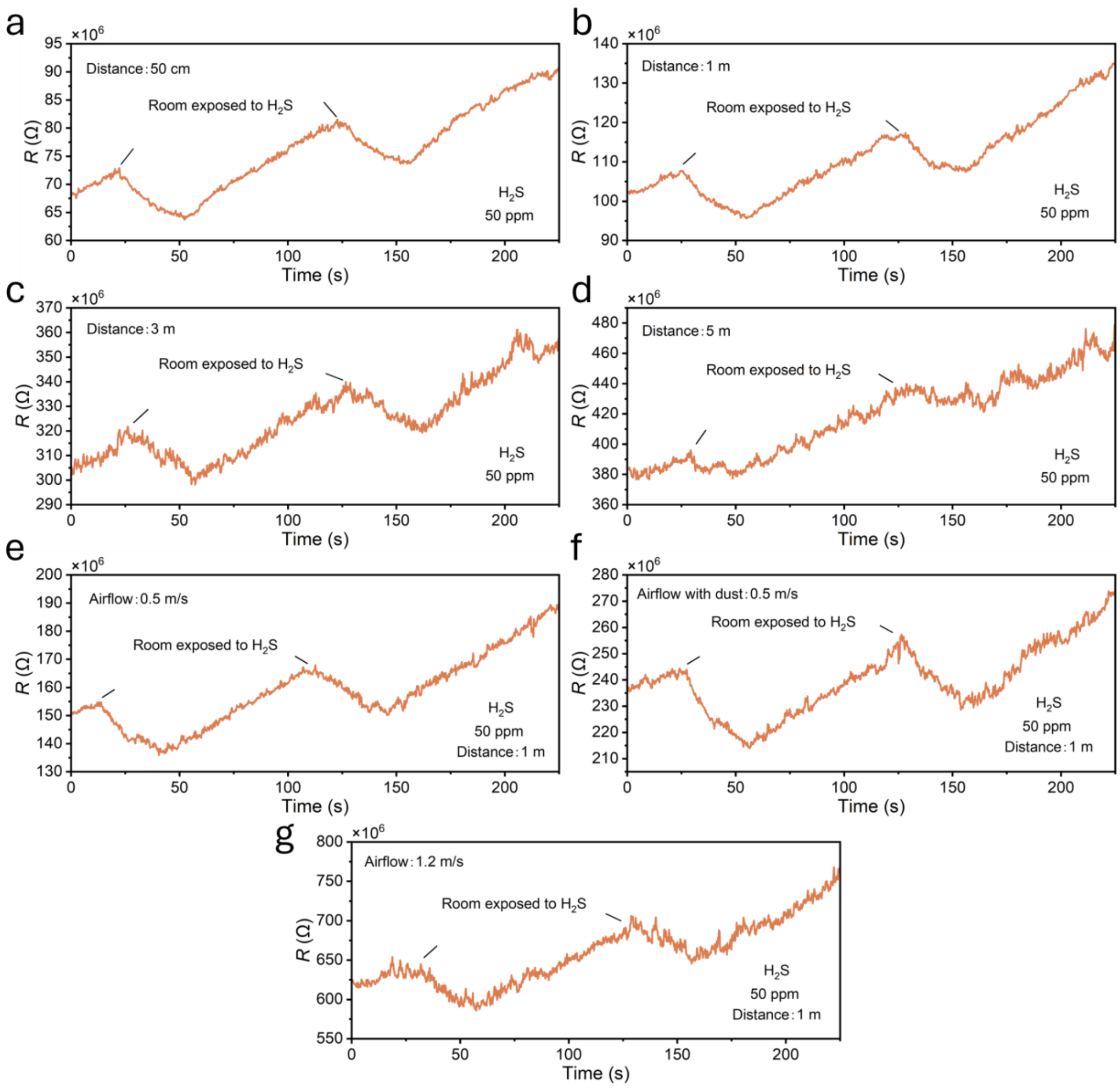

Supplementary Fig. 35 | a-d, Response and recovery curves at different distances from the leak outlet: 0.50 m, 1.0 m, 3.0 m, and 5.0 m. **e-g,** Response and recovery curves under different airflow and dust disturbance conditions at a fixed distance of 1.0 m from the leak outlet: **e,** under 0.5 m/s weak airflow; **f,** under 0.5 m/s weak airflow with dust interference (20 mg dried, sieved soil powder); **g,** under 1.2 m/s strong airflow.

High-concentration robustness was evaluated by exposing the sensor to 200, 400, 600, 800, and 1000 ppm H_2S. The sensor exhibited rapid and reversible response/recovery across this range, with an improved signal-to-noise ratio at higher concentrations and no saturation up to 1000 ppm. The baseline returned without irreversible drift, indicating the sensor's short-term tolerance to high-concentration shocks under the tested conditions (Supplementary Fig. 36).

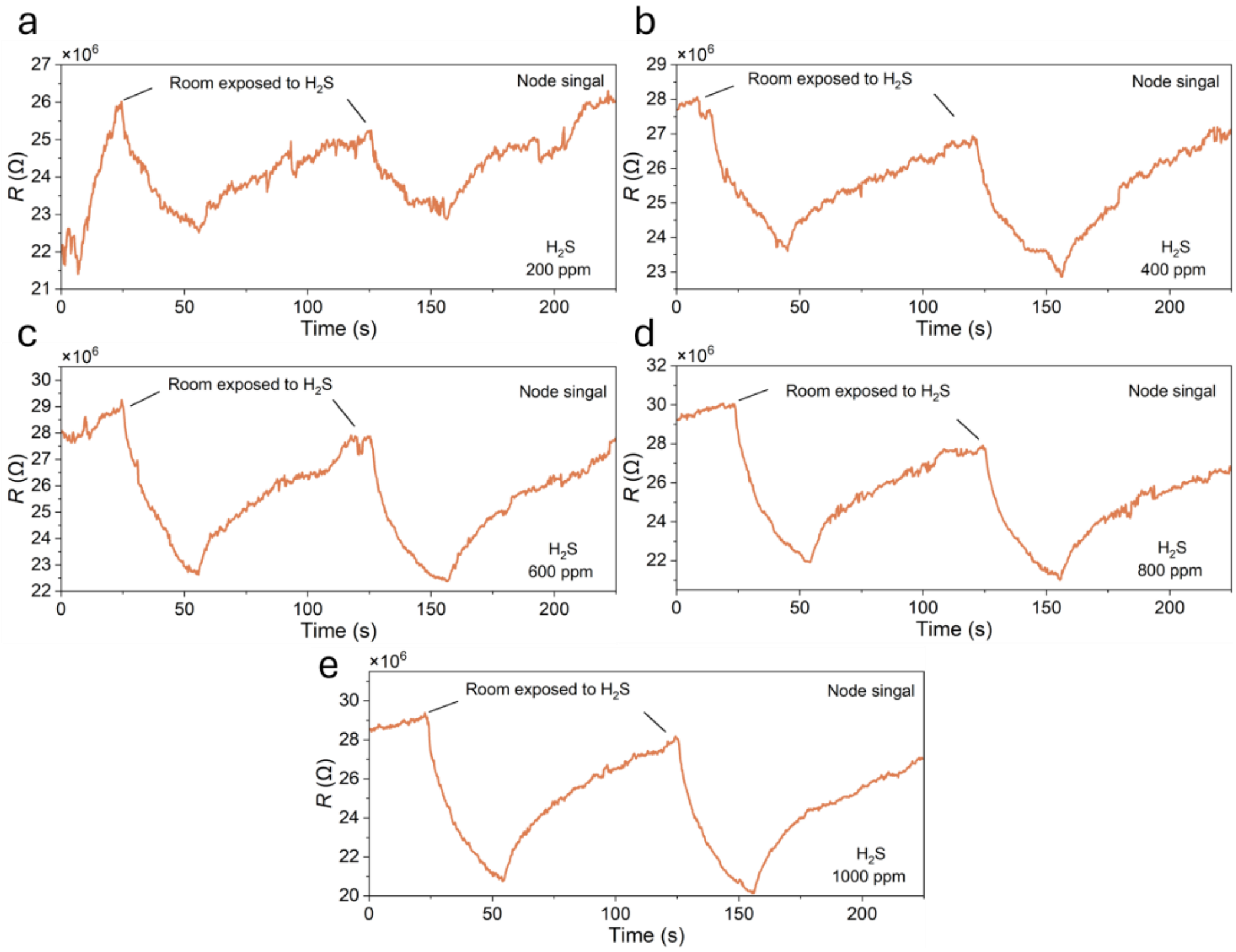

Supplementary Fig. 36 | a-e, Response and recovery curves under high concentrations of H_2S: 200 ppm, 400 ppm, 600 ppm, 800 ppm, 1000 ppm.

Comment 4. XPS and Raman spectroscopy detected oxygen vacancies and zinc vacancies respectively, but the contribution of these two defects to H_2S adsorption was not compared via DFT calculations. The statement that oxygen vacancies are the primary defect is too general to explain why ZMA450 exhibits

weaker sensing performance despite having a higher oxygen vacancy concentration (greater proportion in XPS measurements) than ZMA550.

Reply: We agree that attributing the sensing behavior to oxygen vacancies alone, based solely on XPS and Raman, is not sufficiently specific to explain the inferior performance of ZMA450 relative to ZMA550. To further clarify this, we performed detailed DFT calculations to compare the contributions of oxygen vacancies and zinc vacancies to H_2S adsorption and the associated defect energetics.

DFT calculations show that oxygen vacancies have a significantly higher H_2S adsorption energy (-0.3096 eV) compared to zinc vacancies (-0.1304 eV), confirming that oxygen vacancies are the primary adsorption sites for H_2S. These findings suggest that oxygen vacancies play a key role in the adsorption mechanism, and are likely the most active defect sites for H_2S interaction. In comparison, the zinc vacancies, despite their presence, contribute less to H_2S adsorption. Additionally, we further confirmed the charge distribution upon H_2S adsorption at the oxygen and zinc vacancy sites by calculating the differential charge density. To quantify this, we performed Bader charge calculations, which clearly show the amount of charge transferred from the H_2S molecule to the material. Specifically, we found that when H_2S adsorbs at the oxygen vacancy, a charge of -0.1658 is transferred to the material, while at the zinc vacancy, the transferred charge is -0.1370. This further supports the dominant role of oxygen vacancies in the adsorption and reaction processes.

In parallel with our DFT findings, XPS analysis reveals a higher oxygen-vacancy-related component in ZMA450, yet EBSD shows that ZMA450 has much smaller grains and a significantly higher grain-boundary density than ZMA550. This microstructural difference contributes to more pronounced grain boundary effects in ZMA450, where grain boundaries introduce potential barriers that hinder carrier mobility. UPS-derived surface energetics indicate that ZMA450 has a larger interfacial barrier, which results in severe carrier depletion and reduced inter-grain transport, thereby weakening its sensing performance despite the higher vacancy signal observed via XPS.

These findings are further supported by EPR measurements, which show a defect signal at $g \approx 2.003$-2.004 across all samples, with a higher intensity in the lower-temperature samples. This suggests that the higher population of vacancy-related paramagnetic centers in ZMA450 does not translate into superior sensing output, as the grain boundary limitations dominate its transport properties. We have revised and added the supporting data to the Supplementary Information (Supplementary Fig. 33).

In summary, while ZMA450 has a higher oxygen-vacancy concentration, its finer grain structure and higher grain-boundary density result in transport bottlenecks that hinder its sensing performance. This highlights the complex interplay between defect density and microstructure in optimizing material response.

These new results, including the DFT-calculated H_2S adsorption energies, differential charge density, the Bader charge distribution, and the microstructural analysis, have been added to the Supplementary Information (Supplementary Fig. 32-33, and Tab. 5-6) and explicitly referenced in the revised manuscript (Page 6-7, Line 196-205 and Page 14, Line 440-446), highlighted in red accordingly.

Revised parts of manuscript are as follows:

To further investigate the influence of defects on sensing performance, we performed DFT calculations to evaluate the adsorption of H_2S at both oxygen and zinc vacancy sites. The results confirm that oxygen vacancies play a more significant role in H_2S adsorption, and full data and analysis are given in Supplementary Fig. 32 and Tab. 5-6.

Additionally, revised parts of manuscript are as follows:

Additionally, the electron paramagnetic resonance spectroscopy (EPR) measurements were performed to directly assess the oxygen-vacancy concentration trends in the samples ($g \approx 2.003$-2.004) and reveal a monotonic decrease in signal intensity with increasing annealing temperature, and full data and analysis are given in Supplementary Fig. 33.

The aforementioned surface reactions induce substantial modifications in the electronic structure of ZnO, altering the surface charge distribution and bulk carrier concentration. XPS, UV-vis, and EPR analyses collectively indicate that oxygen-vacancy-related defects represent a dominant class of surface-active sites in the ZnO matrix, giving rise to a Zn-rich and non-stoichiometric surface. Such defect-rich surfaces exhibit enhanced Lewis acidity and facilitate H_2S adsorption and activation at the chemical level. However, as demonstrated by the comparative analysis of ZMA450 and ZMA550, the overall sensing performance is ultimately governed by the coupled effects of defect chemistry and charge-transport efficiency.

Notably, ZMA450 shows a markedly lower response and less favorable response-recovery dynamics, despite its higher apparent oxygen-vacancy fraction inferred from XPS (see discussion below). This non-monotonic trend indicates that the sensing output is not governed by a single defect indicator but rather by the coupled effects of defect chemistry and charge-transport across GBs. The observed trend is further corroborated by the consistent response patterns across different samples when exposed to higher H_2S concentrations under N_2 atmosphere (Supplementary Fig. 2), suggesting that the intrinsic transport properties

dominate the inter-sample differences under oxygen-free conditions. In the case of ZMA450, its finer grain structure and higher grain-boundary density, as revealed by EBSD analysis, create transport bottlenecks, further hindering its sensing performance. This highlights that the balance between defect density and microstructure is crucial for optimizing the material's response.

Revised parts of SI are as follows:

To investigate the influence of oxygen and zinc vacancies on H_2S adsorption and sensor performance, we performed DFT calculations to evaluate the adsorption energies and charge distributions at both vacancy sites (Supplementary Fig. 32). The calculations revealed that H_2S preferentially adsorbs at the oxygen vacancy sites, with a greater charge transfer to the material compared to zinc vacancies. Specifically, we observed that oxygen vacancies contribute more significantly to the adsorption and reaction processes. The adsorption energies for H_2S at oxygen and zinc vacancy sites are -0.3096 eV and -0.1304 eV, respectively (Supplementary Tab. 5).

The differential charge density analysis (Supplementary Fig. 32c and f) provides further insight into the charge transfer upon adsorption. As shown in the differential charge density, the blue region represents the depletion of charge, while the yellow region indicates the accumulation of charge. Notably, for the oxygen vacancy, a large blue region is observed, indicating a significant amount of charge is transferred from the H_2S molecule to the material surface. This suggests that oxygen vacancies are the primary sites for H_2S adsorption, where a large amount of charge is accumulated at the interface. In contrast, the zinc vacancy shows a smaller blue region, indicating a lesser degree of charge transfer, further supporting the conclusion that oxygen vacancies play a more prominent role in H_2S adsorption. Additionally, the Bader charge analysis confirms that oxygen vacancies contribute more charge to the adsorbed H_2S molecule (-0.1658), compared to zinc vacancies (-0.1370), highlighting the role of oxygen vacancies as the primary adsorption site (Supplementary Tab. 6).

These findings support the experimental observation that ZMA450, despite having a higher oxygen-vacancy concentration, exhibits weaker sensing performance due to its smaller grain size and higher grain-boundary density, which limit charge transport and reduce response efficiency. The grain boundary limitations in ZMA450 hinder its ability to effectively translate surface charge modulation into a macroscopic conductance change, thus impairing its sensing performance.

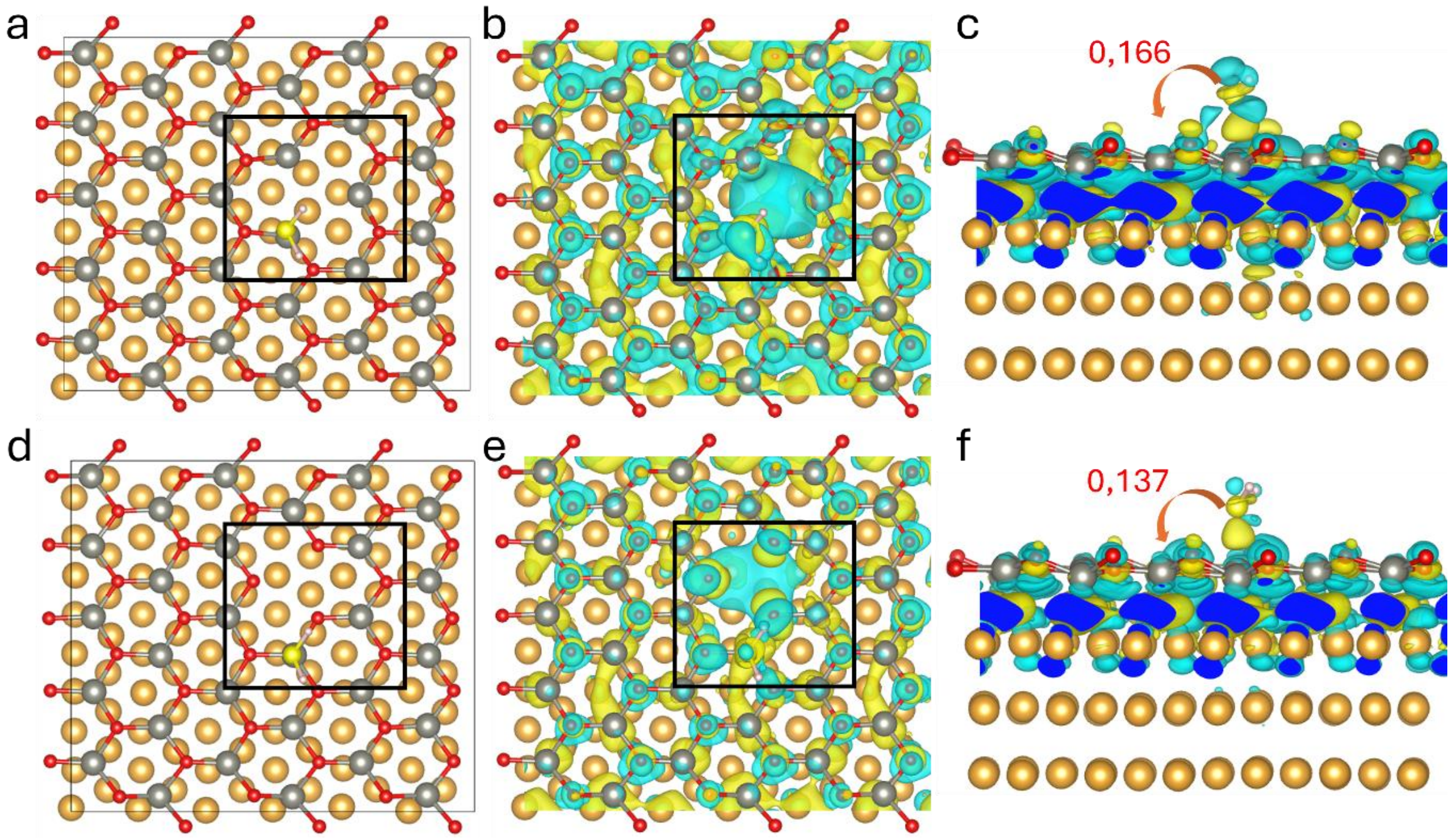

Supplementary Fig. 32 | DFT-based analysis of H_2S adsorption on oxygen and zinc vacancy sites. **a-c,** Oxygen vacancy: adsorption structure, differential charge density, and charge transfer analysis. **d-f,** Zinc vacancy: adsorption structure, differential charge density, and charge transfer analysis.

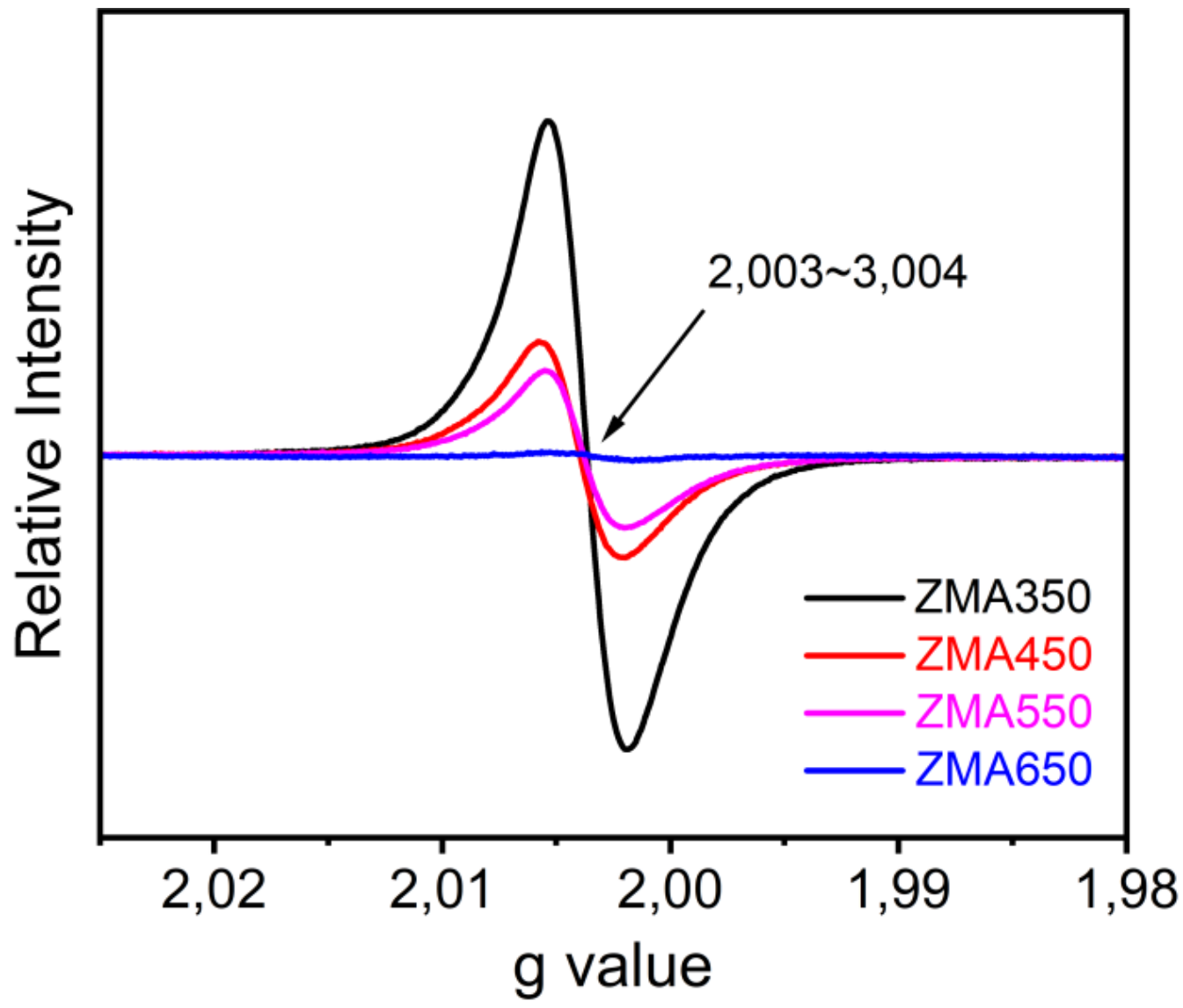

Supplementary Fig. 33 | EPR spectra of ZMA350, ZMA450, ZMA550, ZMA650 samples shown as relative intensity versus g value.

Supplementary Tab. 5 | Adsorption energy comparison (E_{bind}) of H_2S on oxygen and zinc vacancy sites.

Defects type	E_{ads}	$E_{surface}$	E_{gas}	E_{bind}
V_O	-649.49783489	-637.99504020	-11.19318950	-0.30960519

V_{Zn}	-650.35992171	-639.03633234	-11.19318950	-0.13039987

Note: $E_{bind}=E_{(surface+gas)}-E_{surface}-E_{gas}$

Supplementary Tab. 6 | Bader charge transfer for H_2S adsorption at oxygen and zinc vacancy sites.

Species	Q_S	Q_{H1}	Q_{H2}	Q_{sum}	ΔQ
H_2S @ Oxygen Vacancy	5.021889	1.509254	1.303025	7,834168	-0,165833
H_2S @ Zinc Vacancy	5.196020	1.083239	1.034088	7,862959	-0,137042
Pure H_2S	5.882674	1.368198	1.298741	8,000001	/

Note: $\Delta Q_i = Q_i^{adsorbed} - Q_i^{clean}$

Comment 5. The paper claims the sensor responds 4-5 times faster than commercial sensors with 10^5 times lower power consumption. However, Table S1 lacks specific commercial sensor models, testing conditions (e.g., humidity, interfering gas concentrations), and key parameters (e.g., typical detection limits of 0.1-1 ppm for commercial sensors). Relying solely on textual descriptions makes it impossible to verify the authenticity of these performance advantages.

Reply: We thank the reviewer for the careful assessment of the comparison with commercial H_2S sensors. We agree that such comparisons must be transparent and verifiable. Accordingly, we clarify and strengthen the benchmarking framework as follows.

First, we clarify that Supplementary Tab. 1 summarizes representative ZnO-based H_2S sensors reported in the academic literature and does not refer to specific commercial sensor products. The table caption and accompanying text have been revised to explicitly state this point and avoid ambiguity.

Second, the quantitative comparison with commercial sensors is intended as an architecture-level benchmark, rather than a one-to-one performance evaluation under identical testing conditions. Specifically, the μW-level power consumption reported in this work (0.7 μW in the active sensing state) corresponds to the heater-free sensing element operating at room temperature. For comparison with commercial products, the power consumption values (typically 25-1300 mW for representative MOS-based H_2S sensors) were extracted from publicly available manufacturer datasheets, calculated from the reported operating voltage and current ($P = V \times I$). We emphasize that commercial datasheets do not always provide a standardized or complete breakdown of the internal power budget (distinguishing between sensing element, signal conditioning, communication, or heater when present). Therefore, these reported values should be interpreted as a datasheet-verifiable, product-level benchmark, rather than implying identical testing conditions or attributing the power consumption solely to any specific sub-module.

To improve verifiability, we have added a new Table (Supplementary Tab. 8) listing representative commercial sensor models, response time, typical detection limits (where specified), datasheet-reported voltages and currents, calculated power consumption, and the corresponding datasheet sources. Since datasheets generally do not provide standardized testing conditions (e.g., humidity, interfering gas concentrations), this comparison is conservatively framed to highlight the fundamental energy-budget difference between heater-driven and heater-free sensing architectures.

Under this clearly defined and conservative framework, the comparison shows that the heater-free design presented here operates in a distinct μW power regime and exhibits response dynamics approximately 4-5× faster than values reported for commercial heated MOS H_2S sensors, as specified in publicly available datasheet specifications and literatures. The manuscript has been revised accordingly to explicitly state the scope and limitations of this comparison.

These new results and the associated discussion have been added to the Supplementary Information (Supplementary Tab. 7 and Tab. 8) and explicitly referenced in the revised manuscript (Page 17, Line 541-550) and are also highlighted in red accordingly.

Revised parts of manuscript are as following:

Benefiting from heater-free, room-temperature operation, the sensing element in this work operates in the μW power regime with an average power consumption of 0.7 μW in active mode and 0.3 μW in idle mode (details in Supplementary Tab. 7), whereas representative commercial MOS H_2S sensors typically operate in the mW regime (25-1300 mW), as derived from publicly available datasheet specifications. This architecture-level comparison highlights an energy-budget reduction of approximately five orders of magnitude.

In addition, the response dynamics are approximately 4-5× faster than those reported for commercial MOS sensors, based on datasheet values and publicly available literature. These comparisons are intended to highlight fundamental differences in sensing architecture, rather than a one-to-one performance comparison under identical testing conditions (details in Supplementary Tab. 8).

Revised parts of SI are as following:

Power consumption for the commercial sensors listed in Supplementary Tab. 8 was extracted from publicly available manufacturer datasheets. These datasheets report the overall power consumption of the device, which includes both the sensing element and any additional components such as heating elements or signal conditioning circuits. Commercial datasheets generally do not provide a full breakdown of power

consumption across individual modules (sensing element vs. heater vs. signal processing), so these values should be interpreted as a product-level benchmark rather than a direct one-to-one comparison under identical experimental conditions.

Supplementary Tab. 8 | Power Consumption and Testing Parameters for Sensor and Commercial Sensors.

Sensor Type	Model	T_{90} (s)	LOD (ppm)	Operating Voltage (V)	Current (A)	Power (W)	Power Source Breakdown	Source	Notes
**This work**	**H_2S**	**7**	**0.000328**	**3.725**	**0.1668 µA**	**0.6312 µW**	**Sensor**	**SI**	**Sensing element: µW-level, wireless + ADC = mW-level**
				**2.5**	**0.00819**	**0.0205**	**Wireless**	**SI**	**SI**
Commercial 1	SGX-4H₂S-100	30	/	1.3	1	1.3	Sensor	datasheet	W-level, heat requiremtn, heat power, etc
Commercial 2	H_2S Sensor V1.0	20	0.5	3.3-5	0.02	0.066	Sensor	datasheet	mW-level, heat requiremtn, heat power, etc
Commercial 3	2X MQ136	30	1	5	0.18	0.9	Sensor	datasheet	mW-level, heat requiremtn, heat power, etc
Commercial 4	S-H_2S-01	30	0.1	3.3-5.5	0.05	0.025	Sensor & Wire	datasheet	mW-level, heat requiremtn, heat power, wire transmission etc

Commercial Sensor Datasheet Sources:

Commercial Sensor 1: RS Components GmbH, Industrial Hydrogen Sulphide (H_2S) Sensor, Datasheet, available at [https://docs.rs-online.com/50d2/A700000009288588.pdf], accessed [21.01.2026].

Commercial Sensor 2: DFRobot, Fermion: H_2S Gas Sensor, Datasheet, available at [https://www.dfrobot.com/product-2702.html?srsltid=AfmBOoo57XTXfPiknsJnx7tR_0KkWUVBd00iB-ZgpHbOBsSuZ9ynozD1], accessed [21.01.2026].

Commercial Sensor 3: HUABAN, 1 x MQ-136 MQ136 Hydrogen Sulfide H_2S, Datasheet, available at [https://www.amazon.de/HUABAN-MQ-136-MQ136-Schwefelwasserstoff-H2S-Detektionsgassensormodul-Alarmsignallampe/dp/B089SQDQFJ?source=ps-sl-shoppingads-lpcontext&ref_=fplfs&smid=A169MKB0ILTINE&th=1], accessed [21.01.2026].

Commercial Sensor 4: Seeed Technology, S-H_2S-01, Datasheet, available at [https://files.seeedstudio.com/products/101990863/RES/RS485%20H₂S%20Sensor%20-%20Datasheet.pdf], accessed [21.01.2026].

Comment 6. BET data shows ZMA450 has the highest specific surface area among all samples, yet its sensing response is significantly lower than ZMA550. The article fails to analyze the trade-off between specific surface area, active site efficacy, and carrier mobility, leaving unexplained why high surface area did not yield high response.

Reply: We thank the reviewer for highlighting the apparent discrepancy between the specific surface area and sensing performance of ZMA450.

We agree that a larger BET surface area does not necessarily guarantee a higher sensing response in chemiresistive sensors. In the revised manuscript, we explicitly clarify that the sensing performance is governed by a trade-off among surface area, the chemical efficacy of active sites, and charge-transport efficiency. Although ZMA450 exhibits the highest specific surface area, EBSD and UPS analyses indicate that it also possesses extremely small grains, a high grain-boundary density, and elevated transport barriers, which significantly hinder carrier mobility and suppress the transduction of surface reactions into macroscopic conductance changes. In addition, an excessive density of surface sites may lead to overly strong adsorption or slow desorption of H_2S-related species, adversely affecting response-recovery behavior.

We have added a concise mechanistic discussion in the revised manuscript (Page 13, Line 395-408) and are also highlighted in red accordingly, to emphasize that optimal sensing performance arises from a balanced interplay between surface area, active-site effectiveness, and carrier transport, rather than from maximizing surface area alone. This framework consistently explains why ZMA550 outperforms ZMA450 despite a lower BET surface area.

Revised parts of manuscript are as following:

This optimized microstructure facilitates enhanced sensing performance through two synergistic mechanisms: (1) surface charge redistribution upon H_2S chemisorption, and (2) effective Schottky barrier modulation at the metal-semiconductor interface. These combined effects enable rapid modulation of electrical resistance and consequent current response. Importantly, the sensing performance does not scale monotonically with specific surface area.

Although ZMA450 exhibits the highest BET surface area, its sensing response is inferior to that of ZMA550, indicating that excessive surface area associated with ultra-fine grains does not necessarily translate into effective sensing. Excessive thermal treatment leads to excessive grain growth, resulting in two detrimental effects: (1) significant reduction in surface active sites, and (2) decreased specific surface area, as confirmed by BET measurements (Supplementary Fig. 29). This over-annealing effect ultimately degrades

the sensing performance despite improved charge transport characteristics. Together, these results suggest the existence of an optimal microstructure window, where surface area, pore accessibility, and carrier transport are balanced to maximize sensing performance.

Revised parts of SI are as following:

Nitrogen adsorption-desorption measurements were performed to investigate the structural and surface modifications induced by phase transitions of MOF-derived materials at different temperatures. Supplementary Fig. 29a displays the N_2 adsorption-desorption isotherms of as-prepared samples within a relative pressure range of 0.0-1.0. The ZMA450 sample exhibits the largest hysteresis loop area, indicating superior N_2 adsorption-desorption capacity, which correlates with its highest BET surface area (27 m^2/g) among the investigated samples. In contrast, samples prepared at lower (350 °C) and higher (650 °C) temperatures demonstrate significantly reduced N_2 adsorption-desorption capacities, corresponding to minimal BET surface areas and pore volumes (Supplementary Fig. 29b). Pore size distribution analysis is shown in Supplementary Fig. 29c. Notably, despite its highest BET surface area, ZMA450 exhibits a relatively narrow and monomodal pore-size distribution centered at ~20 nm, which may limit effective gas diffusion and active-site accessibility under dynamic sensing conditions. In contrast, ZMA550 features a bimodal mesoporous structure with larger characteristic pore sizes (~40 and 70 nm), which is more favorable for rapid gas transport and efficient utilization of surface active sites. These structural differences help rationalize why ZMA550 outperforms ZMA450 in sensing performance despite a lower overall BET surface area. ZMA350 and ZMA650 show negligible well-defined porosity, indicating collapsed pore structures.

Comment 7. Performance testing was conducted only at ~15% RH. The impact of high humidity (e.g., RH>60%, common in food refrigeration and industrial damp environments) and temperature fluctuations (e.g., -10~40 °C) on sensor response was not evaluated.

Reply: We thank the reviewer for highlighting the importance of humidity and temperature effects under realistic operating conditions.

Regarding humidity, we conducted systematic humidity-dependent sensing measurements by exposing the ZMA550 sensor to 1 ppm H_2S under controlled relative humidity levels of 15%, 30%, 50%, 70%, and 90% RH. The humidity was continuously monitored using a calibrated Bosch BME280 sensor, and a schematic of the humidity-controlled sensing setup has been added (Supplementary Fig. 8). The response-recovery curves demonstrate that the sensor maintains a clear and reversible response to H_2S across the entire

humidity range, indicating robust sensing behavior under varying ambient humidity conditions (Supplementary Fig. 9).

In addition, as part of addressing a related comment, we further performed post-operation XPS analysis of the sensing layers to examine possible humidity-induced surface changes. These results are included in the revised Supplementary Information and indicate that the underlying sensing mechanism remains intact under humid conditions (Supplementary Fig. 10). We note that this XPS analysis serves as supporting evidence and does not alter the primary conclusion regarding humidity robustness.

Regarding temperature, although the device is designed for room-temperature operation, we further evaluated its response under representative temperatures spanning a practical window (e.g., -10-40 °C) while keeping the gas concentration and humidity controlled (Supplementary Fig. 11). The sensor remains responsive and reversible across this range, with temperature mainly influencing the response/recovery kinetics and baseline conductance, consistent with thermally activated surface reaction and transport processes. These results support the robustness of the sensor under moderate temperature fluctuations relevant to real-world monitoring scenarios.

These new results and the associated discussion have been added to the Supplementary Information (Supplementary Fig. 8-11) and explicitly referenced in the revised manuscript (Page 8, Line 241-243) and are also highlighted in red accordingly.

Revised parts of manuscript are as following:

The sensor maintains a stable and reversible response to H_2S across a wide humidity range (15-90% RH) (Supplementary Fig. 8-10) and representative temperatures from -10 to 40 °C (Supplementary Fig. 11), demonstrating robust performance under realistic conditions.

Revised parts of SI are as following:

The humidity was continuously monitored using a calibrated Bosch BME280 sensor, and a schematic of the humidity-controlled sensing setup has been added (Supplementary Fig. 8). It is worth noting that the response amplitude of the sensor to 1 ppm H_2S exhibits a gradual increase with increasing relative humidity, as consistently observed over repeated response-recovery cycles at each humidity level (Supplementary Fig. 9). This trend suggests that moderate humidity may facilitate the surface reaction kinetics or charge transfer processes involved in H_2S sensing.

Importantly, despite the humidity-enhanced response, XPS analysis of the sensing layers after operation under different humidity conditions reveals no significant variation in the relative contributions of lattice

oxygen (O_I) and hydroxyl-related oxygen species (O_{III}) (Supplementary Fig. 10). This indicates that the increased response is not associated with substantial modification of surface hydroxyl coverage or irreversible water-induced surface reconstruction. Instead, humidity acts as a secondary modulating factor that influences the magnitude of the sensing response without altering the underlying Schottky-barrier-modulated sensing mechanism.

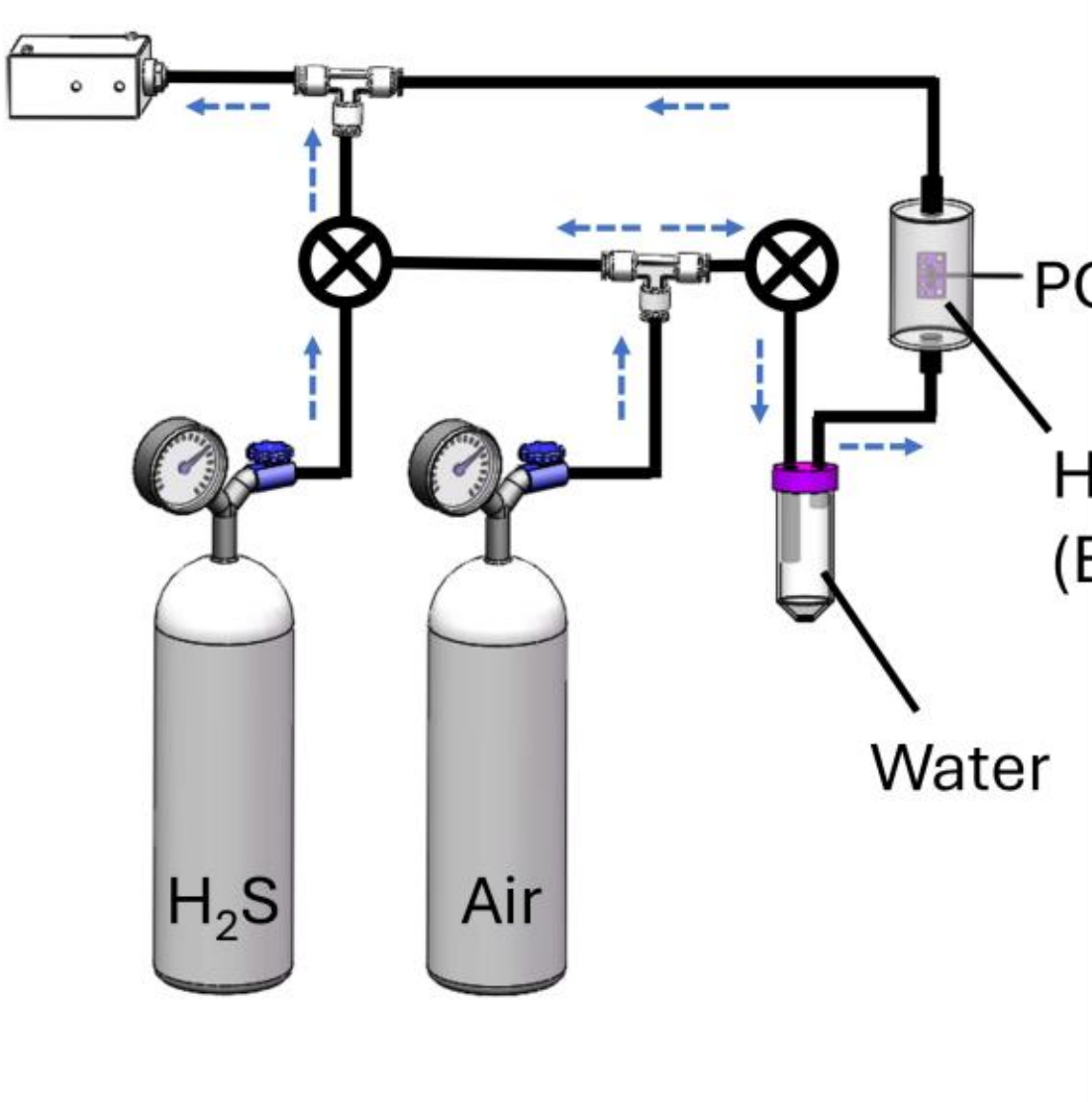

Supplementary Fig. 8 | Schematic diagram of humidity introduction in sensing tests.

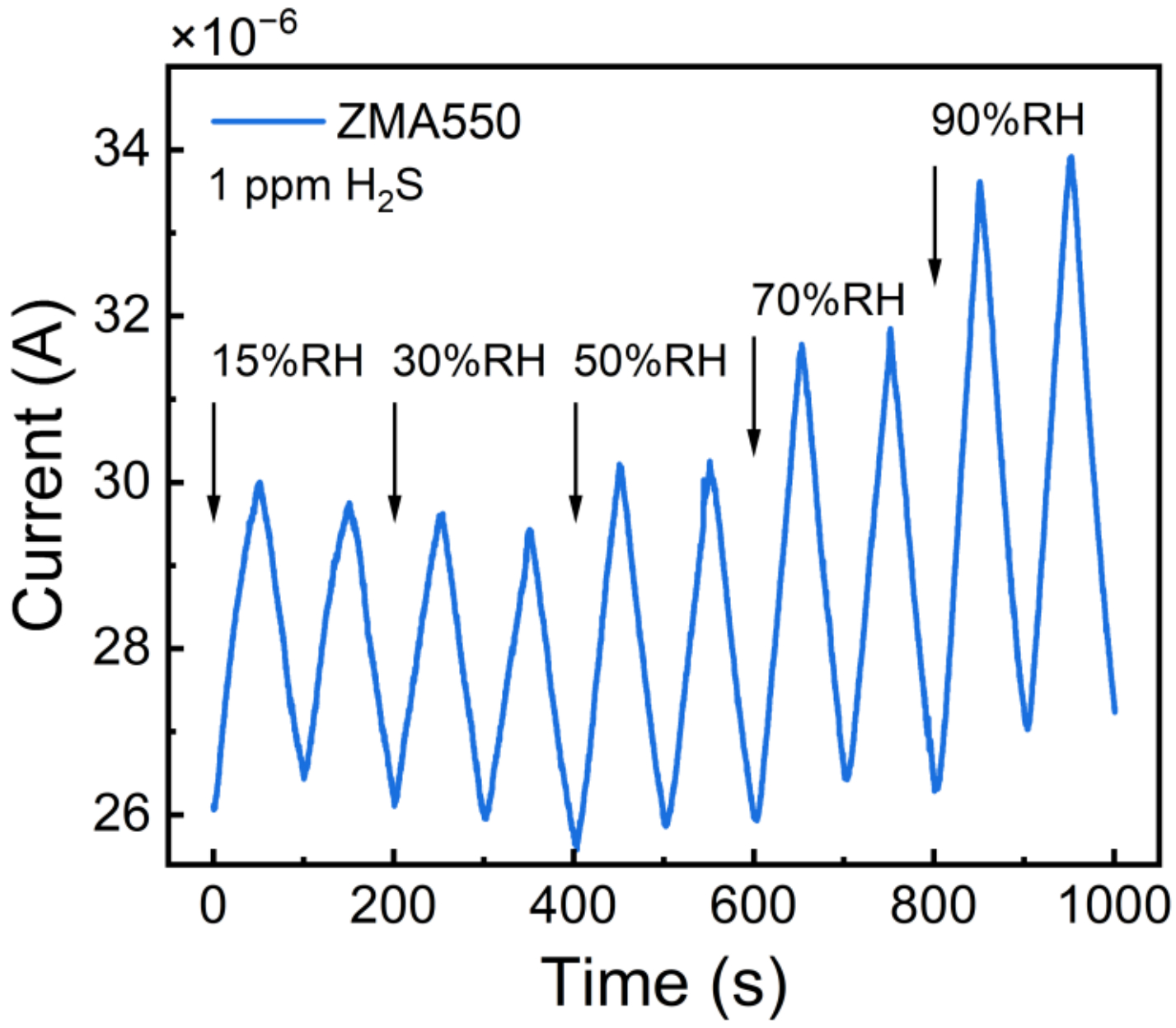

Supplementary Fig. 9 | Current response and recovery curves of ZMA550 sensor towards 1 ppm H_2S at different humidity levels from 15%RH-90%RH.

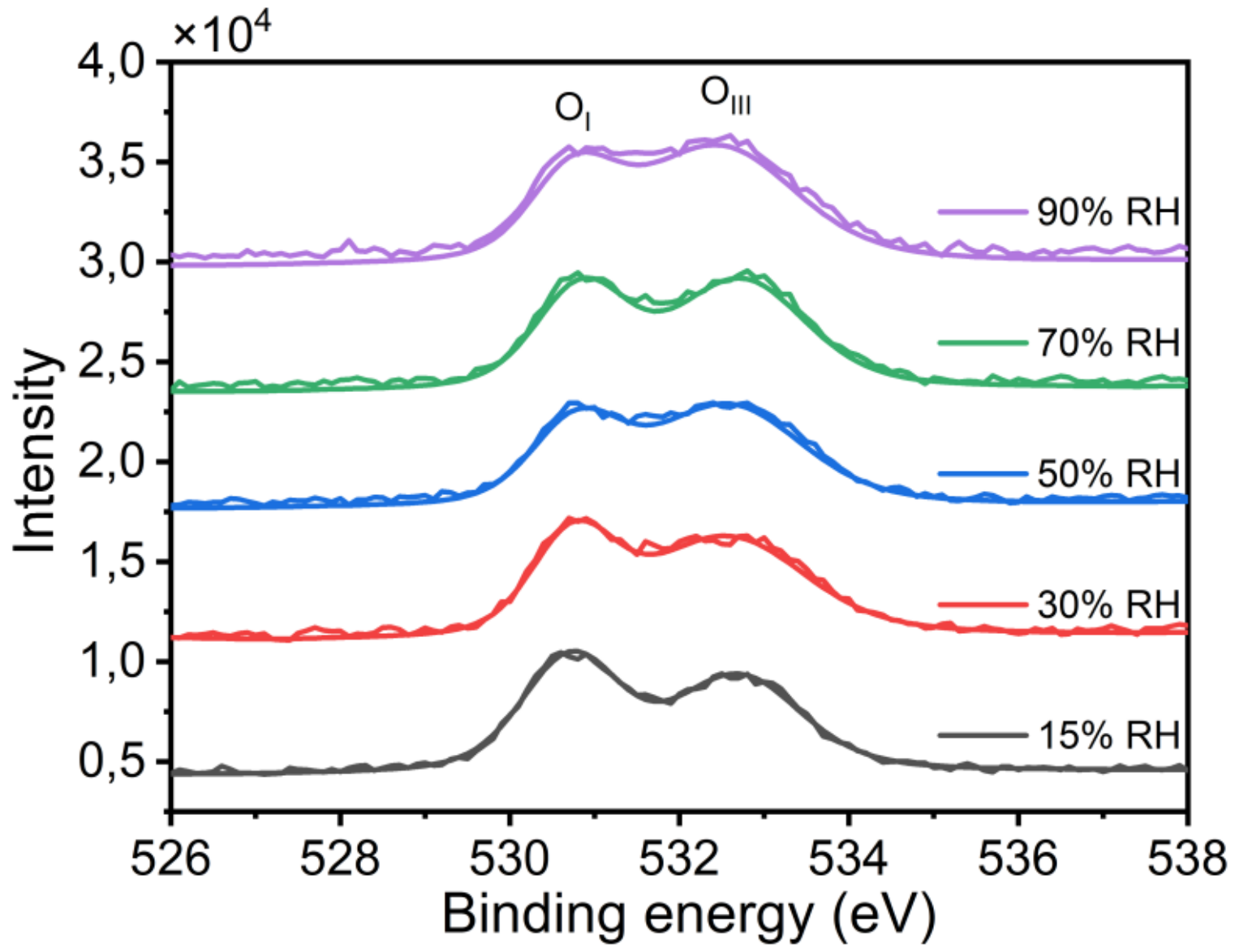

Supplementary Fig. 10 | O 1s XPS results of sensing tests under different humidity levels.

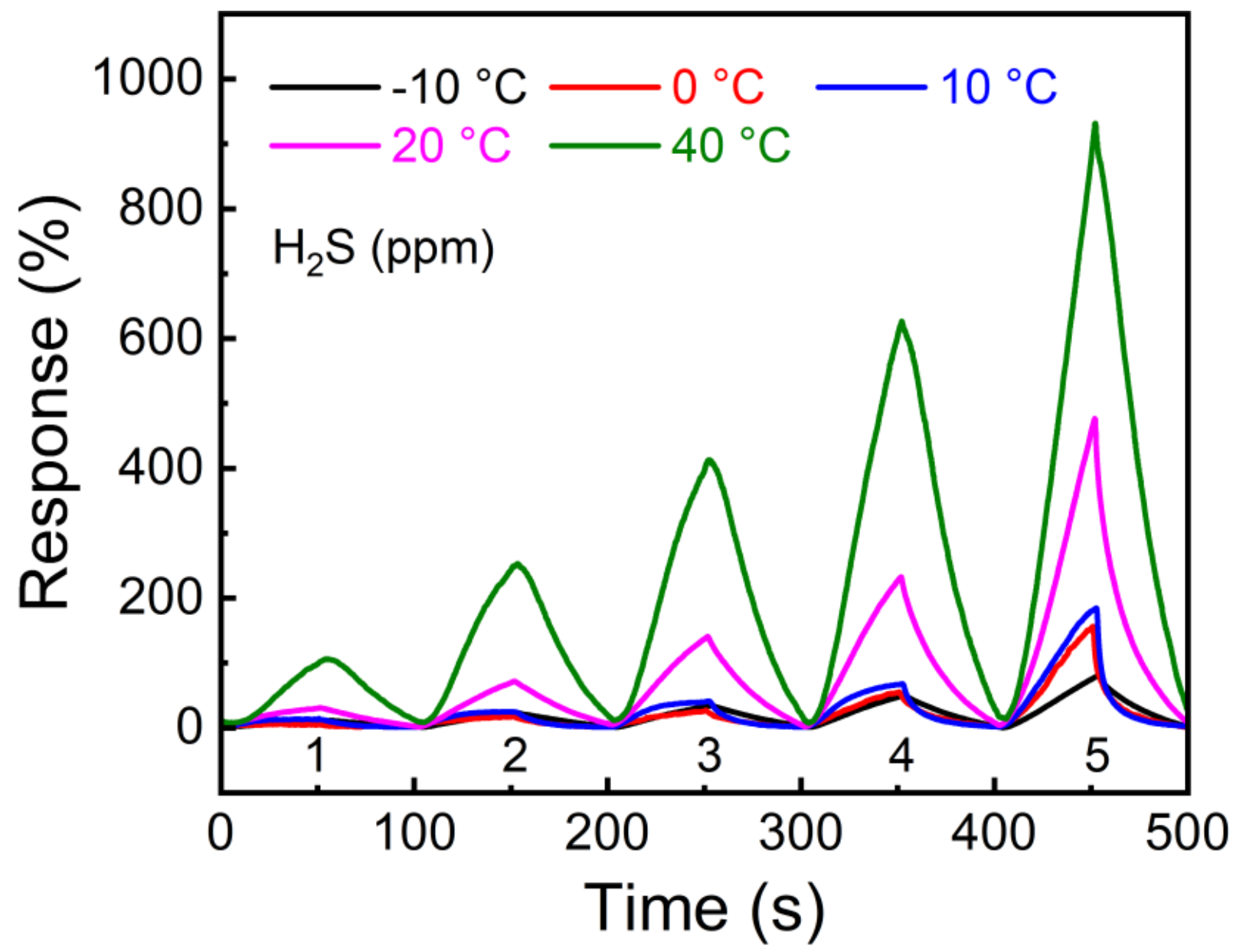

Supplementary Fig. 11 | Response and recovery curves of ZMA550 sensor exposed to varying high H_2S concentrations (1-5 ppm) under different operation temperature.

To Reviewer: 3

General comments: The manuscript presents an ultra-low-power wireless H_2S sensor based on MOF-derived ZnO with engineered grain boundaries and Au/ZnO Schottky interfaces, enabling exceptional room-temperature sensitivity, a 0.328-ppb detection limit, and a rapid 7-second response. Comprehensive structural, electronic, and theoretical analyses validate the synergistic sensing mechanism, while real-world demonstrations- such as food spoilage detection, pipeline leakage monitoring, and confined-space safety-highlight the device's practical applicability. Besides these, there are still some aspects of the detection methods and characterisations that need clarification; therefore, this paper may require major revision. We hope that the authors will give serious consideration to comments or suggestions.

Reply: We sincerely thank the reviewer for the positive and encouraging evaluation of our work, and for recognizing both the ultralow-power performance and the broad real-world applicability of our MOF-derived ZnO wireless H_2S sensor. We appreciate your acknowledgement of the comprehensive structural, electronic, and theoretical analyses supporting the synergistic sensing mechanism, as well as the effectiveness of our demonstrations in practical scenarios such as food spoilage monitoring, pipeline leakage detection, and confined-space safety.

At the same time, we fully understand the reviewer's concerns regarding aspects of the detection methodology and characterization that require further clarification. Your constructive suggestions have been extremely valuable in helping us strengthen the rigor, transparency, and completeness of the manuscript. In the revised version, we have carefully addressed all of your comments in a detailed point-by-point response that thoroughly explains how each issue has been resolved. All modifications made to the manuscript are highlighted in red for ease of inspection. We greatly appreciate your thoughtful feedback, which has significantly improved the overall quality and clarity of our study.

Comment 1. The authors claim an optimal grain boundary density yields peak performance. What is the correlation between grain boundary density (from TEM/BET) and charge transport parameters? Can you provide a direct measurement (e.g., EIS fitting, mobility estimations) linking grain boundary density to transport and sensing response?

Reply: We thank the reviewer for the insightful question regarding the correlation between grain-boundary density and charge transport, and for suggesting direct transport-related measurements.

We agree that establishing a link between grain-boundary density and charge-transport parameters is essential. To this end, we explored multiple experimental approaches. Electrochemical impedance spectroscopy (EIS) measurements were initially performed; however, for the highly resistive powder-based sensing layers investigated here, the impedance spectra did not exhibit resolvable semicircular features associated with bulk or grain-boundary conduction. Attempts to improve current collection by introducing an electrolyte resulted in spectra dominated by the electrolyte response rather than the intrinsic transport properties of the sensing material. These results indicate that conventional EIS fitting is not suitable for quantitatively resolving grain-boundary-controlled transport in this system.

Instead, we employed four-probe DC resistivity measurements, which provide a direct and quantitative descriptor of charge transport in polycrystalline materials. The resistivity was found to vary systematically with grain-boundary density, revealing a clear correlation between increased boundary density and suppressed electrical conductivity. In parallel, EBSD analysis was performed to quantify the grain-boundary density of samples prepared at different annealing temperatures. By correlating the EBSD-derived grain-boundary density with the measured resistivity and sensing response, we demonstrate that an intermediate grain-boundary density yields an optimal balance between surface reactivity and charge transport, leading to peak sensing performance. In addition, as detailed in response to a related comment, EBSD and UPS analyses independently reveal a systematic correlation between grain size, grain-boundary density, and Schottky barrier height, further supporting the grain-boundary-controlled transport picture.

These results establish a direct experimental link between grain-boundary density, charge-transport limitation, and sensing response, thereby supporting the conclusion that an optimal grain-boundary density—rather than a monotonic increase—is responsible for the observed performance maximum.

These new results and the associated discussion have been added to the Supplementary Information (Supplementary, Fig. 5, Fig. 21, Fig. 34 and Tab. 3) and explicitly referenced in the revised manuscript (Page 11, Line 327-330) and are also highlighted in red accordingly.

Revised parts of manuscript are as following:

As detailed in Supplementary Fig. 21, the direct transport measurements, together with EBSD-derived grain-boundary statistics and correlation analysis with sensing response, establish a quantitative link between grain boundary density, charge transport, and sensing performance.

Revised parts of SI are as following:

To directly link grain-boundary density to charge transport and sensing performance, we combined impedance characterization, four-probe resistivity measurements, and EBSD-based grain-boundary statistics. Electrochemical impedance spectroscopy (EIS) of the powder-based sensing layers does not show a resolvable semicircle and is dominated by a linear response, indicating that conventional EIS fitting cannot reliably extract intrinsic grain/boundary resistance components in this highly resistive system (Supplementary Fig. 34). We therefore employ four-probe resistivity as a direct transport descriptor, which are widely used as a direct and quantitative descriptor of electrical transport in solids [46-47]. The resistivity exhibits a non-monotonic dependence on pyrolysis temperature, decreasing first and then increasing, with ZMA550 showing the lowest resistivity among all samples (Supplementary Fig. 5).

To correlate microstructure with transport and sensing, EBSD statistics were used to estimate grain size and grain-boundary density. For samples with extremely fine grains (notably ZMA450 and other low-temperature samples), the EBSD-based boundary quantification shows large uncertainty and is thus not included in the quantitative correlation plot. Accordingly, the correlation analysis focuses on ZMA500, ZMA550, ZMA600, and ZMA650, where EBSD statistics are reliable (Supplementary Fig. 20-21 and Tab. 3). Across these samples, increasing pyrolysis temperature results in grain growth and reduced grain-boundary density, accompanied by a non-monotonic evolution of resistivity (minimum at ZMA550) and a corresponding non-monotonic sensing response (maximum at ZMA550), highlighting an optimal grain-boundary density window that balances charge transport and sensing transduction (Supplementary Fig. 21).

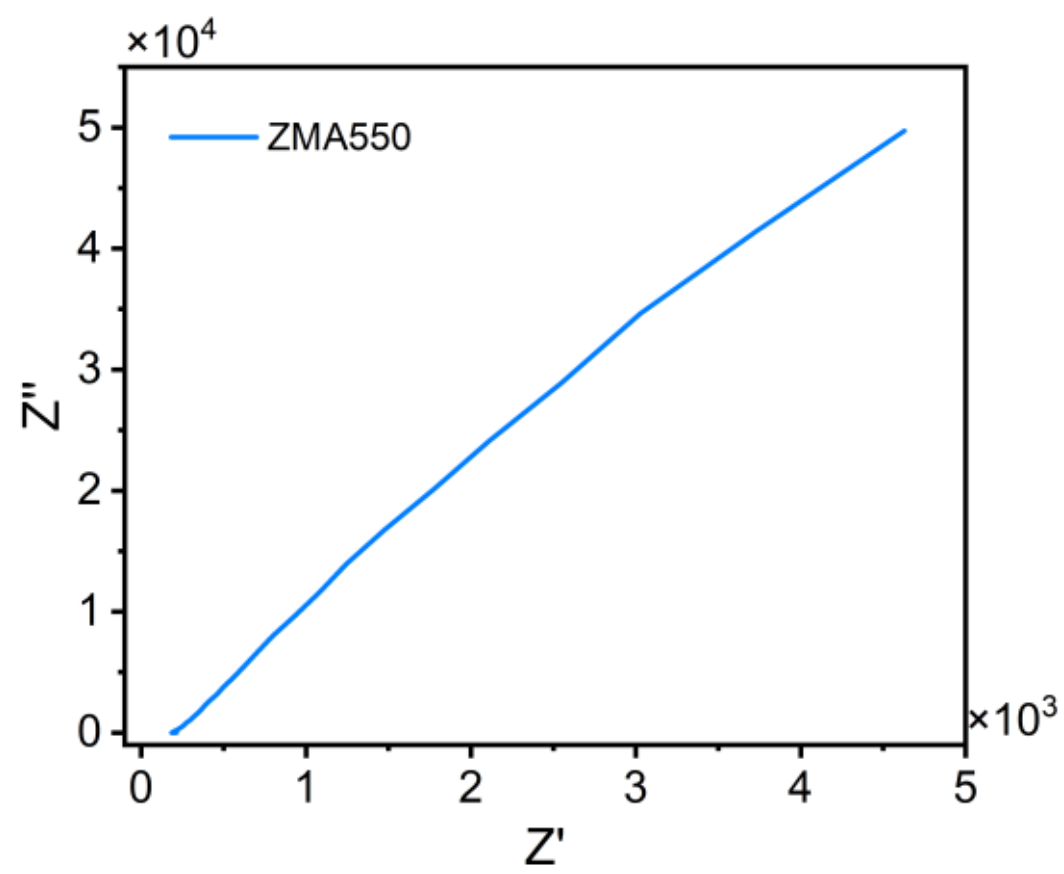

Supplementary Fig. 34 | Electrochemical impedance spectroscopy (EIS) of the ZMA550.

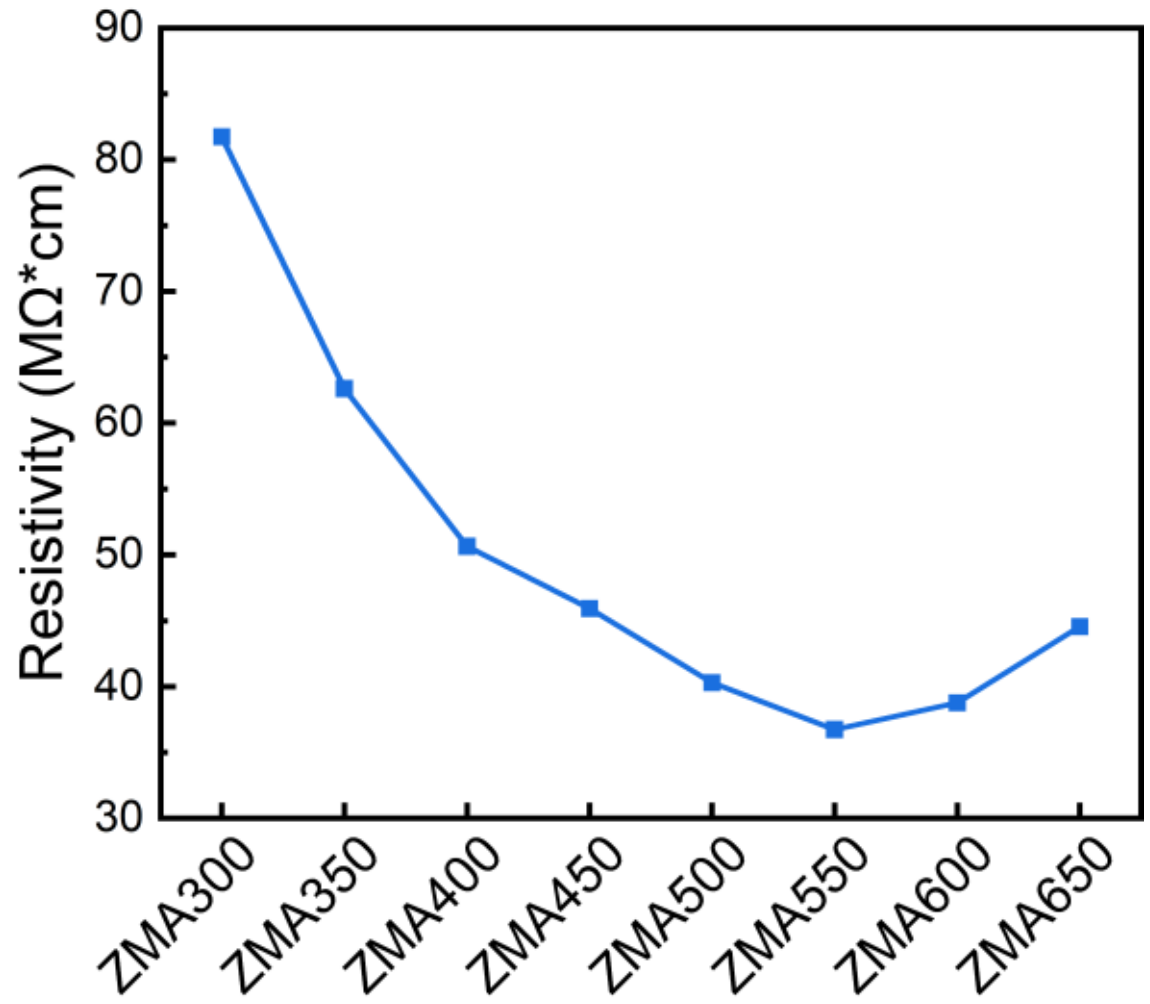

Supplementary Fig. 5 | Electrical resistivity of all as-prepared samples.

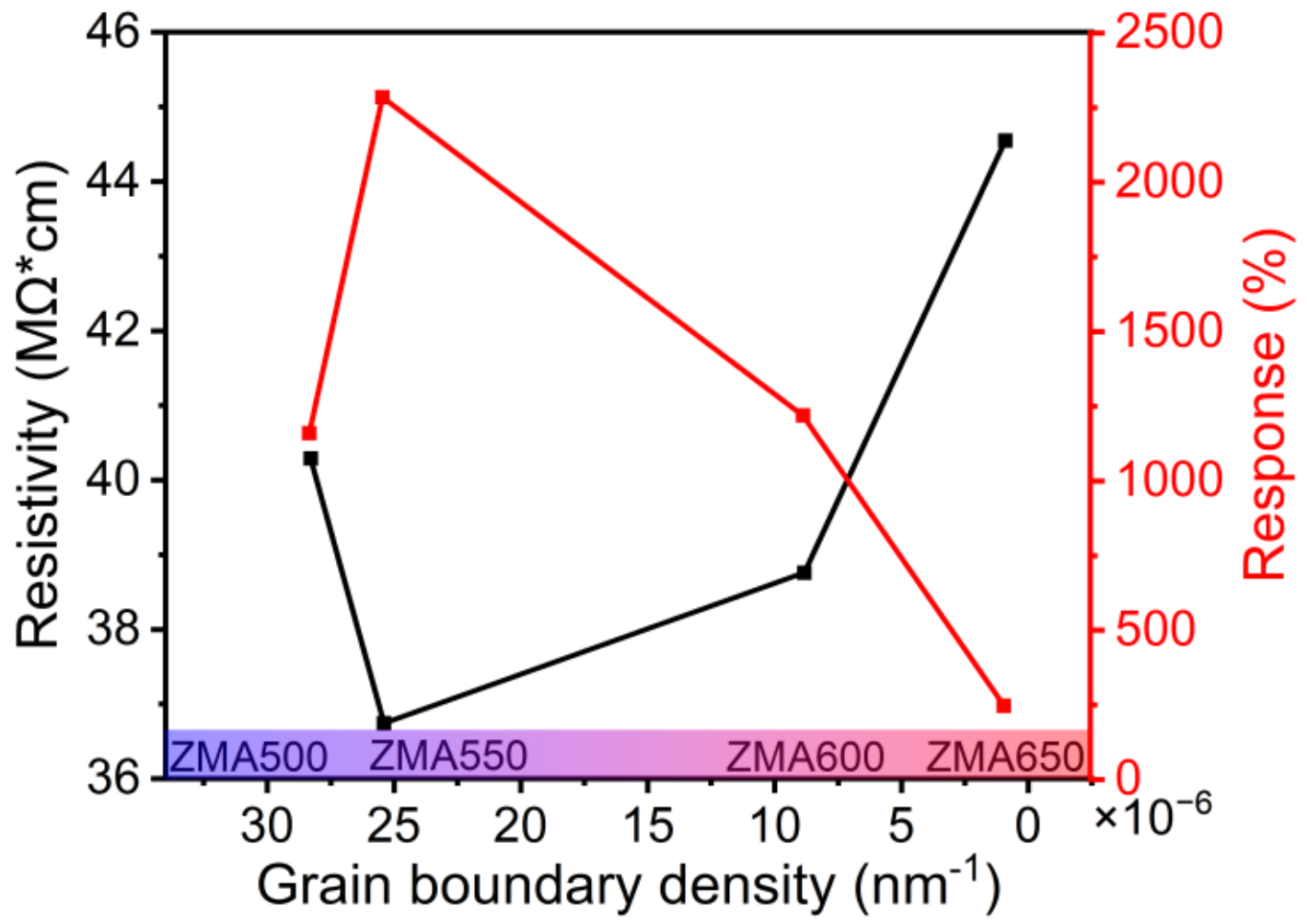

Supplementary Fig. 21 | Correlation between grain-boundary density, electrical resistivity, and H_2S sensing response.

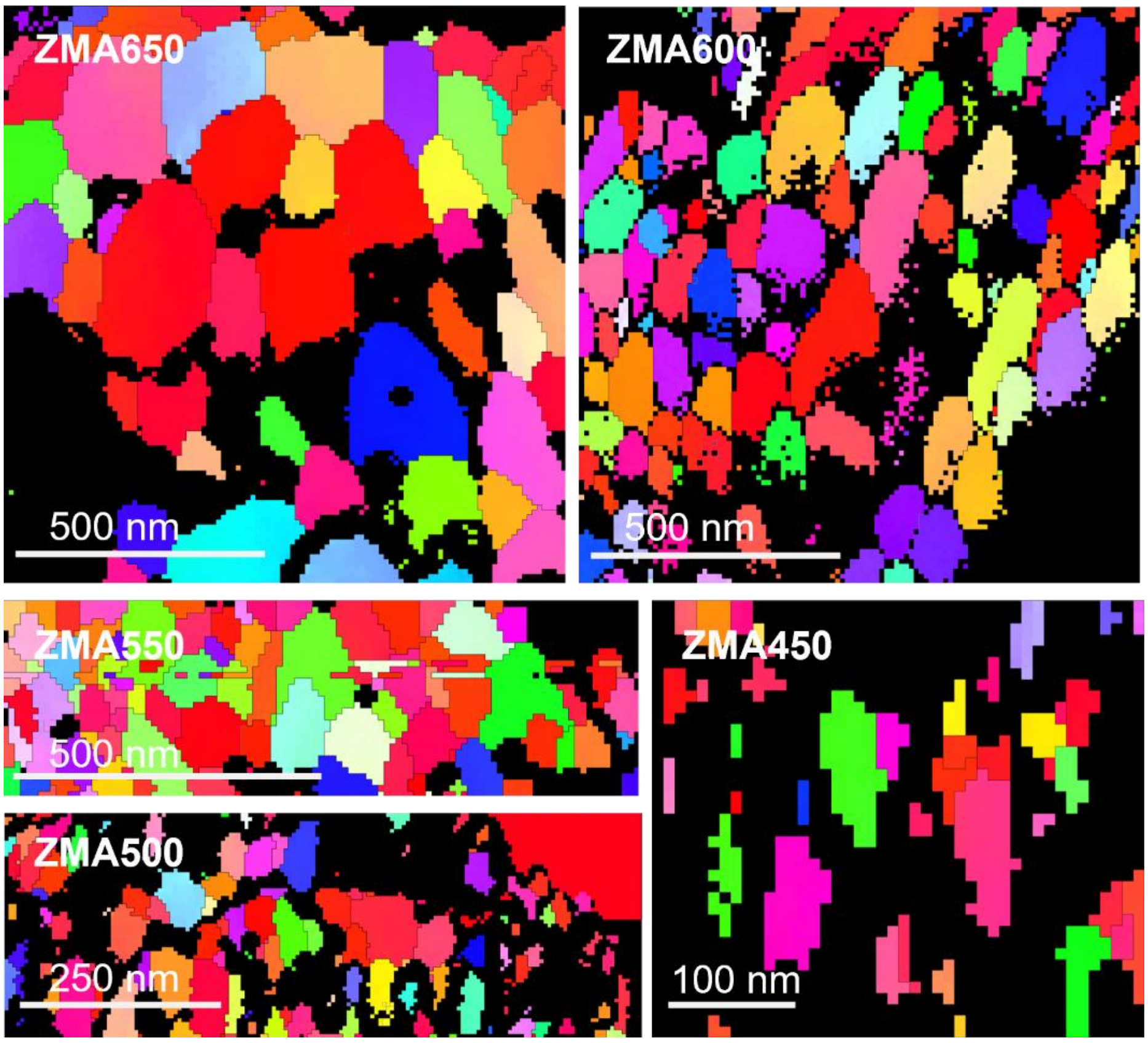

Supplementary Fig. 20 | EBSD images of samples pyrolyzed at 450–650 °C.

Supplementary Tab. 3 | Grain boundary concentration and density of samples pyrolyzed at 500–650 °C.

Samples	Total length of GB/nm	Area /nm^2	GB density/nm^{-1}
ZMA 500	6.08148	215024.9500	2.82827×10^{-5}
ZMA 550	10.14060	399185.0975	2.54033×10^{-5}
ZMA 600	11.96009	1355545.3960	8.82308×10^{-6}
ZMA 650	11.50177	12590887.6200	9.13500×10^{-7}

Comment 2. They attribute the exceptional sensing performance to a ~0.4 eV reduction in the Au/ZnO Schottky barrier. How was the barrier change extracted from UPS, and can alternative explanations (surface dipoles, interface states, defect redistribution) be excluded?

Reply: We thank the reviewer for the question regarding how the Au/ZnO Schottky barrier change was extracted from UPS and whether alternative explanations can be excluded. In the revised manuscript and Supplementary Information, we clarify the quantitative procedure and the scope of the UPS analysis.

Upon H_2S exposure, the work function decreases from 4.3 to 3.9 eV, while the valence-band onset shifts from 2.6 to 3.0 eV (Fig. 3e–f). As a result, the ionization potential, defined as IP = WF + ($E_F - E_{VBM}$), remains constant at 6.9 eV before and after H_2S exposure. This indicates that H_2S adsorption primarily induces a redistribution of surface band alignment and vacuum-level shift, rather than altering the intrinsic electronic structure of the material. Using the ZnO optical bandgap E_g=4.07 eV (Supplementary Fig. 17), the electron affinity can be estimated as χ=IE−E_g, giving $\chi\approx$2.83 eV for both states. The effective electron barrier at the Au/ZnO junction is then evaluated using a standard Au/ZnO contact model, where the operating-state barrier is governed by the relative alignment between the metal Fermi level and the semiconductor bands. Importantly, the extracted ~0.4 eV barrier reduction refers to the effective interfacial barrier change under sensing conditions, consistent with the concurrent UPS shifts (WF and VB_{onset}) and the device-level conductance modulation.

We further assessed potential alternative origins. (i) Interface states: The valence-band spectra plotted on a semi-log scale do not show pronounced additional in-gap tails or emergent valence-band components beyond those attributable to ZnO and Au, arguing against strong interface-state formation as the dominant cause of the observed energetics shift. (ii) Surface dipoles: We acknowledge that surface dipoles can shift the vacuum level (and thus WF). However, the simultaneous shift in the valence-band onset together with the WF shift indicates that the H_2S exposure not only modifies the vacuum level but also changes the band alignment relative to E_F, consistent with charge redistribution/band-bending modulation that directly impacts the effective Au/ZnO barrier. While UPS does not provide the microscopic origin of the vacuum level shift, it gives absolute energetic positions, thus supporting the observed trend. (iii) Defect redistribution: Any defect-chemistry contribution would need to produce comparable and systematic shifts in both WF and band-edge positions; our measurements show reversible electronic-structure shifts during sensing, supporting barrier modulation as the primary transduction pathway.

These new results and the associated discussion have been added to the Supplementary Information (Supplementary Fig. 18 and Tab. 2) and explicitly referenced in the revised manuscript (Page 10, Line 296-299) and are also highlighted in red accordingly.

Revised parts of manuscript are as following:

A quantitative analysis combining UPS data and the optical bandgap of ZnO (E_g, Supplementary Fig. 17) reveals a 0.4 eV decrease in the barrier height (details shown in Supplementary Fig. 18 and Tab. 2), highlighting the strong influence of surface electronic structure modulation on the Au-ZnO contact.

Revised parts of SI are as following:

UPS observables and how they are extracted:

Ultraviolet photoelectron spectroscopy (UPS, He I, $h\nu$ = 21.22 eV) provides two key observables for the ZnO sensing layer:

(i) Work function (WF, Φ) from the secondary-electron cutoff

$$\Phi = h\nu - (E_{cutoff} - E_F)$$

where E_{cutoff} is the secondary-electron cutoff energy and E_F is the Fermi level (set to 0 eV in the UPS spectra).

(ii) Valence band onset relative to E_F

The valence band onset is determined by linear extrapolation of the leading edge of the valence band:

$$\Delta E_V = E_F - E_{VBM}$$

where E_{VBM} is the valence band maximum (VBM). In this work, ΔE_V corresponds to the reported VB_{onset} values in Fig. 3f.

From Fig. 3e-f, the measured values are:

- Before H_2S exposure: Φ=4.3 eV; ΔE_V=2.6 eV
- After H_2S exposure: Φ=3.9 eV; ΔE_V=3.0 eV

These two UPS observables together constrain both the vacuum-level position (via Φ) and the band-edge position relative to E_F (via ΔE_V).

Band-edge positions and the "effective barrier" on the ZnO side:

For an n-type semiconductor, the energy separation between the conduction band minimum (CBM) and the Fermi level at/near the surface is:

$$\Delta E_C = E_{CBM} - E_F$$

Using the optical bandgap of ZnO (taken as E_g=4.07 in this work), and the UPS-derived $\Delta E_V = E_F - E_{VBM}$, we obtain:

$$E_{CBM} - E_F = E_g - (E_F - E_{VBM}) = E_g - \Delta E_V$$

Therefore:

Before H_2S exposure:

$$\Delta E_C^{before} = 4.07 - 2.6 = 1.47 \text{ eV}$$

After H_2S exposure:

$$\Delta E_C^{after} = 4.07 - 3.0 = 1.07 \text{ eV}$$

Thus, the surface/near-interface electron barrier on the ZnO side decreases by:

$$\Delta(\Delta E_C)=1.47-1.07=0.4\ eV$$

$\Delta E_C=E_{CBM}-E_F$ is a direct energetic descriptor for electron depletion at the ZnO surface/near-interface region: a smaller ΔE_C implies a less depleted (more electron-rich) near-surface region, a narrower depletion width, and a reduced energetic penalty for interfacial electron transport. Under operating conditions of a metal/n-ZnO junction, this reduction manifests as an effective lowering of the interfacial transport barrier, consistent with the observed conductance increase upon H_2S exposure. The reported 0.4 eV barrier reduction is extracted from the band-edge alignment change relative to E_F (via ΔE_V and E_g), which is from the ZnO-side depletion/transport barrier, rather than relying solely on an ideal Schottky-Mott absolute barrier value.

Assessment of alternative explanations:

(i) Interface states

To assess whether new interfacial electronic states dominate the energetics shift, the valence-band spectra were inspected on a semi-log scale (Supplementary Fig. 18 and Tab. 2). No pronounced additional in-gap tailing or emergent valence-band components beyond those attributable to ZnO and Au are observed. This argues against strong interface-state formation as the primary origin of the barrier modulation inferred from ΔE_V. However, we acknowledge that interface states may still play a minor role in the overall energetics shift.

(ii) Surface dipoles

Surface dipoles can shift the vacuum level and therefore modify the work function (Φ). However, a pure dipole-driven vacuum-level shift would not necessarily produce a systematic change in $\Delta E_V=E_F-E_{VBM}$. In contrast, in this work ΔE_V changes by 0.4 eV (2.6→3.0 eV), which directly alters $\Delta E_C=E_{CBM}-E_F$ and yields the extracted 0.4 eV effective barrier reduction. Therefore, while dipoles may contribute to the work function shift, the band-edge alignment change relative to E_F indicates that charge redistribution and band-bending modulation accompany any vacuum-level effect and directly impact the effective transport barrier under sensing conditions. While UPS does not provide the microscopic origin of the vacuum level shift, it does give absolute energetic positions, supporting the observed shift in barrier modulation.

(iii) Defect redistribution

Defect-related surface chemistry may influence the magnitude of the UPS shifts. Nevertheless, the key quantity used to quantify the effective barrier modulation here is $\Delta E_C=E_g-\Delta E_V$, derived from the band-edge position relative to E_F. The concerted, systematic shifts in UPS observables before/after gas exposure support

a barrier-modulation scenario consistent with the device response, rather than requiring a dominant irreversible defect redistribution mechanism. Any defect-chemistry contribution would need to produce comparable and systematic shifts in both work function and band-edge positions, which is not observed in the current system.

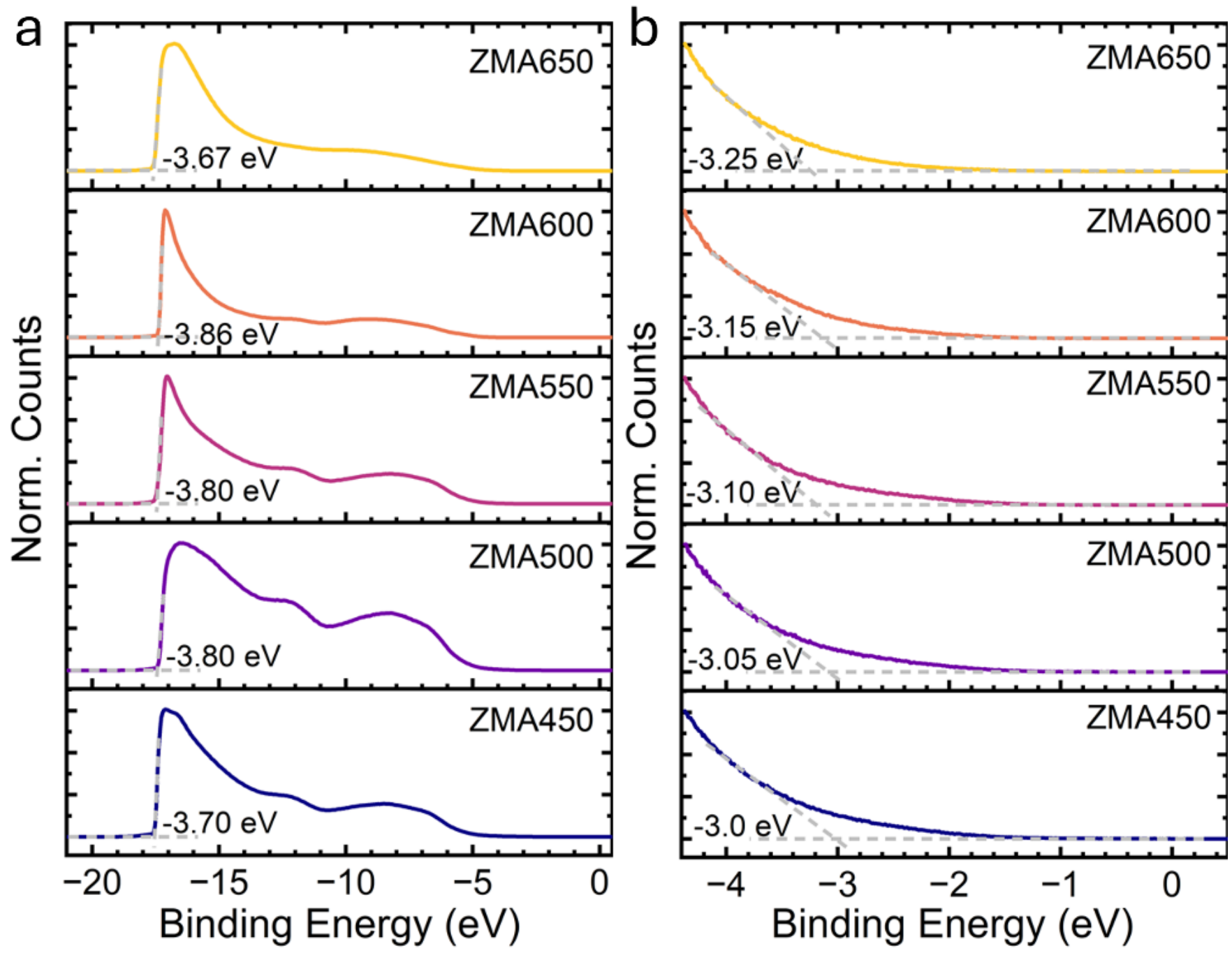

Supplementary Fig. 18 | a, UPS spectra in the secondary electron cutoff region for work-function determination (by linear extrapolation of the cutoff edge). **b,** UPS valence-band spectra showing the valence-band onset for samples pyrolyzed at 450-650 °C after H_2S sensing tests.

Supplementary Tab. 2 | Comparison of barrier parameters for samples pyrolyzed at 450-650 °C after H_2S sensing tests.

Samples	φ_{Au} (eV)	φ_{ZnO} (eV)	VB_{onset} (eV)	Barrier (eV)
ZMA 450		3.70	3.00	1.07
ZMA 500		3.80	3.05	1.02
ZMA 550	4.7	3.80	3.10	0.97
ZMA 600		3.86	3.15	0.92
ZMA 650		3.67	3.25	0.82

Comment 3. The sensing mechanism heavily relies on oxygen vacancies. Is there direct evidence (e.g., EPR, photoluminescence spectroscopy) confirming vacancy concentration trends with temperature beyond XPS deconvolution?

Reply: We sincerely thank the reviewer for requesting direct evidence of oxygen-vacancy trends beyond XPS deconvolution. In response, we performed EPR measurements on the samples as an independent probe of defect-related paramagnetic centers. All samples exhibit a clear resonance at g ≈ 2.003-2.004, which is characteristic of defect-related unpaired electrons in ZnO and is commonly associated with oxygen-vacancy-related paramagnetic centers. Importantly, while the g value confirms the defect type, the relative defect population is reflected by the EPR signal intensity (evaluated from the peak-to-peak amplitude or integrated intensity under identical measurement settings). We observe a monotonic decrease of the EPR signal intensity with increasing annealing/pyrolysis temperature, providing direct spectroscopic evidence that vacancy-related defect centers progressively diminish at higher temperatures.

In addition, to place the defect trend in the context of device performance, we note that the sensing response is determined not only by defect chemistry but also by transport properties. Therefore, complementary microstructural/transport characterizations (EBSD statistics and resistivity measurements) are provided in the Supplementary Information (Supplementary Fig. 21) to support the overall structure-transport-function relationship.

These new results and the associated discussion have been added to the Supplementary Information (Supplementary Fig. 33) and explicitly referenced in the revised manuscript (Page 14, Line 440-453) and are also highlighted in red accordingly.

Revised parts of manuscript are as follows:

Additionally, the electron paramagnetic resonance spectroscopy (EPR) measurements were performed to directly assess the oxygen-vacancy concentration trends in the samples (g ≈ 2.003-2.004) and reveal a monotonic decrease in signal intensity with increasing annealing temperature, and full data and analysis are given in Supplementary Fig. 33.

Additionally revised parts of manuscript are as follows:

To further investigate the influence of defects on sensing performance, we performed DFT calculations to evaluate the adsorption of H_2S at both oxygen and zinc vacancy sites. The results confirm that oxygen vacancies play a more significant role in H_2S adsorption, and full data and analysis are given in Supplementary Fig. 32 and Tab. 5-6.

The aforementioned surface reactions induce substantial modifications in the electronic structure of ZnO, altering the surface charge distribution and bulk carrier concentration. XPS, UV-vis, and EPR analyses collectively indicate that oxygen-vacancy-related defects represent a dominant class of surface-active sites

in the ZnO matrix, giving rise to a Zn-rich and non-stoichiometric surface. Such defect-rich surfaces exhibit enhanced Lewis acidity and facilitate H_2S adsorption and activation at the chemical level. However, as demonstrated by the comparative analysis of ZMA450 and ZMA550, the overall sensing performance is ultimately governed by the coupled effects of defect chemistry and charge-transport efficiency.

Revised parts of SI are as follows:

EPR was employed as an independent probe to validate defect-related signals beyond XPS deconvolution. All samples exhibit a distinct EPR resonance at g ≈ 2.003-2.004, which is characteristic of defect-related unpaired electrons in ZnO and is commonly associated with oxygen-vacancy-related paramagnetic centers. The g values remain within a narrow range across samples, indicating that the dominant paramagnetic defect type is comparable, whereas the relative defect population is reflected by the signal intensity.

To quantify the temperature-dependent trend, the EPR intensity of the g ≈ 2.003-2.004 feature was evaluated under identical measurement conditions (microwave power, modulation amplitude, receiver gain; intensity normalized by sample mass). The results show that the EPR intensity decreases monotonically with increasing pyrolysis temperature, indicating a progressive reduction of vacancy-related paramagnetic centers at higher temperatures. This behavior is consistent with thermally assisted defect relaxation and enhanced oxygen re-coordination during high-temperature treatment. Together with the microstructure/transport results in the Supplementary Fig. 21, the EPR results support the conclusion that vacancy-related defects contribute to surface activation, whereas peak sensing performance requires an optimal balance between defect density and charge-transport efficiency.

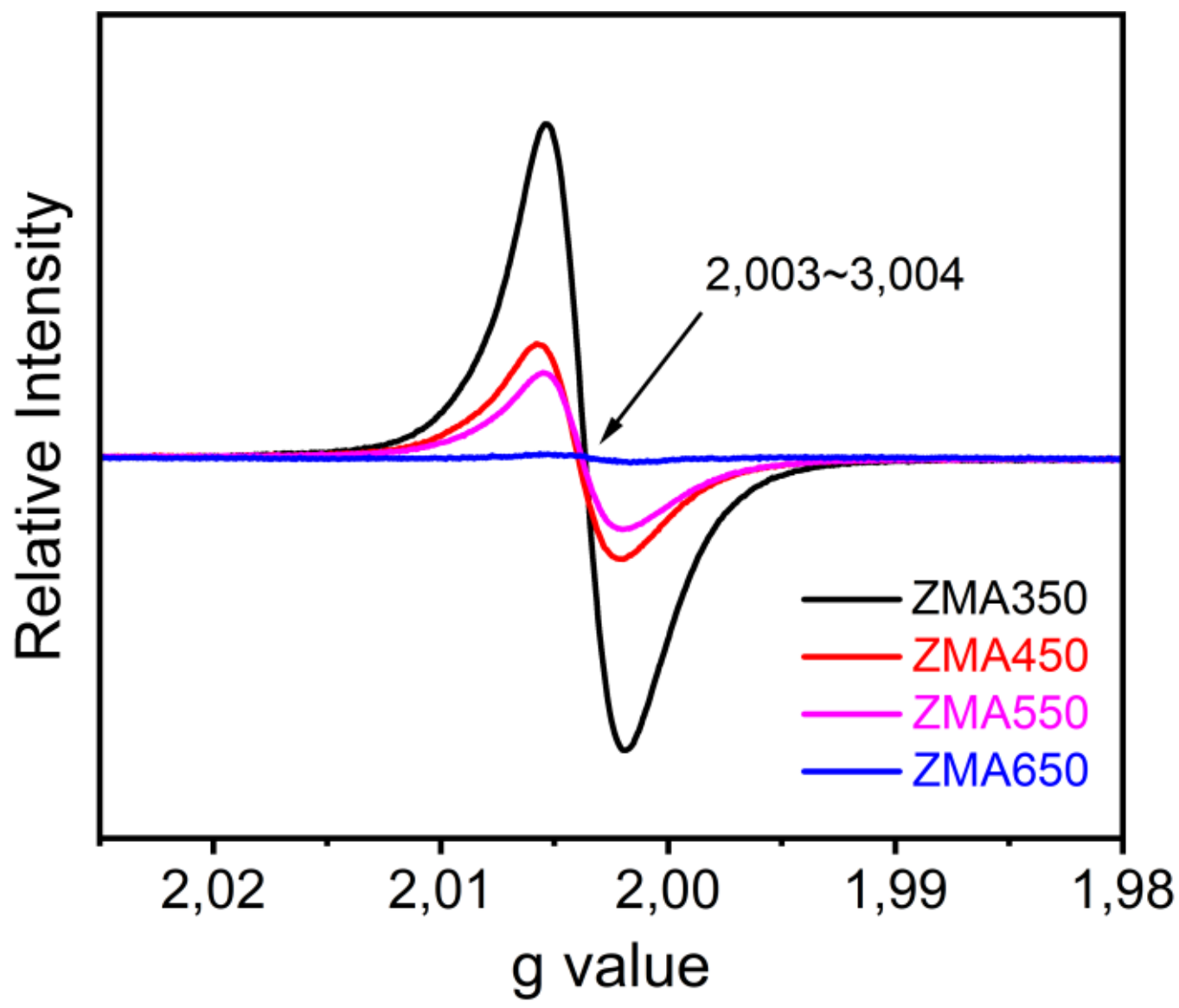

Supplementary Fig. 33 | EPR spectra of ZMA350, ZMA450, ZMA550, ZMA650 samples shown as relative intensity versus g value.

Comment 4. The authors propose the formation of SH- and Zn-SH complexes. Do you have spectroscopic evidence (e.g., FT-IR, in situ Raman) to support the chemical reaction pathways?

Reply: We agree that spectroscopic evidence is required to support the proposed formation of SH^- and Zn-S(H)-related surface complexes. In the revised manuscript, we clarify that this assignment is supported by in situ FT-IR measurements performed during H_2S exposure, together with complementary post-sensing characterization and DFT analysis.

In the in situ FT-IR spectra, no pronounced S-H stretching vibration is detected in the 2550-2600 cm^{-1} region. This observation is consistent with previous reports that H_2S preferentially undergoes dissociative adsorption on ZnO, forming strongly coordinated sulfur-containing surface species whose characteristic vibrational modes are located in the low-wavenumber region (<500 cm^{-1}), rendering the S-H stretching mode weak or undetectable. Therefore, the absence of an S-H stretching band does not preclude the formation of Zn-S(H)-type surface intermediates.

Importantly, H_2S exposure induces reversible and correlated changes in multiple surface-related vibrational features, including Zn-OH groups, hydrogen-bonded species, carbonate/bicarbonate bands, sulfur-containing surface intermediates, and Zn-O lattice vibrations. These reversible spectral responses indicate that H_2S interacts with ZnO through surface coordination and adsorption-desorption processes, rather than irreversible sulfidation. Consistently, post-sensing TEM, EDX, XPS, and PXRD analyses do not reveal the formation of ZnS secondary phases.

The DFT calculations, presented in the Supplementary Information, are used to rationalize the energetics and electronic structure perturbations associated with the grain boundary density-dependent variations in the Schottky barrier height during H_2S exposure, associated with the Zn-S(H)-related adsorption configurations inferred from the in situ FT-IR results, thereby linking the experimentally observed surface chemistry with the electronic structure modulation responsible for the sensing response.

These new results and the associated discussion have been added to the Supplementary Information (Supplementary Fig. 30) and explicitly referenced in the revised manuscript (Page 14, Line 429-439) and are also highlighted in red accordingly.

Revised parts of the manuscript are as follows:

In situ FTIR measurements provide experimental support for this surface reaction pathway, revealing reversible perturbations of surface hydroxyl groups, acid-base sites, and Zn-O-related lattice vibrations during H_2S adsorption, consistent with the formation of sulfur-containing surface intermediates rather than irreversible sulfidation (Supplementary Fig. 30). The DFT calculations show that the dissociative adsorption of H_2S results in significant electron transfer to the ZnO surface, leading to a reduction of the interfacial barrier (Details discussion can be found in (Supplementary Fig. 31). These findings are consistent with the in situ FT-IR measurements, where surface hydroxyl groups and Zn-O vibrations show dynamic shifts during H_2S exposure and removal. Together, the DFT results and in situ FT-IR data establish a coherent understanding of how H_2S adsorption modulates the electronic structure and surface chemistry of ZnO, confirming the reversible nature of the sensing process and its dependence on surface coordination dynamics rather than irreversible chemical transformation.

Revised parts of SI are as follows:

In situ Fourier-transform infrared (FTIR) spectroscopy was employed to probe the dynamic surface chemical evolution of ZnO during H_2S exposure, aiming to bridge static electronic structure characterization with real-time sensing behavior.

During H_2S exposure, no pronounced S–H stretching vibration was observed in the 2550–2600 cm^{-1} region. This observation is consistent with previous studies reporting that H_2S preferentially undergoes dissociative adsorption on ZnO surfaces, forming strongly coordinated sulfur-containing species (e.g., HS or S) bound to surface Zn sites. The characteristic vibrational modes of such Zn–S(H)-related intermediates are typically located in the low-wavenumber region (<500 cm^{-1}), rendering the S–H stretching mode weak or undetectable in the present measurements. Therefore, the absence of an S–H band does not preclude the formation of Zn–S(H)-type surface intermediates.

Several surface-related vibrational features exhibit pronounced and reversible intensity changes during H_2S on/off cycles (Supplementary Fig. 30 right). Specifically, the band at ~3680 cm^{-1}, assigned to terminal or weakly hydrogen-bonded Zn-OH groups, together with hydrogen-bond-associated bands near ~3000 cm^{-1}, shows reversible attenuation and recovery upon H_2S exposure and removal. In addition, paired bands at ~1400 and ~890 cm^{-1}, characteristic of surface carbonate/bicarbonate species, display correlated reversible variations [41-44]. These observations indicate that H_2S adsorption dynamically perturbs surface hydroxyl groups, acid-base sites, and adsorption equilibria on ZnO, rather than inducing permanent chemical

transformation. The reversibility of these spectral changes strongly suggests that the sensing process is governed by surface coordination and adsorption-desorption dynamics instead of irreversible sulfidation.

In the low-wavenumber region, a broad absorption band spanning 400-600 cm^{-1} with an inflection near ~520 cm^{-1} is assigned to Zn-O lattice vibrations. A reversible band at ~1060 cm^{-1} is observed upon H_2S exposure/removal, which is attributed to S-O stretching of surface Zn-SO_x species formed during the sensing process (Supplementary Fig. 30 left) [41-42, 45]. The modulation of these features during H_2S exposure reflects surface-sensitive perturbations of the Zn-O framework associated with adsorption-induced electronic and structural rearrangements. Importantly, post-sensing TEM, EDX, XPS, and PXRD analyses do not reveal the formation of ZnS secondary phases (Supplementary Fig. 12), confirming that the interaction between H_2S and ZnO is confined to reversible surface coordination rather than bulk-phase sulfidation.

Taken together, the in situ FTIR results provide direct evidence that H_2S adsorption induces reversible surface chemical and coordination changes on ZnO, dynamically modulating surface hydroxyls, acid–base equilibria, and Zn–O-related lattice environments. These surface processes are inherently coupled to electronic structure reconfiguration, thereby providing dynamic experimental support for the barrier-tunable sensing mechanism inferred from static electronic structure analysis.

Density functional theory (DFT) calculations were carried out to rationalize the energetics and electronic structure perturbations associated with H_2S-derived surface intermediates inferred from the in situ FTIR observations.

Representative adsorption configurations of H_2S and its dissociation products (HS and S) on the ZnO surface were considered. The calculated adsorption energies indicate that dissociative adsorption configurations involving Zn–S(H)-type coordination are energetically more favorable than molecularly adsorbed H_2S, consistent with the absence of a detectable S–H stretching vibration in the in situ FTIR spectra. Charge density difference and Bader charge analyses reveal noticeable electron transfer from the adsorbed sulfur-containing species to the ZnO surface, resulting in increased electron density localized near surface Zn sites. This charge redistribution is consistent with the experimentally observed upward shift of the Fermi level and reduction of the interfacial barrier upon H_2S exposure.

The DFT results provide electronic structure and energetic support for the surface intermediates and adsorption configurations inferred from in situ FTIR measurements. Together, these complementary experimental and theoretical results establish a coherent picture in which dissociative H_2S adsorption and

reversible Zn–S(H)-type surface coordination dynamically modulate the electronic structure of ZnO, underpinning the observed barrier-tunable sensing behavior.

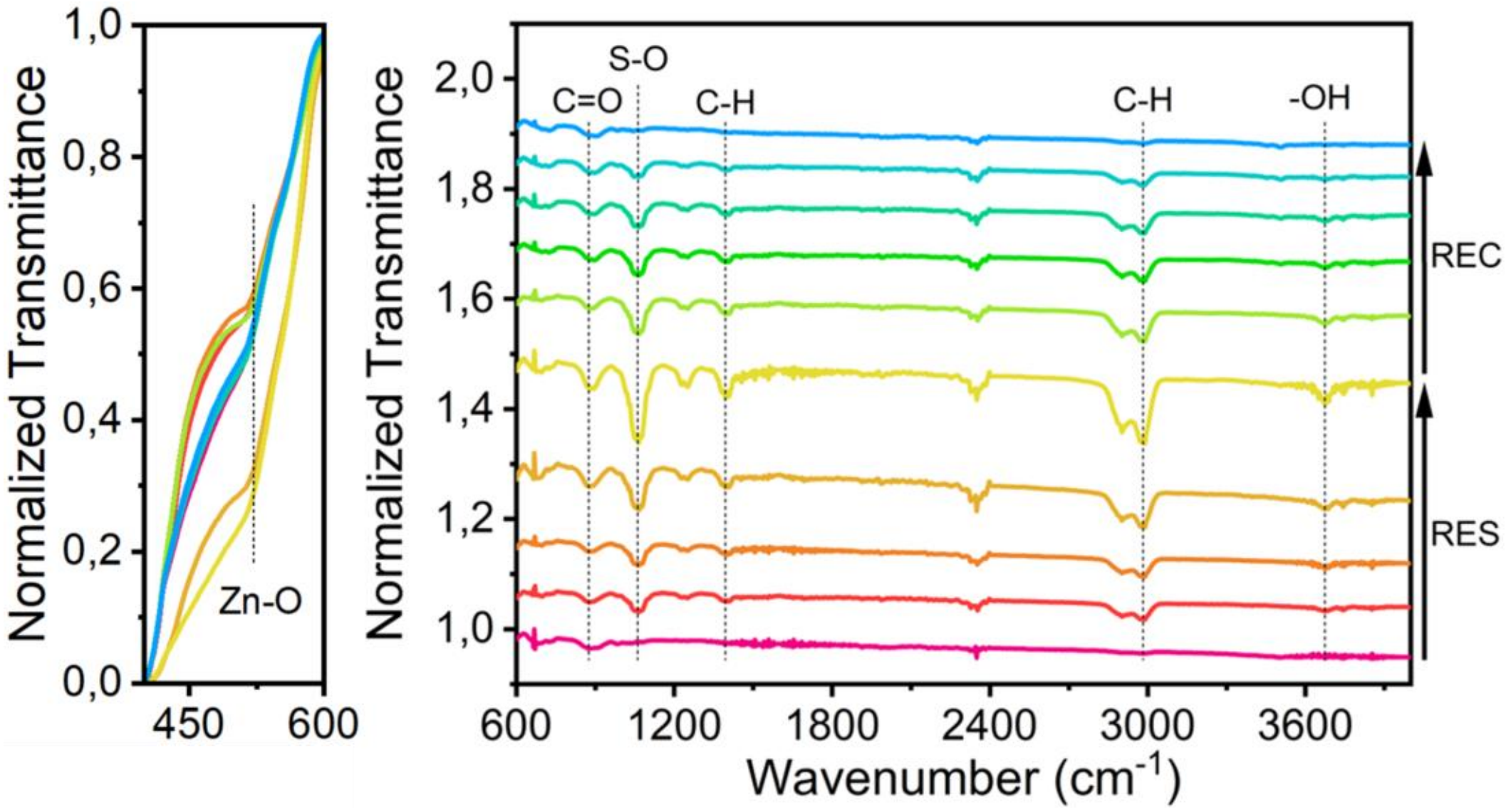

Supplementary Fig. 30 | In situ FTIR spectra of ZMA550 during H_2S adsorption and desorption.

Comment 5. All tests were performed at ~15% RH. How does the sensor behave across a realistic humidity range (30-90% RH) relevant for industrial and food-storage environments?

Reply: We sincerely thank the reviewer for highlighting the importance of evaluating sensor performance under realistic humidity conditions relevant to industrial and food-storage environments. In response, we conducted systematic humidity-dependent sensing measurements by exposing the ZMA550 sensor to 1 ppm H_2S under controlled relative humidity levels of 30-90% RH (with 15% RH included as a reference) (Supplementary Fig. 9). The humidity was continuously monitored using a calibrated Bosch BME280 sensor, and a schematic of the humidity-controlled sensing setup has been added (Supplementary Fig. 8). The response-recovery curves demonstrate that the sensor maintains a clear and reversible response to H_2S across the entire humidity range, indicating robust sensing behavior under varying ambient humidity conditions.

In addition, as part of addressing related comments, we performed complementary post-operation XPS analyses to examine potential humidity-induced surface changes (Supplementary Fig. 10). These results further support that the underlying sensing behavior remains intact under humid conditions; however, they serve as supporting evidence and do not alter the primary conclusion regarding humidity robustness.

Furthermore, although the device is designed for room-temperature operation, we also evaluated its response under representative temperatures within a practical window (-10 to 40 °C) while maintaining controlled gas concentration and humidity. The sensor remains responsive and reversible across this range (Supplementary Fig. 11), with temperature mainly affecting response/recovery kinetics rather than sensing functionality, consistent with thermally activated surface reaction and transport processes.

These new results and the associated discussion have been added to the Supplementary Information (Supplementary Fig. 8-11) and explicitly referenced in the revised manuscript (Page 8, Line 241-243) and are also highlighted in red accordingly, thereby strengthening the experimental basis of the proposed grain-boundary/Schottky-barrier synergistic mechanism.

Revised parts of the manuscript are as follows:

The sensor maintains a stable and reversible response to H_2S across a wide humidity range (15-90% RH) (Supplementary Fig. 8-10) and representative temperatures from -10 to 40 °C (Supplementary Fig. 11), demonstrating robust performance under realistic conditions.

Revised parts of SI are as follows:

The humidity was continuously monitored using a calibrated Bosch BME280 sensor, and a schematic of the humidity-controlled sensing setup has been added (Supplementary Fig. 8). It is worth noting that the response amplitude of the sensor to 1 ppm H_2S exhibits a gradual increase with increasing relative humidity, as consistently observed over repeated response–recovery cycles at each humidity level (Supplementary Fig. 9). This trend suggests that moderate humidity may facilitate the surface reaction kinetics or charge transfer processes involved in H_2S sensing.

To elucidate the underlying mechanism, XPS measurements were performed on the sensing layers after operation at different humidity levels, with particular focus on the O 1s region (Supplementary Fig. 10). The analysis reveals no significant change in the lattice oxygen (O_I) peak, while the intensity of the hydroxyl-related oxygen species (O_{III}) peak increases with humidity. This indicates that water vapor primarily influences the formation of hydroxyl-related oxygen species on the surface, rather than substantial modification of the lattice oxygen or irreversible water-induced surface reconstruction. Thus, humidity modulates the sensing response through surface hydroxylation, without affecting the core Schottky-barrier-modulated sensing mechanism.

In the pristine samples, the O 1s envelope was deconvoluted into lattice oxygen (O_I, ~530.0 eV), oxygen vacancies (O_{II}, ~531.1 eV), and loosely bound surface oxygen species (O_{III}, ~532.2 eV) (Supplementary Fig.

28b and c). Following sensing tests under varying humidity conditions, the O 1s spectrum was dominated by O_I (lattice oxygen, ~530.9 eV) and O_{III} (hydroxyl/adsorbed water-related oxygen species, ~532.9 eV). The emergence of O_{III} correlates with humidity-induced surface hydroxylation and the formation of H_2O-related species, whereas the relative intensity of the oxygen vacancy-related signal (O_{II}) is not explicitly observed due to the overlap with hydroxylated oxygen species. Importantly, these changes do not diminish the central role of oxygen vacancies in the sensing mechanism. Oxygen vacancies remain the primary reactive sites for H_2S adsorption, and the increased O_{III} component highlights the surface modification that occurs in the presence of humidity.

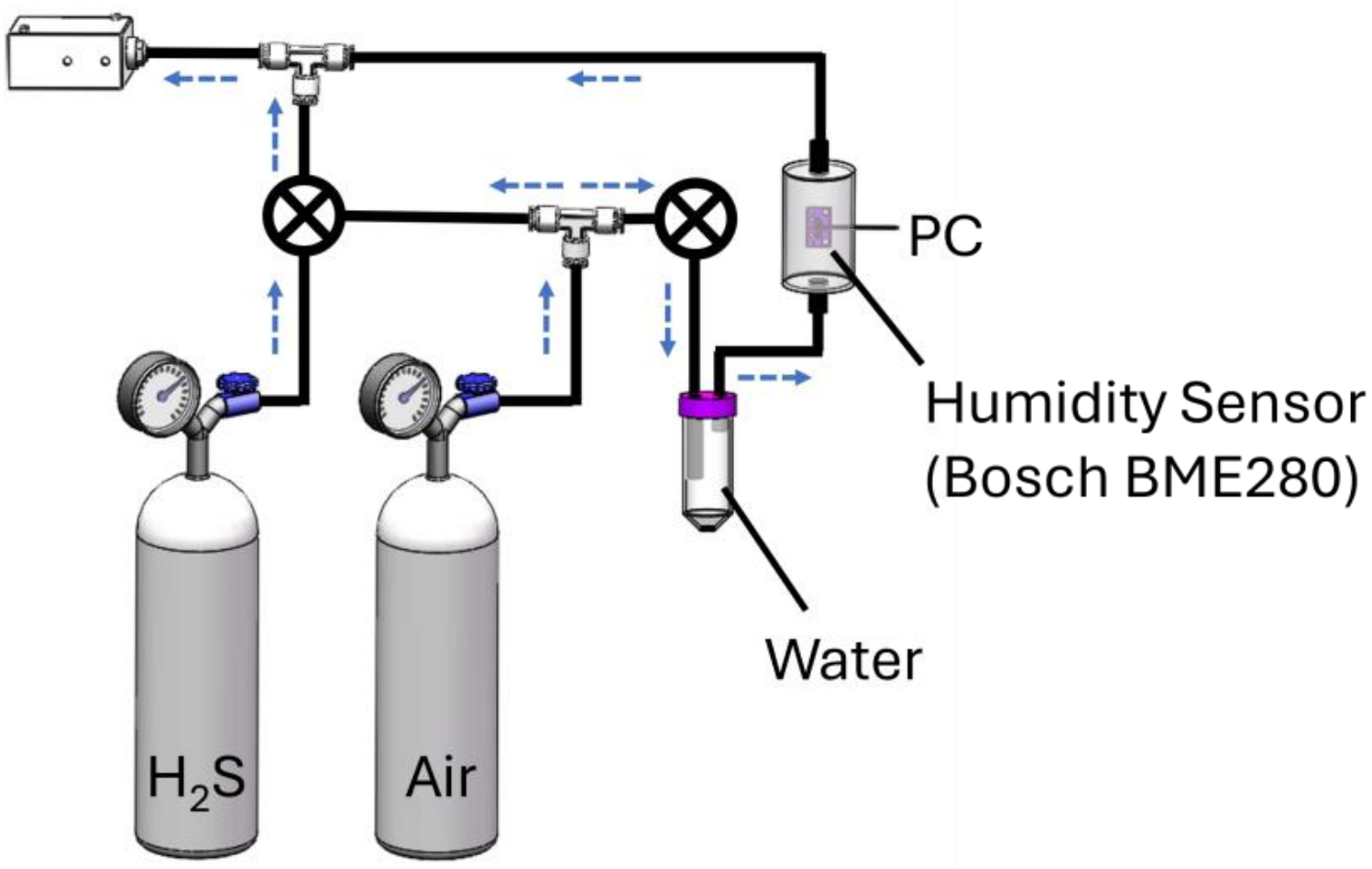

Supplementary Fig. 8 | Schematic diagram of humidity introduction in sensing tests.

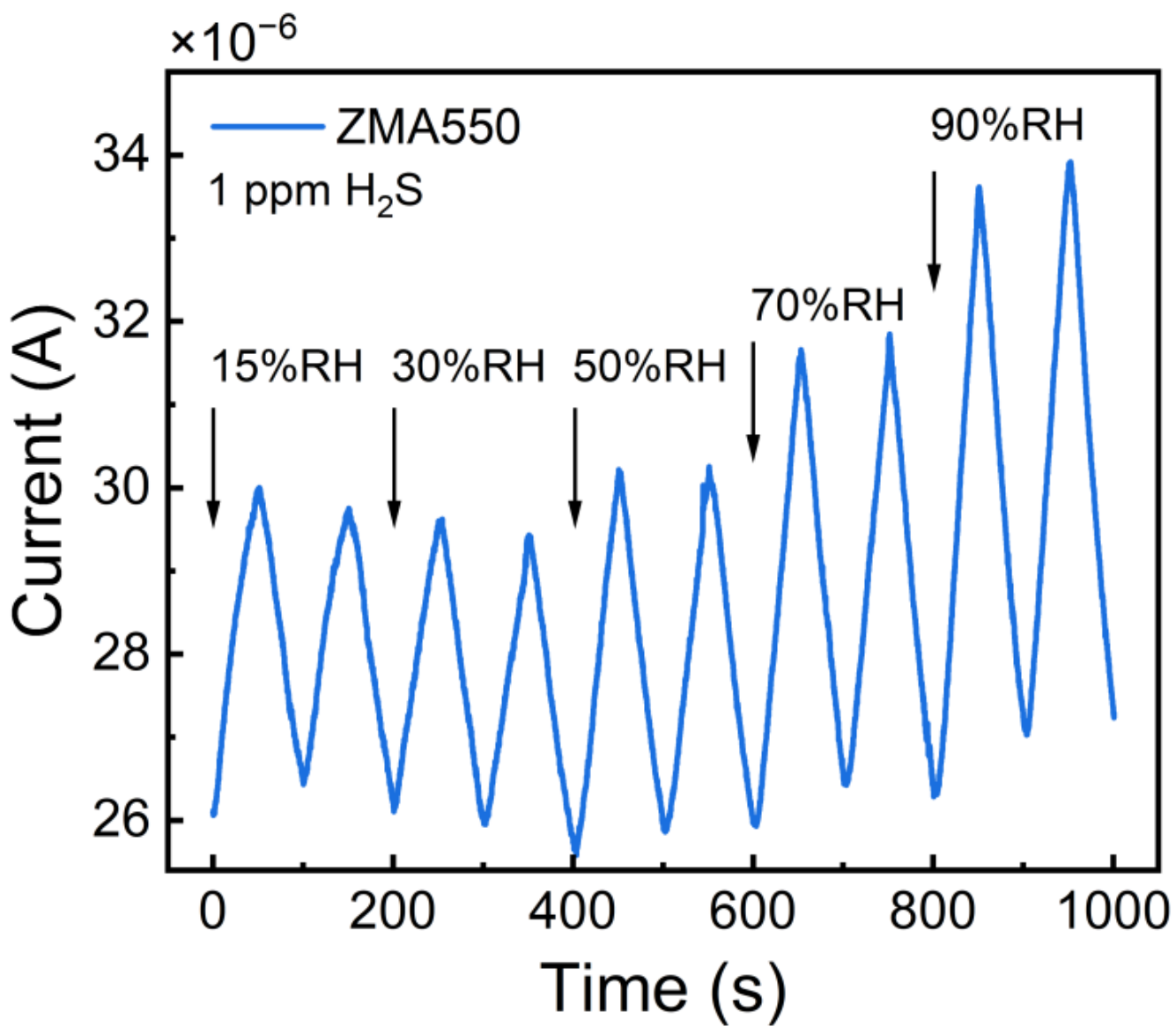

Supplementary Fig. 9 | Current response and recovery curves of ZMA550 sensor towards 1 ppm H_2S at different humidity levels from 15%RH-90%RH.

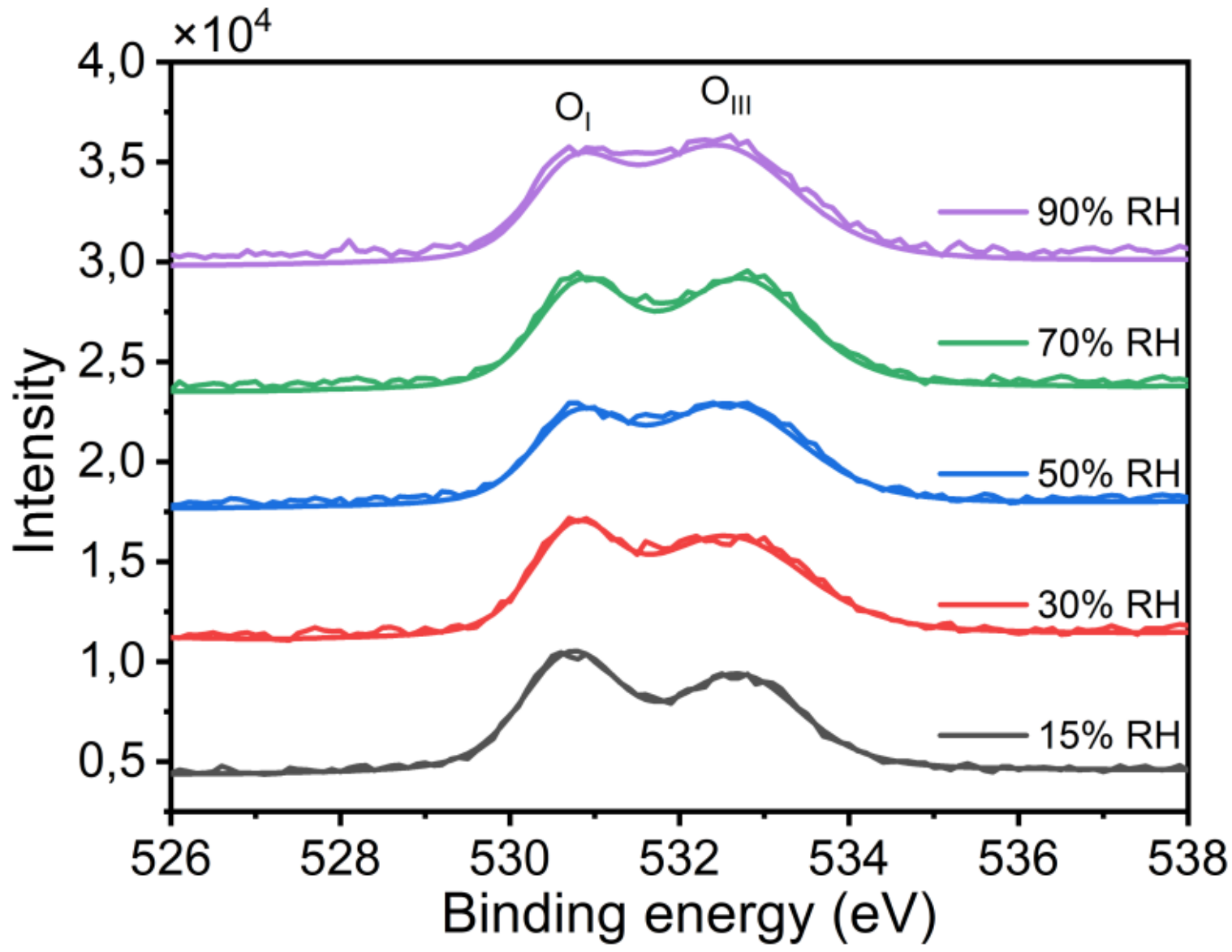

Supplementary Fig. 10 | O 1s XPS results of sensing tests under different humidity levels.

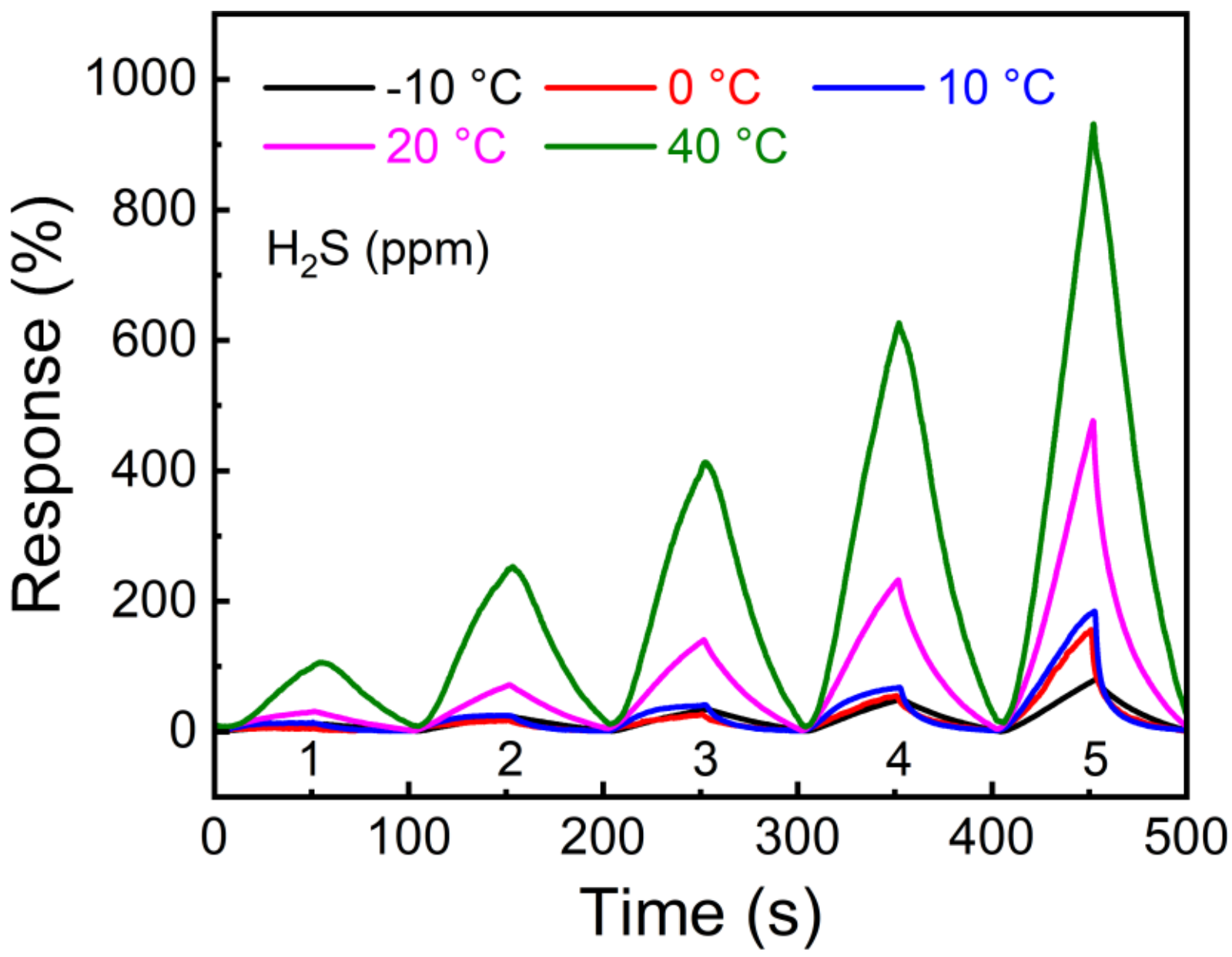

Supplementary Fig. 11 | Response and recovery curves of ZMA550 sensor exposed to varying high H_2S concentrations (1-5 ppm) under different operation temperature.

Comment 6. The 270-day stability data show little drift, which is unusual for ZnO in ambient conditions. Was the device continuously operated or periodically tested? How is contamination, electrode corrosion, or ZnS formation ruled out over long exposure cycles?

Reply: We thank the reviewer for raising this important point regarding the long-term stability protocol and the possibility of degradation in ambient conditions. The 270-day stability data were obtained from periodic measurements rather than continuous operation. Specifically, the device was stored under ambient conditions

and tested at approximately monthly intervals using the same sensing protocol (fixed bias, fixed H_2S concentration, and identical measurement settings). In the manuscript, we originally reported sensor stability over 270 days, based on monthly tests conducted throughout the testing period. However, it is important to note that since the initial submission of the manuscript, an additional 6 months of stability data have been collected, bringing the total stability testing period to 450 days. We have updated the manuscript accordingly to reflect this longer testing period.

To evaluate potential degradation pathways during long-term use, we performed multiple post-operation characterizations. First, we carried out HRTEM/EDX, PXRD, and XPS analyses on the sensing layer after the stability campaign (Supplementary Fig. 12). HRTEM revealed clear lattice fringes characteristic of ZnO with no ZnS crystalline domains, and PXRD showed no new diffraction peaks, confirming that ZnS did not form during the long-term exposure. XPS analysis revealed a weak sulfur signal in the S 2p region at 169 eV, consistent with SO_4^{2-} species but not ZnS, supporting that sulfur incorporation occurred in the form of sulfate phases rather than ZnS formation.

Second, optical micrographs of the device before and after long-term testing show that the Au electrodes remain intact without visible corrosion or delamination (inset of Supplementary Fig. 12b). Together, these results confirm that the observed low drift originates from the intrinsic robustness of the device architecture and that long-term exposure to H_2S does not induce electrode corrosion or bulk ZnS formation under our testing conditions.

These new results and the associated discussion have been added to the Supplementary Information (Fig. 2 and Supplementary Fig. 12) and explicitly referenced in the revised manuscript (Page 8, Line 243-249) and are also highlighted in red accordingly.

Revised parts of the manuscript are as follows:

Furthermore, the long-term stability was evaluated by periodic measurements over 450 days (monthly tests under identical sensing conditions) rather than continuous operation (Fig. 2g). Remarkably, monthly stability tests conducted thereafter show negligible signal degradation over an extended period, underscoring the sensor's reliability and suitability for long-term deployment in real-world environments. Post-test optical inspection and structural/chemical characterizations (HRTEM, PXRD, XPS, and EDX) confirm the integrity of the electrodes and exclude detectable secondary-phase formation (e.g., ZnS) after the stability campaign (Supplementary Fig. 12).

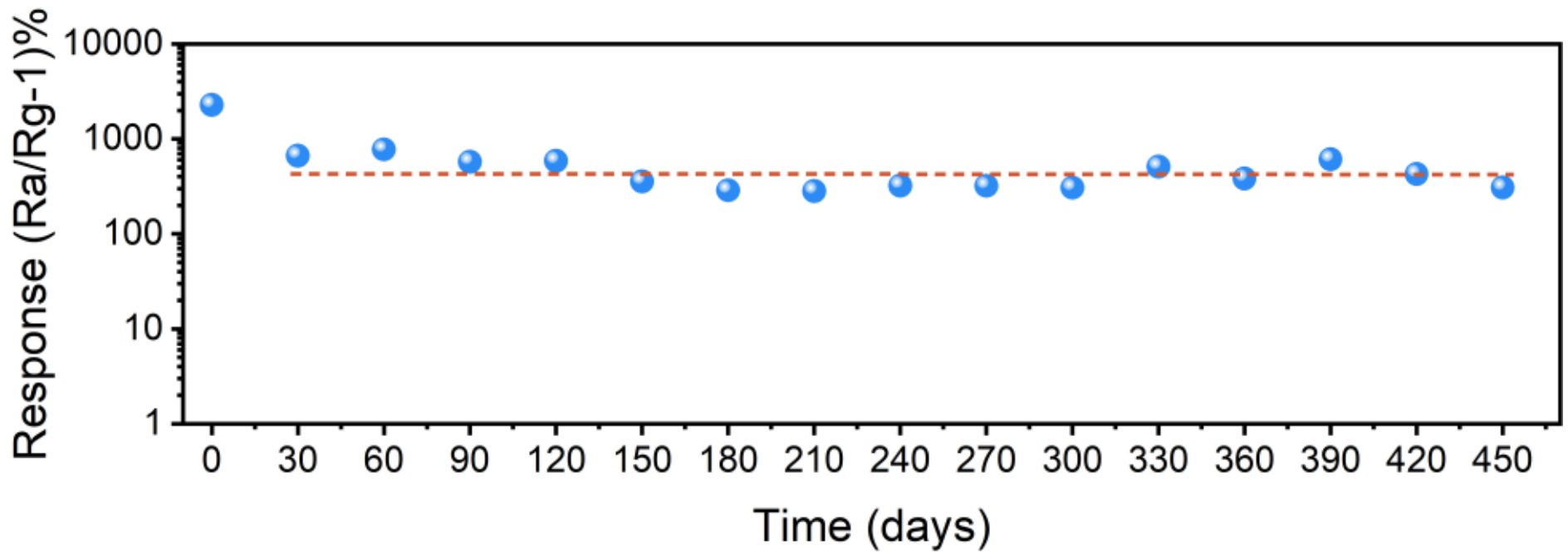

Fig. 2g | Long-term stability of ZMA550 over 450 days.

Revised parts of SI are as follows:

To examine whether long-term operation induces sulfidation or secondary-phase formation, the sensing layer after the stability campaign was characterized by HRTEM, PXRD, XPS, and EDX elemental mapping. HRTEM images revealed clear lattice fringes characteristic of ZnO and no ZnS crystalline domains, confirming that the ZnO structure remains intact at the nanoscale (Supplementary Fig. 12a). PXRD patterns did not show any additional peaks corresponding to ZnS, excluding the formation of the ZnS crystalline phase (Supplementary Fig. 12b). XPS analysis revealed a weak sulfur signal in the S 2p region at 169 eV, characteristic of SO_4^{2-} species, but no peaks indicative of ZnS formation were observed (Supplementary Fig. 12c). EDX mapping confirmed the absence of sulfur-rich ZnS domains or phase segregation (Supplementary Fig. 12d). These results collectively confirm that the long-term sensing process under our testing conditions is not accompanied by detectable bulk ZnS formation.

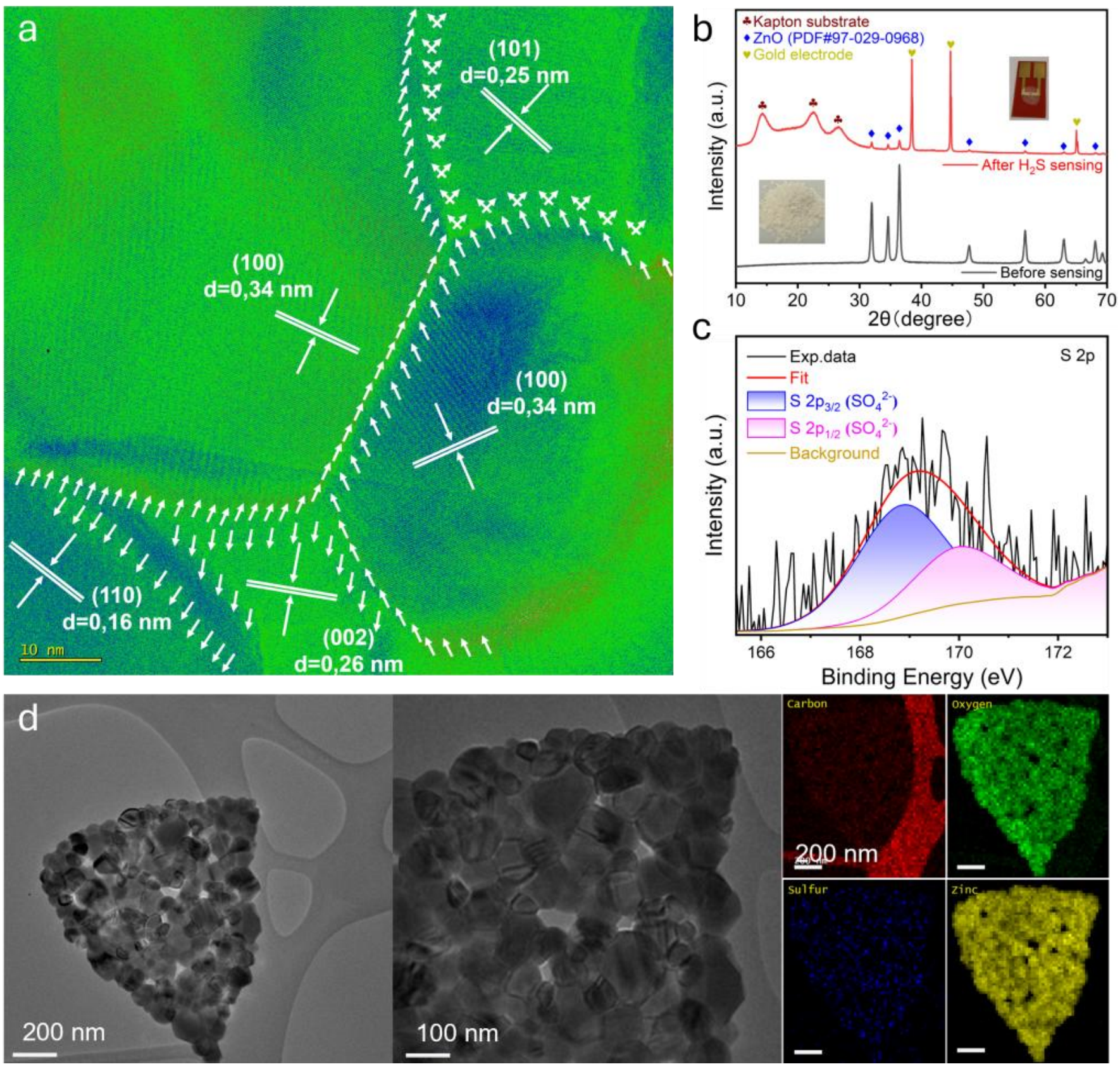

Supplementary Fig. 12 | HRTEM, PXRD, XPS, and EDX characterization of the sensing layer after long-term exposure to H_2S. **a,** HRTEM image showing lattice fringes characteristic of ZnO without ZnS formation. **b,** PXRD pattern confirming no ZnS phase formation. **c,** XPS S 2p spectrum revealing SO_4^{2-} species and the absence of ZnS peaks. **d,** EDX elemental mapping showing homogeneous elemental distribution without sulfur-rich ZnS domains.

Comment 7. Selectivity is shown for 10 gases, but cross-interference under mixtures is unclear. How does the sensor respond when H_2S coexists with oxidizing gases (e.g., NO_2, SO_2) or reductive gases (e.g., NH_3) simultaneously?

Reply: We sincerely thank the reviewer for raising the important point regarding the interference of both oxidizing (e.g., NO_2, SO_2) and reducing (e.g., NH_3) gases in real-world environments. To address this, we conducted dynamic interference stability tests by exposing the sensor to 3 ppm H_2S in the presence of both oxidizing and reducing gases simultaneously. Specifically, we performed a series of tests with six different combinations of interfering gases:

NO_2, SO_2, NH_3 (individual gases)

NO_2 + SO_2, NO_2 + NH_3, SO_2 + NH_3 (gas mixtures)

$NO_2 + SO_2 + NH_3$ (all three gases together)

The response and recovery behavior were monitored over 18 consecutive cycles, with each cycle featuring a different combination of these interfering gases. As shown in Supplementary Fig. 13, the sensor exhibited stable, reproducible, and reversible responses to H_2S across all cycles, even when multiple interfering gases were present simultaneously. No significant degradation in response, baseline drift, or response attenuation was observed, demonstrating that the sensor maintains its sensing performance despite the simultaneous presence of both oxidizing and reducing gases.

In addition to these tests, we also performed tests with volatile organic compounds (VOCs), which are another category of common interferents. These results, along with a detailed description of the experimental setup and gas introduction protocol, have been included in Supplementary Fig. 14-15. These findings provide further evidence of the sensor's robustness against a wide range of potential interferences in complex, real-world gas environments.

These new results and the associated discussion have been added to the Supplementary Information (Supplementary Fig. 13-15) and explicitly referenced in the revised manuscript (Page 8, Line 250-258) and are also highlighted in red accordingly.

Revised parts of the manuscript are as follows:

To assess cross-interference under mixed-gas conditions relevant to practical environments, we performed dynamic co-exposure tests in which H_2S was introduced together with representative oxidizing gases (NO_2 and SO_2) and a reducing gas (NH_3), both individually and in mixtures. The sensor maintains a stable and reproducible H_2S response even when oxidizing and reducing interferents coexist simultaneously, demonstrating strong anti-interference capability in complex gas matrices (Supplementary Fig. 13-15). The robustness is particularly relevant to the boiled-egg spoilage headspace tests, where multiple VOCs (e.g., amines, organic acids, and small sulfur-containing species) may be present. The mixed-gas evaluation therefore supports that the response observed in the spoilage demonstration is primarily governed by H_2S-associated surface/interfacial modulation rather than nonspecific responses to coexisting volatiles.

Revised parts of SI are as follows:

To evaluate the robustness of the H_2S sensing signal under realistic multi gas environments, we conducted a dynamic interference stability test by repeatedly exposing the sensor to 3 ppm H_2S over 18 consecutive response–recovery cycles. During each cycle, one set of interfering gases was introduced simultaneously with H_2S, while all other experimental parameters (e.g., concentration control, total flow rate,

temperature, and bias/measurement settings) were kept constant. In particular, to directly address the coexistence of oxidizing and reducing species, we tested representative oxidizing gases (NO_2, SO_2) and a reducing gas (NH_3) both individually and in mixtures, including NO_2 + SO_2, NO_2 + NH_3, SO_2 + NH_3, and NO_2 + SO_2 + NH_3. The sensor exhibits highly reproducible H_2S response amplitudes with negligible baseline drift across all cycles, indicating that the sensing output is dominated by H_2S-triggered interfacial modulation rather than transient cross-interference from coexisting gases (Supplementary Fig. 13). A schematic of the gas-introduction protocol and the overall setup is provided in Supplementary Fig. 14, and a photograph of the actual experimental setup is shown in Supplementary Fig. 15 to ensure transparency and reproducibility.

In addition to the inorganic interferents above, we further evaluated potential cross-sensitivity to representative VOC interferents that are commonly reported in food spoilage headspaces, particularly during egg fermentation. These VOCs include amines (triethylamine, propylamine, butylamine), organic acids (acetic acid and butyric acid), sulfur-containing species (dimethyl sulfide), and small oxygenated molecules (formaldehyde, ethanol, and acetone). The sensor maintains stable and reversible H_2S responses under these VOC interference conditions as well (Supplementary Fig. 13), further supporting its robustness in complex gas matrices.

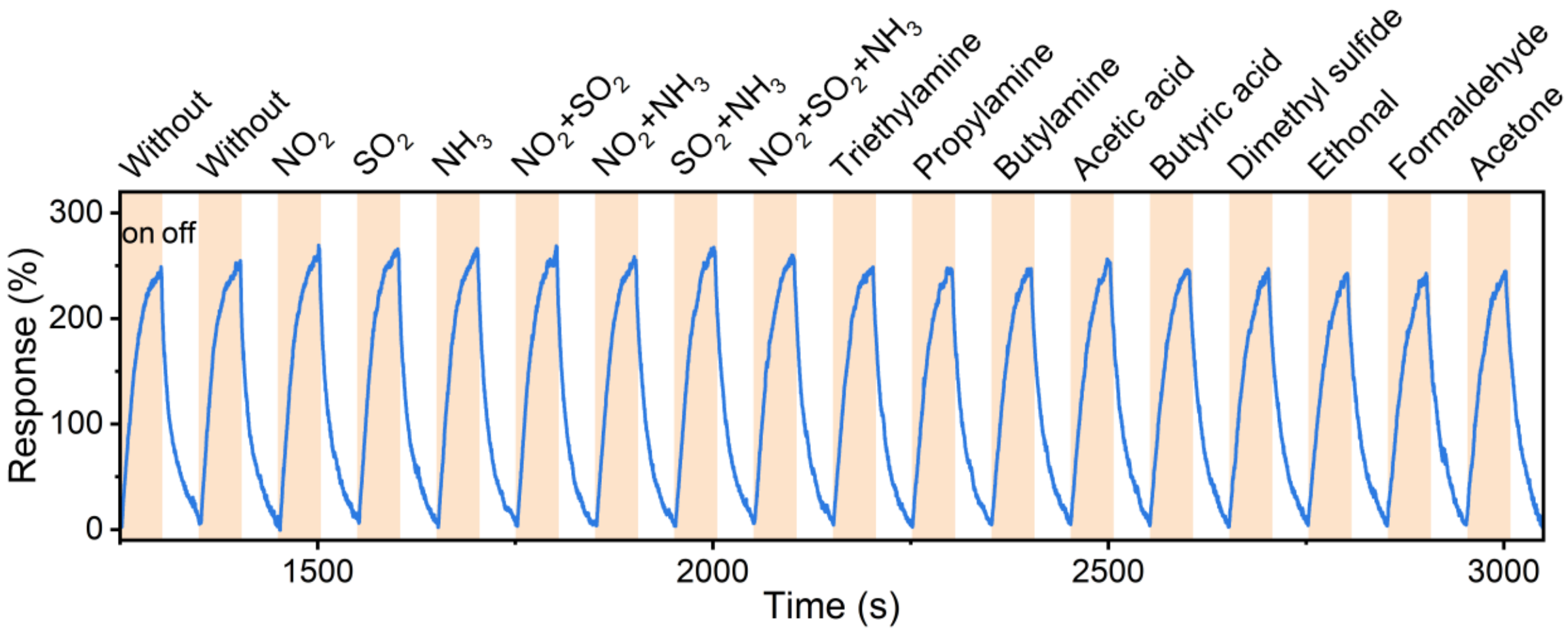

Supplementary Fig. 13 | Dynamic interference stability of the H_2S sensor.

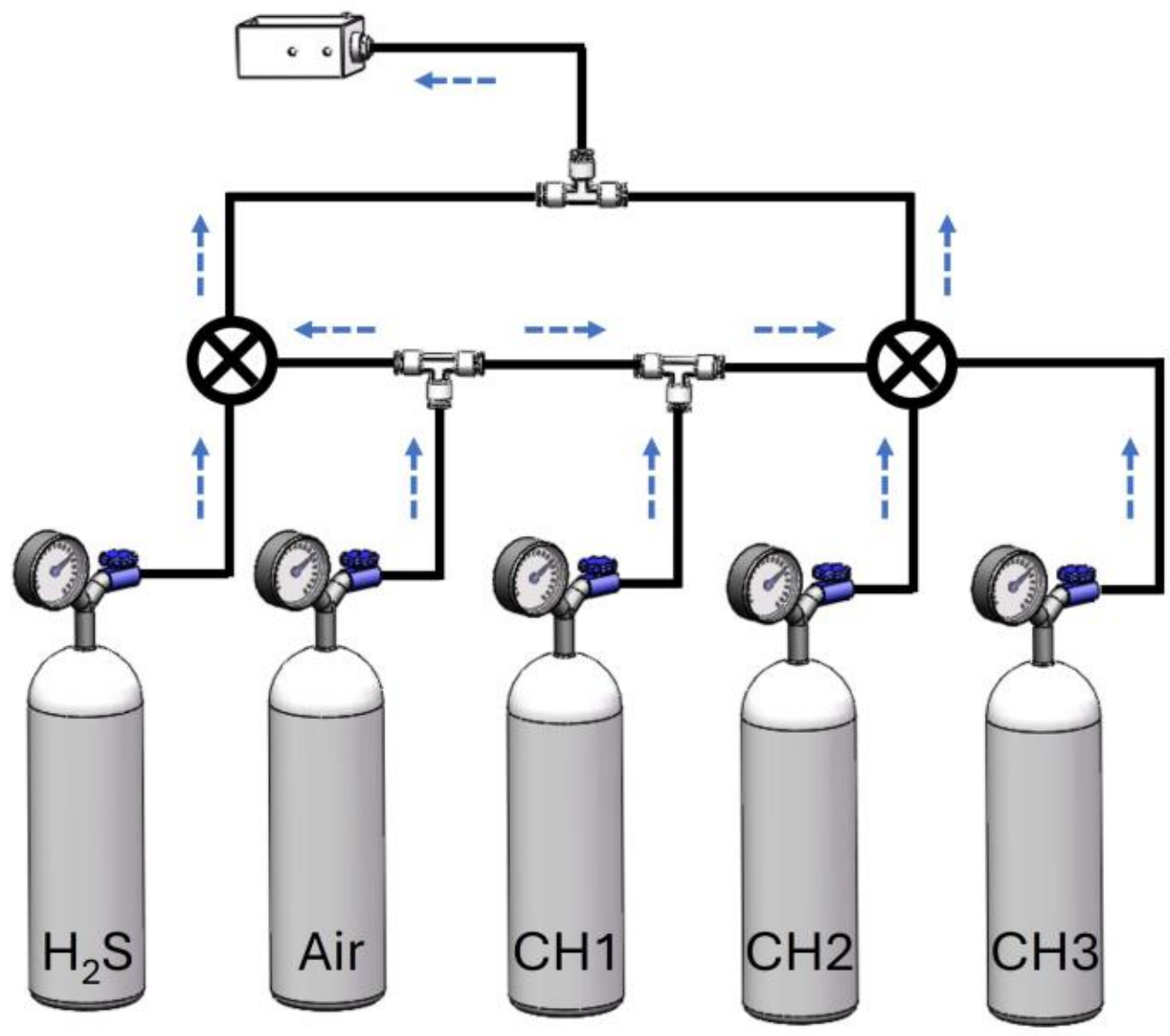

Supplementary Fig. 14 | Experimental setup for dynamic interference stability measurements.

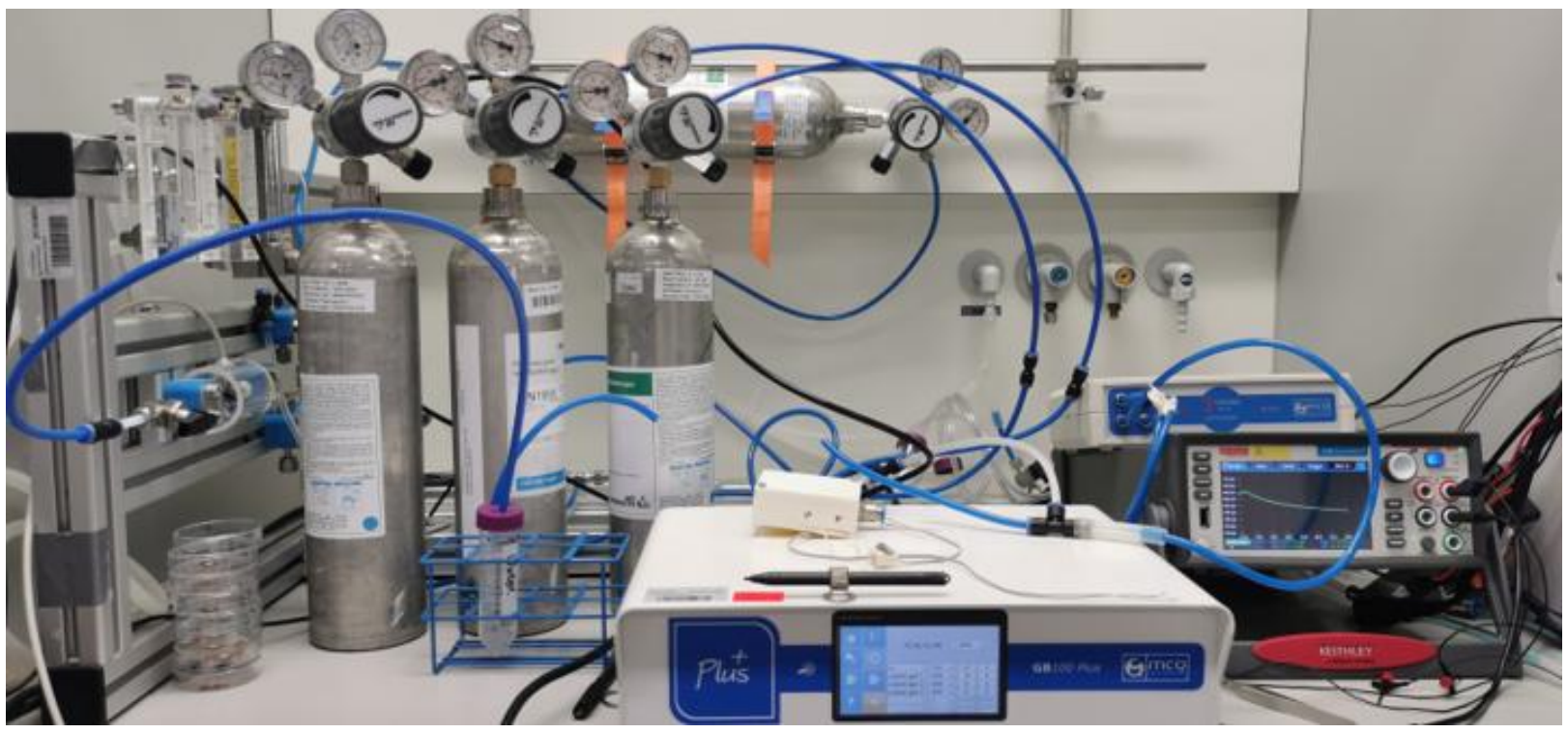

Supplementary Fig. 15 | Photograph of the actual experimental setup.

Comment 8. Baseline anchoring using second-derivative zero points may artificially influence ultra-low concentration detection. Can you demonstrate that the LOD (0.328 ppb) is not dependent on the correction protocol?

Reply: We thank the reviewer for raising this concern about the potential influence of baseline correction methods on the LOD. We would like to clarify that our LOD calculation does not rely on second-derivative zero-point baseline anchoring or any similar signal manipulation techniques. Instead, the LOD is determined directly from the raw current response amplitudes (ΔI) measured at various H_2S concentrations, using a log-log calibration. The sensitivity (slope) is obtained from linear fitting of the log-log response-concentration plot.

Furthermore, the baseline noise (RMS) is calculated directly from the air measurements, with no background correction applied beyond basic noise averaging. The LOD is then computed using the standard

3σ criterion, ensuring that the result reflects the intrinsic sensor response and is not artificially amplified by any complex baseline processing.

These new results and the associated discussion have been added to the Supplementary Information (Supplementary Fig. 6) and explicitly referenced in the revised manuscript (Page 7, Line 235-237) and are also highlighted in red accordingly.

Revised parts of the manuscript are as follows:

Comparative analysis of sensitivity and LOD under N_2 and air atmospheres revealed optimal performance in air, with an unprecedented LOD of 0.328 ppb achieved for H_2S detection (Fig. 2e, details shown in Supplementary Fig. 6).

Revised parts of SI are as follows:

The sensor response at each H_2S concentration was obtained from the time-resolved current curves by extracting the maximum response amplitude during H_2S exposure (Supplementary Fig. 6a). The concentration–response relationship was constructed as a log–log plot and fitted using a linear model:

$$\lg(\text{Current}) = k \cdot \lg(C) + b$$

where C is the H_2S concentration and k is the slope (sensitivity in the log–log domain) used for LOD estimation. The baseline noise (RMS) was quantified from the current–time signal recorded under air conditions. To remove slow instrumental drift without affecting high-frequency noise, the baseline was fitted using a low-order (5th-order) polynomial, and the RMS was calculated from the residuals ($Y_i - Y_{fit}$) (Supplementary Fig. 6b). This procedure serves only to isolate stochastic noise and does not involve derivative-based baseline anchoring [40]. The LOD is then calculated using the 3σ method:

$$\text{LOD} = 3.3\ \delta / \text{S}$$

where δ represents the standard deviation of the baseline noise extracted from the sensor signal under air background gas at room temperature, and S denotes the sensitivity, defined as the slope of the linear fitting of the sensor response versus H_2S concentration in the low-concentration regime. This approach ensures that the LOD is intrinsic to the sensor's response to H_2S, with no dependence on arbitrary baseline correction procedures.

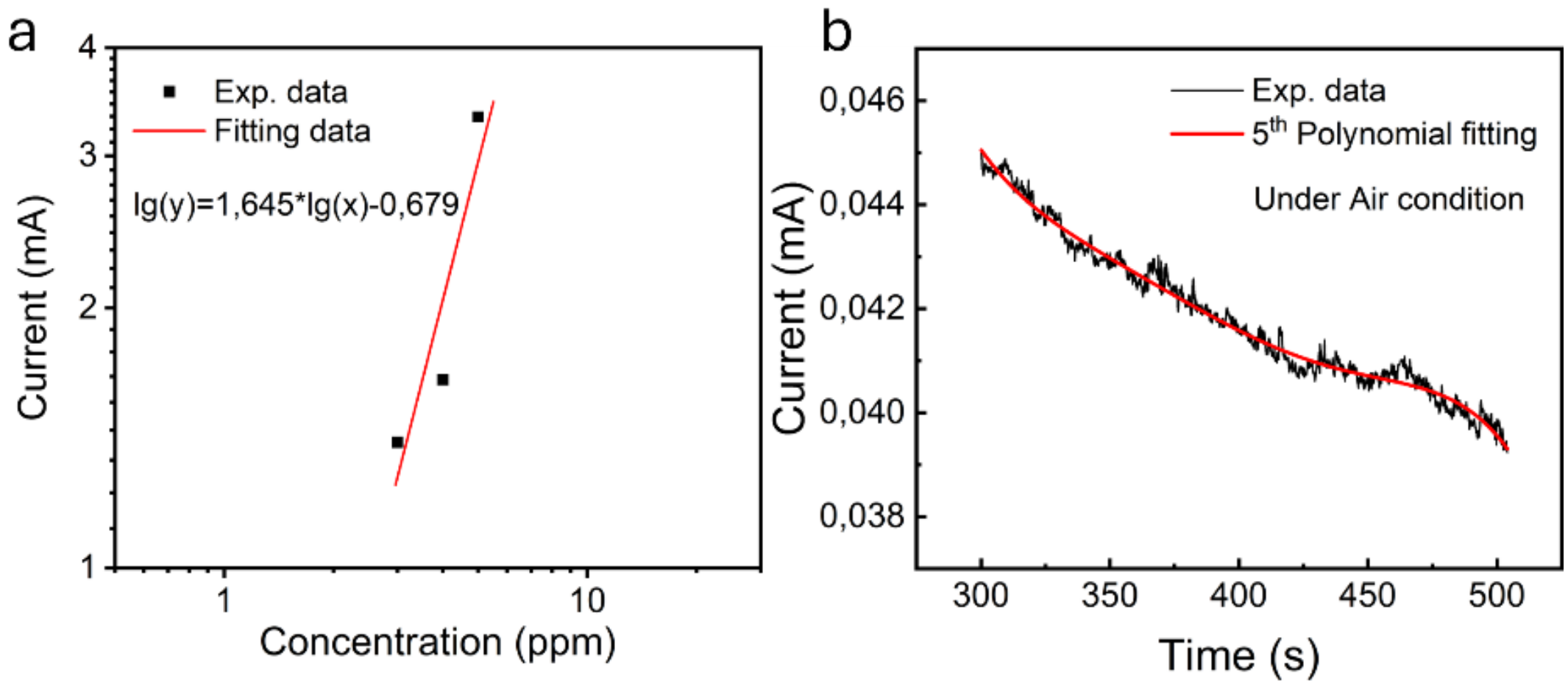

Supplementary Fig. 6 | a, Response concentration log-log plots to different concentrations of H_2S under air condition. **b,** RMS noise levels of response as a function of time under air conditions.

Comment 9. The authors state a 4-5× faster response and 10^5-fold lower power consumption compared to commercial sensors. Which specific commercial models were used as benchmarks, and under what conditions?

Reply: We sincerely thank the reviewer for the insightful comment and for highlighting the need for clarity in the comparison with commercial sensors. We apologize for any ambiguity regarding the comparison, and we have revised the manuscript and Supplementary Information to address these concerns.

First, we clarify that the 4-5× faster response and 10^5-fold lower power consumption are based on a comparison with representative commercial MOS-based H_2S sensors, specifically SGX-4H_2S-100 (RS Components), H_2S Sensor V1.0 (DFRobot, Fermion), MQ136 (HUABAN), and S-H_2S-01 (Seeed Technology) that are widely used in the field. These sensors were chosen as benchmarks due to their prevalence in H_2S detection applications and their corresponding publicly available datasheet values.

Second, we emphasize that the comparison is architecture-level and is not based on identical experimental conditions, as commercial datasheets do not always provide standardized or complete testing conditions (e.g., humidity, interfering gases, etc.). The power consumption values for these commercial sensors (typically 25-1300 mW) were extracted from publicly available datasheets and calculated from the reported operating voltage and current (P = V × I). These datasheet values reflect the overall power consumption, which includes the energy required for the micro-heater used in elevated-temperature operation, and the comparisons are framed accordingly to highlight the fundamental energy-budget difference between heater-free, room-temperature sensing and conventional heater-driven MOS sensing.

To improve verifiability, we have added Supplementary Tab. 8 to list the specific commercial sensor models, their operating voltages, currents, and calculated power consumption, as well as their typical detection limits and datasheet sources. In the revised manuscript, we explicitly state that the comparison is based on publicly available datasheet values, and not a one-to-one performance evaluation under identical testing conditions. This distinction has been added to the manuscript and the Supplementary Information.

These new results and the associated discussion have been added to the Supplementary Information (Supplementary Tab. 7 and Tab. 8) and explicitly referenced in the revised manuscript (Page 17, Line 541-550) and are also highlighted in red accordingly.

Revised parts of the manuscript are as follows:

Benefiting from heater-free, room-temperature operation, the sensing element in this work operates in the µW power regime with an average power consumption of 0.7 µW in active mode and 0.3 µW in idle mode (details in Supplementary Tab. 7), whereas representative commercial MOS H_2S sensors typically operate in the mW regime (25-1300 mW), as derived from publicly available datasheet specifications. This architecture-level comparison highlights an energy-budget reduction of approximately five orders of magnitude. In addition, the response dynamics are approximately 4-5× faster than those reported for commercial MOS sensors, based on datasheet values and publicly available literature. These comparisons are intended to highlight fundamental differences in sensing architecture, rather than a one-to-one performance comparison under identical testing conditions (details in Supplementary Tab. 8).

Revised parts of SI are as follows:

Power consumption for the commercial sensors listed in Supplementary Tab. 8 was extracted from publicly available manufacturer datasheets. These datasheets report the overall power consumption of the device, which includes both the sensing element and any additional components such as heating elements or signal conditioning circuits. Commercial datasheets generally do not provide a full breakdown of power consumption across individual modules (sensing element vs. heater vs. signal processing), so these values should be interpreted as a product-level benchmark rather than a direct one-to-one comparison under identical experimental conditions.

Supplementary Tab. 8 | Power Consumption and Testing Parameters for Sensor and Commercial Sensors.

Sensor Type	Model	T_{90} (s)	LOD (ppm)	Operating Voltage (V)	Current (A)	Power (W)	Power Source Breakdown	Source	Notes
**This work**	**H_2S**	**7**	**0.0003** **28**	**3.725**	**0.1668 µA**	**0.6312 µW**	**Sensor**	**SI**	**Sensing element: µW-level, wireless + ADC = mW-level**
				**2.5**	**0.00819**	**0.0205**	**Wireless**	**SI**	
Commercial 1	SGX-4H_2S-100	30	/	1.3	1	1.3	Sensor	datasheet	W-level, heat requiremtn, heat power, etc
Commercial 2	H_2S Sensor V1.0	20	0.5	3.3-5	0.02	0.066	Sensor	datasheet	mW-level, heat requiremtn, heat power, etc
Commercial 3	MQ136	30	1	5	0.18	0.9	Sensor	datasheet	mW-level, heat requiremtn, heat power, etc
Commercial 4	S-H_2S-01	30	0.1	3.3-5.5	0.05	0.025	Sensor & Wire	datasheet	mW-level, heat requiremtn, heat power, wire transmission etc

Commercial Sensor Datasheet Sources:

Commercial Sensor 1: RS Components GmbH, Industrial Hydrogen Sulphide (H_2S) Sensor, Datasheet, available at [https://docs.rs-online.com/50d2/A700000009288588.pdf], accessed [21.01.2026].

Commercial Sensor 2: DFRobot, Fermion: H_2S Gas Sensor, Datasheet, available at [https://www.dfrobot.com/product-2702.html?srsltid=AfmBOoo57XTXfPiknsJnx7tR_0KkWUVBd00iB-ZgpHbOBsSuZ9ynozD1], accessed [21.01.2026].

Commercial Sensor 3: HUABAN, 1 x MQ-136 MQ136 Hydrogen Sulfide H_2S, Datasheet, available at [https://www.amazon.de/HUABAN-MQ-136-MQ136-Schwefelwasserstoff-H2S-Detektionsgassensormodul-Alarmsignallampe/dp/B089SQDQFJ?source=ps-sl-shoppingads-lpcontext&ref_=fplfs&smid=A169MKB0ILTINE&th=1], accessed [21.01.2026].

Commercial Sensor 4: Seeed Technology, S-H_2S-01, Datasheet, available at [https://files.seeedstudio.com/products/101990863/RES/RS485%20H_2S%20Sensor%20-%20Datasheet.pdf], accessed [21.01.2026].

Comment 10. The upper concentration limit is not clearly defined. Does the sensor saturate beyond 100 ppm, and what is the linearity range?

Reply: We thank the reviewer for the insightful comment regarding the upper concentration limit and potential saturation of the sensor. In response to this, we extended the H_2S concentration range to 200-1000 ppm and tested the sensor's response and recovery characteristics under these conditions.

First, we observe that while the response at higher concentrations (200-1000 ppm) remains high, the magnitude of the response is generally lower than that observed at lower concentrations (10-100 ppm). This behavior is consistent with the concentration-response relationship, where the sensor exhibits linear response from 200 to 900 ppm, but from 800 to 1000 ppm, the response follows a different linear relationship.

By fitting the highest response values with a linear regression, we find that the sensor maintains a linear response from 200 to 900 ppm, but the response at 1000 ppm is still keep increasing with exposure of H_2S, suggesting that the sensor does not saturate within the tested range. However, the response curve from 800 to 1000 ppm shows a shift in slope, indicating a new linear regime beyond 900 ppm.

These findings suggest that the sensor continues to function effectively at concentrations as high as 1000 ppm, but the sensitivity decreases progressively as the concentration increases, likely due to the occupation of reactive adsorption sites on the sensor surface. However, no saturation is observed at 1000 ppm, and the sensor continues to exhibit high response without a plateau.

These new results and the associated discussion have been added to the Supplementary Information (Supplementary Fig. 4) and explicitly referenced in the revised manuscript (Page 7, Line 222-227) and are also highlighted in red accordingly.

Revised parts of manuscript are as follows:

Furtherly, in the extended range of 200-1000 ppm, the response continues to increase with concentration and no plateau is observed up to 1000 ppm, indicating that complete saturation is not reached within the tested window. Detailed results and analysis of the response behavior at these concentrations are provided in the Supplementary Information, including the concentration-response relationship and the observed changes in slope at higher concentrations (Supplementary Fig. 4).

Revised parts of SI are as follows:

In the extended concentration range of 200-1000 ppm, the sensor maintains a linear response up to 900 ppm, with an increased slope observed beyond 900 ppm. At 1000 ppm, the response remains high but exhibits a significantly higher value than expected from the lower concentration range, suggesting that the

sensor continues to exhibit a high response without reaching saturation, but with a different linear regime beyond 900 ppm. The change in slope at concentrations above 900 ppm indicates that the sensor's sensitivity is reduced in this range due to the occupation of reactive adsorption sites (Supplementary Fig. 4).

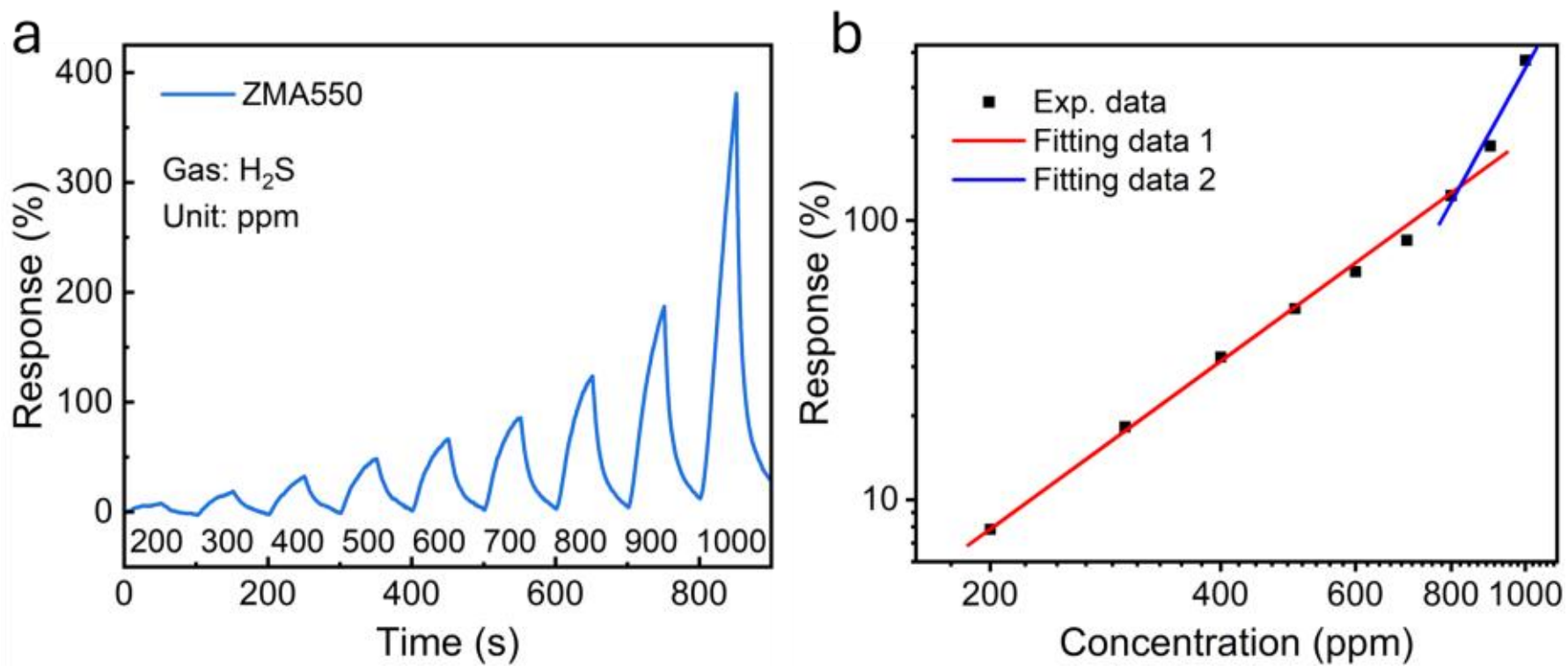

Supplementary Fig. 4 | a, Dynamic response and recovery curves of the ZMA550 sensor exposed to varying high H_2S concentrations (200-1000 ppm) air condition. **b,** Response concentration log-log plots to different concentrations of H_2S.

Comment 11. Power values are calculated from instantaneous resistance and voltage. What is the total power, including the wireless module's duty cycle, ADC sampling, and Bluetooth transmission? Is the 0.7 µW value just the sensing layer, or the entire system?

Reply: We thank the reviewer for raising this important point. We clarify that the reported 0.7 µW (active) and 0.3 µW (idle) values correspond exclusively to the sensing element, calculated from the applied bias voltage and the instantaneous sensor resistance/current during operation (Supplementary Tab. 7).

To address the system-level power consumption requested by the reviewer, we directly measured the electrical power of the complete sensing device, including the sensor interface, ADC sampling, microcontroller operation, wireless duty cycle, and Bluetooth transmission, under the same operating and data-transmission settings used in this work. With a regulated 2.5 V supply, the communication/electronics module draws an average current of 8.19 mA, corresponding to a total power consumption of approximately 20.48 mW.

These results clearly distinguish the intrinsic µW-level power consumption of the sensing layer from the mW-level power required for signal acquisition and wireless data transmission. The manuscript has been revised to explicitly separate sensor-only power from device-level power, and the detailed are provided in the Supplementary Information (Supplementary Tab. 7).

These new results and the associated discussion have been added to the Supplementary Information (Supplementary Tab. 7) and explicitly referenced in the revised manuscript (Page 17, Line 541-550) and are also highlighted in red accordingly.

Revised parts of the manuscript are as follows:

Benefiting from heater-free, room-temperature operation, the sensing element in this work operates in the μW power regime with an average power consumption of 0.7 μW in active mode and 0.3 μW in idle mode (details in Supplementary Tab. 7), whereas representative commercial MOS H_2S sensors typically operate in the mW regime (25-1300 mW), as derived from publicly available datasheet specifications. This architecture-level comparison highlights an energy-budget reduction of approximately five orders of magnitude. In addition, the response dynamics are approximately 4-5× faster than those reported for commercial MOS sensors, based on datasheet values and publicly available literature. These comparisons are intended to highlight fundamental differences in sensing architecture, rather than a one-to-one performance comparison under identical testing conditions (details in Supplementary Tab. 8).

Revised parts of SI are as follows:

The sensing-element power reported in the manuscript (0.7 μW in active mode and 0.3 μW in idle mode) was calculated from the applied bias voltage and the instantaneous sensor resistance/current according to $P_{sensor} = V_{bias} \times I_{sensor}$ (Supplementary Tab. 7). This value reflects only the intrinsic power consumption of the sensing layer.

To quantify the system-level power consumption, the total supply current of the electronics and wireless communication module was measured under the same operating conditions and data-transmission settings as used in the experiments. With a regulated supply voltage of 2.5 V, the module draws an average current of 8.19 mA, corresponding to a total power consumption of approximately 20.48 mW. This value inherently includes the duty-cycled operation of ADC sampling, microcontroller processing, and Bluetooth wireless transmission.

Accordingly, the sensing-element power represents only a negligible fraction of the overall device power budget, while the dominant contribution arises from the communication and control electronics.

Comment 12. Does H_2S exposure affect the stability of the electronics (corrosion, signal noise, leakage currents)?

Reply: We sincerely thank the reviewer for raising this important question. Regarding the electronics, we have conducted extensive cycle testing under low-to-moderate H_2S concentrations (up to 1000 ppm) and observed no signs of instability, such as abnormal signal shifts, increased noise, or leakage currents. The device functioned reliably throughout the repeated H_2S exposure cycles, indicating that H_2S exposure, under the tested concentrations, does not adversely affect the electronics.

Currently, the electronic circuits are not encapsulated; however, the absence of corrosion or instability after extended testing suggests that no immediate corrosion risks are present at the low-to-moderate concentrations of H_2S encountered during the tests. Should a more corrosive environment be encountered in future applications, the electronics and sensor can be easily separated physically, with a simple two-wire connection between the sensor head and the readout, allowing the electronics to be protected by appropriate measures (e.g., encapsulation, sealing).

Additionally, material and sensors stability under H_2S exposure was previously verified using TEM, XRD, and EDX (Supplementary Fig. 12), confirming that the sensing material remains chemically and structurally stable under the tested conditions.

These new results and the associated discussion have been added to the Supplementary Information (Supplementary Fig. 12) and are also highlighted in red accordingly.

Revised parts of SI are as follows:

To examine whether long-term operation induces sulfidation or secondary-phase formation, the sensing layer after the stability campaign was characterized by HRTEM, PXRD, XPS, and EDX elemental mapping. HRTEM images revealed clear lattice fringes characteristic of ZnO and no ZnS crystalline domains, confirming that the ZnO structure remains intact at the nanoscale (Supplementary Fig. 12a). PXRD patterns did not show any additional peaks corresponding to ZnS, excluding the formation of the ZnS crystalline phase (Supplementary Fig. 12b). XPS analysis revealed a weak sulfur signal in the S 2p region at 169 eV, characteristic of SO_4^{2-} species, but no peaks indicative of ZnS formation were observed (Supplementary Fig. 12c). EDX mapping confirmed the absence of sulfur-rich ZnS domains or phase segregation (Supplementary Fig. 12d). These results collectively confirm that the long-term sensing process under our testing conditions is not accompanied by detectable bulk ZnS formation.

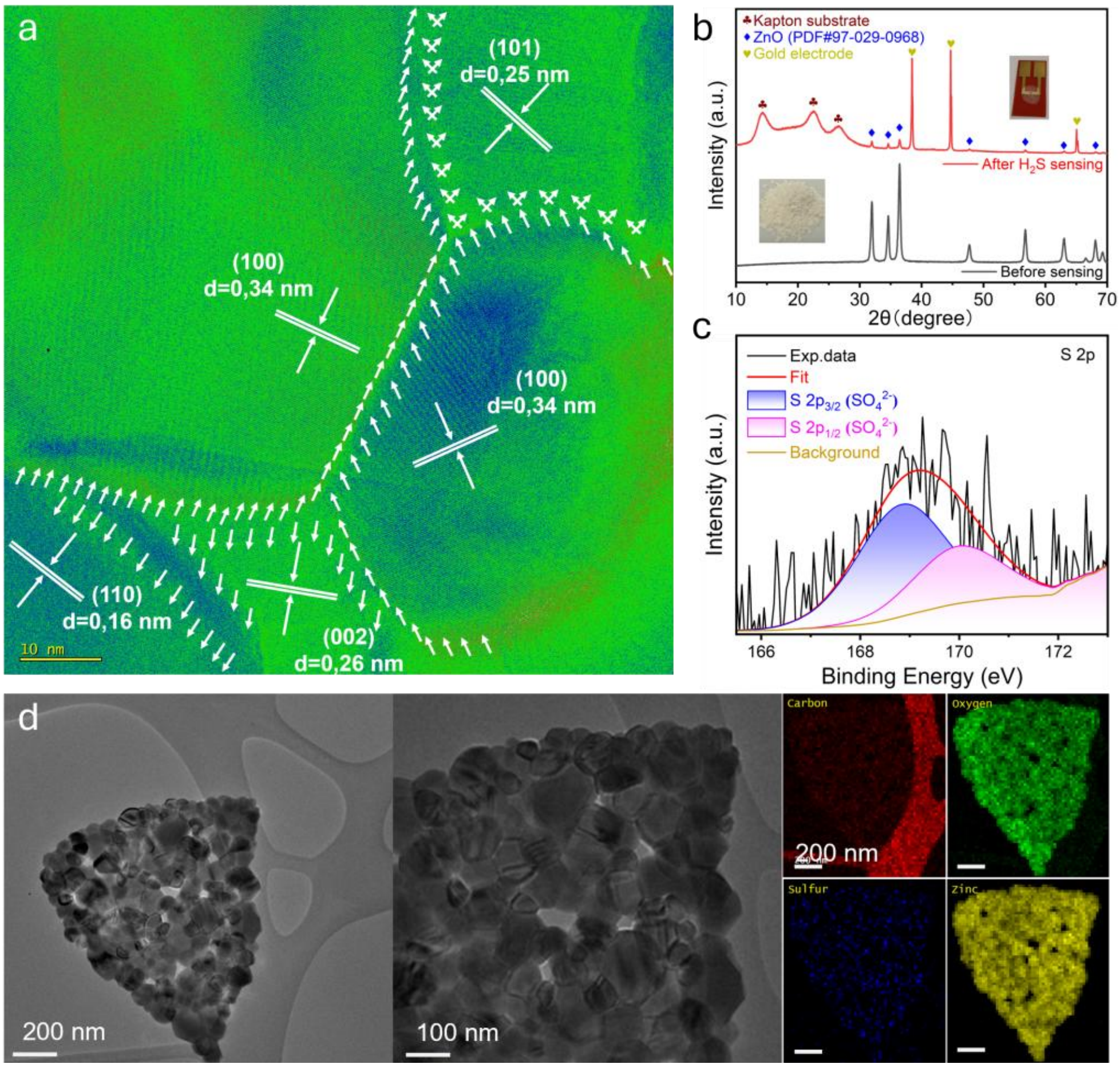

Supplementary Fig. 12 | HRTEM, PXRD, XPS, and EDX characterization of the sensing layer after long-term exposure to H_2S. **a,** HRTEM image showing lattice fringes characteristic of ZnO without ZnS formation. **b,** PXRD pattern confirming no ZnS phase formation. **c,** XPS S 2p spectrum revealing SO_4^{2-} species and the absence of ZnS peaks. **d,** EDX elemental mapping showing homogeneous elemental distribution without sulfur-rich ZnS domains.

Comment 13. The leakage test uses a localized defect at 50 ppm release. How does the sensor behave at realistic diffusion gradients or turbulent flow conditions?

Reply: We sincerely thank the reviewer for raising an important point about airflow disturbances and their impact on sensor behavior under realistic conditions. In the revised manuscript, we have expanded our evaluation to better simulate industrial leak-monitoring scenarios that involve airflow perturbations and high-concentration H_2S exposure.

Regarding the concern about airflow disturbances, we simulated industrial airflow perturbations by fixing the sensor-to-leak distance at 1.0 m and applied sequential conditions: still air → weak airflow →

weak airflow + dust → strong airflow. An axial fan (diameter ~10 cm) was placed between the sensor and the gas source, operating at two voltage levels (8 V and 15 V) to simulate weak and strong convection. Specifically, at 8 V, the fan generates a weak convection velocity of approximately v_{weak} = 0.5 m/s, and at 15 V, a strong convection velocity of v_{strong} = 1.2 m/s, with time averages taken over ≥30 s. Under weak airflow-only conditions, baseline fluctuations increase and the apparent response amplitude is partially reduced; the strongest airflow produces the largest noise. For dust interference, 20 mg dried, sieved soil powder was placed at the fan inlet and naturally entrained under weak airflow (30 s × 2 pulses). Although the curves exhibit larger noise during dust exposure, clear response and recovery features remain identifiable (details in Supplementary Fig. 35e-g), indicating robustness against combined airflow and dust disturbances.

Additionally, we expanded the distance testing to evaluate the sensor's performance over a more realistic range of distances. The sensor was placed at 0.50 m, 1.0 m, 3.0 m, and 5.0 m from the leak outlet in still air, with all other parameters kept constant. As expected, the response decreased with increasing distance due to dilution, but even at 5 m, a weak yet identifiable response was still observed, demonstrating the sensor's ability to detect leakage over meter-scale distances (details in Supplementary Fig. 35a-d).

To assess the high-concentration H_2S robustness, we exposed the sensor to 200, 400, 600, 800, and 1000 ppm H_2S and recorded the corresponding response/recovery curves. The sensor exhibited reversible responses without observable saturation up to 1000 ppm, with higher concentrations producing larger response amplitudes and improved signal-to-noise ratios. Importantly, no irreversible baseline drift was observed, indicating that the sensor is resistant to short-term high-concentration shocks under practical conditions (details in Supplementary Fig. 36).

These new results have been added to the Supplementary Information (Supplementary Fig. 35 and Fig. 36) and explicitly referenced in the revised manuscript (Page 16, Line 520-526), and are also highlighted in red accordingly.

Revised parts of manuscript are as follows:

To better simulate practical leak-monitoring conditions, the sensor under several realistic scenarios were evaluated. Beyond the original 5 cm condition, the sensor was positioned at 0.50 m, 1.0 m, 3.0 m, and 5.0 m from the leak outlet in still air. To simulate industrial airflow disturbances, the sensor was fixed at 1.0 m from the leak outlet and exposed to weak airflow, weak airflow with dust, and strong airflow. These results emphasize the sensor's effectiveness in realistic conditions, ensuring early leak detection and reducing safety and economic risks (details in Supplementary Fig. 35).

To evaluate sensing behavior at high H_2S concentrations (>100 ppm) in industrial scenarios, the sensor at 200, 400, 600, 800, and 1000 ppm was measured. The baseline returned without irreversible drift, indicating the sensor's short-term tolerance to high-concentration shocks under the tested conditions (details in Supplementary Fig. 36).

Revised parts of SI are as follows:

To better simulate practical leak-monitoring conditions, the sensor under several realistic scenarios were evaluated, including (i) long-distance detection (0.50-5.0 m), (ii) airflow and dust disturbances under forced convection, and (iii) high-concentration H_2S robustness (200-1000 ppm) to assess saturation and irreversible damage.

Long-distance detection was examined by positioning the sensor at 0.50 m, 1.0 m, 3.0 m, and 5.0 m from the leak outlet under still-air conditions (fan off), while keeping all other operating parameters unchanged. As expected, the response decreased with distance, but a noticeable response was still observed at 5.0 m, demonstrating the sensor's ability to detect leaks over meter-scale distances (Supplementary Fig. 35a-d).

To simulate industrial airflow perturbations, we fixed the sensor-to-leak distance at 1.0 m and four sequential conditions were applied: (i) still-air baseline; (ii) weak forced convection generated by a miniature DC fan powered at 8 V; (iii) weak convection with dust challenge, implemented by placing 20 mg of dried, sieved soil powder at the fan inlet for natural entrainment (30 s per pulse, two pulses, separated by baseline recovery); and (iv) strong forced convection generated by increasing the fan voltage to 15 V. Airflow disturbances were introduced using an axial fan (diameter ~10 cm), placed between the sensor and the gas source, operating at two voltage levels (8 V and 15 V) to simulate weak and strong convection. Specifically, at 8 V, the fan generates a weak convection velocity of approximately v_{weak} = 0.5 m/s, and at 15 V, a strong convection velocity of v_{strong} = 1.2 m/s, with time averages taken over ≥30 s.

Under weak airflow-only conditions, baseline fluctuations increase and the apparent response amplitude is partially reduced; the strongest airflow produces the largest noise. For dust interference, 20 mg dried, sieved soil powder was placed at the fan inlet and naturally entrained under weak airflow (30 s × 2 pulses). Despite increased noise during dust exposure, the response and recovery features remained identifiable, indicating the sensor’s robustness against combined airflow and dust disturbances (Supplementary Fig. 35e-g), indicating robustness against combined airflow and dust disturbances.

High-concentration robustness was evaluated by exposing the sensor to 200, 400, 600, 800, and 1000 ppm H_2S. The sensor exhibited rapid and reversible response/recovery across this range, with an improved signal-to-noise ratio at higher concentrations and no saturation up to 1000 ppm. The baseline returned without irreversible drift, indicating the sensor's short-term tolerance to high-concentration shocks under the tested conditions (Supplementary Fig. 36).

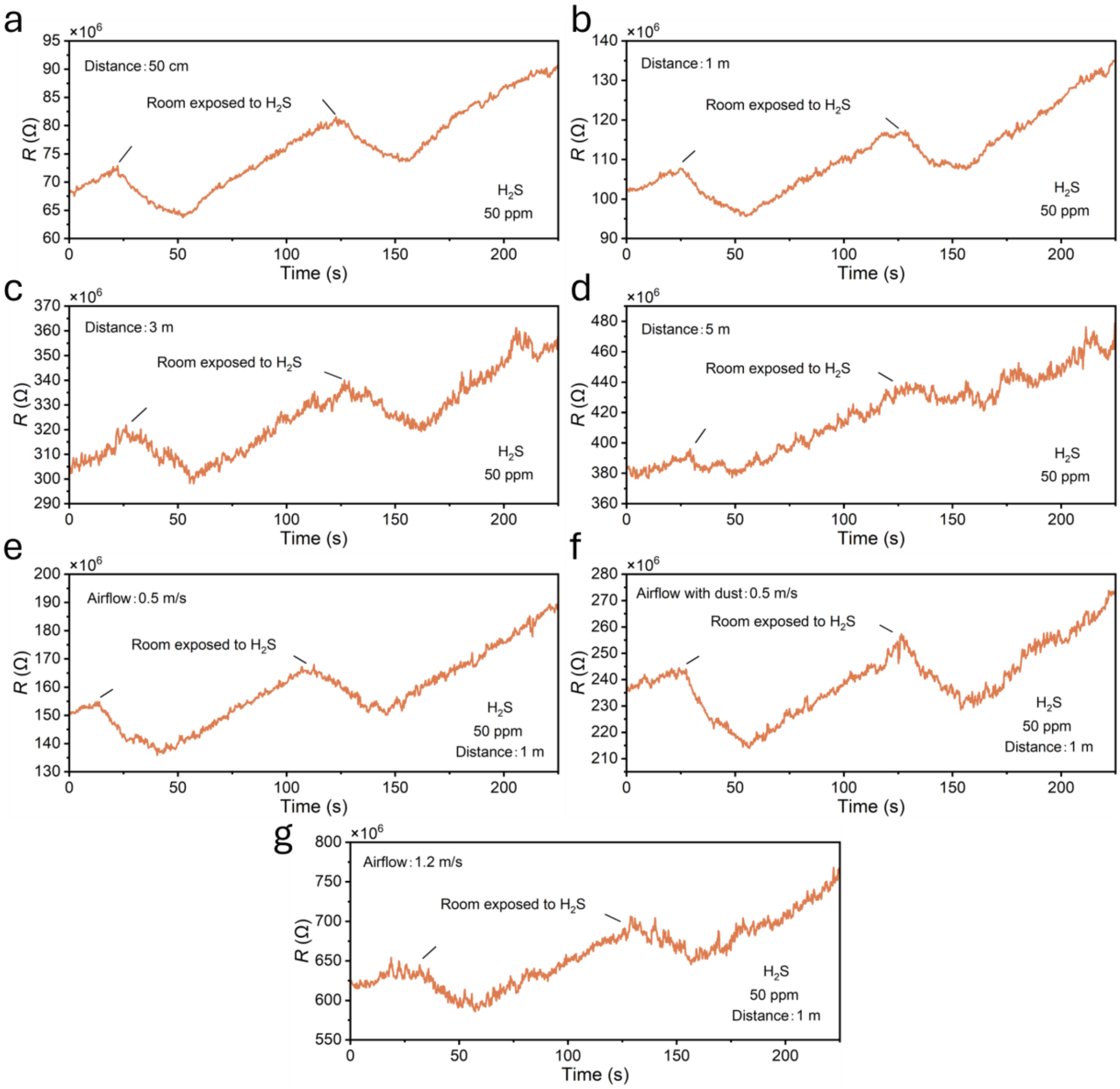

Supplementary Fig. 35 | a-d, Response and recovery curves at different distances from the leak outlet: 0.50 m, 1.0 m, 3.0 m, and 5.0 m. **e-g,** Response and recovery curves under different airflow and dust disturbance conditions at a fixed distance of 1.0 m from the leak outlet: **e,** under 0.5 m/s weak airflow; **f,** under 0.5 m/s weak airflow with dust interference (20 mg dried, sieved soil powder); **g,** under 1.2 m/s strong airflow.

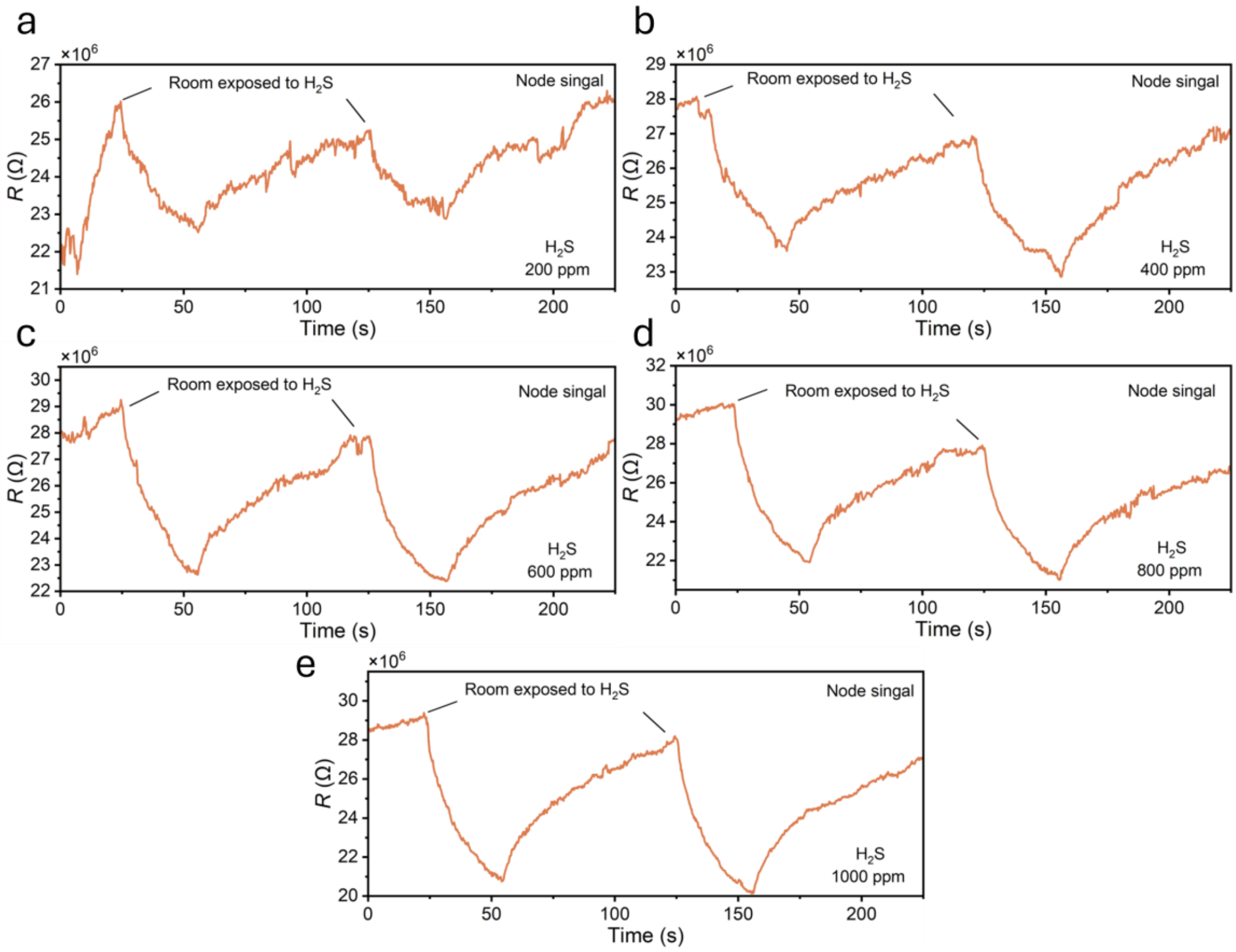

Supplementary Fig. 36 | a-e, Response and recovery curves under high concentrations of H_2S: 200 ppm, 400 ppm, 600 ppm, 800 ppm, 1000 ppm.

Comment 14. The confined chamber demonstration uses a uniform 1 ppm distribution. Can the sensor detect spatial gradients or very slow accumulation rates typical of real industrial settings?

Reply: We sincerely thank the reviewer for raising an important point about the sensor's ability to detect spatial gradients and very slow accumulation rates typical of real industrial environments. To address this concern, we conducted two experiments to evaluate the sensor's performance under conditions of spatial concentration gradients and continuous low- to high-concentration exposure.

In the first experiment, we evaluated the sensor's response to spatial concentration gradients by incrementally introducing low concentrations of H_2S (1 ppm, 2 ppm, 3 ppm, 4 ppm, 5 ppm) over short durations, followed by recovery periods. In the second set, we examined the sensor's performance under continuous increasing concentrations (10 ppm, 50 ppm, 100 ppm, 500 ppm, 1000 ppm) to simulate slow accumulation rates typical of industrial environments. The results demonstrate that the sensor can detect both spatial gradients and slow concentration increases without saturation or irreversible damage, confirming its robustness for real-world applications.

These new results and the associated discussion have been added to the Supplementary Information (Supplementary Fig. 37) and explicitly referenced in the revised manuscript (Page 17, Line 535-539) and are also highlighted in red accordingly.

Revised parts of manuscript are as follows:

Furthermore, the sensor was measured under incremental low concentrations of H_2S (1, 2, 3, 4, 5 ppm) with short time recovery, as well as under continuously high H_2S concentrations (10, 50, 100, 500, 1000 ppm) to simulate spatial gradients and slow accumulation rates. The results demonstrate that the sensor reliably handles both spatially non-uniform concentration distributions and slow accumulation rates. (details in Supplementary Fig. 37).

Revised parts of SI are as follows:

The sensor was tested under incremental low concentrations of H_2S (1, 2, 3, 4, 5 ppm) with short recovery times, as well as under continuously increasing high concentrations (10, 50, 100, 500, 1000 ppm) to simulate spatial gradients and slow accumulation rates. In the first experiment, incremental low concentrations of H_2S (1, 2, 3, 4, 5 ppm) were introduced sequentially, each for 30 s, followed by a short recovery period, with the sensor's response and recovery monitored after each exposure. As shown in Supplementary Fig. 37a, the sensor demonstrated clear response and recovery characteristics, with a decrease in noise as the concentration increased, suggesting an improvement in the signal-to-noise ratio. In the second experiment, the sensor was exposed to continuously increasing high concentrations of H_2S (10 ppm, 50 ppm, 100 ppm, 500 ppm, 1000 ppm). As shown in Supplementary Fig. 37b, the sensor exhibited stepwise changes in response signal with increasing concentrations, reaching a stable response after exposure to 1000 ppm. The baseline returned after the exposure without irreversible drift, further confirming that the sensor remains unaffected by high concentrations of H_2S and does not exhibit saturation or irreversible damage.

These results confirm that the sensor effectively detects both spatial concentration gradients and slow accumulation rates, with no signs of saturation at high concentrations. The sensor's performance under these conditions validates its robustness and suitability for real-world industrial leak detection, where spatially non-uniform gas distributions and gradual accumulation are common.

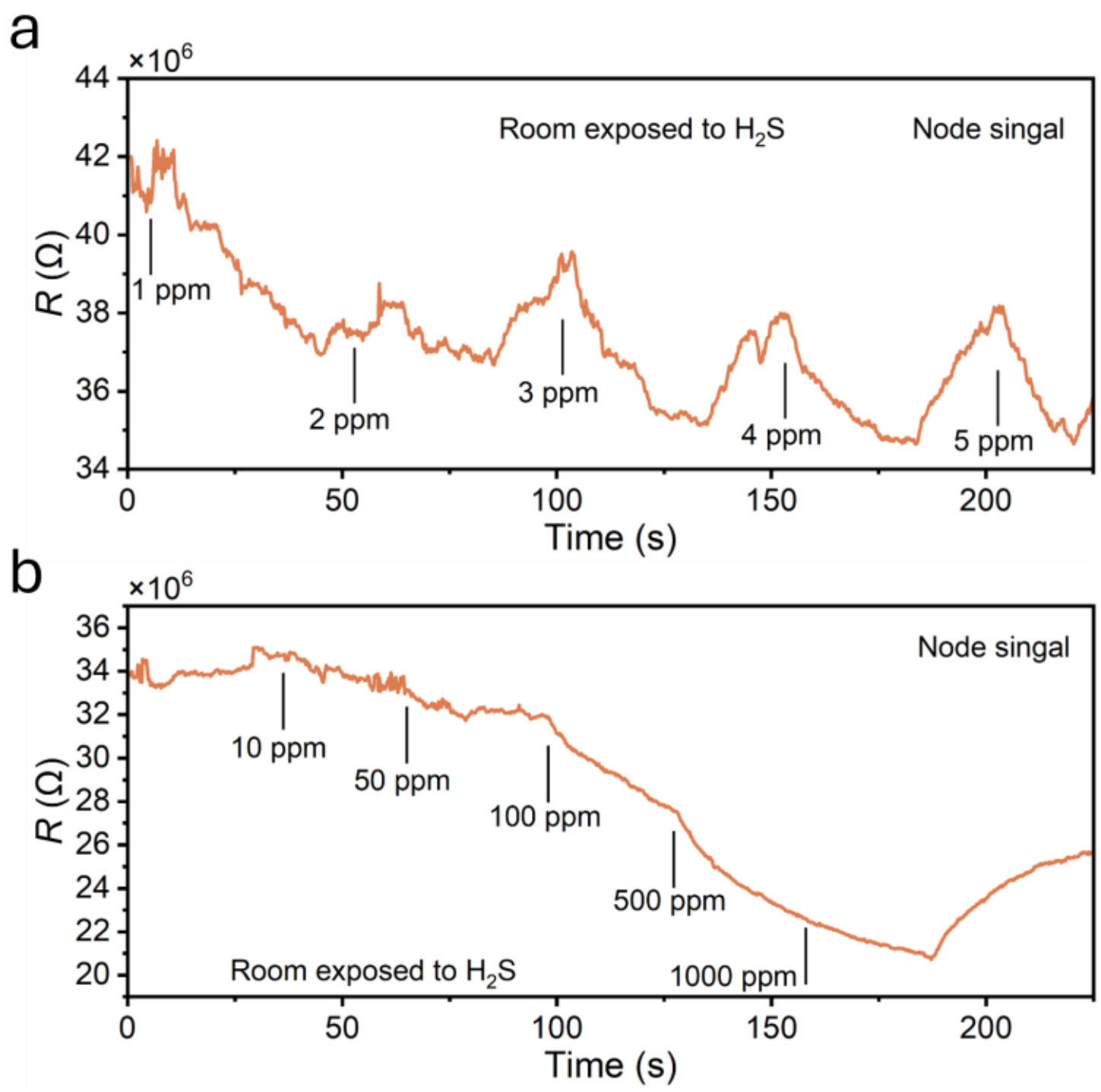

Supplementary Fig. 37 | a, Response and recovery characteristics of the sensor under incremental low concentrations of H_2S (1, 2, 3, 4, 5 ppm) with short recovery periods. **b,** Stepwise response of the sensor to continuously increasing H_2S concentrations (10, 50, 100, 500, 1000 ppm).

Comment 15. The grain-boundary models in DFT represent small oligomeric structures. How do these simplified structures reflect the realistic energetics of ZnO grain boundary? Have you validated the computational predictions with experimental barrier measurements at varying grain boundary densities?

Reply: We thank the reviewer for the insightful question regarding the representativeness of the DFT grain-boundary (GB) models and the experimental validation of GB-induced barrier effects. We agree that real ZnO grain boundaries can be structurally complex; therefore, the DFT GB models employed in this work are intentionally simplified and should be regarded as representative atomic-scale motifs designed to capture the common energetic features of GB cores, namely under-coordinated sites and trap-prone environments that dominate adsorption energetics and local barrier modulation.

To experimentally validate the relevance of GB effects at the device level, we systematically tuned the GB density by varying the pyrolysis temperature and quantified the resulting microstructural evolution using EBSD. The EBSD statistics reveal that increasing pyrolysis temperature leads to progressive grain growth and a monotonic decrease in GB density. Importantly, UPS measurements performed on the same set of samples demonstrate a consistent reduction in the Schottky barrier height with decreasing GB density. By

directly correlating EBSD-derived GB density with UPS-derived barrier height (Supplementary Fig. 19), we establish a quantitative structure-barrier relationship across the temperature series.

These results demonstrate that GB density critically governs the interfacial barrier landscape, thereby providing direct experimental validation for the GB-assisted barrier modulation mechanism inferred from DFT. We further clarify that while UPS-derived barrier values reflect the interfacial electronic structure under pristine conditions, they serve to establish inter-sample correlations; the barrier modulation discussed in the sensing operation corresponds to the effective operating-state barrier of the Au-ZnO junction.

These new results and the associated discussion have been added to the Supplementary Information (Supplementary Fig. 19, Fig. 20 and Tab. 3) and explicitly referenced in the revised manuscript (Page 15, Line 469-490) and are also highlighted in red accordingly.

Revised parts of manuscript are as follows:

Optimal sensing performance is achieved through precise GB engineering, as exemplified by the ZMA550 sample, which reflects a balanced trade-off between carrier transport and surface reactivity rather than a non-monotonic optimization of the barrier height itself. This balance arises from three coupled factors: (1) a sufficient GB density to provide abundant adsorption and charge-trapping sites, (2) moderated GB density to avoid excessive carrier mobility degradation, and (3) the formation of a continuous conductive network that prevents severe carrier localization at intergranular potential barriers. Systematic microstructural analysis combining EBSD and UPS establishes a clear and monotonic structure-barrier relationship across samples pyrolyzed at 500-650 °C (Supplementary Fig. 19). Increasing pyrolysis temperature induces progressive grain growth and a corresponding reduction in GB density, which is accompanied by a systematic decrease in the Schottky barrier height derived from UPS. This trend is consistent with the established picture that GB-rich polycrystalline ZnO hosts stronger depletion and band bending due to a higher density of interfacial trap states. To complement these experimental observations, density functional theory (DFT) simulations were employed to elucidate the mechanistic role of GBs in adsorption-induced barrier modulation. The calculations reveal that GB-associated under-coordinated sites and defect-prone regions facilitate charge transfer and amplify local barrier modulation upon H_2S adsorption. Notably, the “optimal” sensing behavior arises from a device-level trade-off rather than from a non-monotonic dependence of the barrier height on GB density. Specifically, increasing GB density can enlarge the available barrier-modulation window, whereas an excessively high barrier elevates the baseline resistance and compromises response/recovery kinetics. Therefore, an intermediate GB density provides the most

favorable overall performance when sensitivity, transport efficiency, and dynamic characteristics are evaluated in combination. This synergistic interplay between GB-assisted adsorption and transport regulation enables exceptional room-temperature trace H_2S detection with high sensitivity and operational stability.

Revised parts of SI are as follows:

A direct correlation among grain size, GB density, and Schottky barrier height across samples prepared at different pyrolysis temperatures (Supplementary Fig. 19). The EBSD analysis was employed to quantify the grain size and grain-boundary density of ZnO sensing layers pyrolyzed at 450–650 °C. Increasing pyrolysis temperature induces progressive grain growth accompanied by a monotonic reduction in GB density (Supplementary Fig. 20 and Tab. 3). Samples pyrolyzed at ≤450 °C were excluded from quantitative EBSD analysis due to insufficient spatial resolution and indexing reliability (Supplementary Fig. 20).

UPS measurements (Supplementary Fig. 18) performed on the same set of samples reveal a systematic decrease in the Schottky barrier height derived from the work function and valence band onset with decreasing GB density (Supplementary Tab. 3). By correlating EBSD-derived microstructural parameters with UPS-derived electronic structure metrics, we establish a quantitative structure–barrier relationship across the temperature series.

It should be noted that UPS-derived barrier values reflect the interfacial electronic structure under pristine surface conditions and are used here to establish inter-sample trends. In contrast, the barrier modulation discussed in the sensing operation corresponds to the effective operating-state barrier of the Au–ZnO junction under gas exposure. While absolute barrier values under these conditions are not expected to coincide, they provide complementary insights into the structure–property relationship governing the sensing mechanism.

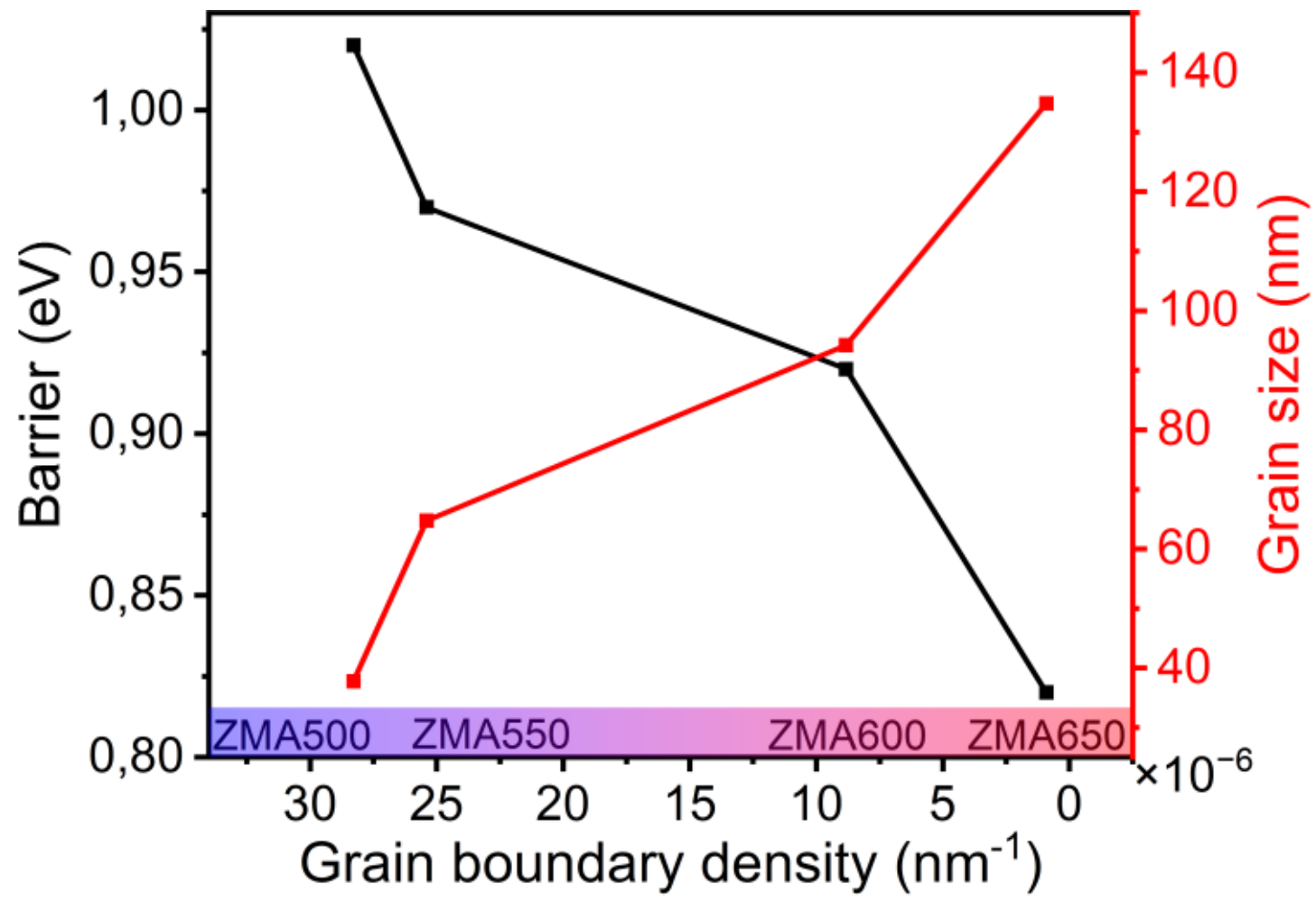

Supplementary Fig. 19 | Grain size and grain boundary density extracted from EBSD, and Schottky barrier height derived from UPS for samples pyrolyzed at 500–650 °C.

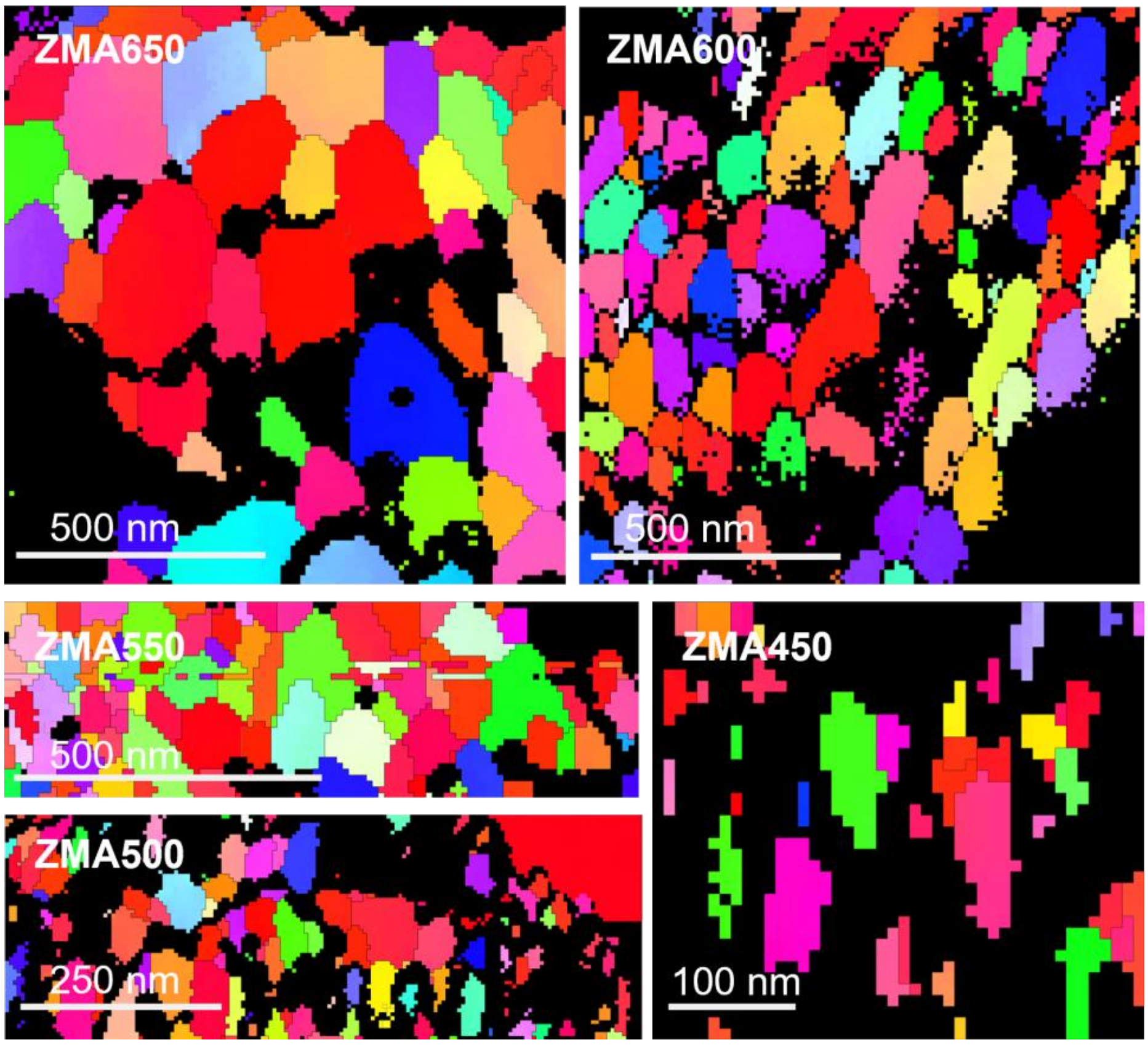

Supplementary Fig. 20 | EBSD images of samples pyrolyzed at 450–650 °C.

Supplementary Tab. 3 | Grain boundary concentration and density of samples pyrolyzed at 500–650 °C.

Samples	Total length of GB/nm	Area /nm^2	GB density/nm^{-1}
ZMA 500	6.08148	215024.9500	2.82827×10^{-5}
ZMA 550	10.14060	399185.0975	2.54033×10^{-5}

ZMA 600	11.96009	1355545.3960	8.82308×10^{-6}
ZMA 650	11.50177	12590887.6200	9.13500×10^{-7}

Comment 16. Some materials in Tab. S1 show comparable high responses. Can the authors more rigorously justify the claim of being state-of-the-art, perhaps by normalizing metrics (response per surface area, per power, per LOD)?

Reply: We thank the reviewer for this valuable suggestion. To strengthen the rigor of the benchmarking, we have augmented Table S1 by adding a normalized sensitivity metric, S/LOD, calculated as the literature-reported response (S) divided by the corresponding LOD. This response-to-LOD ratio provides a quantitative joint metric that accounts for both response magnitude and detection limit without introducing additional assumptions about device geometry or surface area, which are inconsistently reported across studies. The updated Table S1 shows that the present device is positioned among the top-performing H_2S sensors when evaluated using this normalized metric, thereby providing a more rigorous and transparent basis for our "state-of-the-art" claim. The manuscript has been revised accordingly to reference this updated analysis (Supplementary Tab. 1).

These new results and the associated discussion have been added to the Supplementary Information (Supplementary Tab. 1) and explicitly referenced in the revised manuscript (Page 5, Line 168-175) and are also highlighted in red accordingly.

Revised parts of manuscript are as follows:

To substantiate the advancement, a meta-analysis was conducted across four key performance metrics: operating temperature, responsivity, response time, and limitation of detection (LOD), based on representative literature. As shown in Fig. 1e, pairwise comparisons reveal dual breakthroughs: (1) ultra-trace H_2S detection down to 0.328 ppb at room temperature, and (2) a responsivity that ranks among the highest reported values to date. To further strengthen the quantitative basis of this benchmarking, we additionally extracted a normalized sensitivity metric (S/LOD, response-to-LOD ratio) directly from the literature data (Supplementary Tab. 1), which jointly accounts for response magnitude and detection limit and places the present device among the state-of-the-art H_2S sensors.

Revised parts of SI are as follows:

Supplementary Tab. 1 summarizes representative H_2S sensors reported in the literature using key operating and performance metrics. To enable a more rigorous quantitative comparison beyond response

alone, we introduce a normalized sensitivity metric, S/LOD, defined as the reported response (S, using each study's original response definition) divided by the corresponding LOD. This response-to-LOD ratio provides a joint metric reflecting response magnitude and detection limit based solely on literature-reported values, without requiring additional normalization to surface area or device geometry, which are often not consistently available across studies. The resulting S/LOD values highlight that the present device is competitive at the top tier among reported H_2S sensors when considering both response and detection limit simultaneously.

Supplementary Tab. 1 | Literature benchmarking of H_2S sensors with key metrics and normalized sensitivity (S/LOD).

Materials	T (°C)	Conc. (ppm)	Response (%)	LOD (ppm)	Res time	Response def. (S =)	Normalized sensitivity (S/LOD)	Ref
**ZnO nanosheet**	**23**	**100**	**13448.9**	**0.000328**	**7s**	**R_a/R_g-1**	**4.10×10^7**	**This work**
ZnO nanorods	25	0.05	1.7	-	23 min	R_a/R_g	-	[5]
Flower-like ZnO	26	5	581	-	320s	I_g/I_a	-	[6]
ZnO nanobelts	28	10	8	0.5	~100 s	R_a/R_g	16	[7]
$Cs_2AgBiCl_6$	RT	10	90.6	0.005	99.6	$(I_g-I_a)/I_a$	1.81×10^4	[8]
Cu_2O/ZnO	RT	10	42	0.01	300	$(I_g-I_a)/I_a$	4.20×10^3	[9]
F_4-TCNQ/C_{10}-DNTT	RT	5	138	0.28	27.2 mins	$(\Delta R/R_0)$	4.93×10^2	[10]
CdS@PPy/rGO	RT	70	98.98	-	128	$(R_g - R_a) / R_a$	-	[11]
Ag@WO_3	RT	10	11420	0.01	7s	R_g/R_a	1.14×10^6	[12]
ZnO dendrites	30	100	17.3	10	15 s	R_a/R_g	1.73	[13]
ZnO nanorods	50	100	61.7	-	4s	-	-	[14]
ZnO nanosheet	70	100	23	5	252s	R_a/R_g	4.6	[15]
ZnO flowers	92	100	157.3	0.05	57.8s	R_a/R_g	3.15×10^3	[16]
ZnO nanorods	100	100	2.6	-	-	R_a/R_g	-	[17]
SnO_2	125	5	335	0.5	224	R_{air}/R_{gas}	670	[18]

$MgFe_2O_4/MoO_3$	135	7	31.1	-	108	$(R_a-R_g)/R_a$	-	[19]
ZnO tubes	150	450	15	-	-	R_a/R_g	-	[20]
ZnO nanowire	150	20	0.9	0.05	3min	$(R_a-R_g)/R_a$	18	[21]
Lettuce-like ZnO	150	100	113	1	15s	R_a/R_g	113	[22]
Ru/SnO_2	160	20	310.1	100	<1	R_{air}/R_{gas}	3.10	[23]
α-Fe_2O_3	175	10	63.55	0.0025	82.7	$(R_a-R_g)/R_a$	2.54×10^4	[24]
CuO/CeO_2	180	100	2010	10	39	R_{air}/R_{gas}	201	[25]
Flower-like ZnO	180	50	42.2	1	14s	R_a/R_g	42.2	[26]
ZnO nanosheet	200	50	2180	0.01	3.4s	R_a/R_g	2.18×10^5	[27]
Mo doped $BiVO_4$	200	10	43.5	20.5	11	R_g/R_a	2.12	[28]
CuO/ZnO	200	5	83.5	tens	572	R_{air}/R_{gas}	-	[29]
WO_3/SnO_2	200	1	177	-	-	R_{air}/R_{gas}	-	[30]
ZnO hollow tubules	217	50	5.5	0.01	29s	R_a/R_g	550	[31]
Sphere-like ZnO	220	5	230	0.05	219 s	R_a/R_g	4.60×10^3	[32]
ZnO nanoflakes	250	100	94	-	-	$(R_a-R_g)/R_a$	-	[33]
α-Fe_2O_3	300	10	5.31	0.05	30	R_{air}/R_{gas}	106	[34]
ZnO	300	50	0.85	-	13s	$\Delta G/\Delta G_0$	-	[35]
ZnO	300	20	80	-	35s	$(R_a-R_g)/R_a$	-	[36]
ZnO nanoclusters	330	0.5	3.3	0.0625	10s	R_a/R_g	52.8	[37]
ZnO chains	400	20	6300	5	-	$\Delta G/\Delta G_0$	1.26×10^3	[38]
ZnO nanorods	500	50	35	-	200s	$(I_g-I_a)/I_a$	-	[39]

Note: G = conductance; R = resistance; I = current; Oxygen species+H_2S mean that H_2S reacts with chemical abs oxygen, also react with ZnO to produce ZnS. Normalized sensitivity (S/LOD) is calculated as the reported response divided by the corresponding LOD (both taken directly from the literature), serving as a joint metric reflecting response magnitude and detection limit.

Comment 17. H_2S can convert ZnO to ZnS. Has the material been examined post-measurement (XRD, XPS) to ensure no irreversible ZnS formation occurred?

Reply: We sincerely thank the reviewer for raising this important point regarding potential ZnS formation during H_2S exposure. To address this, we performed post-measurement structural and chemical characterizations using HRTEM, PXRD, XPS, and EDX on the sensing layer after long-term exposure to H_2S.

HRTEM images of the sensing material after the stability tests revealed clear lattice fringes and well-defined grain boundaries, characteristic of ZnO, with no indication of any ZnS crystalline domains. This suggests that the ZnO structure remains intact at the nanoscale (Supplementary Fig. 12a). PXRD analysis further confirmed that no new diffraction peaks corresponding to ZnS were observed, ruling out the formation of ZnS as a crystalline phase (Supplementary Fig. 12b). XPS analysis of the sensing layer revealed a weak S 2p signal near 169 eV, characteristic of SO_4^{2-} species. This indicates the presence of sulfur in the sensing layer, but the absence of peaks associated with ZnS formation (Supplementary Fig. 12c). The S 2p signal likely originates from the formation of $ZnSO_4$ or other sulfate-based phases, rather than ZnS. EDX elemental mapping of the sensing layer confirmed that no sulfur-rich domains or ZnS crystalline phases were present, further supporting the conclusion that ZnS did not form during the H_2S sensing operation (Supplementary Fig. 12d). Together, these results provide direct evidence that no irreversible ZnS formation occurred during long-term sensing operation and that the sulfur species present are likely part of sulfate phases (e.g., $ZnSO_4$) rather than ZnS.

These new results and the associated discussion have been added to the Supplementary Information (Supplementary Fig. 12) and explicitly referenced in the revised manuscript (Page 8, Line 243-249) and are also highlighted in red accordingly.

Revised parts of manuscript are as following:

Furthermore, the long-term stability was evaluated by periodic measurements over 450 days (monthly tests under identical sensing conditions) rather than continuous operation (Fig. 2g). Remarkably, monthly stability tests conducted thereafter show negligible signal degradation over an extended period, underscoring the sensor's reliability and suitability for long-term deployment in real-world environments. Post-test optical inspection and structural/chemical characterizations (HRTEM, PXRD, XPS, and EDX) confirm the integrity of the electrodes and exclude detectable secondary-phase formation (e.g., ZnS) after the stability campaign (Supplementary Fig. 12).

Revised parts of SI are as following:

To examine whether long-term operation induces sulfidation or secondary-phase formation, the sensing layer after the stability campaign was characterized by HRTEM, PXRD, XPS, and EDX elemental mapping. HRTEM images revealed clear lattice fringes characteristic of ZnO and no ZnS crystalline domains, confirming that the ZnO structure remains intact at the nanoscale (Supplementary Fig. 12a). PXRD patterns did not show any additional peaks corresponding to ZnS, excluding the formation of the ZnS crystalline phase (Supplementary Fig. 12b). XPS analysis revealed a weak sulfur signal in the S 2p region at 169 eV, characteristic of SO_4^{2-} species, but no peaks indicative of ZnS formation were observed (Supplementary Fig. 12c). EDX mapping confirmed the absence of sulfur-rich ZnS domains or phase segregation (Supplementary Fig. 12d). These results collectively confirm that the long-term sensing process under our testing conditions is not accompanied by detectable bulk ZnS formation.

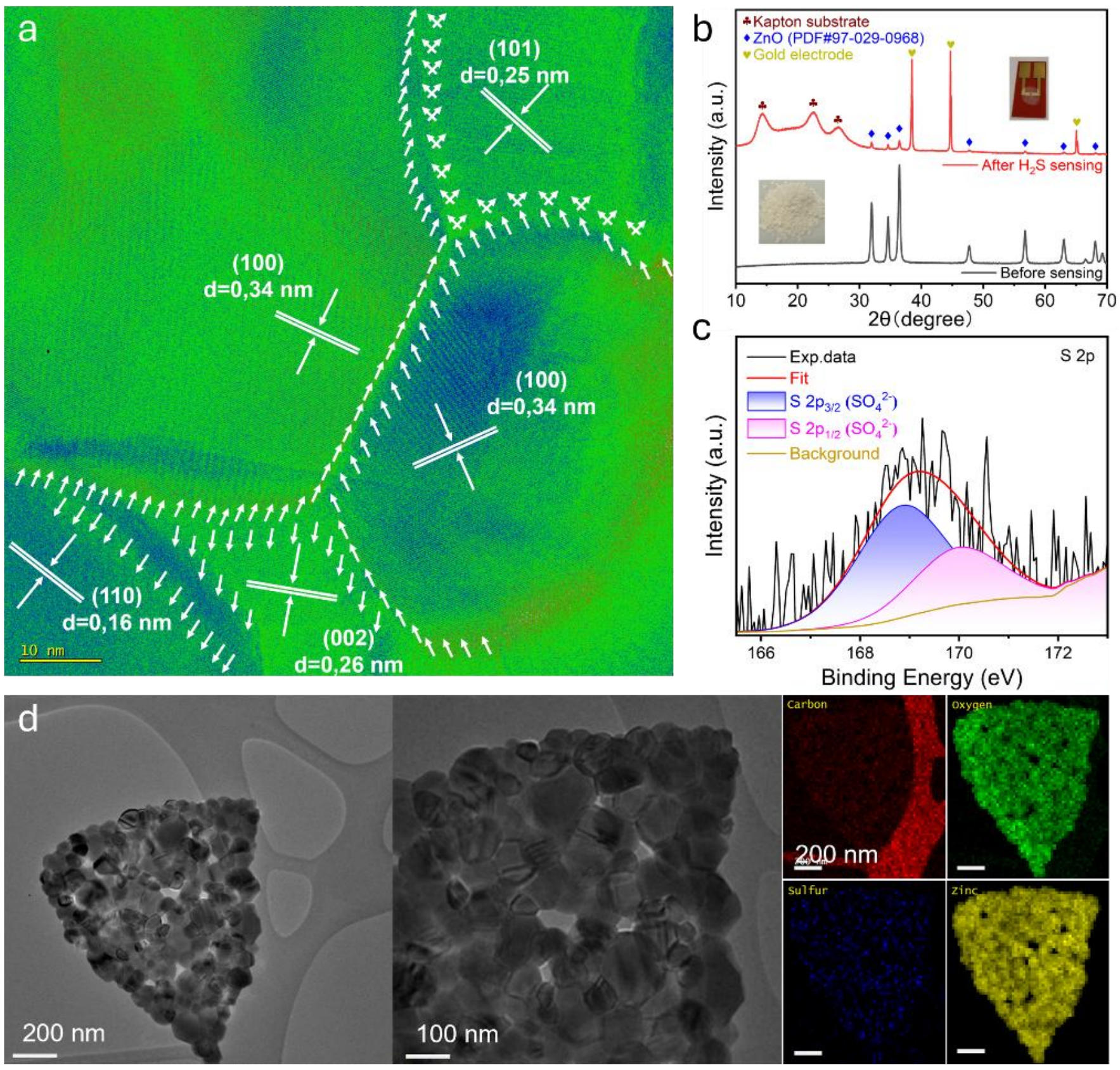

Supplementary Fig. 12 | HRTEM, PXRD, XPS, and EDX characterization of the sensing layer after long-term exposure to H_2S. **a,** HRTEM image showing lattice fringes characteristic of ZnO without ZnS formation. **b,** PXRD pattern confirming no ZnS phase formation. **c,** XPS S 2p spectrum revealing SO_4^{2-} species and the absence of ZnS peaks. **d,** EDX elemental mapping showing homogeneous elemental distribution without sulfur-rich ZnS domains.

Comment 18. The paper states sensors were fabricated "in triplicate." Can you provide statistical variation (standard deviation, error bars) for response values, LOD, and power consumption across multiple sensors?

Reply: We sincerely thank the reviewer for this thoughtful observation. We would like to clarify that the term "in triplicate" in the manuscript refers specifically to the three separate batches of sensors fabricated for this study, not the wireless sensing device. Therefore, power consumption was not measured as part of the sensor testing. We apologize for any confusion this may have caused and appreciate your understanding.

In response to your request for statistical variation, we have calculated the standard deviation (SD) and error bars for the response values and the Limit of Detection (LOD) across the three batches of sensors. The results are as follows:

Response values: The three sets of sensor response values are as follows: 529.52, 629.75, and 548.04. The standard deviation (SD) for these values is calculated to be 43.55. The error bars for the response values are derived from this SD, providing an estimate of the variation across the three sensor batches.

Limit of Detection (LOD): The LOD values for the three sensor batches are 0.0909 ppb, 0.0356 ppb, and 0.0729 ppb, respectively. The standard deviation (SD) for the LOD values is calculated to be 0.023. The error bars for the LOD are derived from this SD, reflecting the variation in the detection limits across the three batches.

These new results and the associated discussion have been added to the Supplementary Information (Supplementary Fig. 7) and explicitly referenced in the revised manuscript (Page 7-8, Line 237-238) and are also highlighted in red accordingly.

Revised parts of the manuscript are as follows:

A detailed analysis of the differences, including standard deviation comparisons, among sensors from three distinct batches is presented in Supplementary Fig. 7.

Revised parts of SI are as follows:

The statistical analysis for the three batches of sensors is summarized below, including the calculated response values, Limit of Detection (LOD), and the corresponding standard deviations (error bars). All experimental conditions were kept consistent, and the measurements were conducted in triplicate for each batch.

The response values for the three sensor batches are as follows: 529.52%, 629.75%, 548.04%

The standard deviation for these values is calculated to be 43.55.

The Limit of Detection (LOD) for each batch of sensors was calculated as follows: 0.0909 ppb, 0.0356 ppb, 0.0729 ppb

The standard deviation for these LOD values is 0.0230 ppb.

The response values show a reasonable variation between the three sensor batches, with a standard deviation of 43.55. This reflects the inherent variation that can occur between different batches of sensors, even when experimental conditions are held constant.

The LOD values, calculated based on the baseline stability and response sensitivity via using the same methodology, show a similar trend, with a standard deviation of 0.0230 ppb. These values are lower than the previous LOD of 0.328 ppb. This decrease can be attributed to improved baseline stability and enhanced response sensitivity. The baseline stability was improved by fitting the baseline data with a 5th-order polynomial, which minimized fluctuations and noise, resulting in smaller residuals. This allowed for more precise detection of small signal variations. Additionally, the increased response sensitivity, reflected in a steeper slope of the response-concentration curve, indicates that the sensor is now more responsive to small changes in concentration. These improvements in baseline stability and response sensitivity together contributed to the lower LOD values. Error bars representing the standard deviations for the response values and LOD values are shown in Supplementary Fig. 7. The error bars visually indicate the variations across the three sensor batches, providing a clear representation of the data's uncertainty.

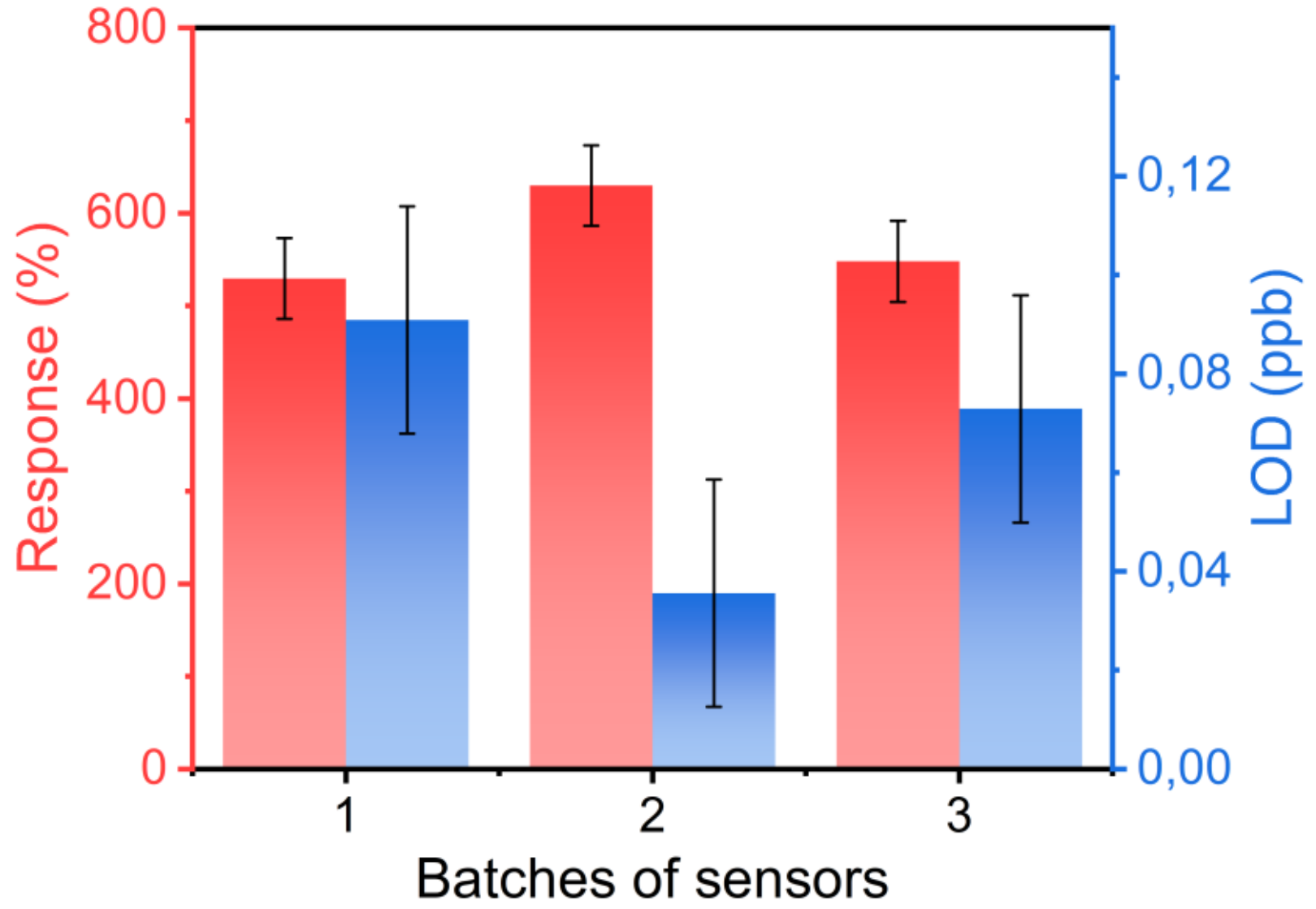

Supplementary Fig. 7 | Bar chart with of sensor response (left Y-axis) and LOD (right Y-axis) for three sensor batches.

Responses to the reviewers' comments

Manuscript ID: NCOMMS-25-71902A

Title: A robust high-performance H_2S sensor enabling real-world wireless monitoring

Editor's comment: Thank you again for submitting your revised manuscript "A robust high-performance H_2S sensor enabling real-world wireless monitoring" to Nature Communications. We have now received reports from two of the reviewers who evaluated the original version; apologies for the delay in this decision, as Reviewer 2 was not able to be reached, and we went back to Reviewer 1 to check their concerns and your responses. On the basis of this round of comments (copied below), we have decided to ask for an additional minor revision of your work.

You will see that, while the reviewers find that your revisions improved the manuscript, some important points remain to be addressed. Please revise your manuscript, addressing all the remaining issues raised by the reviewers.

Reply: Thank you for your constructive feedback on our manuscript titled "A robust high-performance H_2S sensor enabling real-world wireless monitoring." We appreciate the detailed insights provided by both the editor and the reviewers. After carefully reviewing the comments, we have addressed all concerns raised.

In response to the reviewers' suggestions, we have made the following updates:

1. Long-term cycling stability and reversibility of the sensing mechanism.

To directly examine whether long-term operation induces irreversible structural or surface chemical changes, we performed additional post-cycling TEM, SEM, XPS, and EDX characterizations on samples after the 450-day stability test. These measurements were used to assess possible grain boundary relaxation, surface sulfur-related species evolution, and changes in oxygen-related surface states after prolonged cycling. The combined results confirm that the grain morphology and grain-boundary framework remain stable, while only minor surface chemical evolution is observed, without measurable degradation of sensing performance. These findings provide direct support for the structural robustness and quasi-reversible interfacial sensing process under long-term use.

2. Experimental validation of the calculated LOD:

We sincerely thank the reviewer for suggesting sub-ppb level testing. While we recognize the importance of validating the sensor's performance at ultra-low concentrations (e.g., 0.1 ppb), the current gas-

mixing equipment has a practical limit, and we can reliably achieve 10-50 ppb H_2S for direct exposure testing under consistent experimental conditions.

Despite this limitation, the experimental results, including response/recovery curves and repeatability data over multiple cycles, provide robust evidence of the sensor's reliable performance at low ppb concentrations. These results further demonstrate that the sensor is capable of detecting ultra-low concentrations in the ppb range, even though we were unable to test below 10 ppb due to the equipment constraints. We believe that these experimental findings, in conjunction with the calculated LOD, provide a strong validation of the sensor's low-concentration detection capability under realistic experimental conditions.

3. DFT model clarification and structural differences:

We have clarified in both the manuscript and Supplementary Information the role of the simplified grain boundary/defect model and its ability to capture the essential physicochemical interactions responsible for sensing. We also emphasized that while the model simplifies the actual polycrystalline ZnO structure, it provides a representative description of the key mechanisms involved, supported by the consistency between computational results and experimental observations.

We have thoroughly revised the manuscript and Supplementary Information to incorporate these new experimental results and clarifications. **A detailed point-by-point response document has been provided**, addressing each comment in detail. All changes in the manuscript and Supplementary Information have been clearly highlighted in red.

We appreciate the constructive guidance from you and the reviewers. We are confident that the revised version will address all concerns comprehensively and significantly strengthen the manuscript

To Reviewer: 1

General comments: There is still some minor issues.

Reply: We sincerely thank the reviewer for the overall positive evaluation of our work and for recognizing the value of our efforts in developing a high-performance wireless H_2S sensor that integrates rational material design, significant performance advances, and practical real-world implementation. We appreciate your constructive insights, which have helped us further strengthen the depth and rigor of the manuscript. In the revised version, we have carefully addressed all your comments in a detailed point-by-point response that thoroughly explains how each issue has been resolved. All revisions in the manuscript have been highlighted in red. We are grateful for your thoughtful comments, which have substantially improved the quality and clarity of our work.

Comment 1: Could the authors please provide additional systematic characterization results, such as TEM and XPS, for the samples after the 450-day long-term cycling test? This would help verify whether phenomena such as surface sulfate accumulation, irreversible changes in oxygen vacancy concentration, and grain boundary relaxation occur during long-term cycling, thereby further confirming the reversibility and structural stability of the sensing mechanism under long-term use.

Reply: We thank the reviewer for this insightful suggestion. To further probe the long-term structural stability and reversibility of the sensing mechanism, additional TEM, SEM, EDX and XPS characterizations were conducted on the sensing layers after 450-day cycling tests.

TEM, SEM and EDX analyses (Supplementary Figs. 14a-e) consistently demonstrate that both the grain morphology and grain boundary configurations remain well preserved after prolonged operation, with no observable grain coarsening or boundary relaxation. This confirms that the microstructural framework governing carrier transport is structurally stable over extended cycling.

XPS measurements (Supplementary Figs. 14f–h) reveal detectable changes in the S 2p, O 1s, and Zn 2p spectra after long-term cycling. The S 2p spectra show variations in sulfur-related surface species without evidence of pronounced sulfur-species accumulation, while the O 1s spectra indicate modulation of oxygen-related surface states. In parallel, slight changes in the Zn 2p spectra suggest a corresponding adjustment of the local Zn chemical environment.

Importantly, despite these detectable surface chemical evolutions, no degradation in sensing performance is observed over 450 days. This indicates that the accumulated species do not irreversibly block active sites or disrupt interfacial charge transport. Instead, these results point to a quasi-reversible interfacial process, in which surface adsorbates may partially persist but remain dynamically exchangeable and do not compromise the functionality of grain boundaries or Schottky barriers.

Therefore, the combined structural and spectroscopic evidence demonstrates that the sensing mechanism is sustained by robust grain-boundary networks and dynamically stable interfacial electronic states, confirming its long-term operational stability.

These new results and the associated discussion have been added to the Supplementary Information (Supplementary Fig. 14) and explicitly referenced in the revised manuscript (Page 8, Line 245-255) and are also highlighted in red accordingly.

Revised parts of manuscript are as following:

Furthermore, the long-term stability was evaluated by periodic measurements over 450 days (monthly tests under identical sensing conditions) rather than continuous operation (Fig. 2g). Remarkably, the monthly stability tests show negligible signal degradation over the entire testing period, underscoring the sensor's reliability and suitability for long-term deployment in real-world environments. Post-test optical inspection and structural/chemical characterizations (HRTEM, PXRD, XPS, and EDX) confirm the integrity of the electrodes and exclude detectable secondary-phase formation (e.g., ZnS) after the stability campaign (Supplementary Fig. 13). In addition, post-cycling TEM, SEM, and XPS analyses further reveal that the grain morphology and grain-boundary network remain intact after 450-day operation, while only minor surface chemical evolution is observed without inducing measurable performance degradation (Supplementary Fig. 14). Together, these results confirm that the long-term sensing stability arises from both structural robustness and dynamically stable interfacial chemistry.

Revised parts of SI are as following:

The structural and surface evolution of the sensing layer after long-term cycling was systematically investigated. TEM, SEM and EDX images of the sensing layer after 450-day cycling tests (Supplementary Figs. 14a-e) show that the grain morphology and grain boundary network are well preserved, with no evidence of grain coarsening, boundary relaxation, or structural degradation. This indicates that the microstructural framework responsible for carrier transport remains stable during prolonged operation.

XPS analysis of the cycled samples (Supplementary Figs. 14f-h) further reveals detectable changes in the S 2p, O 1s, and Zn 2p spectra after long-term cycling. The S 2p spectra show variations in sulfur-related surface species, while the O 1s spectra indicate modulation of oxygen-related surface states. Meanwhile, slight changes in the Zn 2p spectra suggest a corresponding adjustment of the local Zn chemical environment. Together, these results point to mild surface chemical evolution during repeated sensing cycles, rather than severe irreversible chemical degradation.

Notably, despite the detectable accumulation of sulfur-containing species, the sensing performance remains highly stable over 450 days. This suggests that these species do not form an irreversible passivation layer or block active sites. Instead, they are likely weakly bound or dynamically exchangeable under operating conditions, maintaining access to reactive sites and preserving charge transport pathways.

Therefore, the sensing interface can be described as a quasi-reversible system, in which surface chemical states undergo continuous yet non-destructive evolution. The structural stability of grain boundaries, combined with the dynamic equilibrium of surface adsorbates, ensures that the interfacial barrier modulation and carrier transport mechanism remain functional during long-term operation.

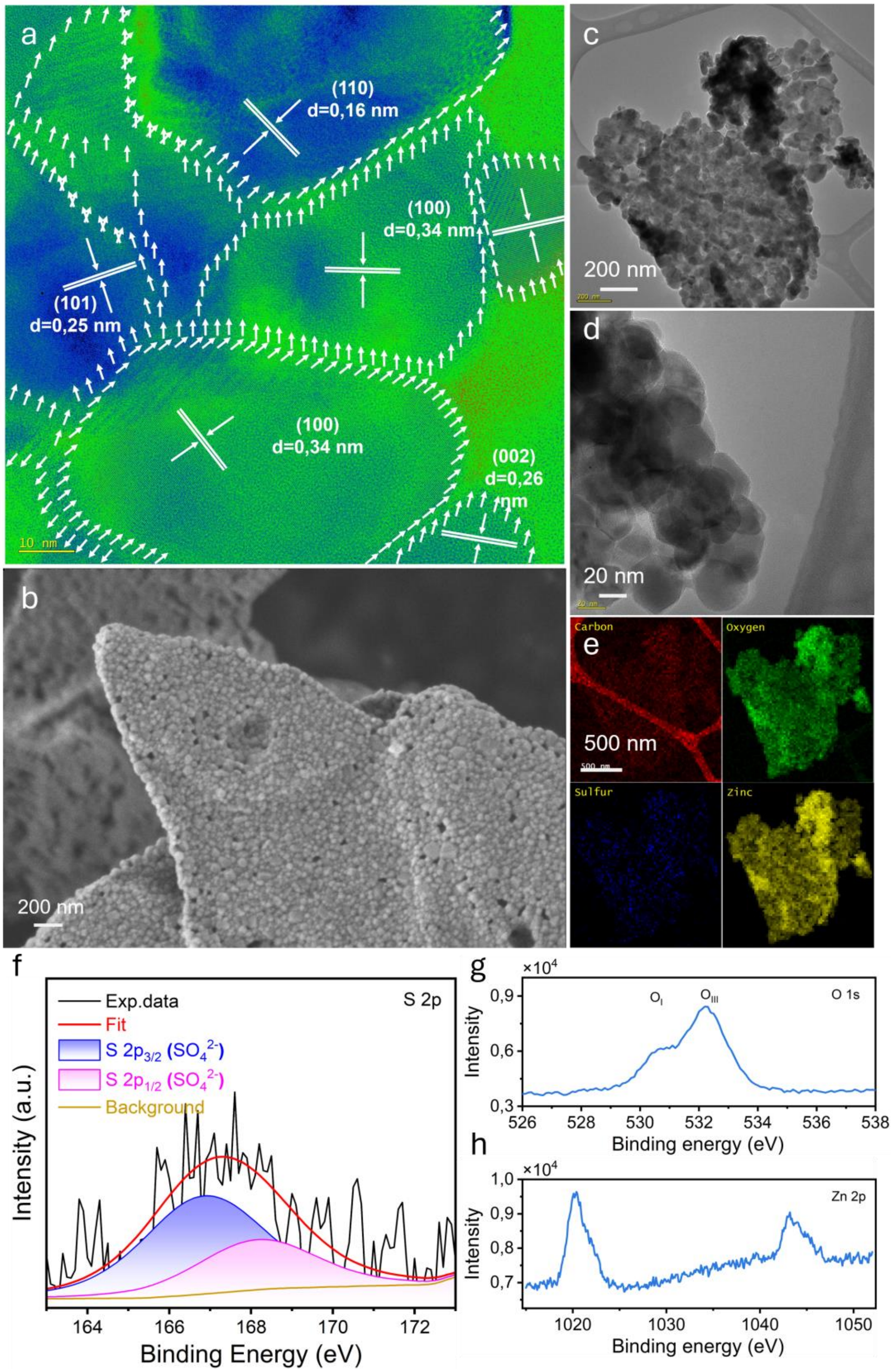

Supplementary Fig. 14 | HRTEM, SEM, EDX and XPS characterization of the sensing layer after the 450-day long-term cycling test. **a,** HRTEM image showing lattice fringes characteristic of ZnO. **b,** SEM images still keep nanosheet structure composed of ZnO particles. **c-e,** TEM and EDX elemental

mapping showing homogeneous elemental distribution. **f-h,** XPS S 2p, O 1s, and Zn 2p spectra revealing reversibility and structural stability of the sensing layer under long-term use.

Comment 2: How did the authors assess the impact of structural differences between the simplified grain boundary/defect model and actual polycrystalline ZnO on the computational results?

Reply: We thank the reviewer for this important question regarding the representativeness of the computational model. We acknowledge that the atomistic model employed in this work represents a simplified grain boundary/defect configuration compared to the structurally complex polycrystalline ZnO in experiments, which contains a distribution of grain boundaries, defects, and local environments. However, the purpose of the model is not to reproduce the full structural complexity, but to capture the essential physicochemical interactions governing the sensing mechanism.

To ensure clarity for readers, we have modified both the manuscript and the Supplementary Information to explicitly highlight that, while the model is simplified, it effectively captures the key interactions responsible for H_2S sensing. Specifically, the simplified model enables a clear evaluation of the adsorption behavior, charge transfer characteristics, and electronic structure modulation associated with oxygen vacancies and grain boundary regions, which are the key functional sites identified experimentally. These fundamental interactions are expected to be generally applicable across different local configurations. The calculations reveal that oxygen vacancy sites and grain boundary regions facilitate enhanced adsorption and charge transfer, leading to modulation of the local electronic structure, which plays a crucial role in the sensing process. These trends are in strong agreement with the experimentally observed changes in interfacial electronic states (as evidenced by UPS) and the correlation between grain boundary density and sensing performance.

While the model does not explicitly account for all structural variations found in real polycrystalline materials, it effectively captures the dominant physicochemical interactions that govern the sensing mechanism. The consistency between the theoretical results and experimental data supports the validity of using simplified models to describe complex interfacial systems at a mechanistic level. This agreement provides confidence that the simplified model provides a physically meaningful and representative description of the key mechanisms involved in H_2S sensing, allowing for a reliable interpretation of the experimental results.

Therefore, we believe that, despite the simplifications made in the model, it still provides valuable insights into the essential interactions and offers a reliable basis for understanding the experimental observations. To further clarify this, we have explicitly stated in the manuscript and SI that the simplified model, though idealized, captures the most important physical processes involved.

The associated revised discussion has been added to the Supplementary Information (Discussion corresponding to Supplementary Figures 33–34) and explicitly referenced in the revised manuscript (Page 14, Line 446-455) and are also highlighted in red accordingly.

Revised parts of manuscript are as following:

To further investigate the influence of defects on sensing performance, we performed DFT calculations to evaluate the adsorption of H_2S at both oxygen and zinc vacancy sites. The results confirm that oxygen vacancies play a more significant role in H_2S adsorption, and full data and analysis are given in Supplementary Fig. 34 and Tab. 5-6. Although the model represents an idealized local configuration rather than the full structural complexity of polycrystalline ZnO, it effectively captures the key physicochemical interactions, such as charge transfer and electronic structure modulation, that govern the sensing behavior. The trends obtained from the calculations, including the preferential adsorption at oxygen vacancies and the correlation between grain boundary density and sensing performance, are in strong agreement with the experimental observations, providing confidence that the simplified model captures the dominant physics underlying the sensing process.

Revised parts of SI are as following:

To establish a theoretical foundation for the experimental observations, DFT simulations were performed to systematically investigate the influence of grain boundary density on sensor performance. Four distinct ZnO configurations were modeled, corresponding to grain boundary densities of 0, 0.5, 1, and 2 (denoted as GB0, GB0.5, GB1, and GB2, respectively) (Supplementary Fig. 33). The simplified model, although not fully representative of the structural complexity of polycrystalline ZnO, captures the key interactions occurring at grain boundaries and vacancy sites. The Figure of Merit (FOM) analysis revealed significant variations in sensing characteristics across different configurations, and comparative evaluation of FOM differences before and after H_2S exposure demonstrated that the GB1 system exhibited minimal FOM variation, indicating optimal Schottky barrier characteristics. These computational findings are in excellent agreement with experimental results, confirming that an intermediate grain boundary density (GB1) maximizes sensing performance toward trace H_2S detection.

Both excessive (GB2) and insufficient (GB0, GB0.5) grain boundary densities were found to elevate the Schottky barrier height, thereby impeding electron transport and degrading sensing performance. This non-monotonic relationship between grain boundary density and sensor performance establishes an important design principle for optimizing metal oxide gas sensors. While the model does not explicitly account for all structural variations found in real polycrystalline materials, it provides a physically meaningful and representative description of the dominant mechanisms governing the sensing process. The agreement between the theoretical results and experimental data supports the validity of the simplified model in describing the essential interactions responsible for H_2S sensing.

To investigate the influence of oxygen and zinc vacancies on H_2S adsorption and sensor performance, we performed DFT calculations to evaluate the adsorption energies and charge distributions at both vacancy sites (Supplementary Fig. 34). Although the model simplifies the structural complexity of polycrystalline ZnO, focusing on local grain boundary and vacancy sites, it effectively captures the key interactions governing the sensing mechanism. Specifically, we find that H_2S preferentially adsorbs at oxygen vacancy sites, with greater charge transfer to the material compared to zinc vacancies. This is consistent with experimental findings that oxygen vacancies play a more significant role in H_2S adsorption.

The adsorption energies for H_2S at oxygen and zinc vacancy sites are -0.3096 eV and -0.1304 eV, respectively (Supplementary Tab. 5). The differential charge density analysis (Supplementary Fig. 34c and f) provides further insight into the charge transfer upon adsorption. As shown in the differential charge density, the blue region represents the depletion of charge, while the yellow region indicates the accumulation of charge. Notably, for the oxygen vacancy, a large blue region is observed, indicating a significant amount of charge is transferred from the H_2S molecule to the material surface. This suggests that oxygen vacancies are the primary adsorption sites, where a large amount of charge is accumulated at the interface. In contrast, the zinc vacancy shows a smaller blue region, indicating a lesser degree of charge transfer, further supporting the conclusion that oxygen vacancies play a more prominent role in H_2S adsorption.

The results of Bader charge analysis (Supplementary Tab. 6) confirm that oxygen vacancies contribute more charge to the adsorbed H_2S molecule (-0.1658), compared to zinc vacancies (-0.1370), highlighting the role of oxygen vacancies as the primary adsorption site.

These findings support the experimental observation that ZMA450, despite having a higher oxygen-vacancy concentration, exhibits weaker sensing performance due to its smaller grain size and higher grain-boundary density, which limit charge transport and reduce response efficiency. The grain boundary limitations in ZMA450 hinder its ability to effectively translate surface charge modulation into a macroscopic conductance change, thus impairing its sensing performance.

Comment 3. The authors emphasize a detection limit of 0.328 ppb. Could you please provide direct exposure test data for H_2S at sub-ppb levels (e.g., 0.1 ppb, 0.3 ppb, 0.5 ppb), including complete response/recovery curves and multi-cycle repeatability results, to experimentally demonstrate the sensor's actual ultra-low concentration detection capability, rather than relying solely on theoretical calculations?

Reply: We thank the reviewer for this important and constructive suggestion. We fully agree that direct exposure measurements at ultra-low H_2S concentrations are essential to complement the calculated LOD with direct experimental evidence.

In the original manuscript, the value of 0.328 ppb was reported as a calculated LOD, derived from the baseline noise and the slope of the response–concentration calibration curve, and the corresponding calculation method was explicitly described. To further strengthen the experimental support, additional direct exposure tests were carried out in the low-concentration range of 10–50 ppb H_2S.

Although the reviewer suggested sub-ppb measurements (e.g., 0.1, 0.3, and 0.5 ppb), under the requirement of maintaining full consistency with all other sensing conditions and ensuring the accuracy and reliability of gas delivery, the current gas-mixing setup could reproducibly achieve a minimum concentration of 10 ppb. We therefore selected this range (10, 20, 30, 40, 50 ppb) as the experimentally accessible low-concentration window for direct validation.

The newly added results, presented in Supplementary Fig. 7, include complete dynamic response/recovery curves for 10–50 ppb H_2S, as well as 8-cycle repeatability measurements at 30 ppb, which clearly demonstrate distinguishable, stable, and reproducible sensing behavior in the ppb-level regime. These data provide direct experimental validation of the sensor's practical ultra-low-concentration sensing capability.

At the same time, we have revised the manuscript to explicitly clarify that 0.328 ppb corresponds to a calculated LOD, rather than a directly demonstrated minimum exposure concentration. The combination of the calculated sub-ppb LOD and the experimentally validated ppb-level response provides a more rigorous and practically meaningful evaluation of the sensor performance.

These new results and the associated discussion have been added to the Supplementary Information (Supplementary Fig. 7) and explicitly referenced in the revised manuscript (Page 7-8, Line 235-238) and are also highlighted in red accordingly.

Revised parts of manuscript are as following:

Comparative analysis of sensitivity and LOD under N_2 and air atmospheres revealed optimal performance in air, with a calculated LOD of 0.328 ppb for H_2S detection (Fig. 2e, details shown in Supplementary Fig. 6). Complementary direct exposure measurements at 10–50 ppb H_2S further support reliable sensing in the ppb-level regime (details shown in Supplementary Fig. 7).

Revised parts of SI are as following:

To complement the calculated LOD, direct H_2S exposure measurements were performed at low concentrations of 10, 20, 30, 40, and 50 ppb, corresponding to the lowest concentrations that could be reproducibly generated under the current gas-mixing conditions.

As shown in Supplementary Fig. 7, the sensor exhibits clear and distinguishable response–recovery behavior across the entire tested concentration range. Notably, even at 10 ppb, the response signal remains significantly above the baseline noise level, indicating reliable signal discrimination at the ppb level. Furthermore, the sensor response shows a consistent increase with increasing H_2S concentration, demonstrating a well-defined concentration-dependent behavior in the low-concentration regime.

To evaluate repeatability, 8 consecutive response–recovery cycles were conducted at 30 ppb. The results show highly reproducible response amplitudes and stable baseline recovery without noticeable signal drift, confirming the robustness and stability of the sensor under repeated low-concentration exposure.

These results provide direct experimental validation of the sensor's ppb-level sensing capability. It should be noted that the previously reported value of 0.328 ppb represents a calculated LOD derived from baseline noise and calibration sensitivity, whereas the present measurements define the experimentally accessible detection range under practical operating conditions.

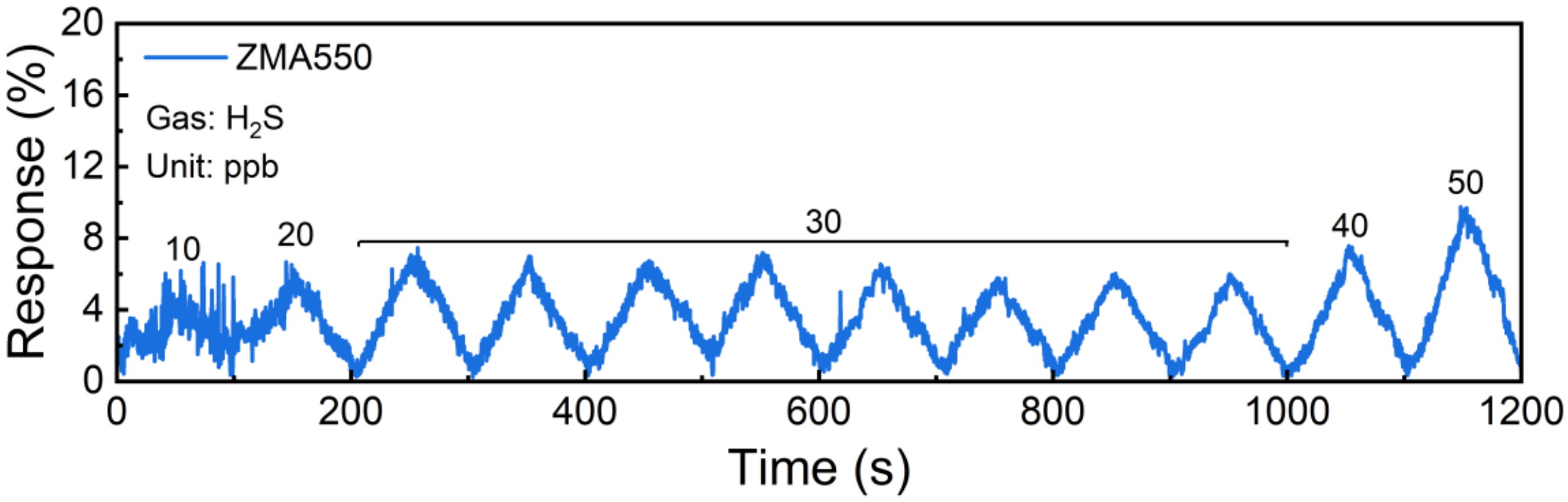

Supplementary Fig. 7 | Dynamic response and recovery curves of the ZMA550 sensor exposed to varying lower H_2S concentrations (10-50 ppb) under air condition.

To Reviewer: 3

General comments: Author has incorporated all the queries.

Reply: We sincerely thank the reviewer for the positive assessment and for recognizing the revisions made in response to the comments. We greatly appreciate the constructive feedback provided throughout the review process, which has helped us to significantly improve the quality and clarity of the manuscript.

A point-by-point response to the reviewers' comments

We sincerely thank the reviewer for the careful evaluation of our revised manuscript and for the positive assessment.

Reviewer #1

Comment: "The authors have answered all the issues, and it is ready to be accepted."

Response: We greatly appreciate the reviewer's positive feedback and acknowledgment that all concerns have been satisfactorily addressed. We are grateful for the constructive comments provided during the review process, which have helped us improve the quality and clarity of the manuscript. We thank the reviewer for the time and effort devoted to evaluating our work and for recommending the manuscript for acceptance.
